# Enzymatic synthesis of benzylisoquinoline alkaloids using a parallel cascade strategy and tyrosinase variants

**DOI:** 10.1038/s41467-022-33122-1

**Published:** 2022-09-16

**Authors:** Yu Wang, Fabiana Subrizi, Eve M. Carter, Tom D. Sheppard, John M. Ward, Helen C. Hailes

**Affiliations:** 1 Department of Chemistry, Christopher Ingold Building, University College London, 20 Gordon Street, London, WC1H 0AJ UK; 2 Department of Biochemical Engineering, Bernard Katz Building, University College London, London, WC1E 6BT UK

**Keywords:** Chemistry, Biotechnology

## Abstract

Benzylisoquinoline alkaloid derived pharmaceuticals are widely applied in modern medicines. Recent studies on the microbial production of benzylisoquinolines have highlighted key biological syntheses towards these natural products. Routes to non-natural benzylisoquinolines have been less explored, particularly halogenated compounds which are more challenging. Here, we show the use of a tyrosinase, tyrosine decarboxylase, transaminase, and norcoclaurine synthase which are combined in a parallel cascade design, in order to generate halogenated benzylisoquinoline alkaloids in high enantiomeric excess. Notably, mutagenesis studies are applied to generate tyrosinase mutants, which enhance the acceptance of halogenated tyrosines for use in the biocatalytic cascades developed.

## Abstract (web summary)

Pharmaceuticals derived from benzylisoquinoline alkaloids find application in modern medicine. Here, the authors report the design of a parallel enzymatic cascade to generate halogenated benzylisoquinoline alkaloids, that included engineering of tyrosinase mutants to enhance substrate capabilities.

## Introduction

Benzylisoquinoline alkaloids (BIAs) play an important role in the field of natural products due to their pharmaceutical properties. The use of plants containing BIAs dates back to 1500 BC, when the ancient Egyptians used opium as a painkiller^1^. Modern medicine revealed the biologically active components as codeine and morphine, both of which exhibit analgesic properties^2,3^. Recent studies on alkaloids have largely accelerated the use of BIA-derived pharmaceuticals, such as the anti-cancer agents noscapine and berberine^4,5^, anti-HIV agents coclaurine and norcoclaurine^6^, and anti-inflammatory drugs berbamine and coptisine^7,8^. Indeed, amongst all approved small-molecule based new drugs from 1981 to 2019, over 30% were based on natural products and 82% of these were semi-synthesised from natural product skeletons^9^

**Fig. 1:**
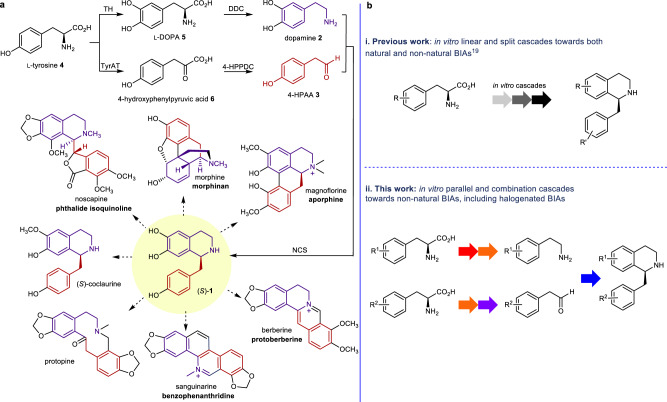
Biosynthetic and biocatalytic routes to BIAs and a parallel cascade adopted in this work. **a** Biosynthetic BIA pathway in plants and examples of the alkaloid formed. L-Tyrosine **4** is converted to L-DOPA **5** by tyrosine hydroxylase (TH), then to dopamine **2** by DOPA decarboxylase (DDC). In a parallel pathway, **4** is converted into 4-HPAA **3** via 4-hydroxyphenylpyruvic acid **6** by tyrosine aminotransferase (TyrAT) and 4-hydroxyphenylpyruvate decarboxylase (4-HPPDC). Compounds **2** and **3** are condensed by the Pictet-Spenglerase norcoclaurine synthase (NCS), generating (*S*)-norcoclaurine, (*S*)-**1**, which is converted into BIAs, such as morphinans, aporphines and protoberberines^11,12^. **b** Cascade reactions to BIAs inspired by nature: i. Previous work on in vitro assembly of natural and non-natural BIAs from single amino acid substrate. ii. The in vitro parallel cascade to non-natural BIAs reported in this study.

Naturally-derived BIAs include around 2500 known compounds derived from the key intermediate (*S*)-norcoclaurine, (*S*)-**1**^10^. In plants, (*S*)-**1** is produced by the condensation between dopamine **2** and 4-hydroxyphenylacetaldehyde (4-HPAA) **3** via a norcoclaurine synthase (NCS) mediated Pictet-Spengler reaction. Both **2** and **3** are derived from L-tyrosine **4**, via L-DOPA **5** and 4-hydroxyphenylpyruvic acid **6** by tyrosine hydroxylase (TH)/DOPA decarboxylase (DDC) and tyrosine aminotransferase (TyrAT)/4-hydroxyphenylpyruvate decarboxylase (4-HPPDC) respectively (Fig. 1a together with some examples of alkaloids formed)^11,12^.

The reconstruction of heterologous BIA pathways into microbes is an exciting area of research, with significant progress being made in recent years: the in vivo BIA pathway in *Escherichia coli* and *Saccharomyces cerevisiae* have been reported with the production of natural BIAs^13–18^. Pathways to BIAs have also been achieved using enzyme cascade reactions in vitro in high yields^19^. The NCS Pictet-Spenglerase reaction is the key step to construct the BIA skeleton in such approaches^13,19^. Previous work has established the NCS mechanistic requirement for the arylethylamine *meta*-hydroxyl group, which binds into the NCS active site, and is deprotonated to enable ring formation^20,21^. We have reported a recombinant tyrosinase from *Candidatus* Nitrosopumilus salaria BD31 (*Cn*TYR) able to effectively convert **4** to L-DOPA **5** without the requirement for expensive co-factors, generating the required *meta*-hydroxyl moeity^19^. Additionally a tyrosine decarboxylase from *Enterococcus faecalis* DC32 (*Ef*TyrDC) was developed for the decarboxylation of **5** and these were used in cascades to generate BIAs (Fig. 1b). However, such cascades do not have to follow the natural pathway starting from L-tyrosine **4**. They can be designed combining other enzymes in different orders, or other substrates, to enable the production of non-natural BIAs.

Here, we show the design of a route to differentially halogenated non-natural BIAs through a parallel cascade and combination strategy, in which arylethylamines and arylacetaldehydes are derived from two different amino acids. To achieve this, a TYR is used to provide the necessary *meta*-hydroxyl group and a TyrDC to produce the amine from the amino acid. Also, a transaminase (TAm) for aldehyde formation, and finally NCS are used for BIA formation (Fig. 1b). Importantly, as well as defining the parallel cascade sequence, to extend the enzyme cascades into other substrate capabilities, *Cn*TYR variants are generated with good monophenolase activities towards halogenated tyrosines, enabling the production of a range of halogenated BIAs.

## Results and discussion

### Designing and implementing parallel cascades

**Fig. 2:**
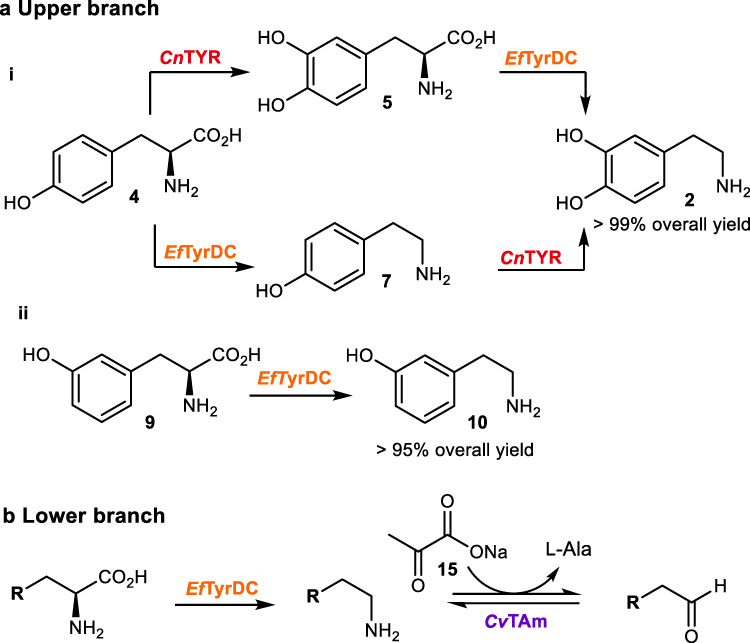
Designing the parallel cascade. **a** The upper branch to arylethylamines. i. Dopamine **2** was generated in a one-pot two step cascade reaction from L-tyrosine **4** combining *Cn*TYR and *Ef*TyrDC; ii. *Meta*-tyramine **10** was produced from *meta*-tyrosine **9** by *Ef*TyrDC; *Reaction conditions* i: a 1 mL of reaction mixture (50 mM HEPES, pH 5.5) containing **4** (2.5 mM, 1 equiv.), 0.4 mgmL^−1^ cell lysate of *Cn*TYR (containing 21% of recombinant protein), 0.4 mgmL^−1^ cell lysate of *Ef*TyrDC (containing 25% of the recombinant protein), sodium ascorbate **8** (4 equiv.), CuSO_4_ (40 μM) and PLP (1.25 mM) at 25 °C, 250 rpm for 6 h and quenched by adding 1 μL of trifluoroacetic acid (TFA). ii: a 1 mL of reaction mixture (50 mM HEPES, pH 7.5) containing **7** (2.5 mM, 1 equiv.), 0.4 mgmL^−1^ cell lysate of *Ef*TyrDC (containing 25% of the recombinant protein), sodium ascorbate **8** (1 equiv.) and PLP (1.25 mM) at 25 °C, 250 rpm for 6 h, and quenched by adding 1 μL of TFA). **b** The lower branch to the aldehydes. See Table 1 for reaction conditions.

To generate the required arylethylamine moiety in the upper branch of the parallel cascade (Fig. 2a), the first step when using L-tyrosine **4** or analogues is the addition of a *meta*-hydroxyl group. In this one-pot process in initial studies, dopamine **2** was formed in a quantitative yield from **4**. Tyramine **7** could also be formed as an intermediate with both enzymes, if the decarboxylation occurs prior to the hydroxylation. Sodium ascorbate **8** was added to prevent the oxidation of substrates and products, and enzyme lysates were used for ease of production and as these are normally used in industry. Studies confirmed that *Ef*TyrDC could also be used with *meta*-L-tyrosine **9** to produce *meta*-tyramine **10** in >95% yield^19^.

For aldehyde production in the lower half of the cascade (Fig. 2b and Table 1), readily available starting materials were required, and amino acids were an ideal starting point. Arylacetaldehydes are particularly challenging to prepare as they are oxidatively sensitive and can self-condense via aldol reactions. Here, to produce aldehydes, the transaminase from *Chromobacterium violaceum* DSM30191 (*Cv*TAm) was selected, in combination with *Ef*TyrDC, as they have both been reported to have broad substrate tolerances^19,22–24^. Amino acids **4**, **5**, **9**, 3-F-L-tyrosine **11**, 3-Cl-L-tyrosine **12** and 3-I-L-tyrosine **13** were investigated for aldehyde production and the amine 3-phenyl-1-propylamine **14** was also used. Decarboxylation (other than for **14**) was followed by deamination with sodium pyruvate **15** as the amine acceptor and pyridoxal 5′-phosphate (PLP) as cofactor. All of the selected substrates were readily accepted by *Ef*TyrDC and *Cv*TAm, giving compounds **16**–**22** in overall conversions of 70–95% (Table 1). Enzyme lysates were used in all cases.

**Table 1:** Lower branch conversion of amino acids and 14 into aldehydes 16–22

*Reaction conditions*: a 1 mL reaction mixture (50 mM HEPES, pH 7.5) containing amino acid substrate **4**, **5**, **9**, **11**–**13** and amine **14** (10 mM, 1 equiv.), 0.4 mgmL^−1^ *Ef*TyrDC cell lysate (containing 25% of recombinant protein), 0.2 mgmL^−1^ cell lysate of *Cv*TAm (containing 80% of recombinant protein), **8** (1 equiv.), PLP (5 mM) and **15** (1 equiv.) at 37 °C, for 6 h and quenched by adding 1 μL of TFA. Conversions were determined by HPLC analysis at 280 nm based on starting material consumption.

**Fig. 3:**
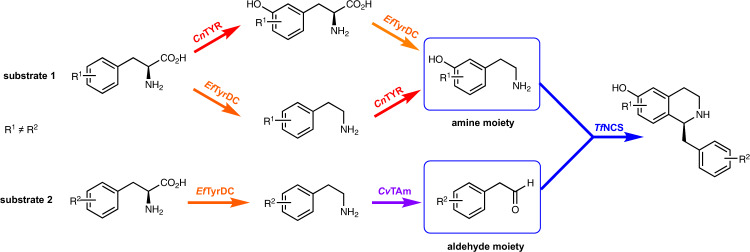
Parallel cascades with *Cn*TYR, *Ef*TyrDC, *Cv*TAm, and *Tf*NCS to non-natural BIAs. The amine moiety was derived from an amino acid (substrate 1) using a *Cn*TYR-*Ef*TyrDC cascade. In a parallel pathway, another amino acid (substrate 2) was converted into the aldehyde moiety by a *Ef*TyrDC-*Cv*TAm cascade. Non-natural BIAs were then synthesised using *Tf*NCS.

The final step in this five enzyme-step parallel cascade was combining the amine and aldehyde moieties with addition of a wild-type (WT) *Tf*NCS enzyme that has displayed good substrate tolerances^25,26^. Overall, two amino acids were used as starting materials (other than when utilising **14**), one for the amine generation (upper branch) and the other for aldehyde formation (lower branch). Nine cascades were constructed, the first five using L-tyrosine **4** (Fig. 3, Table 2, entries 1–5) with *Cn*TYR and *Ef*TyrDC for amine production (25 °C, 6 h). In parallel, *meta*-L-tyrosine **7** (Fig. 3, Table 2, entry 1) was used with *Ef*TyrDC and *Cv*TAm (37 °C, 6 h), followed by the addition of *Tf*NCS (37 °C, 8 h). This produced the non-natural BIA (*S*)-**23** in 47% isolated yield (82% yield by analytical HPLC) and 90% enantiomeric excess (*ee*). The isolation of BIAs, as reported previously can result in the loss of product, so the conversion yields are also given (determined by HPLC against product standards)^19,27^. The cascade to (*S*)-**24**^28^ was achieved with the aldehyde formed from **14** (Table 2, entry 2) using *Cv*TAm, giving (*S*)-**24** in 14% isolated yield (21% yield by HPLC) and 90% *ee*. The lower yield of (*S*)-**24** suggested that the wild-type *Tf*NCS may not accept phenyl propionaldehyde as readily as arylacetaldehydes. Halogenated tyrosines were also adopted into the cascades where **11**, **12** and **13** (Table 2, entries 3–5) were converted into the corresponding halogenated arylacetaldehydes **19**–**21**, which were then condensed with the amine using *Tf*NCS, forming halogenated BIAs (*S*)-**25**–**27** in 40–42% isolated yield (84–86% yields by analytical HPLC) and up to 96% *ee*.

**Table 2:** Use of the parallel cascades to produce BIAs 23–31

^a^Yields were determined by HPLC analysis against products standards. ^b^For preparative-scale reactions, products were purified by preparative HPLC or extraction method (Supplementary Information). ^c^*ee*s were determined by chiral HPLC. *Reaction conditions*: Details are specific to each cascade. E.g. (entry 1) - 50 mL reaction mixture A (RMA) - HEPES (50 mM, pH 5.5), **4** (2.5 mM, 1 equiv.), *Cn*TYR and *Ef*TyrDC (10% (v/v) lysates), **8** (4 equiv.), PLP (0.5 equiv.), CuSO_4_ (40 μM) at 25 °C for 6 h. A 50 mL reaction mixture B (RMB) - HEPES (50 mM, pH 7.5), **9** (7.5 mM, 3 equiv.), *Ef*TyrDC and *Cv*TAm (10% and 5% (v/v) lysates, respectively), **8** (3 equiv.), PLP (1.25 mM), **15** (3 equiv.) at 37 °C for 6 h. RMA + RMB were combined, *Tf*NCS (10% (v/v) lysates) added, and the reaction run at 37 °C for 8 h.

In a similar fashion, four parallel cascades starting from *meta*-L-tyrosine **9** (Table 2, entries 6–9) to produce the amine moiety were established. In the first of these approaches the starting materials were swapped, compared to entry 1, with L-tyrosine **4** producing the aldehyde 4-HPAA **3**, giving (*S*)-**28** in 42% isolated yield (76% yield by HPLC) and 92% *ee*. Similarly, halogenated L-tyrosines **11**–**13** (Table 2, entries 7–9) were used to produce the aldehyde and matched with the amine component derived from **9**, giving halogenated BIAs (*S*)-**29**–**31** in 27–35% isolated yield (58–68% yield by analytical HPLC). The *ee* of (*S*)-**29** was 85%, slightly lower than for (*S*)-**30** (95% *ee*) and (*S*)-**31** (91% *ee*). In general, lower yields for the halogenated BIAs (*S*)-**29**–**31** derived from **9** were noted, compared to BIAs (*S*)-**25**–**27** generated from **4**, presumably reflecting the more activated dopamine catechol ring that interacts with Lys122 in the NCS active site.

Some halogenated BIAs have been reported previously and were produced via a chemoenzymatic route. Maresh et al.^29^ utilised the oxidant NaOCl for aldehyde generation to produce **26** and **27**. However, enzymes can react under milder and more environmentally friendly conditions and only relative rates with *Tf*NCS were described. In addition, in recent work on the synthesis of noscapine in yeast, some C-8 halogenated BIAs were detected by liquid-chromatography mass spectrometry analysis (LC-MS)^30^. Here, the parallel in vitro cascades highlight a versatile amino-acid derived route to halogenated BIA synthesis in high *ee*s.

The tyrosinase (*Cn*TYR) used in the enzyme cascades was found to be limiting as L-tyrosine **4** and tyramine **7** were well accepted but 3-F-L-tyrosine **11** was poorly accepted^19^. For wider applications it was desirable to expand the substrate range, however, the iodo-analogue 3-I-L-tyrosine **13** has been reported to be a ‘mixed type’ inhibitor (a competitive and non-competitive inhibitor) for some tyrosinases^31^. 2-Chlorophenol has also been described to act as a competitive inhibitor towards tyrosinases^32^. Therefore, to expand the capability of these cascades using tyrosinases, mutagenesis of *Cn*TYR was investigated.

### CnTYR mutagenesis

The narrow substrate range of *Cn*TYR is likely due to steric interactions in the active site, precluding access by the halogenated tyrosines. To probe this, several *Cn*TYR variants were proposed based on the DNA alignment of *Cn*TYR with reported engineered tyrosinase variants described by Kanteev et al.^33^ (G63L, E185L, N201A, and N201D, Supplementary Fig. 87a) and Davis et al.^34^, (N132A/L289V, S161I/L163Y, Supplementary Fig. 87b) respectively. Preliminary global docking studies were initially carried out using AutoDock Vina^35^ (Supplementary Methods 10), substrates **4**, **9**, and halogenated tyrosines **11** and **12** to investigate the potential of these variants towards halogenated tyrosines. Using the crystal structure of *Bm*TYR (PDB code: 3NPY)^36^ as a template, homology modelling (SWISS-MODEL)^37^ was used to develop a model of wild-type *Cn*TYR. *Cn*TYR variants were generated in silico using Chimera^38–40^. Residues at 63, 185 and 201 positions of *Cn*TYR were all located at the entrance of the catalytic site. G63 is already a smaller residue so was not modified (Supplementary Fig. 88a), while E185 was not modified due to concern of its remote distance to the catalytic site (Supplementary Fig. 87b). The N201 is a conserved residue in tyrosinases, and is believed to be important for stabilisation of the orientation of the nearby H200 imidazole for Cu coordination; mutation in previous work to Ala or Asp reduced activities. Here, the variant N201S was generated, with the rationale that a switch from the conserved polar residue N to S, might still enable stabilisation of the nearby His residue (Supplementary Fig. 87c). Residues at N132/L289 and S161/L163 of *Cn*TYR are located at outer loops (Supplementary Fig. 87d, e). According to Davis et al.^34^, variant RV145 (equivalent to N132A/L289V) was reported to show a better tolerance towards 4-halophenols and therefore N132A/L289V was also selected for further investigation. The target genes of the two variants were codon optimised for *E.coli* expression and synthesised (Eurofins^TM^). To confirm activities, the *Cn*TYR variants N132A/L289V and N201S were first screened against the tyrosine analogues, including L-tyrosine **4**, tyramine **7**, *meta*-L-tyrosine **9**, 3-F-L-tyrosine **11**, 3-Cl-L-tyrosine **12**, 3-I-L-tyrosine **13**, 3-NH_2_-L-tyrosine **32**, 3-NO_2_-L-tyrosine **33**, *meta*-tyramine **10**, octopamine **34**, synephrine **35**, 4-(2-aminoethyl)aniline **36**, 4-F-L-phenylalanine **37**, 4-Cl-L-phenylalanine **38**, 4-Br-L-phenylalanine **39**, 4-OMe-L-phenylalanine **40**, 4-NH_2_-L-phenylalanine **41** and 4-NO_2_-L-phenylalanine **42** (see Supplementary notes 1 for all structures). A rapid colorimetric screen was used based on the oxidation of **4** and analogues, resulting in the formation of black quinone products (Supplementary Fig. 90a)^41^. Reactions were performed at 25 °C for 3 h using enzyme lysates and the results indicated (Supplementary Fig. 90b) that variant N132A/L289V gave no oxidised analogues, suggesting it was inactive. HPLC analysis confirmed that no oxidised products had been formed. Variant N201S, by comparison, showed strong activities towards the natural substrates L-tyrosine **4** and tyramine **7**. Interestingly, the *meta*-L-tyrosine **9** and 3-Cl-L-tyrosine **12** reactions also turned completely black and with 3-I-L-tyrosine **13** a grey coloration was formed. The substituted phenylalanines **37**–**42** and **33** did not appear to be accepted by either the wild-type *Cn*TYR or variant N201S.

Both the wild-type *Cn*TYR and the variant N201S gave full consumption of L-tyrosine **4** and tyramine **7**, and it was noted that reactions with N201S turned black ~10-times faster than with the wild-type *Cn*TYR (Table 3). The wild-type *Cn*TYR gave conversions for 3-F-L-tyrosine **11**, 3-NH_2_-L-tyrosine **32**, octopamine **34**, synephrine **35**, and 4-(2-aminoethyl)aniline **36** of 24–51% within 3 h, while the N201S gave >90% conversions with those substrates. Importantly, the wild-type TYR did not accept *meta*-L-tyrosine **9**, 3-Cl-L-tyrosine **12**, 3-I-L-tyrosine **13**, and *meta*-tyramine **10**, while the N201S variant gave conversions of 55–64% towards **9**, **12** and **10** and 10% for **13**.

**Table 3:** Comparison of the conversion yields of the wild-type *Cn*TYR and *Cn*TYR_N201S towards different substrates

*Reaction conditions*: HEPES buffer (50 mM, pH 5.5), substrates (2.5 mM), CuSO_4_ (5 μM) and enzyme cell lysates (10% (v/v)) in a total volume of 500 μL were run at 25 °C for 3 h. Reactions were quenched by adding 1 μL trifluoroacetic acid. Conversions were analysed using analytical HPLC at 280 nm based on substrate consumption.

**Fig. 4:**
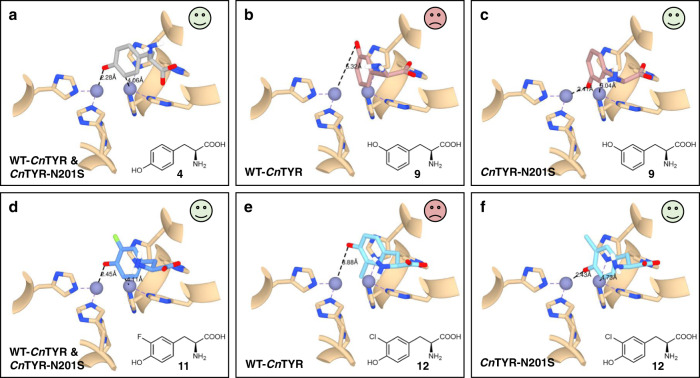
Molecular docking studies with WT-*Cn*TYR and the variant N201S. WT-*Cn*TYR and the variant N201S (homology modelled) with L-tyrosine **4**, *meta*-L-tyrosine **9** and halogenated tyrosines **11** and 12 using AutoDock Vina^35^. **a** Docking of L-tyrosine **4** with the wild-type (WT) *Cn*TYR and *Cn*TYR-N201S: L-tyrosine **1** fits well into the active sites of WT-*Cn*TYR and *Cn*TYR-N201S. **b** Docking of *meta*-L-tyrosine **9** with the WT-*Cn*TYR: **9** can fit into the active site of WT-*Cn*TYR but not in a productive orientation. **c** Docking of **9** with *Cn*TYR-N201S: **9** fits well into the active site of *Cn*TYR-N201S. **d** Docking of 3-F-L-tyrosine **11** with WT-*Cn*TYR and *Cn*TYR-N201S: **11** fits well into the active site of both. **e** Docking of 3-Cl-L-tyrosine **12** with WT-*Cn*TYR: **12** can fit into the active sites WT-*Cn*TYR but not in a productive orientation. **f** Docking of **12** with *Cn*TYR-N201S: **12** fits well into the active sites of *Cn*TYR-N201S. The functional histamine residues in the tyrosinase active sites and substrates are shown in stick and ribbon forms. Enzyme residues are shown in tan. Compounds **4**, **9**, **11**, and **12** are shown in grey, rose, blue, and light blue, respectively. The model of WT-*Cn*TYR was generated by homology modelling (SWISS-MODEL)^37^ using the crystal structure of *Bm*TYR (PDB code: 3NPY)^36^ as a template. *Cn*TYR variants were generated and homology modelled by Chimera^38–40^.

Preliminary molecular docking studies with L-tyrosine **4** and 3-F-L-tyrosine **11** suggested they readily fitted into the active sites of both the wild-type *Cn*TYR and N201S, with the *para*-hydroxyl group bound to one of the Cu^2+^ ions in the di-copper centre of tyrosinases, allowing the *meta*-carbon to be hydroxylated by the other Cu^2+^ (Fig. 4a, d). In addition, *meta*-L-tyrosine **9** and 3-Cl-L-tyrosine **12** could access the enzyme active sites but not in a productive orientation, which agreed with the reported competitive inhibitor behaviour of some substrates with tyrosinases. With the wild-type *Cn*TYR, **12** was orientated with the *para*-hydroxyl group and *meta*-chloro group facing towards to the di-copper centre, with the other *meta*-carbon faced away from the di-copper centre which can then not be hydroxylated (Fig. 4e). Similarly, the *meta*-hydroxyl group of **9** faced away from the di-copper centre in the wild-type *Cn*TYR (Fig. 4b). However, with variant N201S, 3-Cl-L-tyrosine **12** was rotated with the *meta*-chloro group facing away from the di-copper centre, while the *para*-hydroxyl group and the other *meta*-carbon faced towards to the catalytic copper. Therefore, here the *para*-hydroxyl group can bind to one of the coppers and the *meta*-carbon near to the copper centre can be hydroxylated (Fig. 4f). Similarly, substrate **9** was orientated with the *meta*-hydroxyl group facing towards to the Cu^2+^ ion, so the nearby *ortho*-carbon can be hydroxylated (Fig. 4c) by the variant N201S. Further docking experiments suggested that the smaller size of Ser201 compared to Asn201 may allow the substrates **9** and **12** to be oriented into productive conformations (Supplementary Fig. 89a–c for substrates **9** and 89d-f for substrates **12**).

Kinetic studies with *Cn*TYR-N201S revealed the apparent kinetic parameters *K*_*m,*app_. and *k*_cat,app_. towards L-tyrosine **4** were 1.89 mM and 182.9 s^−1^ (*k*_cat,app_./*K*_*m,*app_. 9.68 × 10^4^ s^−1^M^−1^) and the corresponding values for tyramine **7** were 1.78 mM and 197.5 s^−1^ (*k*_cat,app._/*K*_*m,*app_. 1.11 × 10^5^ s^−1^M^−1^), respectively. Compared to the wild-type *Cn*TYR (*k*_cat,app_./*K*_m,app_. 1.78 × 10^4^ s^−1^M^−1^ towards **4** and 1.61 × 10^4^ s^−1^M^−1^ towards **7**)^19^, the catalytic efficiencies of *Cn*TYR-N201S towards both **4** and **7** were 6-fold higher. This could be due to the larger size of the entrance into the active site, enhancing access for substrates **4** and **7**, but would also make it easier for the catechol substrates to access the active site, boosting the diphenolase reaction and over-oxidation of catechols to quinones. While the highest yield of L-DOPA **5** produced by the variant with **4** was 76% (using optimised conditions), 10 equivalents of sodium ascorbate **8** were required. The large amounts of ascorbate added could cause problems during product purifications so the generation of new variants maintaining a higher monophenolase activity was explored.

**Fig. 5:**
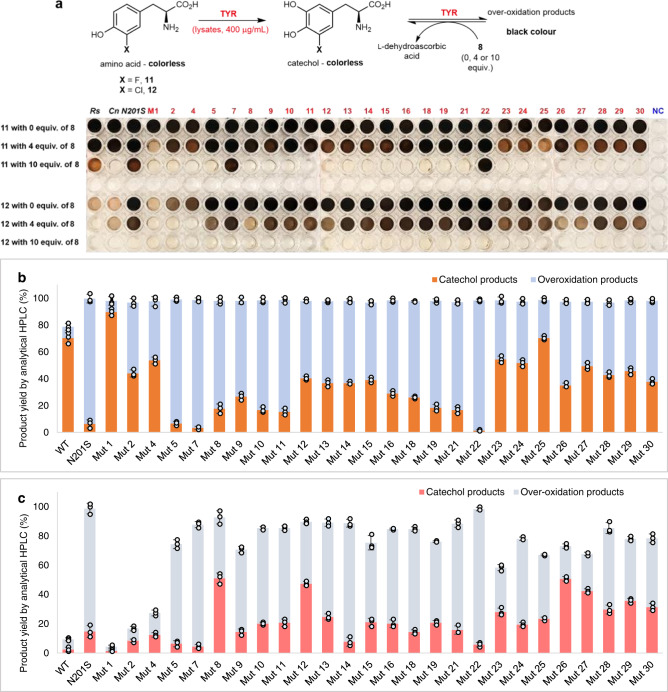
Directed evolution based on CnTYR-N201S and enzyme screening data. **a** Enzyme screening for the random mutagenized variants based on *Cn*TYR-N201S with 3-F-L-tyrosine **11** and 3-Cl-L-tyrosine **12**. *Reaction conditions*: **11** and **12** (2.5 mM, 1 equiv.), enzyme lysates (400 μgmL^−1^) and CuSO_4_ (5 μM) with 0, 4 equivalents and 10 equivalents of sodium ascorbate **8**, respectively. Reactions were performed at 25 °C for 8 h. The black colour indicates the diphenolase activities of the tyrosinases. Note: WT-*Rs*TYR (*Rs*)^19^, *Cn*TYR (*Cn*), and *Cn*TYR-N201S (N201S) lysates were used as positive controls and cell lysates of an empty plasmid as a negative control (NC). **b** Product yields and components of TYR reactions with 4 equiv. of **8** using WT-*Cn*TYR and *Cn*TYR variants towards 3-F-L-tyrosine **11**: orange bars represent the catechol product 3-F-L-DOPA **43** by HPLC against product standards, and the blue bars represent the over-oxidation products (determined by analytical HPLC with short retention times). **c** Product yields and components of TYR reactions with 4 equiv. of **8** using WT-*Cn*TYR and *Cn*TYR variants towards 3-Cl-L-tyrosine **12**: pink bars represent the catechol product 3-Cl-L-DOPA **44** by HPLC against product standards, and the grey bars represent the over-oxidation products as determined by analytical HPLC. Experiments were performed in triplicates. Error bars indicate the standard error of the triplicate reactions.

Directed evolution based on *Cn*TYR-N201S were then carried out for this purpose. Random mutagenesis was performed with *E. coli* XL 1-Red cells and twenty-six stable and positive colonies resulted after three rounds of re-transformation and colorimetric selection (see the methods section). Compounds 3-F-L-tyrosine **11** and 3-Cl-L-tyrosine **12** were screened with sodium ascorbate **8** under different concentrations (0, 4, and 10 equiv.) and used to select appropriate variants. The enzyme monophenolase activities were initially estimated based on the colorimetric assay: if the reaction turned black without **8** and slightly brown with 4 equiv. of **8**, this suggested potentially a better monophenolase activity towards the substrate. All negative controls gave rise to no colour change. Variants M1 and M23–M25 were estimated to display a better monophenolase activity towards **11**, while M8 and M26 gave higher monophenolase activities towards 3-Cl-L-tyrosine **12** (Fig. 5a).

The quantification of products generated by the selected variants with 4 equiv. of ascorbate **8** was then carried out. Variant M1 gave the highest yield of the catechol product 3-F-L-DOPA **43**, with a 90% yield by HPLC analysis (Fig. 5b), followed by variant M25 and wild-type *Cn*TYR (70% yield). Variants M8 and M26 gave the highest amount of 3-Cl-L-DOPA **44** in 55–60% yield (Fig. 5c). Variants M1, M8, M25, and M26 were sequenced as N201S/G205R/V206I, N201S/H202N, N201S/G205R and N201S/G205K, respectively. This indicated that on changing Gly205 to residues, Arg or Lys, the diphenolase activities were decreased, possibly due to steric reasons, with these groups obstructing the catechol products from re-entering the active sites. A similar effect was also observed in a previous study on the mutagenesis of *Bm*TYR: when they mutated the residues Met61, Met184, and Phe197, which are located at the entrance to the active site, to the smaller residues Leu and Ala (M61L, M184L, and F197A), the diphenolase activity was enhanced^33^. Electrostatic interactions may also be important.

It has been reported that the Asn201 residue is highly conserved among tyrosinases, however, its role is still unclear^33,34,42^. According to Kanteev et al.^33^, the Asn residue may stabilise the nearby His residue and coordinate with Cu^2+^ for its uptake, and the substitution of this to either Ala or Asp decreased *Bm*TYR activities. However, the substitution of Asn201 to Ser in *Cn*TYR increased the enzyme activities in this study. This could possibly be due to the Ser201 forming a hydrogen bond with the imidazole ring of the nearby His200 residue, orientating the His residue into a position for better Cu^2+^ uptake. Meanwhile, the smaller size of the Ser201 can allow bulkier substrates to access the active site. Although N201S is a successful candidate for the acceptance of 3-F-L-tyrosine **11** and 3-Cl-L-tyrosine **12**, preliminary docking analysis was insufficient to reveal the function of Ser201. In the future, further experiments using MD simulation studies may provide further insights. The variants generated were then used in the BIA cascades.

### Use of CnTYR variants in BIA synthesis

**Fig. 6:**
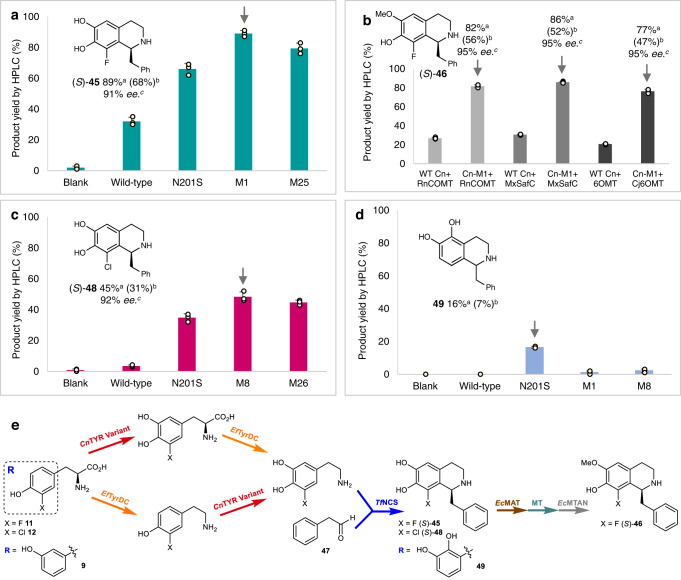
Production of alkaloids (*S*)-45,46,48 and 49 using *Cn*TYR variants. **a** Production of (*S*)-**45** using WT-*Cn*TYR, variants N201S, M1 (N201S/G205R/V206I) and M25 (N201S/G205R). **b** Production of (*S*)-**46** using WT-*Cn*TYR and M1 (N201S/G205R/V206I) together with MTs *Rn*COMT/*Mx*SafC/*Cj*6OMT, *Ec*MAT (SAM supply), and *Ec*MTAN (SAH degradation). **c** Production of (*S*)-**47** using WT- *Cn*TYR, N201S, M8 (N201S/H202N) and M26 (N201S/G205K). **d** Production of (*S*)-**48** using WT-*Cn*TYR, N201S, M1 (N201S/G205R/V206I) and M8 (N201S/H202N). **e** Enzyme cascades to alkaloids (*S*)−**45**, **46**, **48**, and **49**. *Reaction conditions*: Details are provided in the Supplementary Information and are specific to each cascade. E.g., for (*S*)-**46**, a 20 mL reaction mixture of **11** (10 mM, 1 equiv.), *Cn*TYR variants and *Ef*TyrDC lysates (10% (v/v)), **8** (4 equiv.) PLP (5 mM) and CuSO_4_ (5 μM) at 25 °C for 8 h. Compound **47** (1.5 equiv.) and *Tf*NCS lysates (10% (v/v)) were then added for another 16 h reaction. For the methylation step, *Ec*MAT lysates (10% (v/v)), *Ec*MTAN lysates (2.5% (v/v)) and *Rn*COMT/*Mx*SafC/*Cj*6OMT lysates (10% (v/v)) were added for another 8 h reaction. ^a^Yields were determined by HPLC analysis at 280 nm against products standards. Experiments were performed in triplicate, and the error bars indicate the standard error of the triplicate reactions; ^b^Isolated yields are given in parenthesis for preparative-scale reactions and products were purified by preparative HPLC or an extraction method; ^c^*ee*s were determined by chiral HPLC.

Initially the TYR were tested in cascades based upon those previously established, providing (*S*)-**45** (in 35% HPLC yield)^19^ and (*S*)-**46** (in 27% HPLC yield)^43^ from **11** using wild-type *Cn*TYR, *Ef*TyrDC, phenylacetaldehyde **47** and *Tf*NCS, and for (*S*)-**46** a methyltransferase (MT). The tyrosinase reaction previously limited the yields in these enzyme cascades. Notably, when replacing wild-type *Cn*TYR with the variants N201S, M1(N201S/G205R/V206I), and M25 (N201S/G205R), the yield of (*S*)-**45** increased to 66, 89, and 79% by HPLC analysis, respectively (Fig. 6a). The highest yield reached was 89% with the variant N201S/G205R/V206I. Meanwhile, the yield of (*S*)-**46** reached 77–86% (by HPLC analysis) using *Cn*TYR-M1 (N201S/G205R/V206I) (Fig. 6b). For the final methylation step, three *O*-MTs were trialled, the catechol-*O*-MTs from *Rattus norvegicus* (*Rn*COMT)^44^ and *Coptis japonica* (*Cj*-6-OMT)^45,46^, and SafC from *Myxococcus xanthus* (*Mx*SafC)^47^ with all giving the 6-OMe product **46** in good yields^43^. Due to the high cost of the cofactor (*S*)-adenosylmethionine (SAM), a methionine adenosyltransferase (MAT E.C. 2.5.1.6) was used to generate SAM from ATP and L-methionine, and a methylthioadenosine nucleosidase (MTAN, E.C. 3.2.2.9.) to remove the inhibitory by-product (*S*)-adenosylhomocysteine (SAH), both from *E. coli* (*Ec*)^48^. Reaction times for the tyrosinase steps were also shortened to 8 h compared to 24 h using the wild-type enzyme. A similar three enzyme cascade was then established starting from 3-Cl-L-tyrosine **12**. The *Cn*TYR variants N201S, M8 (N201S/H202N) and M26 (N201S/G205K) were investigated in the cascade and gave the chlorinated alkaloid (*S*)-**48** in 35–45% yield (Fig. 6c). Variant N201S/H202N gave the highest yield for (*S*)-**48** of 45% (by HPLC analysis) and 92% *ee*. Interestingly, when using *meta*-L-tyrosine **9** as the starting material in this three-enzyme cascade comprising *Cn*TYR-N201S, *Ef*TyrDC and *Tf*NCS, **49** was generated, indicating that *Cn*TYR-N201S hydroxylated the *ortho*- and more sterically hindered carbon of **9** (Fig. 6d), to give a non-natural alkaloid.

Meanwhile, to determine whether the yields of the halogenated alkaloids could be enhanced further, the *Tf*NCS variants, L76V, A79I, A79F, F80L, M97F, and A182I, were also investigated in the three-enzyme cascades to give **45** and **48** (Fig. 6e). The variant A79I gave the halogenated alkaloids (*S*)-**45** and (*S*)-**48** in slightly higher yields than the wild-type *Tf*NCS, in 92 and 48% yields by HPLC analysis, respectively (see SI, S12) whereas the others were similar or lower^26,49,50^. Preliminary docking studies were also performed with the wild-type and A79I-*Tf*NCS which suggested that the variant A79I folds the iminium carbon in closer proximity to the carbon *ortho*- to the F/Cl on the aromatic ring of halogenated BIA intermediates, which could account for this observation (see Supplementary Table 2, 3.90 Å for (*S*)-**45**)/3.78 Å (for (*S*)-**46** with A79I, and 4.77 Å for (*S*)-**45**)/4.68 Å for (*S*)-**46** with the wild-type).

The enzyme cascades were then extended using halogenated tyrosines as the starting materials, and for the production of aldehydes. Arylethylamines were generated from **11** and **12** as before using *Cn*TYR-M1 (N201S/G205R/V206I) and *Cn*TYR-M8 (N201S/H202N), respectively, and *Ef*TyrDC. Arylacetaldehydes were derived from *Ef*TyrDC and *Cv*TAm, and when synthesised *Tf*NCS-A79I was used for catalysing the Pictet-Spengler reactions due to the slightly higher yields noted above. Initially **11** and **12** were reacted with 2-bromo-phenylacetaldehyde **50**, giving double halogenated BIAs (*S*)-**51** and (*S*)-**52** in 83 and 16% yield by HPLC and >92% *ee*, respectively (Table 4, entries 1 and 2). The low yield of (*S*)-**52** was most likely due to the lower monophenolase activity of the *Cn*TYR variant with **12** and the effect of the more sterically hindered 3-Cl-dopamine derived intermediates in the NCS active site. More halogenated BIAs were then produced using the ‘parallel cascade’ strategy. The amine moiety was generated from **11** as before, and aldehydes produced from **11** to **13** and **4** (Table 4, entries 3–7). This gave BIAs (*S*)-**54**–**57** in 26–78% yields and >90% *ee*. Although some of the products were formed in lower yields due to the difficulties of using more sterically challenging halogenated analogues and unknown side-products, it nevertheless highlights a very valuable and flexible strategy to non-natural BIAs in high enantioselectivities.

**Table 4:** Parallel cascades with *Cn*TYR variants, *Ef*TyrDC, *Cv*TAm, and *Tf*NCS-A79I to halogenated BIAs

^a^Yields were determined by HPLC analysis at 280 nm against products standards. ^b^For preparative-scale reactions products were purified by preparative HPLC or an extraction method. ^c^*ee*s were determined by chiral HPLC. *Reaction conditions*: Details are specific to each cascade. E.g., entry 4, 50 mL reaction mixture A (RMA) - HEPES (20 mM, pH 5.0), **11** (10 mM, 1 equiv,), *Cn*TYR-M1 (N201S/G205R/V206I) and *Ef*TyrDC (10% (v/v) cell lysates), **8** (4 equiv.), PLP (0.5 equiv.), CuSO_4_ (5 μM) was stirred at 25 °C for 8 h. A 20 mL reaction mixture B (RMB) - HEPES (50 mM, pH 7.5), **11** (20 mM, 1 equiv.), *Ef*TyrDC and *Cv*TAm (10% and 5% (v/v) cell lysates, respectively), **8** (4 equiv.), PLP (1 equiv.), **15** (1 equiv.) was stirred at 37 °C for 8 h. Afterwards, RMA and RMB were combined, *Tf*NCS-A79I (10% (v/v) cell lysates) added, and the reaction stirred at 37 °C for 16 h.

In summary, *Ef*TyrDC and *Cv*TAm are capable of accepting a broad range of aromatic amino acids, and so were used here to generate aldehydes for coupling reactions using Pictet-Spenglerases. Such arylacetaldehydes are difficult to synthesise using traditional chemical routes as the aldehydes are prone to oxidations and aldol self-condensations. Enzyme cascades were then developed using parallel reaction strategies and nine non-natural BIAs were initially produced in good yields and *ee*s. Mutagenesis studies were then applied with *Cn*TYR to expand its substrate scope towards halogenated amino-acids. Several *Cn*TYR variants were generated that displayed better monophenolase activities towards 3-F-L-tyrosine **11** and accepted *meta*-L-tyrosine **9** and 3-Cl-L-tyrosine **12**, which are known inhibitors for wild-type tyrosinases. Then extended enzyme cascades using the *Cn*TYR and *Tf*NCS variants were carried out to give 14 halogenated (and 13 non-natural) BIAs in good stereoselectivities. Importantly, this is the first time that double halogenated BIAs have been reported, highlighting the abilities of enzyme cascades to ‘mix and match’ arylethylamines and aldehydes to give different BIAs. This parallel enzyme cascade strategy together with enzyme mutagenesis is a powerful synthetic approach for alkaloid synthesis.

## Methods

### Chemicals

Dopamine **2**, L-tyrosine **4**, L-DOPA **5**, tyramine **7**, sodium ascorbate **8**, 3-Cl-L-tyrosine **12**, 3-I-L-tyrosine **13**, 3-phenyl-1-propylamine **14**, sodium pyruvate **15**, 3-NH_2_-L-tyrosine **32**, 3-NO_2_-L-tyrosine **33**, octopamine **34**, synephrine **35**, 4-(2-aminoethyl)aniline **36**, 4-F-L-phenylalanine **37**, 4-Cl-L-phenylalanine **38**, 4-Br-L-phenylalanine **39**, 4-OMe-L-phenylalanine **40**, 4-NH_2_-L-phenylalanine **41**, 4-NO_2_-L-phenylalanine **42**, PLP, CuSO_4_ · 5H_2_O and kanamycin were purchased from Sigma-Aldrich (Germany). *Meta*-L-tyrosine **9**, *meta*-tyramine **10**, 3-F-L-tyrosine **11**, phenylacetaldehyde **47**, 2-bromophenylacetaldyhyde **50** and IPTG was purchased from Alfa Aesar (Thermo Fisher Scientific, USA). All chemicals were purchased in the highest purity available. Thin layer chromatography was performed using plates with a silica gel matrix on an aluminium support. Ultraviolet light (254 nm) and ninhydrin stain was used to visualise the plates.

### HPLC methods

Analytical methods were performed with a Dionex^TM^ UltiMate^TM^ 3000 HPLC System, with a Dionex^TM^ UltiMate^TM^ 3000 RS Pump, a Dionex^TM^ UltiMate^TM^ 3000 Autosampler, a Dionex^TM^ UltiMateTM 3000 Column Compartment and a UltiMate^TM^ 3000 RS Diode Array Detector (Thermofisher Scientific, US). Preparative methods were developed with a Agilent 1260 Infinity^TM^ HPLC System, with a 1260 Infinity^TM^ Preparative Pump, a 1260 Infinity^TM^ Preparative-scale Fraction Collector, a 1260 Infinity^TM^ Multiple Wavelength Detector, and a 1260 Infinity^TM^ Preparative Autosampler.

#### Analytical HPLC method 1 (achiral)

Achiral quantitative analyses adopted a reverse phase analysis method. Separation was achieved with an ACE 5 C18 column (150 × 4.6 mm) with a flow speed of 1 mL/min at 30 °C. The injection volume was 10 μL. Substrates and products were measured via UV absorbance at 280 nm. Eluent A (H_2_O with (v/v) 0.1% TFA) and eluent B (acetonitrile) were used as a mobile phase over 10 min. The gradient was as: 0.0 min (10% B)-1.0 min (10% B)-6.0 min (70 % B)-6.1 min (100% B)-6.5 min (100% B)-6.6 min (10% B)-10.0 min (10% B).

#### Analytical HPLC method 2 (chiral)

The chiral separation was achieved with an Supelco Astec Chirobiotic^TM^ T column (25 cm × 4.6 mm) or a Supelco Astec Chirobiotic^TM^ T2 column (25 cm × 4.6 mm), and a flow speed of 1 mL/min at 30 °C. The injection volume was 5 μL. Products were measured via UV absorbance at 230 nm. Methanol (0.2% AcOH, 0.1% TEA) was used as a mobile phase over 40 min or 80 min.

#### Analytical HPLC method 3 (chiral)

The chiral separation was achieved with a Supelco Astec Chirobiotic^TM^ T column (25 cm × 4.6 mm) and a flow speed of 0.2 mL/min at 30 °C. The injection volumes were 5 µL. Compounds were detected by UV absorbance at 230 nm. An isocratic mobile phase 20 mM NH_4_OAc (pH 4.0):MeOH (70:30) was used over 120 min.

#### Preparative HPLC method 4

The separation was achieved with a Vydac^TM^ 218TP1022 (C18, 10 µm, 2.2 cm ID × 25 cm L) preparative column or a Supelco^TM^ Discovery BIO wide pore (C18, 10 µm, 2.12 cm × 25 cm) preparative column and a flow speed of 8 mL/min at 25 °C. The injection volume was 900 μL. Products were identified via UV absorbances at 214 nm and 280 nm. Eluent A (H_2_O with 0.1% (v/v) TFA) and eluent B (acetonitrile with 0.1% (v/v) TFA) were used as a mobile phase over 28 min. The gradient was as: 0.0 min (5 % B)-3.0 min (5% B)-20.0 min (70% B)-22.0 min (70% B)-23.0 min (5% B)-28.0 min (5% B).

#### Preparative HPLC method 5

The separation was achieved with a VydacTM 218TP1022 (C18, 10 µm, 2.2 cm ID × 25 cm L) preparative column or a SupelcoTM Discovery BIO wide pore (C18, 10 µm, 2.12 cm × 25 cm) preparative column and a flow speed of 8 mL/min at 25 °C. The injection volume was 900 μL. Products were identified via UV absorbances at 214 and 280 nm. Eluent A (H_2_O with 0.1% (v/v) TFA) and eluent B (acetonitrile with 0.1% (v/v) TFA) were used as a mobile phase over 28 min. The gradient was as: 0.0 min (25% B)-3.0 min (25% B)-20.0 min (90% B)-22.0 min (90% B)-23.0 min (25% B)-28.0 min (25% B).

### Chemical analytics

^1^H and ^13^C NMR spectra were obtained using a Bruker Advance III 700 MHz spectrometer. Chemical shifts specified are relative to trimethylsilane (set at 0 ppm) and referenced to the residual, protonated NMR solvent. Coupling constants in ^1^H-NMR spectra (*J*) are given in Hertz (Hz) and described as singlet (s), doublet (d), doublet of doublets (dd), triplet (t), quartet (q), multiplet (m). Mass spectrometry data of compounds were measured on an Agilent 1100 Series System with a Finnigan LTQ mass spectrometer. An ACE 5 C18 reverse phase column (50 mm × 2.1 mm, 5 μm) was adopted with a mobile phase of eluent A (H_2_O with 0.1% (v/v) formic acid) and eluent B (acetonitrile) over 5 min with a flow rate of 0.6 mL/min. The sample injection volume was 10 μL. Chemical compounds were measured in a positive ion mode, and the operating conditions of the ESI interface were set to a capillary temperature 300 °C, capillary voltage 9 V, spray voltage 4 kV, sheath gas 40, auxiliary gas 10, sweep gas 0 arbitrary units. The gradient of eluents was as: 0.0 min (5% B)-4.0 min (90% B)-4.5 min (5% B)-5.0 min (5% B).

### Recombinant protein expression in *E. coli* BL21 (DE3)

Selected enzyme glycerol stocks (*E. coli* BL21 (DE3)) were plated out on agar plates supplemented with 50 µg/mL kanamycin. A single colony was then picked to inoculate into 10 mL of LB media supplemented with 50 µg/mL kanamycin and grown at 37 °C and 250 rpm overnight (8–16 h). 1 mL of the overnight cultures were inoculated into a 500 mL baffled shaking flask containing 100 mL of TB media supplemented with 50 μg/mL kanamycin at 37 °C, 250 rpm until an OD_600_ = 0.6. Enzyme expression was induced by the addition of 1 mM IPTG (and 100 μM CuSO_4_ · 5H_2_O for *Cn*TYR) to the culture. Cultures were incubated overnight at 25 °C prior for harvesting, whilst shaking at 250 rpm. Cells were harvested by centrifugation (12,000 × *g*, 15 min) and the cell pellets was stored at −20 °C

### Cell lysate preparation

Cell pellets (50 mL culture) were resuspended in 5 mL of 50 mM HEPES buffer (pH 5.5/pH7.5) and lysed by 10 cycles of sonication on ice (10 s on plus 10 s off, 12 watts output) equipped with a Process Timer. Cell lysates were then centrifuged at 4 °C (12,000 × *g*, 15 min). The supernatant was collected and buffer exchanged using SephadexTM G-25 in PD-10 column (GE Healthcare Life Sciences, Germany) with HEPES buffer (50 mM, pH 5.5/pH7.5) to remove the KPi in cell components. The concentration of supernatant protein was measured following the standard Bradford procedure. The samples were duplicated and the average OD_595_ were used for cell lysate concentration calculations.

### Determination of target protein concentrations in cell lysates

The recombinant proteins were expressed and analysed by SDS-PAGE. Then the SDS gel was analysed with a AlphaImagerTM gel documentation system (ProteinSimple, US), and the recombinant protein concentration present in the cell lysate was determined by AlphaView^TM^ FluorChem Q^TM^ software (ProteinSimple, US).

### Mutagenesis methods

#### Site-direct mutagenesis of CnTYR

Protein and DNA Alignment of the *Cn*TYR used in this study and the reported TYR was performed with the online service ‘Clustal Omega’ from EMBL-EBI (Cambridgeshire, UK). Alignment results were shown using the online service ‘Sequence Manipulation Suite’ from the University of Alberta (Alberta, CA). Genes for the designed mutants were synthesised from Eurofins Scientific (Belgium).

#### Random mutagenesis of CnTYR

Random mutagenesis was performed with *E. coli* XL 1-Red competent cells purchased from Agilent Technologies^TM^ (Santa Clara, US). Mutation procedures followed the instruction manual. After mutation, plasmids were extracted using the Qiagen Mini Prep KitTM (Qiagen, Germany) and further transformed with *E. coli* BL21 (DE3) competent cells. After cultivation at 37 °C for 1 h, cells were plated on the LB agar plate with 50 μg/mL kanamycin and 500 μM IPTG. The plates were cultivated at 37 °C overnight. Colonies shown black colour were selected and inoculated in 10 mL of TB broth with 50 μg/mL kanamycin at 37 °C overnight. As a single colony may contain various plasmids, to obtain the stable mutants, again, plasmids were extracted and transformed with *E. coli* BL21 (DE3) competent cells, which was then plated on the LB agar plates with 50 μg/mL kanamycin and 500 μM IPTG and grown overnight. The above steps were repeated twice. Afterwards, colonies shown black were chosen and further cultivated in TB broth. Mutated proteins were expressed, and cell lysates were used for enzyme screening with 3-F-L-tyrosine **11** and 3-Cl-L-tyrosine **12** based on the colorimetric reaction. The DNA of the selected mutants were sequenced by ‘DNA Sequencing Service’ from Eurofins Scientific (Belgium).

### Reporting summary

Further information on research design is available in the Nature Research Reporting Summary linked to this article.

## Supplementary information

Supplementary Information

Reporting Summary

## Data Availability

Data to support this work is available from the corresponding authors upon request. Calibration curves - all calibration curves used for determination of conversions to give products are given in the supplementary information. Complete chemical syntheses and analyses - complete synthetic methods, characterisation of THIQ products and corresponding chiral HPLC data are given in the supplementary information. AutoDock Vina - experiment details of the docking study using AutoDock Vina are given in the supplementary information. 1. The protein sequence for enzymes used in this study are available in the Genbank database under accession code list below: 1). Tyrosinase from *Candidatus Nitrosopumilus salaria* BD31(*Cn*TYR, accession code: EIJ65432.1) 2). Tyrosine decarboxylase from *Enterococcus faecalis* (*Ef*TyrDC, accession code: AFO43338.1) 3). Transaminase from *Chromobacterium violaceum* (*Cv*TAm, accession code: AAQ59973.1) 4). (*S*)-Norcoclaurine synthase from *Thalictrum flavum* (*Δ29Tf*NCS, accession code: AAR22502.1) 5). Catechol-*O*-methyltransferase from rat liver (*Rn*COMT, accession code: AAA40881.1) 6). Catechol-*O*-methyltransferase from *Myxococcus xanthus* (*Mx*SafC, accession code: AAC44130.1) 7). Norcoclaurine 6-*O*-methyltransferase from *Coptis japonica* (*Cj*6OMT, accession code: BAB08004.1) 8). *S*-Adenosylmethionine synthetase from *E. coli* (*Ec*MAT, accession code: AAA24164.1) 9). Methylthioadenosine/SAH nucleosidase from *E. coli* (*Ec*MTAN, accession code: AAB08589.1) 2. The protein sequence for CnTYR variants and TfNCS variants are available in the supplementary information. 3. The crystal structure data for enzymes in this study are provided in the RCSB Protein Data Bank (PDB) under accession code list below: 1). Crystal structure of tyrosinase from *Bacillus megaterium* (*Bm*TYR, accession code: 3NPY) 2). Crystal structure of (*S*)-norcoclaurine synthase from *Thalictrum flavum* (*Δ29Tf*NCS, accession code:5N8Q)
