# Supplementary material for: Enzymatic synthesis of benzylisoquinoline alkaloids using a parallel cascade strategy and tyrosinase variants
Source: Nat Commun. 2022 Sep 16;13:5436. doi: 10.1038/s41467-022-33122-1 (PMC9481557; doi:10.1038/s41467-022-33122-1)
Supplement: Supplementary file 1 — Supplementary Information [file 41467_2022_33122_MOESM1_ESM.pdf]

Supplementary Information

Enzymatic synthesis of benzylisoquinoline alkaloids using a parallel cascade strategy and tyrosinase variants

Yu Wang[1], Fabiana Subrizi[1], Eve M. Carter[1], Tom D. Sheppard[1], John M. Ward[2] and Helen C. Hailes[1]*

[1]Department of Chemistry, Christopher Ingold Building, 20 Gordon Street, University College London, WC1H 0AJ, U.K.

[2]Department of Biochemical Engineering, Bernard Katz Building, University College London, WC1E 6BT, U.K.

*Corresponding author: Helen C. Hailes (h.c.hailes@ucl.ac.uk)

Table of contents

Supplementary Notes 3

1 Structures of starting materials used in the study 3

2 Protein sequences used in the study 3

Supplementary Methods 6

1. Enzyme reaction method for each enzyme cascade 6

2 Protein expression of enzymes used in the study 31

3 Kinetic studies for *Cn*TYR and *Cn*TYR-N201S 32

4 Achiral analytical HPLC results for *Cn*TYR and *Ef*TyrDC reaction products 33

5 Achiral analytical HPLC results for Pictet-Spengler reaction products 34

6 Calibration curves for analytical HPLC results of products 40

7 Chiral analytical HPLC results for cascade reaction products 42

8 Accurate mass spectra for the novel products 51

9 NMR spectroscopic data for cascade reaction products 60

10 Global docking via AutoDock Vina 80

11 Protein sequence alignment for *Cn*TYR, *Rs*TYR and *Bm*TYR 84

12 Docking studies of *Cn*TYR variants with Cl-tyrosine 12 85

13 Colorimetric selection for *Cn*TYR variants with different substrates 86

14 Docking study for *Tf*NCS variants with halogenated BIAs 86

Supplementary References 91

Supplementary Notes

1 Structures of starting materials used in the study

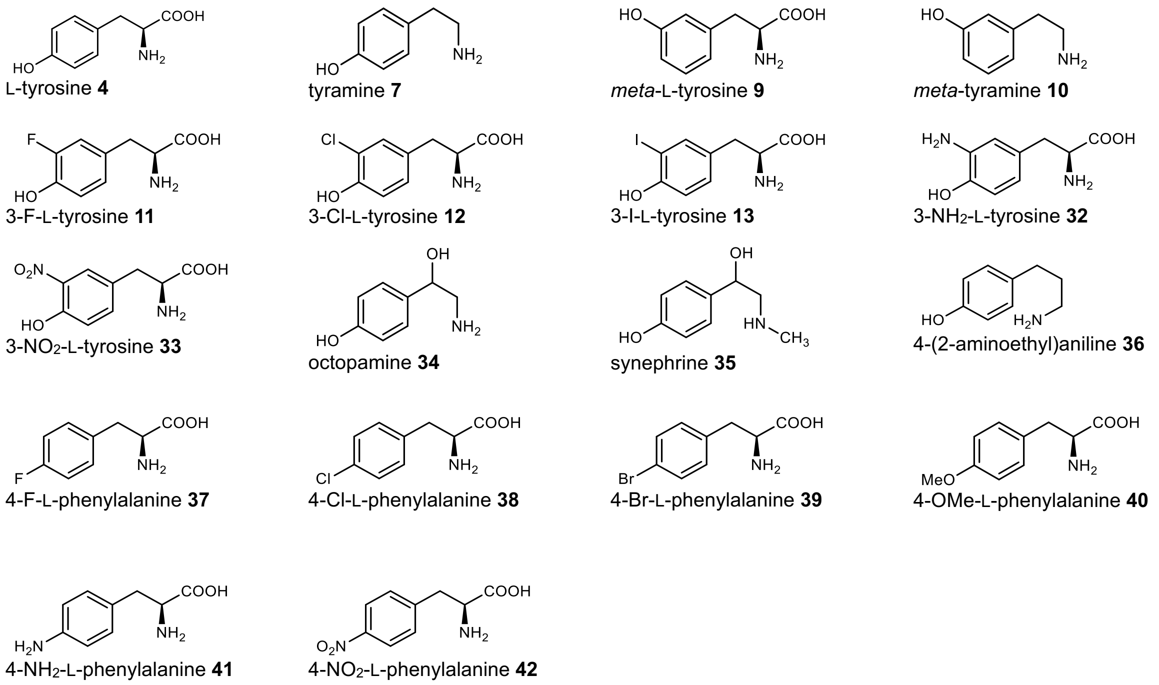

Supplementary Fig. 1 Structures of starting materials used in the study.

2 Protein sequences used in the study

>Tyrosinase from *Candidatus Nitrosopumilus salaria* BD31(*Cn*TYR, GenBank: EIJ65432.1)

MVRKNASSLNPIERENFCKAVLTLKNTKIPGHALNRYDEFVAIHFGVTSRERANLPIGDGAHGNSGFLPWHRE
FLCRFEHALKSVDPTVSLPYWDWSSGDTSDTIDIFNDDFMGPAGTVNSGYFSGTGNSFNSNRPWIVHPSLDQT
SPGQPPLGSTLIRNSNLLSASTLNYLMDLGEMARDSLNESTYNAFRSTLEHPPHNHVHGVTVQGHMGWMTSPN
DPIFFLHHANVDRLWAEWQRTHPGSSNYTPNATEPYGVHLNDPMWPWQGADTTVTTRTHTDSNASLNTLLPSF
STADLVTPNDVLDHIQRCGPYDTDPISKPKEFEKIPKEIIKEIIKDKEKEFGDKNPKEIIKEIIKDKEKEFGD
KNPKEIIKEIIKDKEKEFGDKNPKEIKEIIKDKEKEFGDKNPKEIKEIIKDKEKEFGDKNPKEIIETGDIKIE
NNKDVVEILSTPSTTVSSPKHPKEQSKETLEITNTLFDPLSKINHRLDMLENEIKGTAFIKSTERPNITKRAI
SKNTSTKKTTRKKTKNTKNTMPKKSNTSKRKRISHHHHHH

>*Cn*TYR-N201S from *Candidatus Nitrosopumilus salaria* (mutant based on *Cn*TYR)

MVRKNASSLNPIERENFCKAVLTLKNTKIPGHALNRYDEFVAIHFGVTSRERANLPIGDGAHGNSGFLPWHRE
FLCRFEHALKSVDPTVSLPYWDWSSGDTSDTIDIFNDDFMGPAGTVNSGYFSGTGNSFNSNRPWIVHPSLDQT
SPGQPPLGSTLIRNSNLLSASTLNYLMDLGEMARDSLNESTYNAFRSTLEHPPHSHVHGVTVQGHMGWMTSPN
DPIFFLHHANVDRLWAEWQRTHPGSSNYTPNATEPYGVHLNDPMWPWQGADTTVTTRTHTDSNASLNTLLPSF
STADLVTPNDVLDHIQRCGPYDTDPISKPKEFEKIPKEIIKEIIKDKEKEFGDKNPKEIIKEIIKDKEKEFGD
KNPKEIIKEIIKDKEKEFGDKNPKEIKEIIKDKEKEFGDKNPKEIKEIIKDKEKEFGDKNPKEIIETGDIKIE

NNKDVVEILSTPSTTVSSPKHPKEQSKETLEITNTLFDPLSKINHRLDMLENEIKGTAFIKSTERPNITKRAI
SKNTSTKKTTRKKTKNTKNTMPKKSNTSKRKRISHHHHHH

>*Cn*TYR-N132A/L289V from *Candidatus Nitrosopumilus salaria* (mutant based on *Cn*TYR)

MVRKNASSLNPIERENFCKAVLTLKNTKIPGHALNRYDEFVAIHFGVTSRERANLPIGDGAHGNSGFLPWHRE
FLCRFEHALKSVDPTVSLPYWDWSSGDTSDTIDIFNDDFMGPAGTVNSGYFSGTGNSFASNRPWIVHPSLDQT
SPGQPPLGSTLIRNSNLLSASTLNYLMDLGEMARDSLNESTYNAFRSTLEHPPHNHVHGVTVQGHMGWMTSPN
DPIFFLHHANVDRLWAEWQRTHPGSSNYTPNATEPYGVHLNDPMWPWQGADTTVTTRTHTDSNASLNTLVPSF
STADLVTPNDVLDHIQRCGPYDTDPISKPKEFEKIPKEIIKEIIKDKEKEFGDKNPKEIIKEIIKDKEKEFGD
KNPKEIIKEIIKDKEKEFGDKNPKEIKEIIKDKEKEFGDKNPKEIKEIIKDKEKEFGDKNPKEIIETGDIKIE
NNKDVVEILSTPSTTVSSPKHPKEQSKETLEITNTLFDPLSKINHRLDMLENEIKGTAFIKSTERPNITKRAI
SKNTSTKKTTRKKTKNTKNTMPKKSNTSKRKRISHHHHHH

>Tyrosine decarboxylase from *Enterococcus faecalis* (*Ef*TyrDC, GenBank: AFO43338.1)

MKNEKLAKGEMNLNALFIGDKAENGQLYKDLLIDLVDEHLGWRQNYMPQDMPVISSQERTSESYEKTVNHMKD
VLNEISSRMRTHSVPWHTAGRYWGHMNSETLMPSLLAYNFAMLWNGNNVAYESSPATSQMEEEVGHEFAHLMS
YKNGWGHIVADGSLANLEGLWYARNIKSLPFAMKEVKPELVAGKSDWELLNMPTKEIMDLLESAEDEIDEIKA
HSARSGKHLQAIGKWLVPQTKHYSWLKAADIIGIGLDQVIPVPVDHNYRMDINELEKIVRGLAEEQIPVLGVV
GVVGSTEEGAVDSIDKIIALRDELMKDGIYYYVHVDAAYGGYGRAIFLDEDNNFIPYEDLQDVHEEYGVFKEK
KEHISREVYDAYKAIELAESVTIDPHKMGYIPYSAGGIVIQDIRMRDVISYFATYVFEKGADIPALLGAYILE
GSKAGATAASVWAAHHVLPLNVAGYGKLIGASIEGSHHFYNFLNDLTFKVGDKEIEVHTLTHPDFNMVDYVFK
EKGNDDLVAMNKLNHDVYDYASYVKGNIYNNEFITSHTDFAIPDYGNSPLKFVNSLGFSDEEWNRAGKVTVLR
AAVMTPYMNDKEEFDVYAPKIQAALQEKLEQIYDVKHHHHHH

>Transaminase from *Chromobacterium violaceum* (*Cv*TAm, GenBank: AAQ59973.1)

MAKVYNFSAGPAVLPHQVLAEAQSELLDWHGSGMSVMEMSHRGKEFMEIIHDAEQDLRQLMGIPAGYKVLFLQ
GGASLQFAMAPLNLLGDKDSIDIVNTGHWSKLAIKEAKRYAKVNVVASSEDRNFCYVPEEAAWQRDPNAAYLH
YTSNETIGGLQFPYIPAEQHGVPLVCDMSSDFLSREVDVSRFGMIYAGAQKNIGPSGLTVLLIREDLLGKARA
DIPTMLNYQVHADADSMYNTPGTYPIYIAGLVFKWLKEQGGVKGIATRNEEKAGLLYHVIDSSGGFYSTHIEQ
PFRSKMNVVFKLRDEALDEIFLLEARKNGLAQLKGHRAVGGMRASIYNAMPIEGVKSLVNFMQDFARQYGHHH
HHH

>*Δ29*TfNCS (GenBank: AAR22502.1)

MHHHHHHSSGVDLGTENLYFQSMLHHQGIINQVSTVTKVIHHELEVAASADDIWTVYSWPGLAKHLPDLLPGA
FEKLEIIGDGGVGTILDMTFVPGEFPHEYKEKFILVDNEHRLKKVQMIEGGYLDLGVTYYMDTIHVVPTGKDS
CVIKSSTEYHVKPEFVKIVEPLITTGPLAAMADAISKLVLEHKSKSNSDEIEAAIITVLE

>*Δ29*TfNCS-A79I (mutant based on *Δ29*TfNCS)

MHHHHHHSSGVDLGTENLYFQSMLHHQGIINQVSTVTKVIHHELEVAASADDIWTVYSWPGLAKHLPDLLPGA
FEKLEAIGDGGVGTILDMTFVPGEFPHEYKEKFILVDNEHRLKKVQMIEGGYLDLGVTYYMDTIHVVPTGKDS

CVIKSSTEYHVKPEFVKIVEPLITTGPLAAMADAISKLVLEHKSKSNSDEIEAAIITVLE

>Catechol-*O*-methyltransferase from rat liver(*Rn*COMT, GenBank: AAA40881.1)

MGSSHHHHHHSSGLVPRGSHMGDTKEQRILRYVQQNAKPGDPQSVLEAIDTYCTQKEWAMNVGDAKGQIMDAV
IREYSPSLVLELGAYCGYSAVRMARLLQPGARLLTMEMNPDYAAITQQMLNFAGLQDKVTILNGASQDLIPQL
KKKYDVDTLDMVFLDHWKDRYLPDTLLLEKCGLLRKGTVLLADNVIVPGTPDFLAYVRGSSSFECTHYSSYLE
YMKVVDGLEKAIYQGPSSPDKS

>Catechol-*O*-methyltransferase from *Myxococcus xanthus* (*Mx*SafC, GenBank: AAC44130.1)

MIHHVELTQSVLQYIRDSSVRDNDILRDLREETSKLPLRTMQIPPEQGQLLSLLVRLIGARKTLEVGVFTGYS
TLCAALALPADGRVIACDLSEEWVSIARRYWQRAGVADRIEVRLGDAHHSLEALVGSEHRGTFDLAFIDADKE
SYDFYYEHALRLVRPGGLIILDNTLWSGKVADPSVVGDPETDSLRRINAKLLTDERVDLSMLPIADGLTLARK
RKLAAALEHHHHHH

>Norcoclaurine 6-*O*-methyltransferase from *Coptis japonica* (*Cj*6OMT, GenBank: BAB08004.1)

MEVKKDNLSSQAKLWNFIYGFAESLVLKCAVQLDLANIIHNSGTSMTLSELSSRLPSQPVNEDALYRVMRYLV
HMKLFTKASIDGELRYGLAPPAKYLVKGWDKCMVGSILAITDKDFMAPWHYLKDGLSGESGTAFEKALGTNIW
GYMAEHPEKNQLFNEAMANDSRLIMSALVKECGNIFNGITTLVDVGGGTGTAVRNIANAFPHIKCTVYDLPHV
IADSPGYSEVHCVAGDMFKFIPKADAIMMKCILHDWDDKECIEILKRCKEAVPVKGGKVIIVDIVLNVQSEHP
YTKMRLTLDLDMMLNTGGKERTEEEWKKLIHDAGYKGHKITQITAVQSVIEAYPYHHHHHH

>*S*-adenosylmethionine synthetase from *E. coli* (*Ec*MAT, GenBank: AAA24164.1)

MGSSHHHHHHSSGLVPRGSHMAKHLFTSESVSEGHPDKIADQISDAVLDAILEQDPKARVACETYVKTGMVLV
GGEITTSAWVDIEEITRNTVREIGYVHSDMGFDANSCAVLSAIGKQSPDINQGVDRADPLEQGAGDQGLMFGY
ATNETDVLMPAPITYAHRLVQRQAEVRKNGTLPWLRPDAKSQVTFQYDDGKIVGIDAVVLSTQHSEEIDQKSL
QEAVMEEIIKPILPAEWLTSATKFFINPTGRFVIGGPMGDCGLTGRKIIVDTYGGMARHGGGAFSGKDPSKVD
RSAAYAARYVAKNIVAAGLADRCEIQVSYAIGVAEPTSIMVETFGTEKVPSEQLTLLVREFFDLRPYGLIQML
DLLHPIYKETAAYGHFGREHFPWEKTDKAQLLRDAAGLK

> Methylthioadenosine/SAH nucleosidase from *E. coli* (*Ec*MTAN, GenBank: AAB08589.1)

MGSSHHHHHHSSGLVPRGSHMKIGIIGAMEEEVTLLRDKIENRQTISLGGCEIYTGQLNGTEVALLKSGIGKV
AAALGATLLLEHCKPDVIINTGSAGGLAPTLKVGDIVVSDEARYHDADVTAFGYEYGQLPGCPAGFKADDKLI
AAAEACIAELNLNAVRGLIVSGDAFINGSVGLAKIRHNFPQAIAVEMEATAIAHVCHNFNVPFVVVRAISDVA
DQQSHLSFDEFLAVAAKQSSLMVESLVQKLAHG

Supplementary Methods

1. Enzyme reaction method for each enzyme cascade

*(S)-1-(3-Hydroxybenzyl)-1,2,3,4-tetrahydroisoquinoline-6,7-diol (S)-**23***

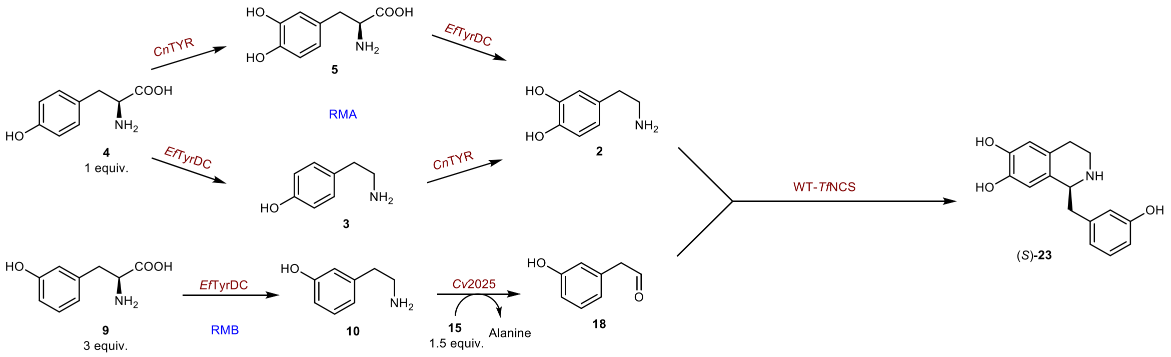

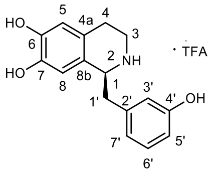

To synthesize the amine moiety, reaction mixture A (RMA, 50 mL, pH 5.5) consisted of 50 mM HEPES, 2.5 mM L-tyrosine **4**, 10 mM sodium ascorbate **8**, 40 μM $CuSO_4 \cdot 5H_2O$ and 1.25 mM PLP. To initiate the hydroxylation and decarboxylation steps, 10% (v/v) of *Cn*TYR lysates and 10% (v/v) of *Ef*TyrDC lysates were added to RMA which was incubated at 25 °C, 250 rpm for 6 h. For the aldehyde moiety, reaction mixture B (RMB, 50 mL, pH 7.5) consisted of 10% (v/v) of MeCN, 50 mM HEPES, 7.5 mM *meta*-L-tyrosine **9**, 7.5 mM **8**, 1.25 mM PLP and 7.5 mM sodium pyruvate **15**. To initiate the decarboxylation and transamination steps, 10% (v/v) of *Ef*TyrDC lysates and 5% (v/v) of *Cv*TAm lysates were added to RMB and the solution was incubated at 37 °C, 250 rpm for 6 h. Then RMA and RMB were mixed. The Pictet-Spengler condensation was performed with 10% (v/v) of *Tf*NCS lysates at 37 °C, 250 rpm for 8 h. In control reactions, all the enzymes were replaced by empty-vector cell lysates. The product was purified using preparative HPLC (method 4, Vydac™ 218TP1022 (C18, 10 μm, 2.2 cm x 25 cm) preparative column, retention time: 15.5 min, run time: 28 mins, flow rate: 8 mL/min). Fractions containing the desired product were freeze-dried to give (*S*)-**23** as an off-white powder (TFA salt, yield by HPLC (calibration curve) 82% (method 1, ACE 5 C18 column (150 × 4.6 mm), retention time: 4.9 min, run time: 10 mins, flow rate: 1 mL/min); final isolated yield 47% (21.7 mg); *ee.* 90% (chiral HPLC, method 2, Supelco Astec Chirobiotic™ T2 column (25 cm × 4.6 mm), retention time: 21.6

min, run time: 80 mins, flow rate: 1 mL/min). ^{1}H NMR (600 MHz; CD_3OD) δ = 7.22-7.19 (1H, m, 6'-H), 6.79-6.75 (3H, m, 3'-H, 5'-H and 7'-H), 6.63 (2H, 2 x s, 5-H and 8-H), 4.62 (1H, dd, *J* = 8.4 Hz, 5.7 Hz, 1-H), 3.41-3.37 (1H, m, 3-HH), 3.39 (1H, dd, *J* = 15.0 Hz, 5.7 Hz, 1'-HH), 3.27-3.23 (1H, m, 3-HH), 3.00-2.97 (2H, m, 1'-HH and 4-HH), 2.93-2.89 (1H, m, 4-HH); ^{13}C NMR (151 MHz; CD_3OD) δ = 159.3, 146.9, 145.8, 138.0, 131.3, 123.69, 123.66, 121.5, 117.4, 116.2, 115.7, 114.1, 57.7, 41.2, 41.0, 25.7; *m/z* [ES+] 272 ([M+H]+, 100%); *m/z* [HRMS ES+] found $[M+H]^+$ 272.1289. $[C_{16}H_{16}FNO_2+H]^+$ requires 272.1287; $[\alpha]_D^{28}$ -18.0 (c 0.36, MeOH).

(S)-1-Phenethyl-1,2,3,4-tetrahydroisoquinoline-6,7-diol (S)-24[1]

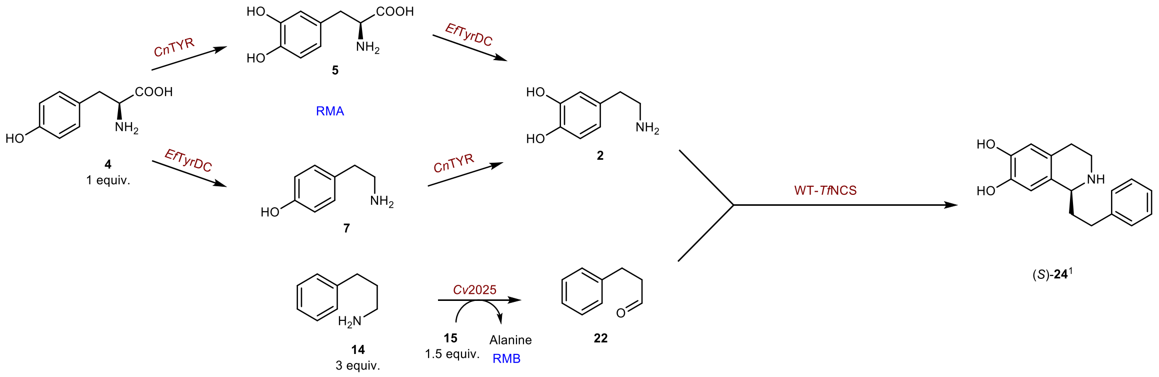

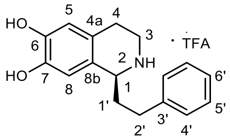

To synthesize the amine moiety, reaction mixture A (RMA, 50 mL, pH 5.5) consisted of 50 mM HEPES, 2.5 mM L-tyrosine **4**, 10 mM sodium ascorbate **8**, 40 µM $CuSO_4 \cdot 5H_2O$ and 1.25 mM PLP. To initiate the hydroxylation and decarboxylation steps, 10% (v/v) of *Cn*TYR lysates and 10% (v/v) of *Ef*TyrDC lysates were added to RMA which was incubated at 25 °C, 250 rpm for 6 h. For the aldehyde moiety, reaction mixture B (RMB, 50 mL, pH 7.5) consisted of 10% (v/v) MeCN, 50 mM HEPES, 7.5 mM phenylpropylamine **14**, 7.5 mM sodium ascorbate **8**, 7.5 mM sodium pyruvate **15** and 1.25 mM PLP. To initiate the transamination step, 5% (v/v) of *Cv*TAm lysates was added to RMB and was incubated at 37 °C, 250 rpm for 6 h. Then RMA and RMB were combined. The Pictet-Spengler condensation was performed with 10% (v/v) of *Tf*NCS lysates at 37 °C, 250 rpm for 16 h.

In control reactions, all the enzymes were replaced by empty-vector cell lysates. The product was purified using preparative HPLC (method 4, Supelco™ Discovery BIO wide pore (C18, 10 μm, 2.12 cm x 25 cm) preparative column, retention time: 19.7 min, run time: 28 mins, flow rate: 8 mL/min). Fractions containing the desired product were freeze-dried to give (*S*)-**24** as a white powder (TFA salt, yield by HPLC (calibration curve) 21% (method 1, ACE 5 C18 column (150 × 4.6 mm), retention time: 5.9 min, run time: 10 mins, flow rate: 1 mL/min); final isolated yield 14% (6.4 mg); *ee.* 90% (chiral HPLC, method 2, Supelco Astec Chirobiotic™ T2 column (25 cm × 4.6 mm), retention time: 22.7 min and 33.2 min, run time: 80 mins, flow rate: 1 mL/min). ^{1}H NMR (700 MHz; CD_3OD) δ = 7.31-7.19 (5H, m, Ph), 6.66 (1H, s, 8-H), 6.62 (1H, s, 5-H), 4.40 (1H, dd, *J* = 7.7 Hz, 4.9 Hz, 1-H), 3.57-3.54 (1H, m, 3-H$\underline{H}$), 3.37-3.34 (1H, m, 3-$\underline{H}$H), 3.04-3.00 (1H, m, 4-H$\underline{H}$), 2.94-2.90 (1H, m, 4-$\underline{H}$H), 2.84-2.77 (2H, m, 1'-H$\underline{H}$ and 1'-$\underline{H}$H), 2.32-2.26 (1H, m, 2'-H$\underline{H}$), 2.24-2.19 (1H, m, 2'-$\underline{H}$H); ^{13}C NMR (175 MHz; CD_3OD) δ = 146.7, 145.8, 141.7, 129.6, 129.3, 127.4, 123.8, 123.7, 116.2, 113.8, 56.1, 40.8, 37.1, 32.4, 25.6; *m/z* [ES+] 270 ($[M+H]^+$, 100%); *m/z* [HRMS ES+] found $[M+H]^+$ 270.1495. $[C_{17}H_{19}NO_2+H]^+$ requires 270.1494; $[\alpha]_D^{28}$ 0.8 (c 0.24, MeOH).

*(S)-1-(3-Fluoro-4-hydroxybenzyl)-1,2,3,4-tetrahydroisoquinoline-6,7-diol (S)-**25***

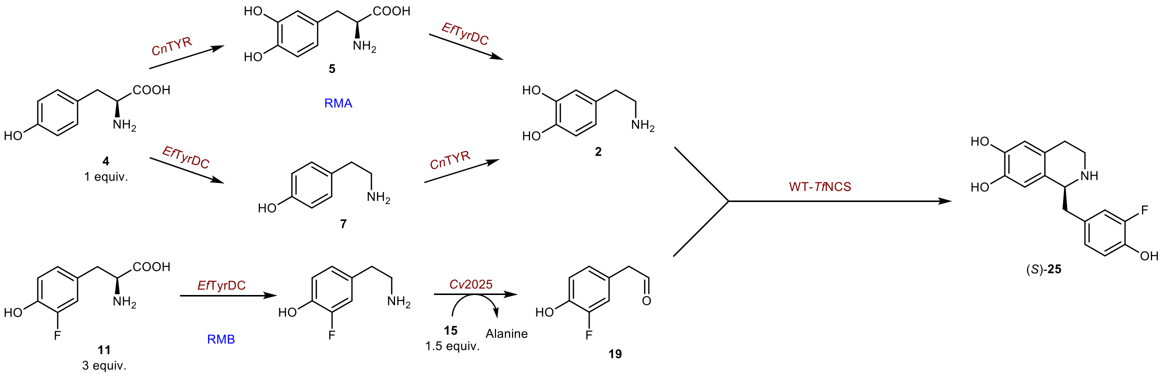

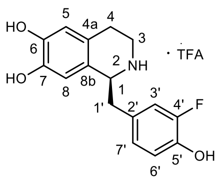

To synthesize the amine moiety, reaction mixture A (RMA, 50 mL, pH 5.5) consisted of 50 mM HEPES, 2.5 mM L-tyrosine **4**, 10 mM sodium ascorbate **8**, 40 μM $CuSO_4{\cdot}5H_2O$ and 1.25 mM PLP. To initiate the hydroxylation and decarboxylation steps, 10% (v/v) of *Cn*TYR

lysates and 10% (v/v) of *Ef*TyrDC lysates were added to RMA which was incubated at 25 °C, 250 rpm for 6 h. For the aldehyde moiety, reaction mixture B (RMB, 50 mL, pH 7.5) consisted of 10% (v/v) of MeCN, 50 mM HEPES, 7.5 mM 3-F-L-tyrosine **11**, 7.5 mM sodium ascorbate **8**, 1.25 mM PLP and 7.5 mM sodium pyruvate **15**. To initiate the decarboxylation and transamination steps, 10% (v/v) of *Ef*TyrDC lysates and 5% (v/v) of *Cv*TAm lysates were added to RMB and the solution was incubated at 37 °C, 250 rpm for 6 h. Then RMA and RMB were mixed. The Pictet-Spengler condensation was performed with 10% (v/v) of *Tf*NCS lysates at 37 °C, 250 rpm for 8 h. In control reactions, all the enzymes were replaced by empty-vector cell lysates. The product was purified using preparative HPLC (method 4, Vydac™ 218TP1022 (C18, 10 μm, 2.2 cm x 25 cm) preparative column, retention time: 16.0 min, run time: 28 mins, flow rate: 8 mL/min). Fractions containing the desired product were freeze-dried to give (*S*)-**25** as a yellow powder (TFA salt, yield by HPLC (calibration curve) 84% (method 1, ACE 5 C18 column (150 × 4.6 mm), retention time: 4.9 min, run time: 10 mins, flow rate: 1 mL/min); final isolated yield 42% (20.3 mg); *ee.* 96% (chiral HPLC, method 2, Supelco Astec Chirobiotic™ T2 column (25 cm × 4.6 mm), retention time: 19.4 min, run time: 80 mins, flow rate: 1 mL/min). ^{1}H NMR (600 MHz; CD_3OD) δ = 7.06-7.03 (1H, m, 6'-H), 6.93-6.91 (2H, m, 3'-H and 7'-H), 6.62 (1H, s, 5-H), 6.59 (1H, s, 8-H), 4.59 (1H, dd, *J* = 8.4 Hz, 5.7 Hz, 1-H), 3.48-3.44 (1H, m, 3-H<u>H</u>), 3.36 (1H, dd, *J* = 14.4 Hz, 5.7 Hz, 1'-H<u>H</u>), 3.28-3.23 (1H, m, 3-<u>H</u>H), 3.00-2.96 (2H, m, 1'-<u>H</u>H and 4-H<u>H</u>) 2.93-2.88 (1H, m, 4-<u>H</u>H); ^{13}C NMR (151 MHz; CD_3OD) δ = 153.0 (d, $^{1}J_{CF}$ = 241.6 Hz), 152.2 (d, $^{2}J_{CF}$ = 40.8 Hz), 146.9, 145.8, 128.1 (d, $^{4}J_{CF}$ = 6.0 Hz), 126.8, 123.7, 123.5, 119.3, 118.0 (d, $^{2}J_{CF}$ = 18.1 Hz), 116.2 (d, $^{3}J_{CF}$ = 6.0 Hz), 114.2 (d, $^{3}J_{CF}$ = 6.0 Hz), 57.7, 40.9, 40.2, 20.4; *m/z* [ES+] 290 ($[M+H]^+$, 100%); *m/z* [HRMS ES+] found $[M+H]^+$ 290.1192. $[C_{16}H_{16}FNO_3+H]^+$ requires 290.1192; $[\alpha]_D^{28}$ -16.2 (c 0.7, MeOH).

*(S)-1-(3-Chloro-4-hydroxybenzyl)-1,2,3,4-tetrahydroisoquinoline-6,7-diol (S)-**26***[2]

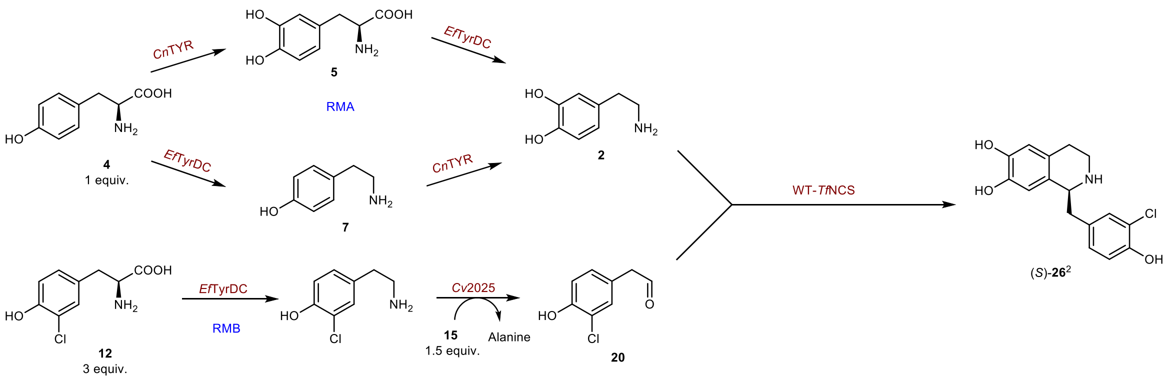

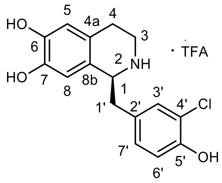

To synthesize the amine moiety, reaction mixture A (RMA, 50 mL, pH 5.5) consisted of 50 mM HEPES, 2.5 mM L-tyrosine **4**, 10 mM sodium ascorbate **8**, 40 μM $CuSO_4{\cdot}5H_2O$ and 1.25 mM PLP. To initiate the hydroxylation and decarboxylation steps, 10% (v/v) of *Cn*TYR lysates and 10% (v/v) of *Ef*TyrDC lysates were added to RMA which was incubated at 25 °C, 250 rpm for 6 h. For the aldehyde moiety, reaction mixture B (RMB, 50 mL, pH 7.5) consisted of 10% (v/v) of MeCN, 50 mM HEPES, 7.5 mM 3-Cl-L-tyrosine **12**, 7.5 mM sodium ascorbate **8**, 1.25 mM PLP and 7.5 mM sodium pyruvate **15**. To initiate the decarboxylation and transamination steps, 10% (v/v) of *Ef*TyrDC lysates and 5% (v/v) of *Cv*TAm lysates were added to RMB and the solution was incubated at 37 °C, 250 rpm for 6 h. Then RMA and RMB were mixed. The Pictet-Spengler condensation was performed with 10% (v/v) of *Tf*NCS lysates at 37 °C, 250 rpm for 8 h. In control reactions, all the enzymes were replaced by empty-vector cell lysates. The product was purified using preparative HPLC (method 5, Vydac^{TM} 218TP1022 (C18, 10 μm, 2.2 cm x 25 cm) preparative column, retention time: 10.0 min, run time: 28 mins, flow rate: 8 mL/min). Fractions containing the desired product were freeze-dried to give (*S*)-**26** as a yellow powder (TFA salt, yield by HPLC (calibration curve) 86% (method 1, ACE 5 C18 column (150 × 4.6 mm), retention time: 5.2 min, run time: 10 mins, flow rate: 1 mL/min); final isolated yield 40% (20.1 mg); *ee.* 94% (chiral HPLC, method 2, Supelco Astec Chirobiotic^{TM} T2 column (25 cm × 4.6 mm), retention time: 20.6 min, run time: 80 mins, flow rate: 1 mL/min). ^{1}H NMR (600 MHz; CD_3OD) δ = 7.30 (1H, d, *J* = 1.8

Hz, 3'-H), 7.07 (1H, dd, J = 7.8 Hz, 1.8 Hz, 7'-H), 6.93 (1H, d, J = 7.8 Hz, 6'-H), 6.63 (1H, s, 5-H), 6.60 (1H, s, 8-H), 4.59 (1H, dd, J = 8.4 Hz, 5.7 Hz, 1-H), 3.48-3.44 (1H, m, 3-HH), 3.36 (1H, dd, J = 15.0 Hz, 5.7 Hz, 1'-HH), 3.26-3.23 (1H, m, 3-HH), 3.02-2.88 (3H, m, 1'-HH, 4-HH and 4-HH); ^{13}C NMR (151 MHz; CD_3OD) δ = 154.0, 146.9, 145.8, 132.0, 130.1, 128.5, 123.7, 123.5, 122.2, 118.2, 116.2, 114.2, 57.7, 41.0, 40.9, 25.7; *m/z* [ES+] 306 ([M+H]+, 100%); *m/z* [HRMS ES+] found $[M+H]^+$ 306.0895. $[C_{16}H_{16}{}^{35}ClNO_3+H]^+$ requires 306.0897; $[\alpha]_D^{27}$ -17.0 (c 0.1, MeOH).

*(S)-1-(3-Iodo-4-hydroxybenzyl)-1,2,3,4-tetrahydroisoquinoline-6,7-diol (S)-**27**[2]*

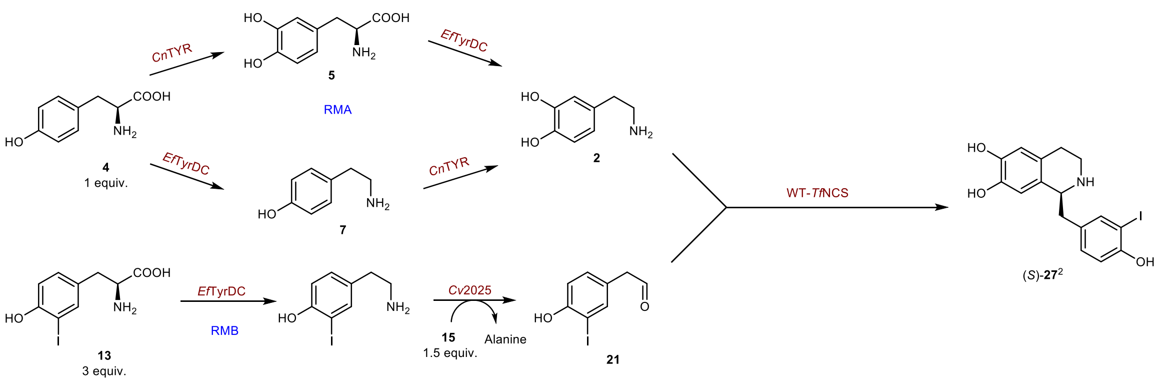

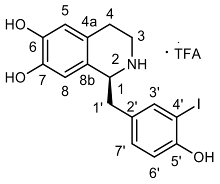

To synthesize the amine moiety, reaction mixture A (RMA, 50 mL, pH 5.5) consisted of 50 mM HEPES, 2.5 mM L-tyrosine **4**, 10 mM sodium ascorbate **8**, 40 μM $CuSO_4{\cdot}5H_2O$ and 1.25 mM PLP. To initiate the hydroxylation and decarboxylation steps, 10% (v/v) of *Cn*TYR lysates and 10% (v/v) of *Ef*TyrDC lysates were added to RMA which was incubated at 25 °C, 250 rpm for 6 h. For the aldehyde moiety, reaction mixture B (RMB, 50 mL, pH 7.5) consisted of 10% (v/v) of MeCN, 50 mM HEPES, 7.5 mM 3-I-L-tyrosine **13**, 7.5 mM sodium ascorbate **8**, 1.25 mM PLP and 7.5 mM sodium pyruvate **15**. To initiate the decarboxylation and transamination steps, 10% (v/v) of *Ef*TyrDC lysates and 5% (v/v) of *Cv*TAm lysates were added to RMB and the solution was incubated at 37 °C, 250 rpm for 6 h. Then RMA and RMB were mixed. The Pictet-Spengler condensation was performed with 10% (v/v) of *Tf*NCS lysates at 37 °C, 250 rpm for 8 h. In control reactions, all the enzymes were replaced

by empty-vector cell lysates. The product was purified using preparative HPLC (method 5, Vydac™ 218TP1022 (C18, 10 µm, 2.2 cm x 25 cm) preparative column, retention time: 11.0 min, run time: 28 mins, flow rate: 8 mL/min). Fractions containing the desired product were freeze-dried to give (*S*)-**27** as a yellow powder (TFA slats, yield by HPLC (calibration curve) 85% (method 1, ACE 5 C18 column (150 × 4.6 mm), retention time: 5.7 min, run time: 10 mins, flow rate: 1 mL/min); final isolated yield 40% (24.7 mg); *ee.* 92% (chiral HPLC, method 2, Supelco Astec Chirobiotic™ T2 column (25 cm × 4.6 mm), retention time: 22.8 min, run time: 80 mins, flow rate: 1 mL/min). ^{1}H NMR (600 MHz; CD_3OD) δ = 7.67 (1H, d, *J* = 1.8 Hz, 3'-H), 7.12 (1H, dd, *J* = 7.8 Hz, 1.8 Hz, 7'-H), 6.84 (1H, d, *J* = 7.8 Hz, 6'-H), 6.62 (1H, s, 5-H), 6.59 (1H, s, 8-H), 4.58 (1H, dd, *J* = 8.4 Hz, 5.7 Hz, 1-H), 3.47-3.43 (1H, m, 3-HH), 3.35 (1H, dd, *J* = 15.0 Hz, 5.7 Hz, 1'-HH), 3.27-3.22 (1H, m, 3-HH), 3.01-2.87 (3H, m, 1'-HH, 4-HH and 4-HH); ^{13}C NMR (151 MHz; CD_3OD) δ = 157.8, 146.9, 145.8, 141.3, 131.7, 129.2, 123.7, 123.5, 116.2, 116.2, 114.2, 85.3, 57.8, 41.0, 39.8, 25.7; *m/z* [ES+] 398 ([M+H]+, 100%); *m/z* [HRMS ES+] found $[M+H]^+$ 398.0248. $[C_{16}H_{16}INO_3+H]^+$ requires 398.0253; $[\alpha]_D^{27}$ -7.0 (c 0.44, MeOH).

*(S)-1-(4-Hydroxybenzyl)-1,2,3,4-tetrahydroisoquinolin-6-ol (S)-**28***[3]

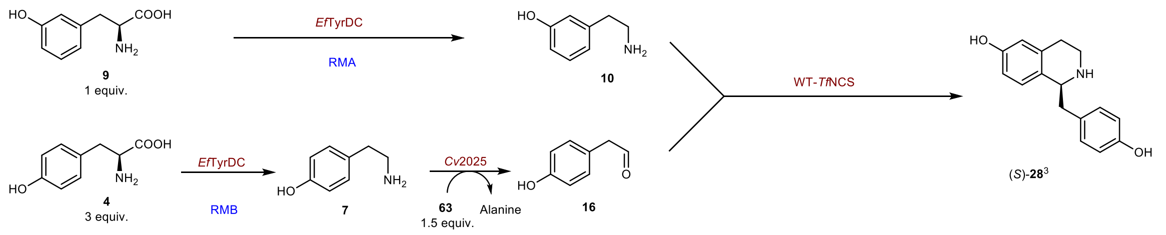

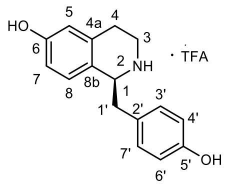

To synthesize the amine moiety, reaction mixture A (RMA, 20 mL, pH 7.5) consisted of 50 mM HEPES, 10 mM *meta*-L-tyrosine **9** and 5 mM PLP. To initiate the decarboxylation step, 10% (v/v) of *Ef*TyrDC lysates was added to RMA which was incubated at 25 °C, 250 rpm for 6 h. For the aldehyde moiety, reaction mixture B (RMB, 240 mL, pH 7.5) consisted of 10% (v/v) MeCN, 50 mM HEPES, 2.5 mM L-tyrosine **4**, 2.5 mM sodium ascorbate **8**, 1.25

mM PLP and 2.5 mM sodium pyruvate **15**. To initiate the decarboxylation and transamination steps, 10% (v/v) of *Ef*TyrDC lysates and 5% (v/v) of *Cv*TAm lysates were added to RMB and was incubated at 37 °C, 250 rpm for 6 h. Then RMA and RMB were mixed. The Pictet-Spengler condensation was performed with 10% (v/v) of *Tf*NCS lysates at 37 °C, 250 rpm for 8 h. In control reactions, all the enzymes were replaced by empty-vector cell lysates. The product was purified using preparative HPLC (method 4, Vydac™ 218TP1022 (C18, 10 μm, 2.2 cm x 25 cm) preparative column, retention time: 16.4 min, run time: 28 mins, flow rate: 8 mL/min). Fractions containing the desired product were freeze-dried to give (*S*)-**28** as a white powder (TFA salt, yield by HPLC (calibration curve) 76% (method 1, ACE 5 C18 column (150 × 4.6 mm), retention time: 5.2 min, run time: 10 mins, flow rate: 1 mL/min); final isolated yield 42% (29.6 mg); *ee.* 92% (chiral HPLC, method 2, Supelco Astec Chirobiotic™ T2 column (25 cm × 4.6 mm), retention time: 18.7 min, run time: 40 mins, flow rate: 1 mL/min). ^{1}H NMR (600 MHz; CD_3OD) δ = 7.11 (2H, d, *J* = 8.4 Hz, 3'-H and 7'-H), 7.00 (1H, d, *J* = 8.4 Hz, 8-H), 6.80 (2H, d, *J* = 8.4 Hz, 4'-H and 6'-H), 6.69-6.65 (2H, m, 5-H and 7-H), 4.63 (1H, dd, *J* = 8.4 Hz, 5.7 Hz, 1-H), 3.50-3.46 (1H, m, 3-HH), 3.38 (1H, dd, *J* = 14.4 Hz, 5.7 Hz, 1'-HH), 3.29-3.26 (1H, m, 3-HH), 3.10-3.05 (1H, m, 1'-HH), 3.02-2.97 (2H, m, 4-HH and 4-HH); ^{13}C NMR (150 MHz; CD_3OD) δ = 158.5, 158.2, 134.0, 131.7, 129.2, 127.0, 123.6, 116.9, 116.0, 115.6, 58.0, 40.5, 40.4, 26.4; *m/z* [ES+] 256 ([M+H]$^+$, 100%); *m/z* [HRMS ES+] found [M+H]$^+$ 256.1342. [$C_{16}H_{16}FNO_2$+H]$^+$ requires 256.1338; $[\alpha]_D^{28}$ -6.0 (c 0.32, MeOH).

*(S)-1-(3-Fluoro-4-hydroxybenzyl)-1,2,3,4-tetrahydroisoquinolin-6-ol (S)-**29***

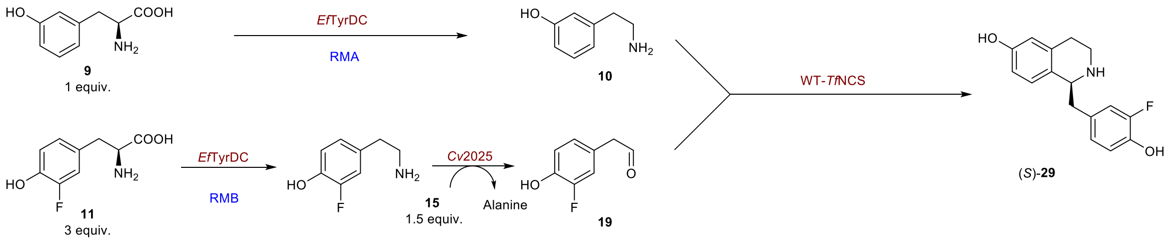

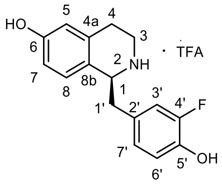

To synthesize the amine moiety, reaction mixture A (RMA, 20 mL, pH 7.5) consisted of 50 mM HEPES, 10 mM *meta*-L-tyrosine **9** and 5 mM PLP. To initiate the decarboxylation step, 10% (v/v) of *Ef*TyrDC lysates was added to RMA which was incubated at 25 °C, 250 rpm for 6 h. For the aldehyde moiety, reaction mixture B (RMB, 20 mL, pH 7.5) consisted of 10% (v/v) MeCN, 50 mM HEPES, 30 mM 3-F-L-tyrosine **11**, 30 mM sodium ascorbate **8**, 5 mM PLP and 30 mM sodium pyruvate **15**. To initiate the decarboxylation and transamination steps, 10% (v/v) of *Ef*TyrDC lysates and 5% (v/v) of *Cv*TAm lysates were added to RMB and was incubated at 37 °C, 250 rpm for 6 h. Then RMA and RMB were mixed. The Pictet-Spengler condensation was performed with 10% (v/v) of *Tf*NCS lysates at 37 °C, 250 rpm for 8 h. In control reactions, all the enzymes were replaced by empty-vector cell lysates. The product was purified using preparative HPLC (method 4, Vydac^{TM} 218TP1022 (C18, 10 µm, 2.2 cm x 25 cm) preparative column, retention time: 17.2 min, run time: 28 mins, flow rate: 8 mL/min). Fractions containing the desired product were freeze-dried to give (*S*)-**29** as a yellow powder (TFA salt, yield by HPLC (calibration curve) 58% (method 1, ACE 5 C18 column (150 × 4.6 mm), retention time: 5.3 min, run time: 10 mins, flow rate: 1 mL/min); final isolated yield 21% (20 mg); *ee.* 85% (chiral HPLC, method 2, Supelco Astec Chirobiotic^{TM} T2 column (25 cm × 4.6 mm), retention time: 17.8 and 19.8 min, run time: 40 mins, flow rate: 1 mL/min). ^{1}H NMR (600 MHz; CD_3OD) δ = 7.05-7.01 (2H, m, 6'-H and 8-H), 6.93-6.81 (2H, m, 5-H and 7-H), 6.70-6.66 (2H, m, 3'-H and 7'-H), 4.66 (1H, dd, *J* = 8.4 Hz, 6.0 Hz, 1-H), 3.52-3.47 (1H, m, 3-H<u>H</u>), 3.40 (1H, dd, *J* = 15.0 Hz, 6.0 Hz, 1'-H<u>H</u>), 3.11-3.06(1H, m, 3-<u>H</u>H), 3.03-2.98 (2H, m, 1'-<u>H</u>H), 3.02-2.98 (2H, m, 4-H<u>H</u> and 4-<u>H</u>H); ^{13}C NMR (150 MHz; CD_3OD) δ = 158.6, 153.0 (d, $^1J_{CF}$ = 214.4 Hz), 145.7 (d, $^2J_{CF}$ = 55.9 Hz), 134.0, 129.1, 127.5 (d, $^3J_{CF}$ = 24.2 Hz), 126.8, 123.4 (d, $^4J_{CF}$ = 7.6 Hz), 118.0 (d, $^3J_{CF}$ = 34.7 Hz), 117.8 (d, $^2J_{CF}$ = 36.24 Hz), 116.1, 115.7, 57.8, 40.6, 40.2, 26.4; *m/z* [ES+] 274 ([M+H]$^+$, 100%); *m/z* [HRMS ES+] found [M+H]$^+$ 274.1241. [$C_{16}H_{16}FNO_2$+H]$^+$ requires 274.1243; $[\alpha]_D^{28}$ -9.0 (c 0.25, MeOH).

*(S)-1-(3-Chloro-4-hydroxybenzyl)-1,2,3,4-tetrahydroisoquinolin-6-ol (S)-**30***

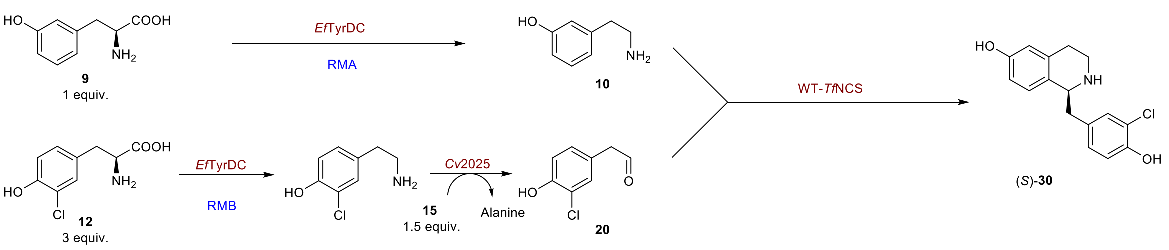

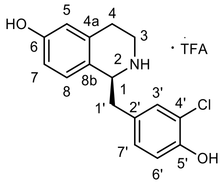

To synthesize the amine moiety, reaction mixture A (RMA, 20 mL, pH 7.5) consisted of 50 mM HEPES, 10 mM *meta*-L-tyrosine **9** and 5 mM PLP. To initiate the decarboxylation step, 10% (v/v) of *Ef*TyrDC lysates was added to RMA which was incubated at 25 °C, 250 rpm for 6 h. For the aldehyde moiety, reaction mixture B (RMB, 20 mL, pH 7.5) consisted of 10% (v/v) MeCN, 50 mM HEPES, 30 mM 3-Cl-L-tyrosine **12**, 30 mM sodium ascorbate **8**, 5 mM PLP and 30 mM sodium pyruvate **15**. To initiate the decarboxylation and transamination steps, 10% (v/v) of *Ef*TyrDC lysates and 5% (v/v) of *Cv*TAm lysates were added to RMB and was incubated at 37 °C, 250 rpm for 6 h. Then RMA and RMB were mixed. The Pictet-Spengler condensation was performed with 10% (v/v) of *Tf*NCS lysates at 37 °C, 250 rpm for 8 h. In control reactions, all the enzymes were replaced by empty-vector cell lysates. The product was purified using preparative HPLC (method 5, Vydac^{TM} 218TP1022 (C18, 10 µm, 2.2 cm x 25 cm) preparative column, retention time: 12.0 min, run time: 28 mins, flow rate: 8 mL/min). Fractions containing the desired product were freeze-dried to give (*S*)-**30** as a yellow powder (TFA slats, yield by HPLC (calibration curve) 67% (method 1, ACE 5 C18 column (150 × 4.6 mm), retention time: 5.6 min, run time: 10 mins, flow rate: 1 mL/min); final isolated yield 35% (27.1 mg); *ee.* 95% (chiral HPLC, method 2, Supelco Astec Chirobiotic^{TM} T2 column (25 cm × 4.6 mm), retention time: 19.5 min, run time: 40 mins, flow rate: 1 mL/min). ^{1}H NMR (600 MHz; CD_3OD) δ = 7.27 (1H, d, *J* = 1.8 Hz, 3'-H), 7.06 (1H, dd, *J* = 8.4 Hz, 1.8 Hz, 7'-H), 6.99 (1H, d, *J* = 8.4 Hz, 8-H), 6.92 (1H, d, *J* = 8.4 Hz, 6'-H), 6.69-6.65 (2H, m, 5-H and 7-H), 4.65 (1H, dd, *J* = 9.0 Hz, 6.0 Hz, 1-H), 3.51-3.47 (1H, m, 3-HH), 3.38 (1H, dd, *J* = 14.4 Hz, 6.0 Hz, 1'-HH), 3.30-3.27 (1H, m, 3-HH), 3.11-3.06 (1H, m, 1'-HH), 3.02-2.98 (2H, m, 4-HH and 4-HH); ^{13}C NMR (151 MHz; CD_3OD) δ = 158.6,

154.0, 134.1, 132.1, 130.2 129.2, 128.6, 123.4, 122.2, 118.1, 116.1, 115.6, 57.7, 40.6, 40.0, 26.4; *m/z* [ES+] 290 ($[M+H]^+$, 100%); *m/z* [HRMS ES+] found $[M+H]^+$ 290.0943. $[C_{16}H_{16}{}^{35}ClNO_2+H]^+$ requires 290.0948; $[\alpha]_D^{28}$ -0.4 (c 0.48, MeOH).

*(S)-1-(3-Iodo-4-hydroxybenzyl)-1,2,3,4-tetrahydroisoquinolin-6-ol (S)-**31***

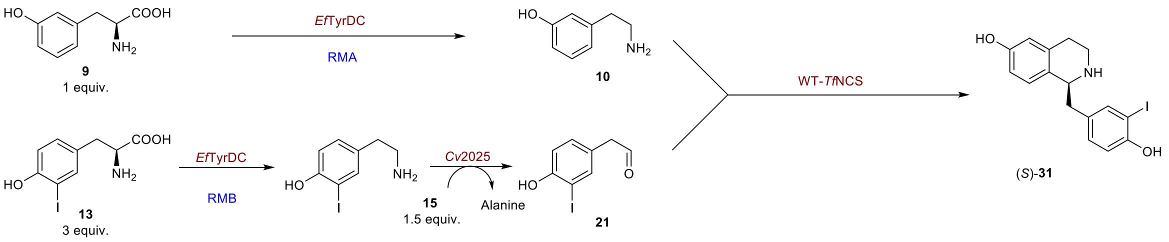

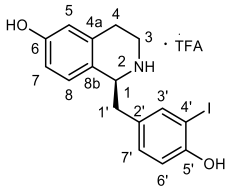

To synthesize the amine moiety, reaction mixture A (RMA, 20 mL, pH 7.5) consisted of 50 mM HEPES, 10 mM *meta*-L-tyrosine **9** and 5 mM PLP. To initiate the decarboxylation step, 10% (v/v) of *Ef*TyrDC lysates was added to RMA which was incubated at 25 °C, 250 rpm for 6 h. For the aldehyde moiety, reaction mixture B (RMB, 20 mL, pH 7.5) consisted of 10% (v/v) MeCN, 50 mM HEPES, 30 mM 3-I-L-tyrosine **13**, 30 mM sodium ascorbate **8**, 5 mM PLP and 30 mM sodium pyruvate **15**. To initiate the decarboxylation and transamination steps, 10% (v/v) of *Ef*TyrDC lysates and 5% (v/v) of *Cv*TAm lysates were added to RMB and was incubated at 37 °C, 250 rpm for 6 h. Then RMA and RMB were mixed. The Pictet-Spengler condensation was performed with 10% (v/v) of *Tf*NCS lysates at 37 °C, 250 rpm for 8 h. In control reactions, all the enzymes were replaced by empty-vector cell lysates. The product was purified using preparative HPLC (method 5, Vydac™ 218TP1022 (C18, 10 µm, 2.2 cm x 25 cm) preparative column, retention time: 13.5 min, run time: 28 mins, flow rate: 8 mL/min). Fractions containing the desired product were freeze-dried to give (*S*)-**31** as a yellow powder (TFA salt, yield by HPLC (calibration curve) 68% (method 1, ACE 5 C18 column (150 × 4.6 mm), retention time: 6.0 min, run time: 10 mins, flow rate: 1 mL/min); final isolated yield 35% (33.5 mg); *ee.* 91% (chiral HPLC, method 2, Supelco Astec Chirobiotic™ T2 column (25 cm × 4.6 mm), retention time: 21.2 min, run time: 40 mins, flow

rate: 1 mL/min). ^{1}H NMR (600 MHz; CD_3OD) δ = 7.65 (1H, d, *J* = 1.8 Hz, 3'-H), 7.13 (1H, dd, *J* = 8.4 Hz, 2.4 Hz, 7'-H), 7.00 (1H, d, *J* = 8.4 Hz, 8-H), 6.84 (1H, d, *J* = 8.4 Hz, 6'-H), 6.70-6.65 (2H, m, 5-H and 7-H), 4.63 (1H, dd, *J* = 8.4 Hz, 6.0 Hz, 1-H), 3.51-3.47 (1H, m, 3-HH), 3.38 (1H, dd, *J* = 15.0 Hz, 6.0 Hz, 1'-HH), 3.30-3.26 (1H, m, 3-HH), 3.10-3.05 (1H, m, 1'-HH), 3.02-2.94 (2H, m, 4-HH and 4-HH); ^{13}C NMR (150 MHz; CD_3OD) δ = 158.6, 157.8, 141.4, 134.0, 131.8, 129.2, 123.4, 116.2, 116.1, 115.6, 85.3, 57.8, 40.6, 39.8, 26.4; *m/z* [ES+] 382 ($[M+H]^+$, 100%); *m/z* [HRMS ES+] found $[M+H]^+$ 382.0302. $[C_{16}H_{16}INO_2+H]^+$ requires 382.0304; $[\alpha]_D^{28}$ -18.0 (c 1.0, MeOH).

*(S)-1-Benzyl-8-fluoro-1,2,3,4-tetrahydroisoquinoline-6,7-diol (S)-**45**[4]*

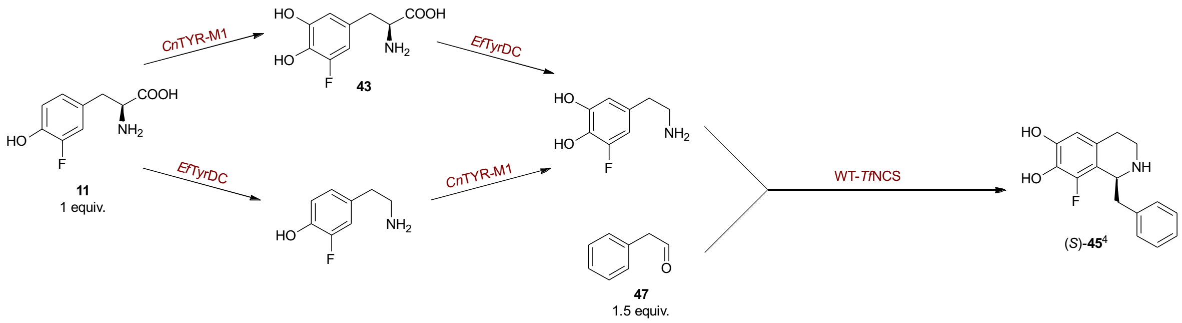

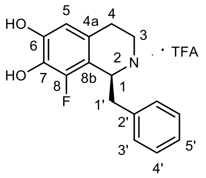

The reaction mixture (20 mL, pH 5.5) consisted of 10% (v/v) of MeCN, 50 mM HEPES, 10 mM 3-F-L-tyrosine **11**, 40 mM sodium ascorbate **8**, $CuSO_4 \cdot 5H_2O$ (40 μM for WT-*Cn*TYR and 5 μM for *Cn*TYR-variants) and 5 mM PLP. To initiate the hydroxylation and decarboxylation steps, 10% (v/v) of *Cn*TYR or *Cn*TYR-variant lysates and 10% (v/v) of *Ef*TyrDC lysates were added to the reaction mixture which was incubated at 25 °C, 250 rpm for 24 h with *Cn*TYR or 8 h with *Cn*TYR-variants. Then, the reaction was adjusted to pH 7.5 with 2.5 M NaOH and 15 mM phenylacetaldehyde **47** were added to the reaction mixture. The Pictet-Spengler condensation was performed with 10% (v/v) of *Tf*NCS lysates at 37 °C, 250 rpm for 16 h. In control reactions, all the enzymes were replaced by empty-vector cell lysates. The product was purified using preparative HPLC (method 4, Supelco™ Discovery BIO wide pore (C18, 10 μm, 2.12 cm x 25 cm) preparative column, retention time: 18.6 min,

run time: 28 mins, flow rate: 8 mL/min). Fractions containing the desired product were freeze-dried to give (*S*)-**45** as an off-white powder (TFA salt, yield by HPLC (calibration curve) 35% with *Cn*TYR and 89% with *Cn*TYR-M1 (N201S/G205R/V206I) (method 1, ACE 5 C18 column (150 × 4.6 mm), retention time: 5.5 min, run time: 10 mins, flow rate: 1 mL/min); final isolated yield 23% (17 mg) with *Cn*TYR and 68% (50.3 mg) with *Cn*TYR-M1 (N201S/G205R/V206I); *ee.* 91% (chiral HPLC, method 2, Supelco Astec Chirobiotic™ T column (25 cm × 4.6 mm), retention time: 12 min, run time: 40 mins, flow rate: 1 mL/min). ^{1}H NMR (600 MHz; CD_3OD) δ = 7.39-7.30 (5H, m, Ph-H), 6.51 (1H, s, 5-H), 4.91 (1H, dd, *J* = 9.5 Hz, 4.3 Hz, 1-H), 3.50 (1H, ddd, *J* = 12.6 Hz, 8.4 Hz, 6.6 Hz, 3-HH), 3.41 (1H, dd, *J* = 15.0 Hz, 4.3 Hz, 1'-HH), 3.29-3.26 (1H, m, 3-HH), 3.16 (1H, dd, *J* = 15.0 Hz, 9.5 Hz, 1'-HH), 2.97-2.93 (2H, m, 4-HH and 4-HH); ^{13}C NMR (151 MHz; CD_3OD) δ = 150.2 (d, $^1J_{CF}$ = 237.0 Hz), 149.1 (d, $^3J_{CF}$ = 7.5 Hz), 136.5, 133.8 (d, $^3J_{CF}$ = 15.0 Hz), 130.5, 130.3, 128.9, 123.2 (d, $^2J_{CF}$ = 4.5 Hz), 112.1 (d, $^2J_{CF}$ = 12.0 Hz), 111.8 (d, $^4J_{CF}$ = 1.5 Hz), 53.3, 39.9, 38.9, 25.2; *m/z* [ES+] 274 ($[M+H]^+$, 100%); *m/z* [HRMS ES+] found $[M+H]^+$ 274.1234. $[C_{16}H_{16}FNO_2+H]^+$ requires 274.1243; $[\alpha]_D^{25}$ 5.8 (c 0.45, MeOH).

*(S)-1-Benzyl-8-fluoro-6-methoxy-1,2,3,4-tetrahydroisoquinolin-7-ol (S)-**46***[5]

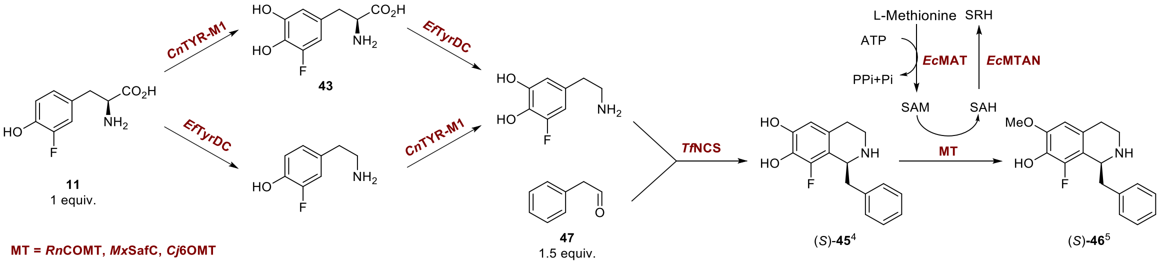

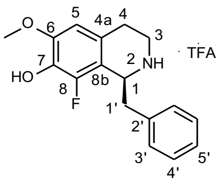

Flowing the synthesis step of (*S*)-**45**, the solution containing (*S*)-**45** was then spin down, and 40 mM $MgCl_2$, 200 mM KCl, 20 mM L-methionine and 20 mM ATP were added to the solution. To initial the methylation step, 10% (v/v) of *Rn*COMT/*Mx*SafC/*Cj*6OMT lysates, 10% (v/v) of *Ec*MAT lysates and 2.5% (v/v) of *Ec*MTAN lysates were added to the supernatant which was incubated at 37 °C, 250 rpm for another 8 h. In control reactions, all the enzymes

were replaced by empty-vector cell lysates. The reaction mixture was then spin down and the supernatant was extracted with ethyl acetate (20 mL x 3). The organic layers were combined and dried with $MgSO_4$. Solvents were removed under vacuum. Then the extracted crude compounds were redissolved in 5 mL of H_2O: MeCN = 1:1. The product was purified using preparative HPLC (method 4, Supelco^{TM} Discovery BIO wide pore (C18, 10 μm, 2.12 cm x 25 cm) preparative column, retention time: 19.4 min, run time: 28 mins, flow rate: 8 mL/min). Fractions containing the desired product were freeze dried to give (S)-**46** as an off-white powder (TFA salt, yield by HPLC (calibration curve) is listed in Supplementary Figure 4; (method 1, ACE 5 C18 column (150 × 4.6 mm), retention time: 6.3 min, run time: 10 mins, flow rate: 1 mL/min); final isolated yield is 56% (43 mg); *ee.* 95% (chiral HPLC, method 2, Supelco Astec Chirobiotic^{TM} T2 column (25 cm × 4.6 mm), retention time: 14.2 min, run time: 40 mins, flow rate: 1 mL/min). ^{1}H NMR (700 MHz; CD_3OD) δ = 7.39-7.37 (2H, m, 4'-H and 6'-H), 7.33-7.31 (3H, m, 3'-H, 5'-H and 6'-H), 6.68 (1H, s, 5-H), 4.93 (1H, dd, *J* = 9.8 Hz, 4.6 Hz, 1-H), 3.87 (1H, s, OCH_3), 3.52 (1H, dt, *J* = 13.3 Hz, 7.7 Hz, 3-HH), 3.42 (1H, dd, *J* = 14.7 Hz, 4.6 Hz, 1'-HH), 3.34-3.32 (1H, m, 3-HH), 3.17 (1H, dd, *J* = 14.7 Hz, 9.8 Hz, 1'-HH), 3.04-3.02 (2H, m, 4-HH and 4-HH); ^{13}C NMR (175 MHz; CD_3OD) δ = 151.0 (d, $^3J_{CF}$ = 5.4 Hz), 149.5 (d, $^1J_{CF}$ = 238.0 Hz), 136.3, 134.9 (d, $^2J_{CF}$ = 14.0 Hz), 130.4, 130.2, 128.8, 123.0 (d, $^3J_{CF}$ = 3.5 Hz), 113.7 (d, $^2J_{CF}$ = 12.3 Hz), 108.1 (d, $^4J_{CF}$ = 1.8 Hz), 56.7, 53.2, 39.6, 38.8, 25.2; *m/z* [ES+] 288 ($[M+H]^+$, 100%); *m/z* [HRMS ES+] found $[M+H]^+$ 288.1394; $[C_{17}H_{18}FNO_2+H]^+$ requires 288.1394; $[\alpha]_D^{26}$ 2.0 (c 0.2, MeOH).

*(S)-1-Benzyl-8-chloro-1,2,3,4-tetrahydroisoquinoline-6,7-diol (S)-**48***

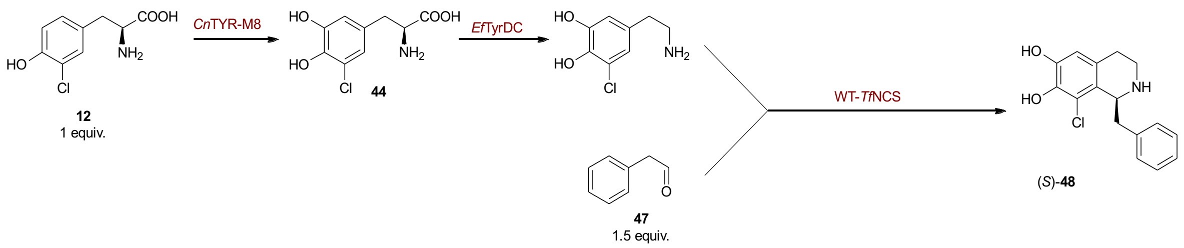

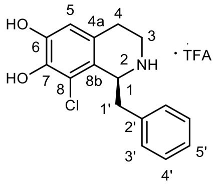

The reaction mixture (20 mL, pH 5.5) consisted of 10% (v/v) of MeCN, 50 mM HEPES, 10 mM 3-Cl-L-tyrosine **12**, 40 mM sodium ascorbate **8**, $CuSO_4 \cdot 5H_2O$ (40 μM for WT-*Cn*TYR and 5 μM for *Cn*TYR-variants) and 5 mM PLP. To initiate the hydroxylation and decarboxylation steps, 10% (v/v) of *Cn*TYR or *Cn*TYR-variant lysates and 10% (v/v) of *Ef*TyrDC lysates were added to the reaction mixture which was incubated at 25 °C, 250 rpm for 24 h with *Cn*TYR or *Cn*TYR-variants. Then, the reaction was adjusted to pH 7.5 with 2.5 M NaOH and 15 mM phenylacetaldehyde **47** were added to the reaction mixture. The Pictet-Spengler condensation was performed with 10% (v/v) of *Tf*NCS lysates at 37 °C, 250 rpm for 24 h. In control reactions, all the enzymes were replaced by empty-vector cell lysates. The product was purified using preparative HPLC (method 4, Supelco™ Discovery BIO wide pore (C18, 10 μm, 2.12 cm x 25 cm) preparative column, retention time: 20.2 min, run time: 28 mins, flow rate: 8 mL/min). Fractions containing the desired product were freeze-dried to give (*S*)-**48** as a yellow powder (TFA salt, yield by HPLC (calibration curve) 45% (method 1, ACE 5 C18 column (150 × 4.6 mm), retention time: 5.9 min, run time: 10 mins, flow rate: 1 mL/min); final isolated yield 31% (23.9 mg); *ee.* 92% (chiral HPLC, method 2, Supelco Astec Chirobiotic™ T2 column (25 cm × 4.6 mm), retention time: 19.8 min, run time: 40 mins, flow rate: 1 mL/min). ^{1}H NMR (700 MHz; CD_3OD) δ = 7.41-7.34 (5H, m, Ph-H), 6.55 (1H, s, 5-H), 4.93 (1H, dd, *J* = 10.2 Hz, 4.2 Hz, 1-H), 3.66-3.61 (1H, m, 3-H$\underline{H}$), 3.46 (1H, dd, *J* = 14.7 Hz, 4.2 Hz, 1'-H$\underline{H}$), 3.34-3.32 (1H, m, 3-$\underline{H}$H), 3.11 (1H, dd, *J* = 14.7 Hz, 10.2 Hz, 1'-$\underline{H}$H), 3.06-2.95 (2H, m, 4-H$\underline{H}$ and 4-$\underline{H}$H); ^{13}C NMR (175 MHz; CD_3OD) δ = 147.9, 142.9, 136.5, 130.3, 130.2, 128.9, 124.2, 121.4, 119.5, 114.7, 55.6, 38.3, 37.4, 25.1; *m/z* [ES+] 290 ($[M+H]^+$, 100%); *m/z* [HRMS ES+] found $[M+H]^+$ 290.0948. $[C_{16}H_{16}{}^{35}ClNO_2+H]^+$ requires 290.0945; $[\alpha]_D^{25}$ -6.0 (c 0.19, MeOH).

(S)-1-Benzyl-1,2,3,4-tetrahydroisoquinoline-5,6-diol ***49***

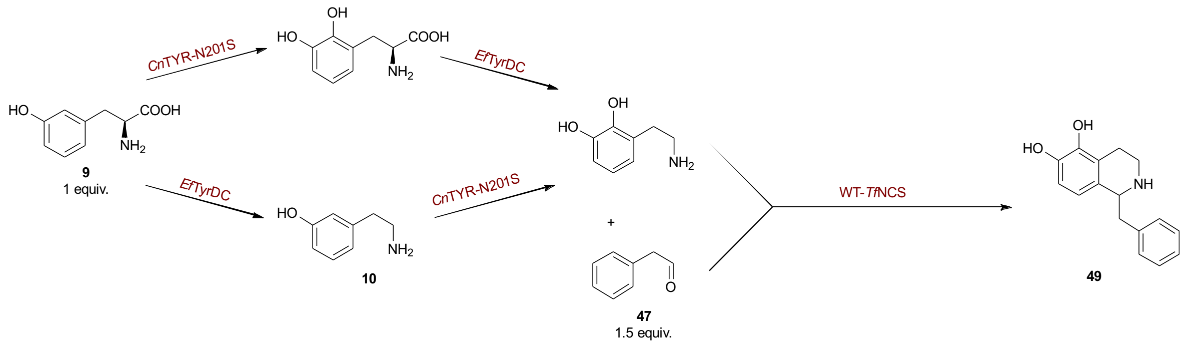

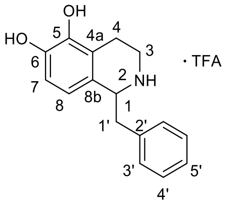

The reaction mixture (20 mL, pH 5) consisted of 10% (v/v) MeCN, 50 mM HEPES, 10 mM *meta*-L-tyrosine **9**, 40 mM sodium ascorbate **8**, 5 µM $CuSO_4{\cdot}5H_2O$ and 5 mM PLP. To initiate the hydroxylation and decarboxylation steps, 10% (v/v) of *Cn*TYR-N201S lysates and 10% (v/v) of *Ef*TyrDC lysates were added to the reaction mixture which was incubated at 25 °C, 250 rpm for 36 h. Then, the reaction was adjusted to pH 7.5 with 2.5 M NaOH and 15 mM phenylacetaldehyde **47** were added to the reaction mixture. The Pictet-Spengler reaction was performed with 10% (v/v) of *Tf*NCS lysates at 37 °C, 250 rpm for 24 h. In control reactions, all the enzymes were replaced by empty-vector cell lysates. The product was purified using preparative HPLC (method 4, Supelco™ Discovery BIO wide pore (C18, 10 µm, 2.12 cm x 25 cm) preparative column, retention time: 15.1 min, run time: 28 mins, flow rate: 8 mL/min). Fractions containing the desired product were freeze-dried to give (*S*)-**111** as a white powder (TFA salt, yield by HPLC (calibration curve) 12% (method 1, ACE 5 C18 column (150 × 4.6 mm), retention time: 4.8 min, run time: 10 mins, flow rate: 1 mL/min); final isolated yield 5% (3.5 mg). ^{1}H NMR (700 MHz; CD_3OD) δ = 7.22-7.14 (5H, m, Ph-H), 6.97 (1H, d, *J* = 7.7 Hz, 8-H), 6.65 (1H, d, *J* = 7.7 Hz, 7-H), 4.72 (1H, dd, *J* = 8.4 Hz, 6.3 Hz, 1-H), 3.52-3.49 (1H, m, 3-HH), 3.34-3.32 (1H, m, 1'-HH), 3.27-3.23 (2H, m, 3-HH and 1'-HH), 3.11-3.07 (2H, m, 4-HH and 4-HH); ^{13}C NMR (175 MHz; CD_3OD) δ = 145.9, 145.8, 139.1, 130.6, 128.7, 127.3, 124.2, 121.0, 115.1, 110.4, 60.5, 42.1, 40.7, 25.2; *m/z* [ES+] 255 ([M+H]$^+$, 100%); *m/z* [HRMS ES+] found [M+H]$^+$ 256.1335. [$C_{16}H_{17}NO_2$+H]$^+$ requires 255.1332.

*(S)-1-(2-Bromobenzyl)-8-fluoro-1,2,3,4-tetrahydroisoquinoline-6,7-diol (S)-**51***

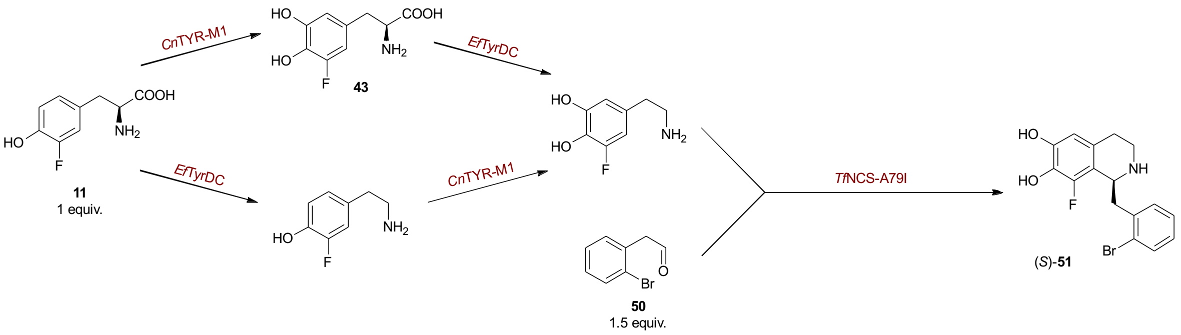

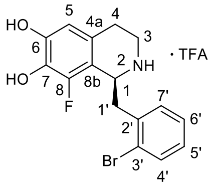

The reaction mixture (20 mL, pH 5) consisted of 10% (v/v) of MeCN, 50 mM HEPES, 10 mM 3-F-L-tyrosine **11**, 40 mM sodium ascorbate **8**, 5 µM $CuSO_4{\cdot}5H_2O$ and 5 mM PLP. To initiate the hydroxylation and decarboxylation steps, 10% (v/v) of *Cn*TYR-M1 (N201S/G205R/V206I) lysates and 10% (v/v) of *Ef*TyrDC lysates were added to the reaction mixture which was incubated at 25 °C, 250 rpm for 8 h. Then the solution was adjusted to pH 7.5 with 2.5 M NaOH and 15 mM 2-bromophenylacetaldehyde **50** were added to the solution. The Pictet-Spengler condensation was performed with 10% (v/v) of *Tf*NCS-A79I lysates at 37 °C, 250 rpm for 16 h. In control reactions, all the enzymes were replaced by empty-vector cell lysates. The product was purified using preparative HPLC (method 4, Supelco™ Discovery BIO wide pore (C18, 10 µm, 2.12 cm x 25 cm) preparative column, retention time: 19.3 min, run time: 28 mins, flow rate: 8 mL/min). Fractions containing the desired product were freeze-dried to give (*S*)-**51** as an orange powder (TFA salt, yield by HPLC (calibration curve) 83% (method 1, ACE 5 C18 column (150 × 4.6 mm), retention time: 6.3 min, run time: 10 mins, flow rate: 1 mL/min); final isolated yield 57% (51.2 mg); *ee.* 95% (chiral HPLC, method 2, Supelco Astec Chirobiotic™ T2 column (25 cm × 4.6 mm), retention time: 18 min and 31 min, run time: 80 mins, flow rate: 1 mL/min). ^{1}H NMR (700 MHz; CD_3OD) δ = 7.65 (1H, dd, *J* = 7.7 Hz, 1.4 Hz, 4'-H), 7.34 (1H, td, *J* = 7.7 Hz, 1.4 Hz, 6'-H), 7.27 (1H, dd, *J* = 7.0 Hz, 1.4 Hz, 7'-H), 7.24 (1H, td, *J* = 7.7 Hz, 1.4 Hz, 5'-H), 6.52 (1H, s, 5-H), 5.03 (1H, dd, *J* = 7.7 Hz, 7.0 Hz, 1-H), 3.74-3.69 (1H, m, 3-HH), 3.51 (1H, dd, *J* = 14.0 Hz, 7.0 Hz, 1'-HH), 3.42-3.35 (2H, m, 3-HH and 1'-HH), 3.06-3.00 (2H, m, 4-HH

and 4-HH); ^{13}C NMR (175 MHz; CD_3OD) δ = 150.8 (d, $^1J_{CF}$ = 238.0 Hz), 149.1 (d, $^3J_{CF}$ = 7.0 Hz), 135.6, 134.4, 133.5 (d, $^2J_{CF}$ = 15.8 Hz), 133.1, 130.8, 129.2, 125.8, 122.9 (d, $^3J_{CF}$ = 5.3 Hz), 111.8 (d, $^4J_{CF}$ = 3.5 Hz),111.4 (d, $^2J_{CF}$ = 14.0 Hz), 51.2, 39.8, 38.5, 24.8; *m/z* [ES+] 352 ($[M+H]^+$, 100%); *m/z* [HRMS ES+] found $[M+H]^+$ 352.0343. $[C_{16}H_{15}F^{79}BrNO_2+H]^+$ requires 352.0343; $[\alpha]_D^{26}$ 42.2 (c 0.19, MeOH).

*(S)-1-(2-Bromobenzyl)-8-chloro-1,2,3,4-tetrahydroisoquinoline-6,7-diol (S)-**52***

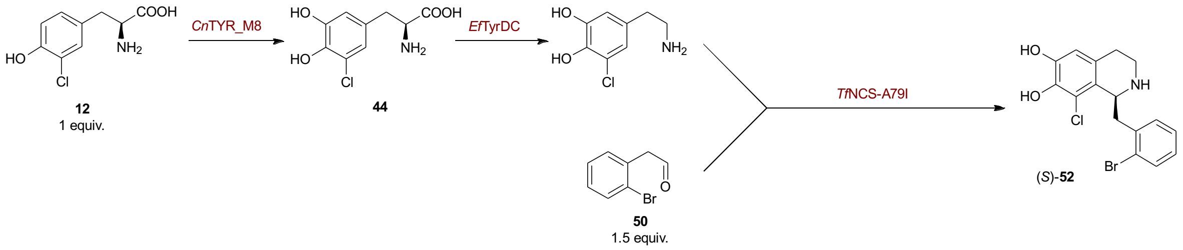

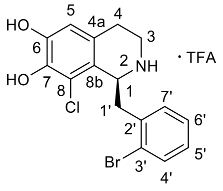

The reaction mixture (20 mL, pH 5) consisted of 10% (v/v) MeCN, 50 mM HEPES, 10 mM 3-Cl-L-tyrosine **12**, 40 mM sodium ascorbate **8**, 5 μM $CuSO_4{\cdot}5H_2O$ and 5 mM PLP. To initiate the hydroxylation steps, 10% (v/v) of *Cn*TYR-M8 (N201S/H202N) lysates and 10% (v/v) of *Ef*TyrDC lysates was added to the reaction mixture which was incubated at 25 °C, 250 rpm for 24 h. The solution was then adjusted to pH 7.5 with 2.5 M NaOH and 15 mM 2-bromophenylacetaldehyde **50** were added to the solution. The Pictet-Spengler condensation was performed with 10% (v/v) of *Tf*NCS-A79I lysates at 37 °C, 250 rpm for 24 h. In control reactions, all the enzymes were replaced by empty-vector cell lysates. The product was purified using preparative HPLC (method 4, Supelco™ Discovery BIO wide pore (C18, 10 μm, 2.12 cm x 25 cm) preparative column, retention time: 21.8 min, run time: 28 mins, flow rate: 8 mL/min). Fractions containing the desired product were freeze-dried to give (*S*)-**52** as a dark green powder (TFA salt, yield by HPLC (calibration curve) 16% (method 1, ACE 5 C18 column (150 × 4.6 mm), retention time: 6.7 min, run time: 10 mins, flow rate: 1 mL/min); final isolated yield 7% (6.5 mg); *ee.* 92% (chiral HPLC, method 2, Supelco Astec Chirobiotic™ T2 column (25 cm × 4.6 mm), retention time: 22.6 min and 32

min, run time: 80 mins, flow rate: 1 mL/min). ^{1}H NMR (700 MHz; CD_3OD) δ = 7.65 (1H, dd, *J* = 8.4 Hz, 0.7 Hz, 4'-H), 7.35-7.33 (1H, m, 6'-H), 7.30-7.23 (2H, m, 5'-H and 7'-H), 6.67 (1H, s, 5-H), 5.10 (1H, dd, *J* = 8.4 Hz, 6.3 Hz, 1-H), 3.80-3.76 (1H, m, 3-H<u>H</u>), 3.49 (1H, dd, *J* = 14.7 Hz, 6.3 Hz, 1'-H<u>H</u>), 3.45-3.39 (2H, m, 3-<u>H</u>H and 1'-<u>H</u>H), 3.34-3.32 (1H, m, 4-H<u>H</u>); 3.10-3.07 (1H, m, 4-<u>H</u>H); ^{13}C NMR (175 MHz; CD_3OD) δ = 147.3, 141.0, 138.8, 134.1, 130.9, 130.6, 130.4, 128.1, 127.3, 125.4, 124.6, 112.4, 52.5, 38.0, 36.2, 27.5; *m/z* [ES+] 367 ($[M+H]^+$, 100%); *m/z* [HRMS ES+] found $[M+H]^+$ 368.0044. $[C_{16}H_{15}{}^{35}Cl^{79}BrNO_2+H]^+$ requires 368.0047; $[\alpha]_D^{26}$ 37.4 (c 0.12, MeOH).

(S)-8-Fluoro-1-(3-fluoro-4,5-dihydroxybenzyl)-1,2,3,4-tetrahydroisoquinoline-6,7-diol (S)-53

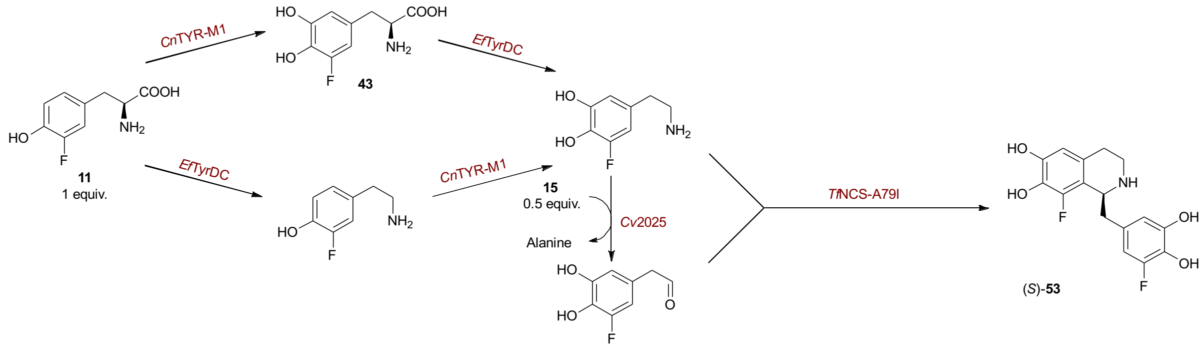

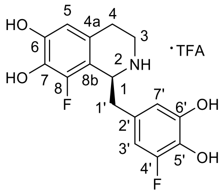

The reaction mixture (40 mL, pH 5) consisted of 50 mM HEPES, 10 mM 3-F-L-tyrosine **11**, 40 mM sodium ascorbate **8**, 5 μM $CuSO_4{\cdot}5H_2O$ and 5 mM PLP. To initiate the hydroxylation and decarboxylation steps, 10% (v/v) of *Cn*TYR-M1 (N201S/G205R/V206I) lysates and 10% (v/v) of *Ef*TyrDC lysates were added to the solution which was incubated at 25 °C, 250 rpm for 8 h. Then the solution was adjusted to pH 7.5 with 2.5 M NaOH and 5 mM sodium pyruvate **15**, 5% (v/v) of *Cv*TAm lysates and 10% (v/v) of *Tf*NCS-A79I lysates were performed at 37 °C, 250 rpm for 16 h. In control reactions, all the enzymes were replaced by empty-vector cell lysates. The product was purified using preparative HPLC (method 4, Supelco™ Discovery BIO wide pore (C18, 10 μm, 2.12 cm x 25 cm) preparative column, retention time: 15.4 min, run time: 28 mins, flow rate: 8 mL/min)). Fractions containing the

desired product were freeze-dried to give product (*S*)-**53** as an red powder (TFA salt, yield by HPLC (calibration curve) 26% (method 1, ACE 5 C18 column (150 × 4.6 mm), retention time: 4.8 min, run time: 10 mins, flow rate: 1 mL/min); final isolated yield 16% (13.4 mg); *ee.* 95% (chiral HPLC, method 3, Supelco Astec Chirobiotic™ T column (25 cm × 4.6 mm), retention time: 76.9 min and 85.5 min, run time: 120 mins, flow rate: 1 mL/min). ^{1}H NMR (600 MHz; CD_3OD) δ = 6.33-6.30 (3H, m, 5-H, 3'-H and 7'-H), 4.92-4.90 (1H, m, 1-H), 3.51-3.47 (1H, m, 3-H<u>H</u>), 3.41 (1H, dd, *J* = 14.4 Hz, 4.8 Hz, 1'-H<u>H</u>), 3.29-3.26 (1H, m, and 3-<u>H</u>H), 3.17-3.14 (1H, m, 1'-<u>H</u>H), 3.00-2.92 (2H, m, 4-H<u>H</u>, 4-<u>H</u>H); ^{13}C NMR (150 MHz; CD_3OD) δ = 156.7 (d, $^{1}J_{CF}$ = 235.5 Hz), 151.7 (d, $^{1}J_{CF}$ = 211.5 Hz), 148.1 (d, $^{3}J_{CF}$ = 6.0 Hz), 147.0 (d, $^{3}J_{CF}$ = 7.5 Hz), 137.8 (d, $^{3}J_{CF}$ = 7.5 Hz), 132.0 (d, $^{3}J_{CF}$ = 7.5 Hz), 129.8 (d, $^{2}J_{CF}$ = 12.0 Hz), 128.4 (d, $^{2}J_{CF}$ = 15.0 Hz), 118.6 (d, $^{2}J_{CF}$ = 12.0 Hz), 112.7 (d, $^{4}J_{CF}$ = 3.0 Hz), 110.9 (d, $^{4}J_{CF}$ = 3.0 Hz), 108.4 (d, $^{2}J_{CF}$ = 12.0 Hz), 55.2, 43.6, 41.0, 27.7; *m/z* [ES+] 324 ($[M+H]^+$, 100%); *m/z* [HRMS ES+] found $[M+H]^+$ 324.1045. $[C_{16}H_{15}F_2NO_4+H]^+$ requires 324.1047; $[\alpha]_D^{25}$ 1.6 (c 0.10, MeOH).

*(S)-8-Fluoro-1-(3-fluoro-4-hydroxybenzyl)-1,2,3,4-tetrahydroisoquinoline-6,7-diol (S)-**54***

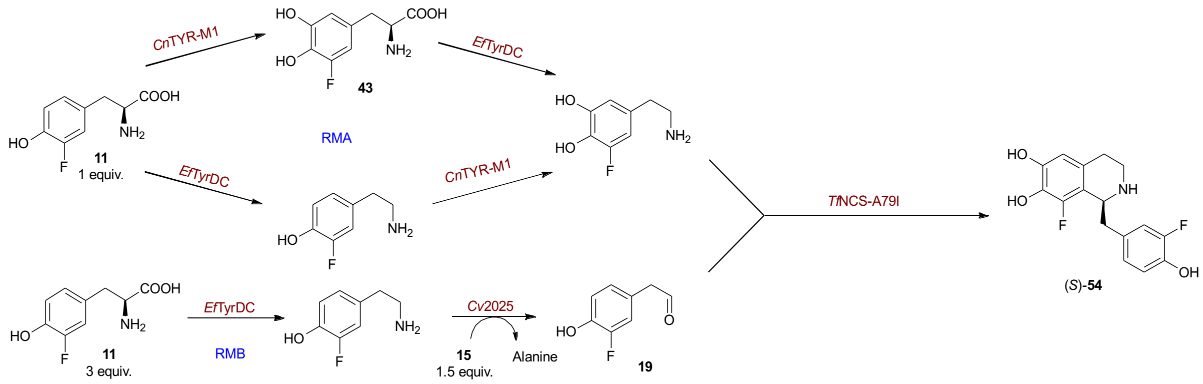

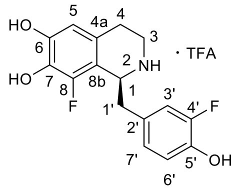

To synthesize the amine moiety, reaction mixture A (RMA, 20 mL, pH 5) consisted of 50 mM HEPES, 10 mM 3-F-L-tyrosine **11**, 40 mM sodium ascorbate **8**, 5 μM $CuSO_4·5H_2O$ and 5 mM PLP. To initiate the hydroxylation and decarboxylation steps, 10% (v/v) of *Cn*TYR-M1 (N201S/G205R/V206I) lysates and 10% (v/v) of *Ef*TyrDC lysates were added to RMA which

was incubated at 25 °C, 250 rpm for 8 h. For the aldehyde moiety, reaction mixture B (RMB, 20 mL, pH 7.5) consisted of 10% (v/v) of MeCN, 50 mM HEPES, 30 mM 3-F-L-tyrosine **11**, 30 mM sodium ascorbate **8**, 5 mM PLP and 30 mM sodium pyruvate **15**. To initiate the decarboxylation and transamination steps, 10% (v/v) of *Ef*TyrDC lysates and 5% (v/v) of *Cv*TAm lysates were added to RMB and the solution was incubated at 37 °C, 250 rpm for 8 h. Then RMA and RMB were mixed. The Pictet-Spengler condensation was performed with 10% (v/v) of *Tf*NCS-A79I lysates at 37 °C, 250 rpm for 16 h. In control reactions, all the enzymes were replaced by empty-vector cell lysates. The product was purified using preparative HPLC (method 4, Supelco™ Discovery BIO wide pore (C18, 10 µm, 2.12 cm x 25 cm) preparative column, retention time: 16.2 min, run time: 28 mins, flow rate: 8 mL/min)). Fractions containing the desired product were freeze-dried to give the product (*S*)-**54** as an orange powder (TFA salt, yield by HPLC (calibration curve) 43% (method 1, ACE 5 C18 column (150 × 4.6 mm), retention time: 5.2 min, run time: 10 mins, flow rate: 1 mL/min); final isolated yield 31% (25 mg), *ee.* 90% (chiral HPLC, method 2, Supelco Astec Chirobiotic™ T column (25 cm × 4.6 mm), retention time: 6.4 min and 8.7 min, run time: 40 mins, flow rate: 1 mL/min). ^{1}H NMR (600 MHz; CD_3OD) δ = 6.99-6.94 (3H, m, 3'-H, 6'-H, and 7'-H), 6.41 (1H, s, 5-H), 4.80 (1H, dd, *J* = 5.4 Hz, 2.4 Hz, 1-H), 3.50 (1H, dd, *J* = 6.6 Hz, 2.4 Hz, 3-H<u>H</u>), 3.26-3.16 (3H, m, 3-<u>H</u>H, 1'-H<u>H</u> and 1'-H<u>H</u>), 3.07-3.05 (2H, m, 4-H<u>H</u>, 4-<u>H</u>H); ^{13}C NMR (150 MHz; CD_3OD) δ = 155.6 (d, $^{1}J_{CF}$ = 240.0 Hz), 150.7 (d, $^{1}J_{CF}$ = 237.0 Hz), 147.0 (d, $^{3}J_{CF}$ = 4.5 Hz), 143.7 (d, $^{2}J_{CF}$ = 12.0 Hz), 134.8 (d, $^{3}J_{CF}$ = 6.0 Hz), 132.1 (d, $^{3}J_{CF}$ = 4.5 Hz), 128.3 (d, $^{2}J_{CF}$ = 12.0 Hz), 126.7 (d, $^{4}J_{CF}$ = 3.0 Hz), 118.8 (d, $^{2}J_{CF}$ = 15.0 Hz), 117.2 (d, $^{3}J_{CF}$ = 6.0 Hz), 116.2 (d, $^{2}J_{CF}$ = 10.5 Hz), 110.8 (d, $^{4}J_{CF}$ = 3.0 Hz), 55.2, 45.8, 44.2, 27.0; *m/z* [ES+] 308 ($[M+H]^+$, 100%); *m/z* [HRMS ES+] found $[M+H]^+$ 308.1101. $[C_{16}H_{15}F_2NO_3+H]^+$ requires 308.1098; $[\alpha]_D^{25}$ 11.9 (c 0.21, MeOH).

*(S)-1-(3-Chloro-4-hydroxybenzyl)-8-fluoro-1,2,3,4-tetrahydroisoquinoline-6,7-diol (S)-**55***

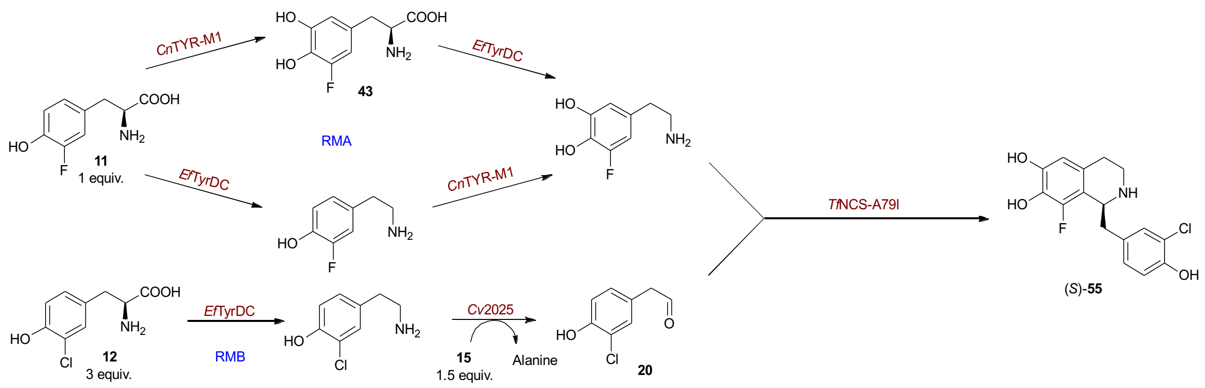

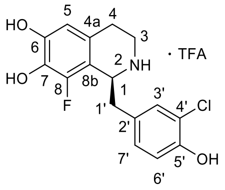

To synthesize the amine moiety, reaction mixture A (RMA, 20 mL, pH 5) consisted of 50 mM HEPES, 10 mM 3-F-L-tyrosine **11**, 40 mM sodium ascorbate **8**, 5 μM $CuSO_4 \cdot 5H_2O$ and 5 mM PLP. To initiate the hydroxylation and decarboxylation steps, 10% (v/v) of *Cn*TYR-M1 (N201S/G205R/V206I) lysates and 10% (v/v) of *Ef*TyrDC lysates were added to RMA which was incubated at 25 °C, 250 rpm for 8 h. For the aldehyde moiety, reaction mixture B (RMB, 20 mL, pH 7.5) consisted of 10% (v/v) of MeCN, 50 mM HEPES, 30 mM 3-Cl-L-tyrosine **12**, 30 mM sodium ascorbate **8**, 5 mM PLP and 30 mM sodium pyruvate **15**. To initiate the decarboxylation and transamination steps, 10% (v/v) of *Ef*TyrDC lysates and 5% (v/v) of *Cv*TAm lysates were added to RMB and the solution was incubated at 37 °C, 250 rpm for 8 h. Then RMA and RMB were mixed. The Pictet-Spengler condensation was performed with 10% (v/v) of *Tf*NCS-A79I lysates at 37 °C, 250 rpm for 16 h. In control reactions, all the enzymes were replaced by empty-vector cell lysates. The product was purified using preparative HPLC (method 4, Supelco™ Discovery BIO wide pore (C18, 10 μm, 2.12 cm x 25 cm) preparative column, retention time: 18.1 min, run time: 28 mins, flow rate: 8 mL/min)). Fractions containing the desired product were freeze-dried to give the product (*S*)-**55** as a yellow powder (yield by HPLC (calibration curve) 47% (method 1, ACE 5 C18 column (150 × 4.6 mm), retention time: 5.5 min, run time: 10 mins, flow rate: 1 mL/min); final isolated yield 33% (27.7 mg); *ee.* 95% (chiral HPLC, method 2, Supelco Astec Chirobiotic™ T column (25 cm × 4.6 mm), retention time: 9.6 min and 11.6 min, run time: 40 mins, flow rate:

1 mL/min). ^{1}H NMR (700 MHz; CD_3OD) δ = 7.25 (1H, d, J = 1.8 Hz, 3'-H), 7.04 (1H, dd, J = 8.4 Hz, 1.8 Hz, 7'-H), 6.90 (1H, d, J = 8.4 Hz, 6'-H), 6.49 (1H, s, 5-H), 4.83 (1H, dd, J = 9.1 Hz, 4.9 Hz, 1-H), 3.50-3.46 (1H, m, 3-HH), 3.28-3.26 (2H, m, 3-HH and 1'-HH), 3.04 (1H, dd, J = 15.4 Hz, 9.1 Hz, 1'-HH), 2.98-2.91 (3H, m, 1'-HH, 4-HH, 4-HH); ^{13}C (175 MHz; CD_3OD) δ = 154.0, 150.0 ($^1J_{CF}$ = 238.3 Hz), 149.0 ($^3J_{CF}$ = 7.0 Hz), 133.7 ($^2J_{CF}$ = 14.0 Hz), 131.8, 129.9, 128.3, 123.1 ($^3J_{CF}$ = 3.5 Hz), 122.0, 118.1, 111.8 ($^2J_{CF}$ = 12.3 Hz), 111.7 ($^4J_{CF}$ = 1.8 Hz), 58.2, 38.8, 38.6, 25.0; *m/z* [ES+] 324 ($[M+H]^+$, 100%), *m/z* [HRMS ES+] found $[M+H]^+$ 324.0796; $[C_{16}H_{15}{}^{35}ClFNO_3+H]^+$ requires 324.0797; $[\alpha]_D^{26}$ 7.4 (c 0.24, MeOH).

*(S)-1-(3-Iodo-4-hydroxybenzyl)-8-fluoro-1,2,3,4-tetrahydroisoquinoline-6,7-diol (S)-**56***

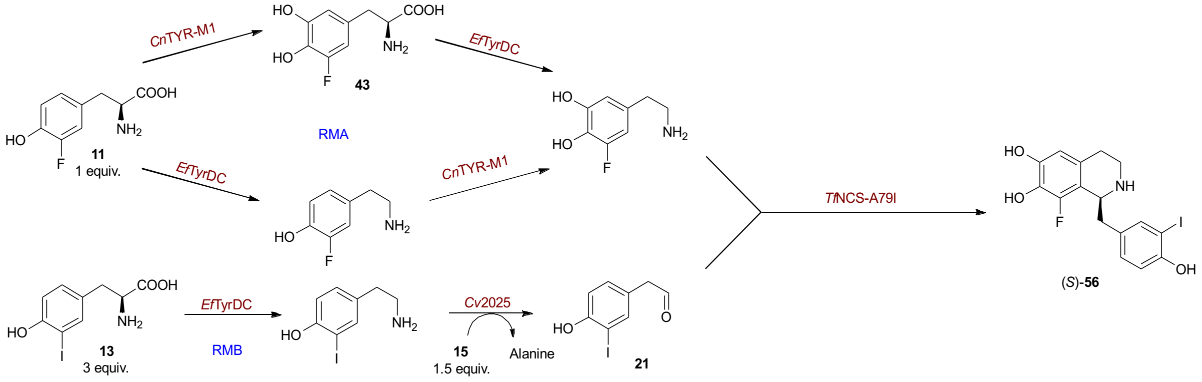

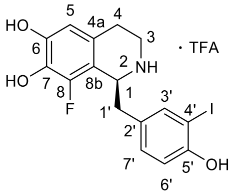

To synthesize the amine moiety, reaction mixture A (RMA, 20 mL, pH 5) consisted of 50 mM HEPES, 10 mM 3-F-L-tyrosine **11**, 40 mM sodium ascorbate **8**, 5 μM $CuSO_4{\cdot}5H_2O$ and 5 mM PLP. To initiate the hydroxylation and decarboxylation steps, 10% (v/v) of *Cn*TYR-M1 (N201S/G205R/V206I) lysates and 10% (v/v) of *Ef*TyrDC lysates were added to RMA which was incubated at 25 °C, 250 rpm for 8 h. For the aldehyde moiety, reaction mixture B (RMB, 20 mL, pH 7.5) consisted of 10% (v/v) of MeCN, 50 mM HEPES, 30 mM 3-I-L-tyrosine **13**, 30 mM sodium ascorbate **8**, 5 mM PLP and 30 mM sodium pyruvate **15**. To initiate the decarboxylation and transamination steps, 10% (v/v) of *Ef*TyrDC lysates and 5% (v/v) of *Cv*TAm lysates were added to RMB and the solution was incubated at 37 °C, 250 rpm for 8 h. Then RMA and RMB were mixed. The Pictet-Spengler condensation was performed

with 10% (v/v) of *Tf*NCS-A79I lysates at 37 °C, 250 rpm for 16 h. In control reactions, all the enzymes were replaced by empty-vector cell lysates. The product was purified using preparative HPLC (method 4, Supelco™ Discovery BIO wide pore (C18, 10 µm, 2.12 cm x 25 cm) preparative column, retention time: 21.1 min, run time: 28 mins, flow rate: 8 mL/min)). Fractions containing the desired product were freeze-dried to give the product (*S*)-**56** as a yellow powder (TFA salt, yield by HPLC (calibration curve) 42% (method 1, ACE 5 C18 column (150 × 4.6 mm), retention time: 5.9 min, run time: 10 mins, flow rate: 1 mL/min); final isolated yield 28% (28.6 mg); *ee.* 95% (chiral HPLC, method 2, Supelco Astec Chirobiotic™ T column (25 cm × 4.6 mm), retention time: 13.7 min and 18.1 min, run time: 40 mins, flow rate: 1 mL/min). ^{1}H NMR (700 MHz; CD_3OD) δ = 7.63 (1H, s, 3'-H), 7.10 (1H, dd, J = 7.7 Hz, 1.4 Hz, 7'-H), 6.82 (1H, d, J = 8.4 Hz, 6'-H), 6.50 (1H, s, 5-H), 4.82 (1H, dd, J = 9.1 Hz, 4.2 Hz, 1-H), 3.49-3.45 (1H, m, 3-HH), 3.29-3.25 (2H, m, 3-HH and 1'-HH), 3.02 (1H, dd, J = 14.7 Hz, 9.1 Hz, 1'-HH), 2.98-2.90 (3H, m, 1'-HH, 4-HH, 4-HH); ^{13}C (175 MHz; CD_3OD) δ = 157.7, 150.0 ($^{1}J_{CF}$ = 238.0 Hz), 148.9 ($^{3}J_{CF}$ = 7.0 Hz), 141.1, 133.7 ($^{2}J_{CF}$ = 14.0 Hz), 131.4, 128.9, 123.1 ($^{3}J_{CF}$ = 3.5 Hz), 116.1, 111.8 ($^{2}J_{CF}$ = 12.3 Hz), 117.0 ($^{4}J_{CF}$ = 1.8 Hz), 85.2, 53.2, 38.8, 38.3, 25.0; *m/z* [ES+] 416 ($[M+H]^+$, 100%), *m/z* [HRMS ES+] found $[M+H]^+$ 416.0151; $[C_{16}H_{15}IFNO_3+H]^+$ requires 416.0153, $[\alpha]_D^{26}$ 12.7 (c 0.12, MeOH).

*(S)-8-Fluoro-1-(4-hydroxybenzyl)-1,2,3,4-tetrahydroisoquinoline-6,7-diol (S)-**57***

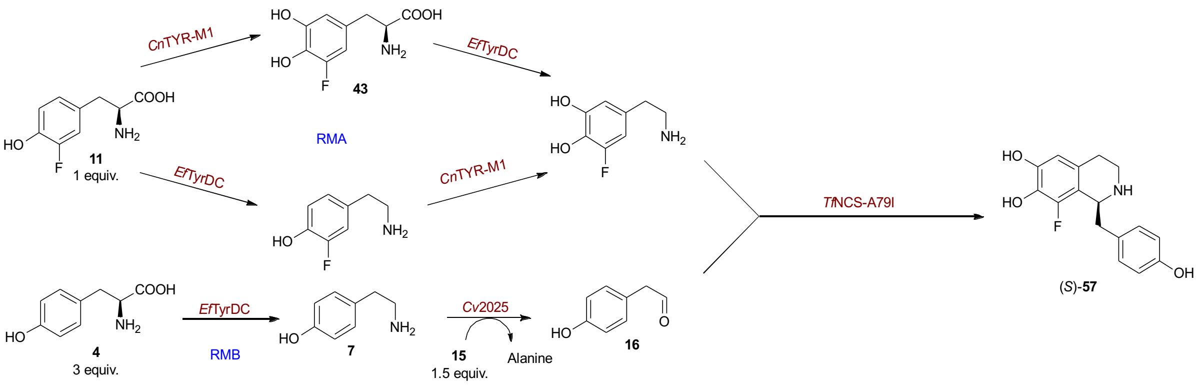

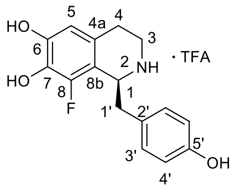

To synthesize the amine moiety, reaction mixture A (RMA, 20 mL, pH 5) consisted of 50

mM HEPES, 10 mM 3-F-L-tyrosine **11**, 40 mM sodium ascorbate **8**, 5 µM $CuSO_4 \cdot 5H_2O$ and 5 mM PLP. To initiate the hydroxylation and decarboxylation steps, 10% (v/v) of *Cn*TYR-M1 (N201S/G205R/V206I) lysates and 10% (v/v) of *Ef*TyrDC lysates were added to RMA which was incubated at 25 °C, 250 rpm for 8 h. For the aldehyde moiety, reaction mixture B (RMB, 240 mL, pH 7.5) consisted of 10% (v/v) of MeCN, 50 mM HEPES, 2.5 mM L-tyrosine **4**, 2.5 mM sodium ascorbate **8**, 1.25 mM PLP and 2.5 mM sodium pyruvate **15**. To initiate the decarboxylation and transamination steps, 10% (v/v) of *Ef*TyrDC lysates and 5% (v/v) of *Cv*TAm lysates were added to RMB and the solution was incubated at 37 °C, 250 rpm for 8 h. Then RMA and RMB were mixed. The Pictet-Spengler condensation was performed with 10% (v/v) of *Tf*NCS-A79I lysates at 37 °C, 250 rpm for 16 h. In control reactions, all the enzymes were replaced by empty-vector cell lysates. The product was purified using preparative HPLC (method 4, Supelco™ Discovery BIO wide pore (C18, 10 µm, 2.12 cm x 25 cm) preparative column, retention time: 14.8 min, run time: 28 mins, flow rate: 8 mL/min)). Fractions containing the desired product were freeze-dried to give the product (*S*)-**57** as a yellow powder (TFA salt, yield by HPLC (calibration curve) 78% (method 1, ACE 5 C18 column (150 × 4.6 mm), retention time: 5.1 min, run time: 10 mins, flow rate: 1 mL/min); final isolated yield 52% (40.2 mg); *ee.* 95% (chiral HPLC, method 2, Supelco Astec Chirobiotic™ T column (25 cm × 4.6 mm), retention time: 8.6 min and 11.2 min, run time: 40 mins, flow rate: 1 mL/min). ^{1}H NMR (700 MHz; CD_3OD) δ = 7.10 (2H, br. d, *J* = 8.7 Hz, 3'-H and 7'-H), 6.78 (2H, br. d, *J* = 8.7 Hz, 4'-H and 6'-H), 6.49 (1H, s, 5-H), 4.80 (1H, dd, *J* = 9.5 Hz, 4.2 Hz, 1-H), 3.48-3.44 (1H, m, 3-H<u>H</u>), 3.34-3.31 (1H, m, 1'-H<u>H</u>), 3.27-3.24 (1H, m, 3-<u>H</u>H), 3.03 (1H, dd, *J* = 14.7 Hz, 9.5 Hz, 1'-<u>H</u>H), 2.95-2.90 (2H, m, 4-H<u>H</u> and 4-<u>H</u>H); ^{13}C (175 MHz; CD_3OD) δ = 156.7, 150.1 ($^1J_{CF}$ = 236.3 Hz), 148.9 ($^3J_{CF}$ = 7.0 Hz), 133.7 ($^2J_{CF}$ = 14.0 Hz), 130.8, 126.7, 123.1 ($^3J_{CF}$ = 5.3 Hz), 116.0, 112.1 ($^2J_{CF}$ = 12.3 Hz), 111.7 ($^4J_{CF}$ = 1.8 Hz), 64.5, 39.3, 38.9, 30.6; *m/z* [ES+] 290 ($[M+H]^+$, 100%), *m/z* [HRMS ES+] found $[M+H]^+$ 290.1181; $[C_{16}H_{16}FNO_3+H]^+$ requires 290.1188, $[\alpha]_D^{26}$ -7.2 (c 0.21, MeOH).

2 Protein expression of enzymes used in the study

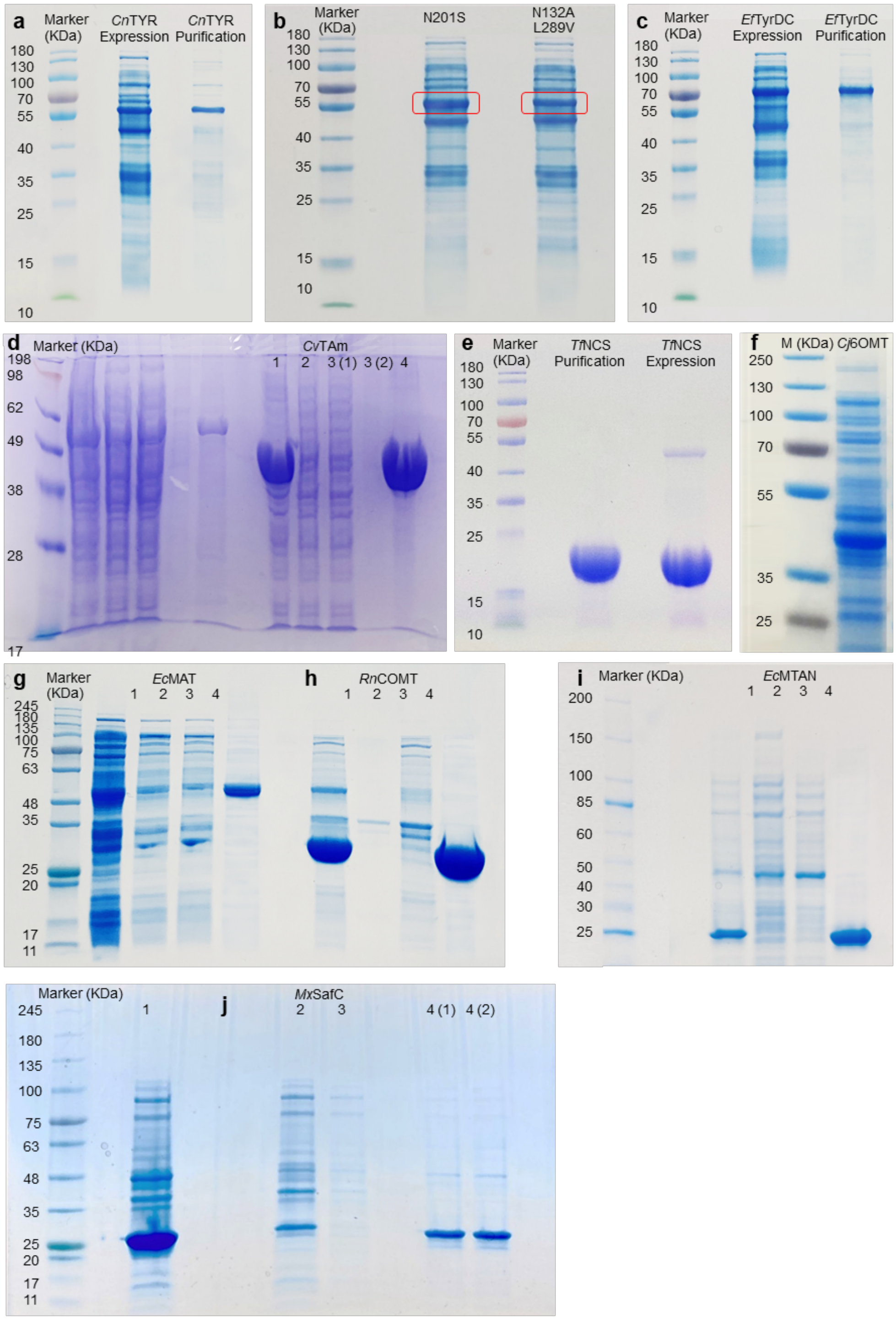

Supplementary Fig. 2 Images of SDS-PAGE electrophoresis for the protein expression of proteins used in the study. For enzyme purification: Lane 1: cell lysates; Lane 2: flow through with cell lysates; Lane 3: Flow through with wash buffer (containing 20 mM imidazole); Lane 4: Purified protein. Protein markers for **a**, **b**, **c** and **e** were from Fisher BioReagents™ EZ-Run™ Prestained Rec Protein Ladder, Fisher BioReagents, UK; The protein marker for **d** was from SeeBlue™ Plus2 Pre-stained Protein Standard, Invitrogen™, ThermoFisher Scientific, UK; The protein markerfor **f** was from Thermo Scientific™ PageRuler™ Plus Prestained Protein Ladder, ThermoFisher Scientific, UK; Protein markers for **g**, **h** and **j** were from BLUEstain™ Protein ladder, DoldBio, UK; The protein marker for **I** was from Unstained Protein Ladder, Broad Range, New England Biolabs, UK. One representative image from n=3 is shown here.

3 Kinetic studies for *Cn*TYR and *Cn*TYR-N201S

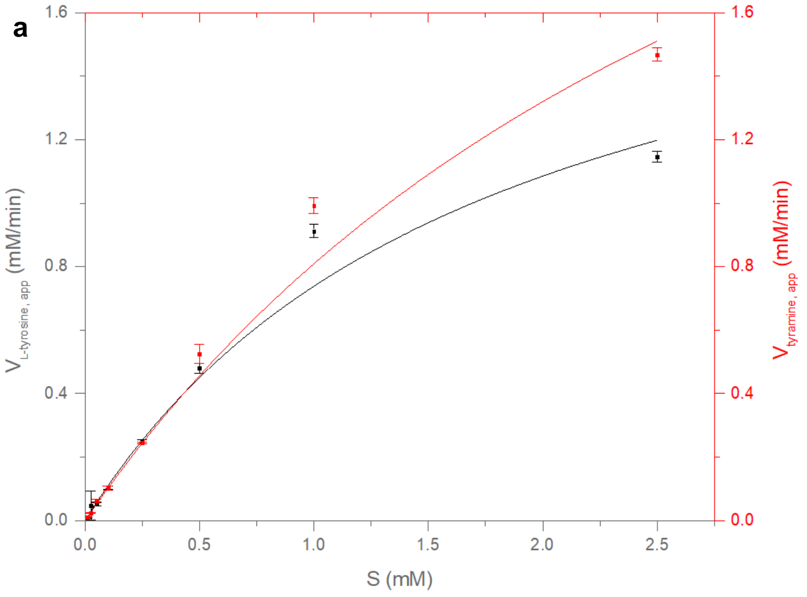

Model	Michealis-Menten Fit	
Plot	$V_{L\text{-tyrosine, app.}}$	$V_{tyramine, app.}$
$V_{max, app.}$ (mM/min)	2.05	3.58
$K_{m, app.}$ (mM)	1.78	3.43
$K_{cat, app.}$ (S^{-1})	31.6	55.2
R^2 (COD)	0.99	0.99

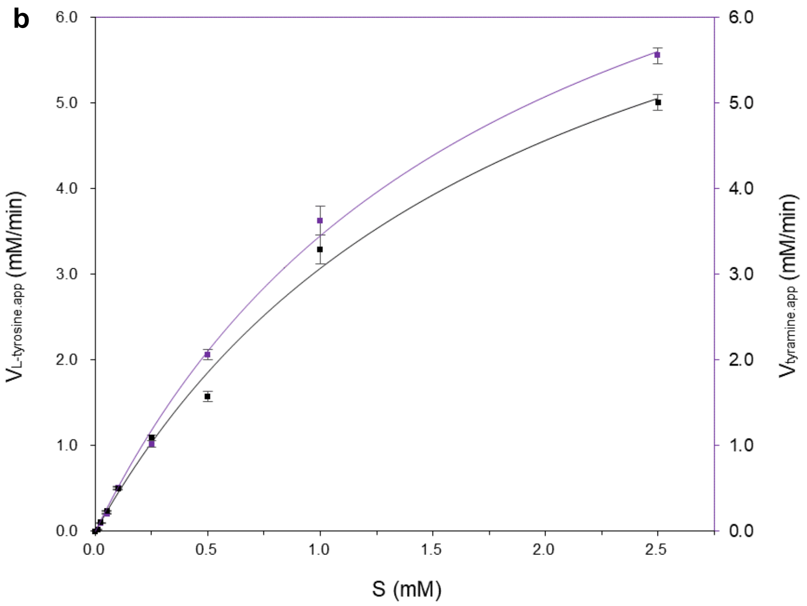

Model	Michealis-Menten Fit	
Plot	$V_{L\text{-tyrosine, app.}}$	$V_{tyramine, app.}$
$V_{max, app.}$ (mM/min)	8.87	9.58
$K_{m, app.}$ (mM)	1.89	1.78
$K_{cat, app.}$ (S^{-1})	182.89	197.53
R^2 (COD)	0.99	0.99

Supplementary Fig. 3 Fitting of the Michaelis-Menten Function for the wildtype *Cn*TYR and the variant *Cn*TYR-N201S using Origin Software. a Fitting for the wildtype *Cn*TYR. **b** Fitting for the variant *Cn*TYR-N201S. The apparent $K_{m.app}$ and the $K_{ca.appt}$ were calculated by Origin Software. Curves in grey and black represent the Michaelis-Menten fit towards L-tyrosine **4**. Curves in red and purple represent the Michaelis-Menten fit towards tyramine **7**. Experiments were repeated three times, and measurements were taken from distinct samples.

4 Achiral analytical HPLC results for *Cn*TYR and *Ef*TyrDC reaction products

Achiral separation was achieved using **Analytical HPLC method 1**.

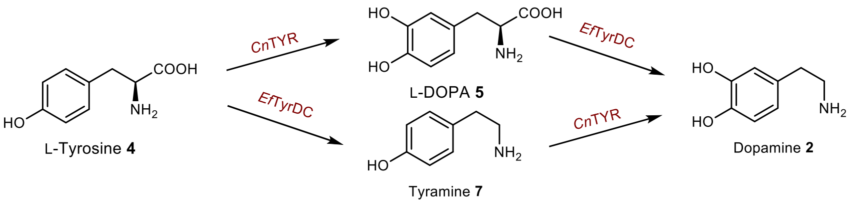

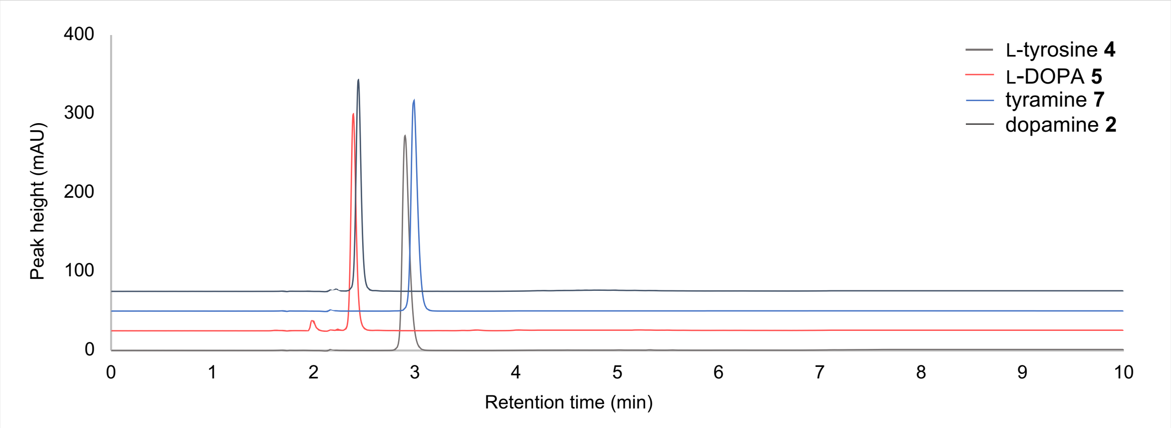

Supplementary Fig. 4 Analytical HPLC traces for the *Cn*TYR-*Ef*TyrDC reaction with L-tyrosine 4. Grey represents HPLC trace of L-tyrosine **4**. Pink represents HPLC trace of L-DOPA **2**. Blue represents HPLC trace of tyramine **7**. Black represents HPLC trace of dopamine **2**.

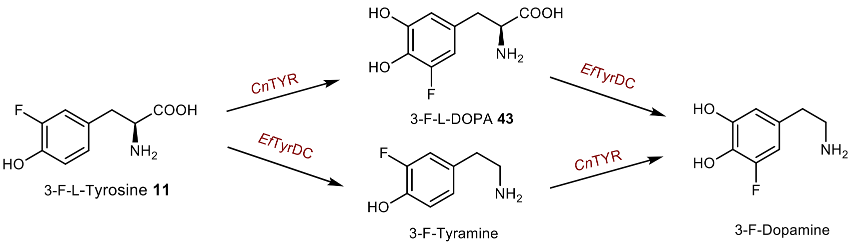

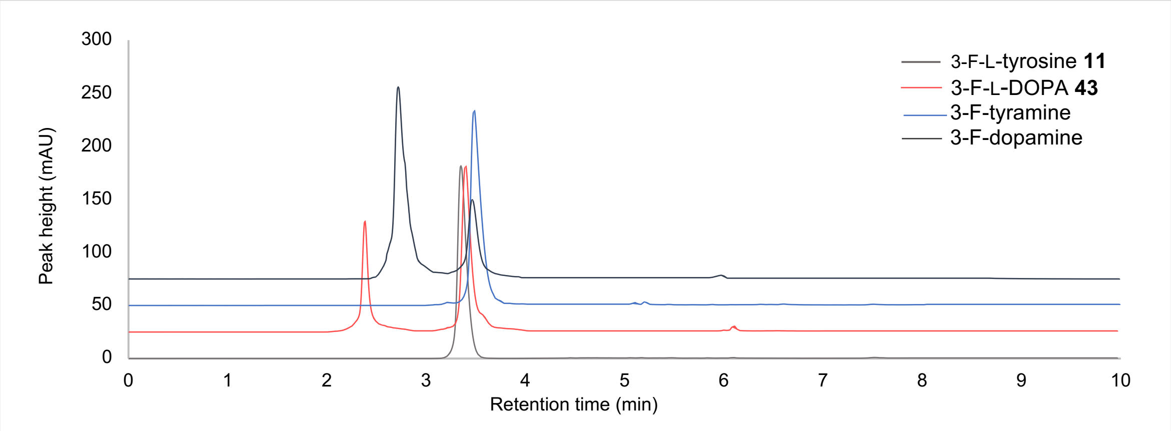

Supplementary Fig. 5 Analytical HPLC result for the *Cn*TYR-*Ef*TyrDC reaction with 3-F-L-tyrosine 11. Grey represents HPLC trace of 3-F-L-tyrosine **11**. Pink represents HPLC trace of 3-F-L-DOPA **43**. Blue represents HPLC trace of 3-F-tyramine. Black represents HPLC trace of 3-F-dopamine.

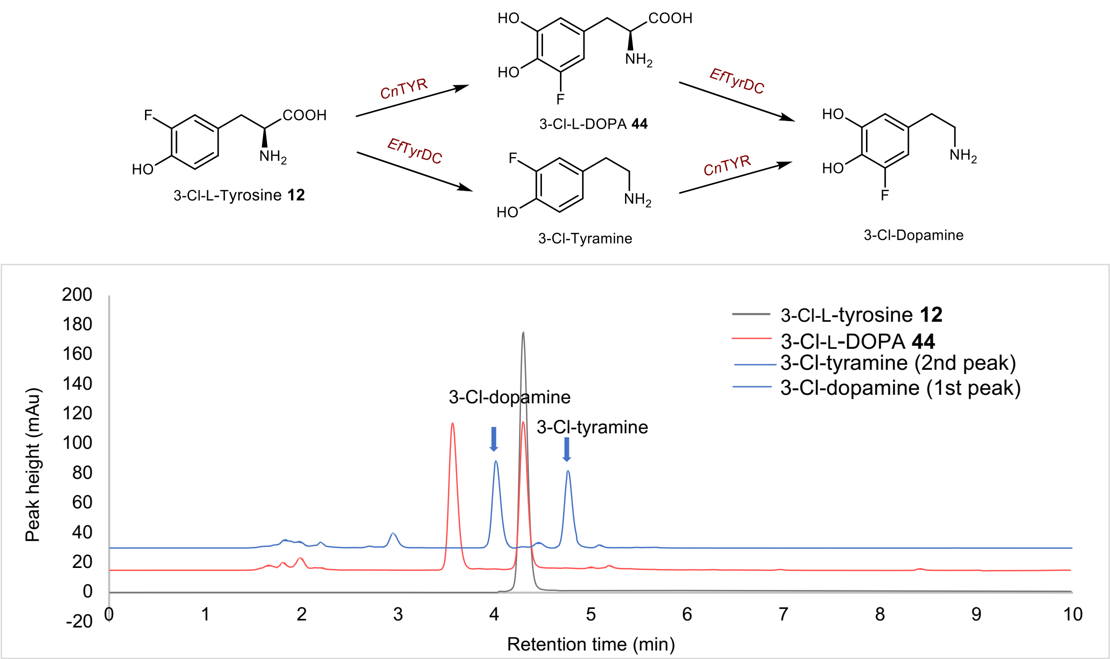

Supplementary Fig. 6 Analytical HPLC result for the *Cn*TYR-M8 (N201S/H202N)-*Ef*TyrDC reaction with 3-Cl-L-tyrosine 12. Grey represents HPLC trace of 3-Cl-L-tyrosine **12**. Pink represents HPLC trace of 3-Cl-L-DOPA **44** (the first peak) and **12** (the second peak). Blue represents HPLC trace of 3-Cl-dopamine (the first peak) and 3-Cl-tyramine (the second peak).

5 Achiral analytical HPLC results for Pictet-Spengler reaction products

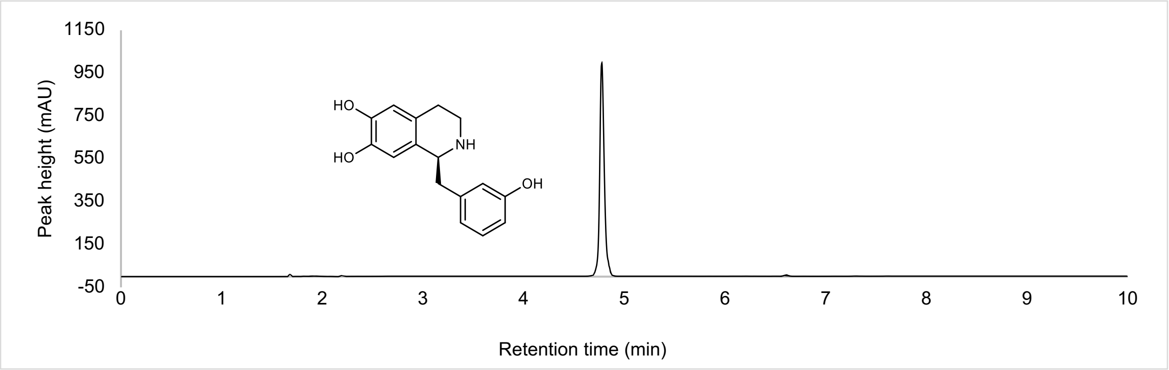

Supplementary Fig. 7 Analytical HPLC result for (*S*)-1-(3-hydroxybenzyl)-1,2,3,4-tetrahydroisoquinoline-6,7-diol (*S*)-**23**.

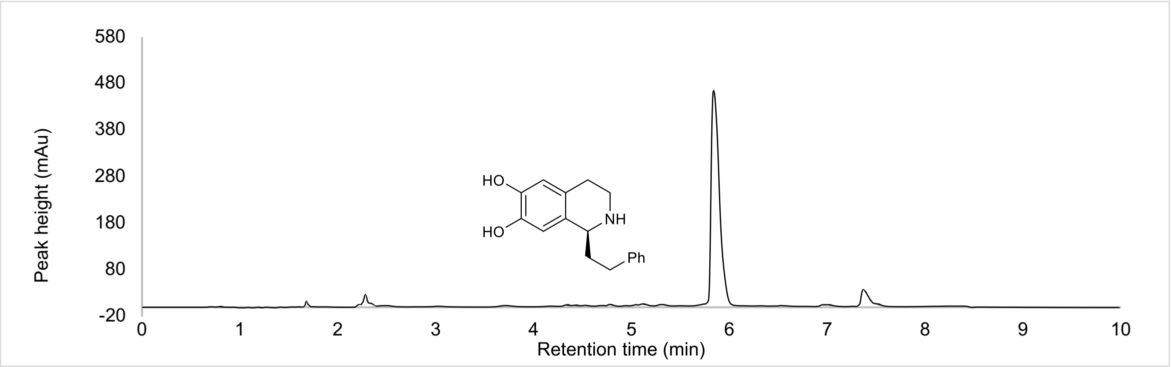

Supplementary Fig. 8 Analytical HPLC result for (*S*)-1-phenethyl-1,2,3,4-tetrahydroisoquinoline-6,7-diol (*S*)-**24**[1].

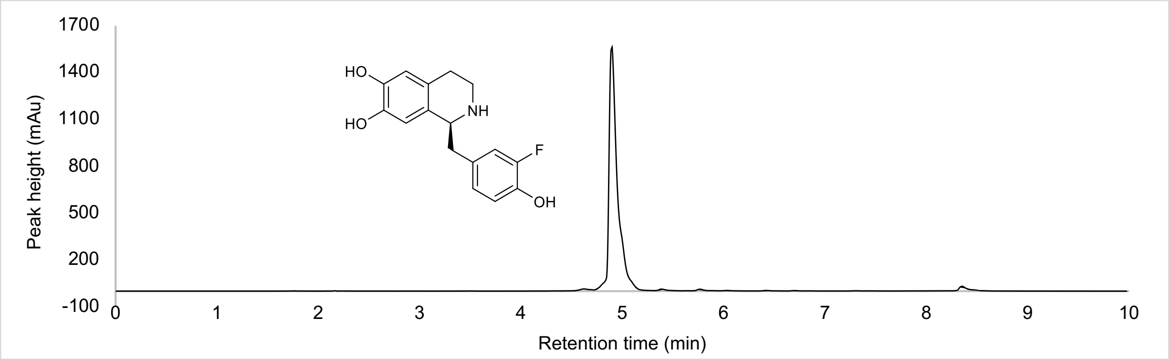

Supplementary Fig. 9 Analytical HPLC result for (*S*)-1-(3-fluoro-4-hydroxybenzyl)-1,2,3,4-tetrahydroisoquinoline-6,7-diol (*S*)-**25**.

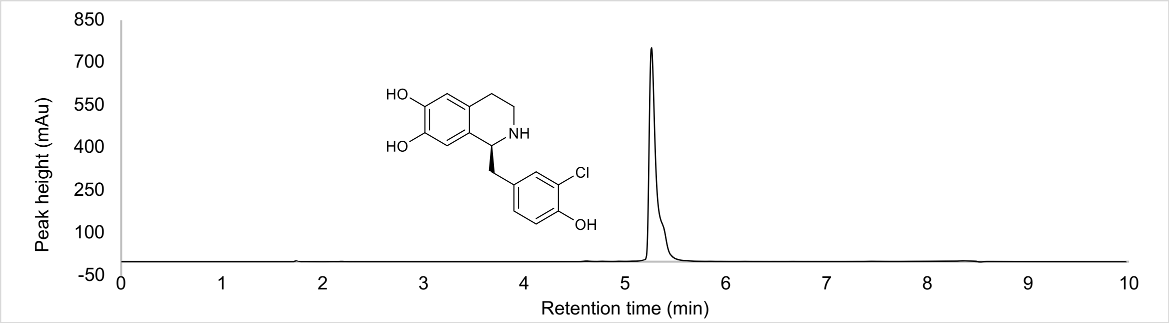

Supplementary Fig. 10 Analytical HPLC result for (*S*)-1-(3-chloro-4-hydroxybenzyl)-1,2,3,4-tetrahydroisoquinoline-6,7-diol (*S*)-**26**[2].

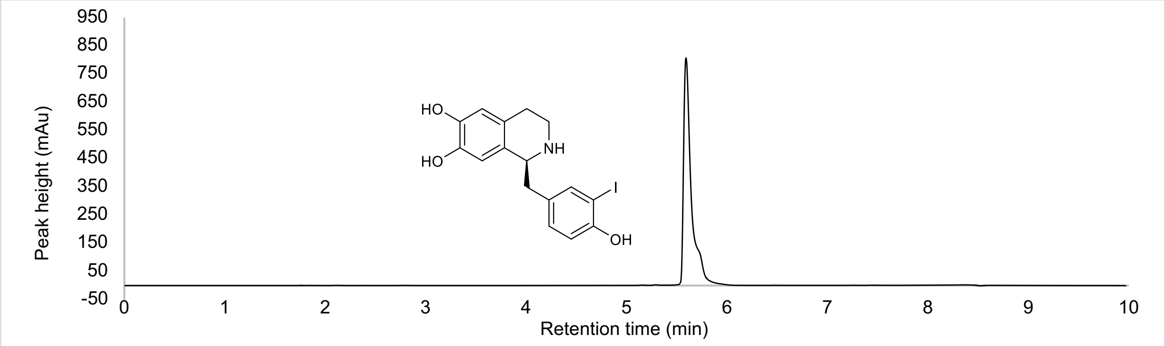

Supplementary Fig. 11 Analytical HPLC result for (*S*)-1-(3-iodo-4-hydroxybenzyl)-1,2,3,4-tetrahydroisoquinoline-6,7-diol (*S*)-**27**[2].

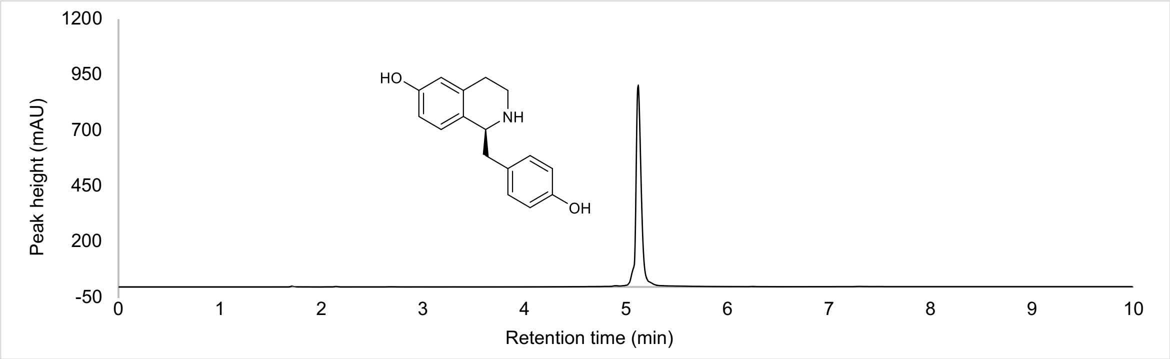

Supplementary Fig. 12 Analytical HPLC result for (*S*)-1-(4-hydroxybenzyl)-1,2,3,4-tetrahydroisoquinolin-6-ol (*S*)-**28**[3].

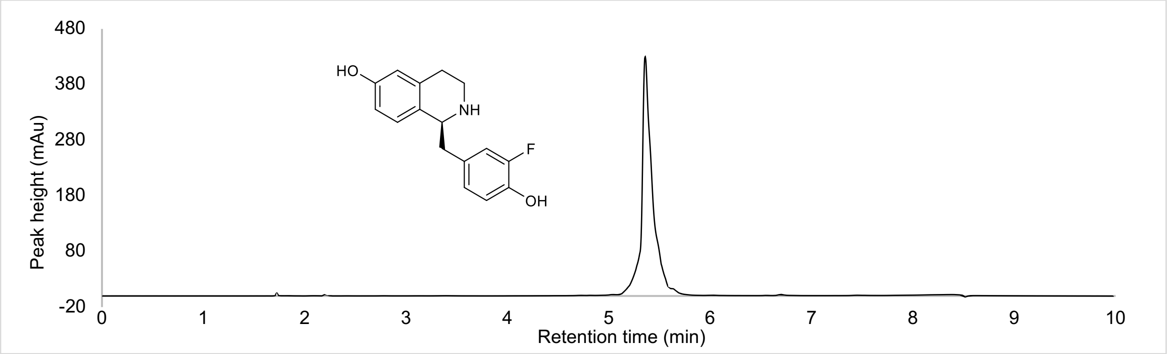

Supplementary Fig. 13 Analytical HPLC result for (*S*)-1-(3-fluoro-4-hydroxybenzyl)-1,2,3,4-tetrahydroisoquinolin-6-ol (*S*)-**29**.

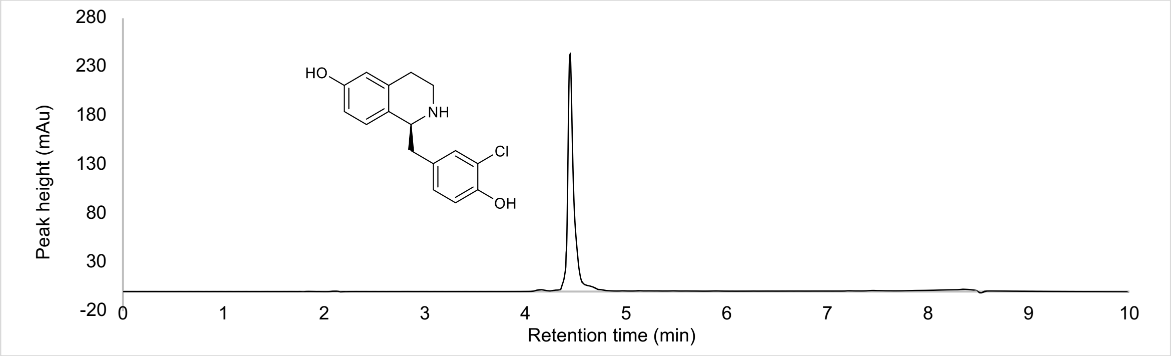

Supplementary Fig. 14 Analytical HPLC result for (*S*)-1-(3-chloro-4-hydroxybenzyl)-1,2,3,4-tetrahydroisoquinolin-6-ol (*S*)-**30**.

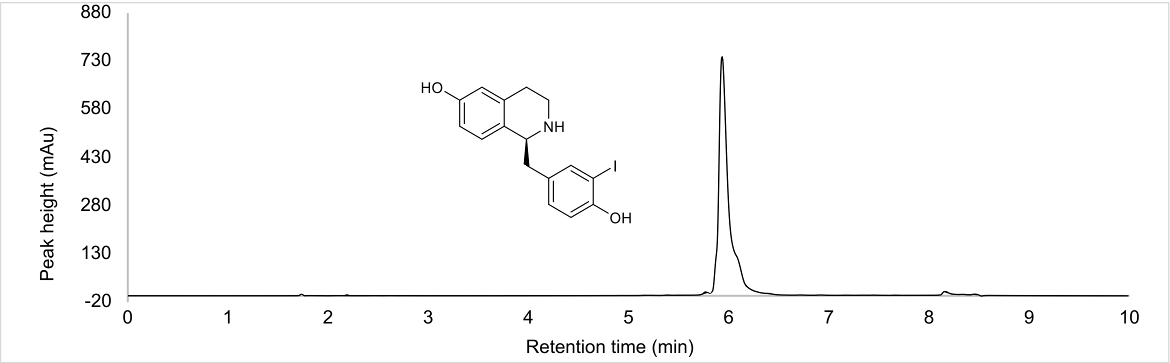

Supplementary Fig. 15 Analytical HPLC result for (*S*)-1-(3-Iodo-4-hydroxybenzyl)-1,2,3,4-tetrahydroisoquinolin-6-ol (*S*)-**31**.

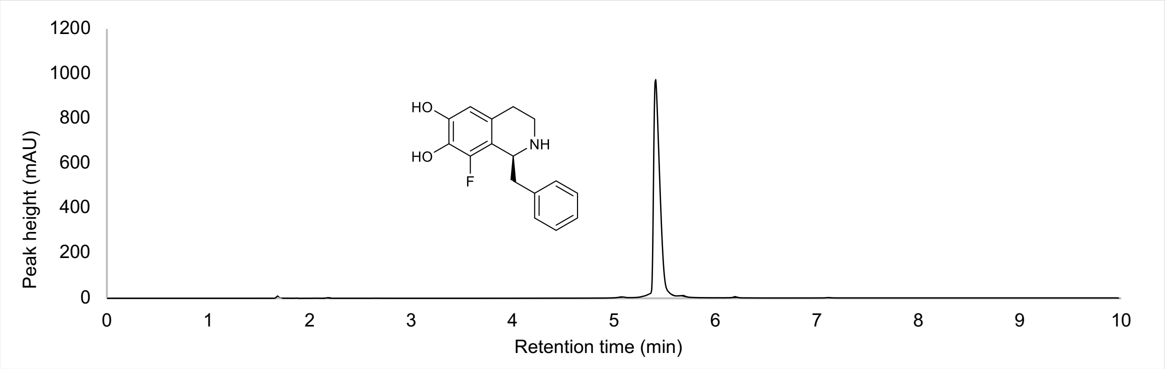

Supplementary Fig. 16 Analytical HPLC result for (*S*)-1-benzyl-8-fluoro-1,2,3,4-tetrahydroisoquinoline-6,7-diol (*S*)-**45**[4].

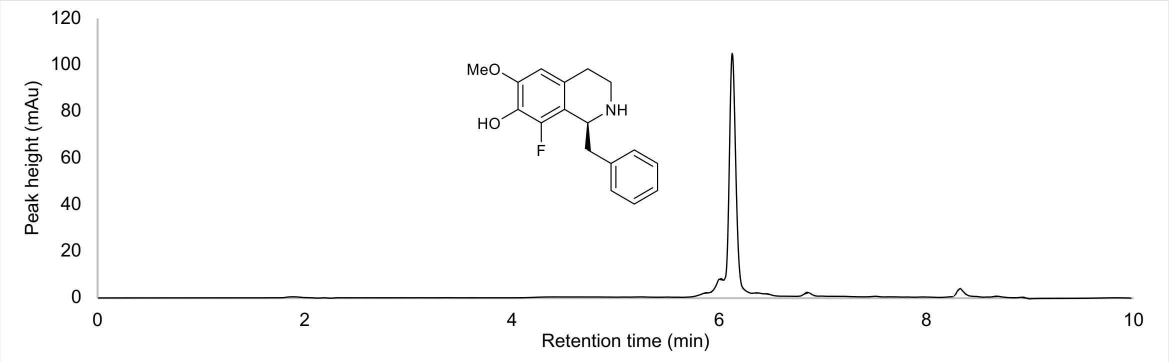

Supplementary Fig. 17 Analytical HPLC result for (*S*)-1-benzyl-8-fluoro-6-methoxy-1,2,3,4-tetrahydroisoquinolin -7-ol (*S*)-**46**[5].

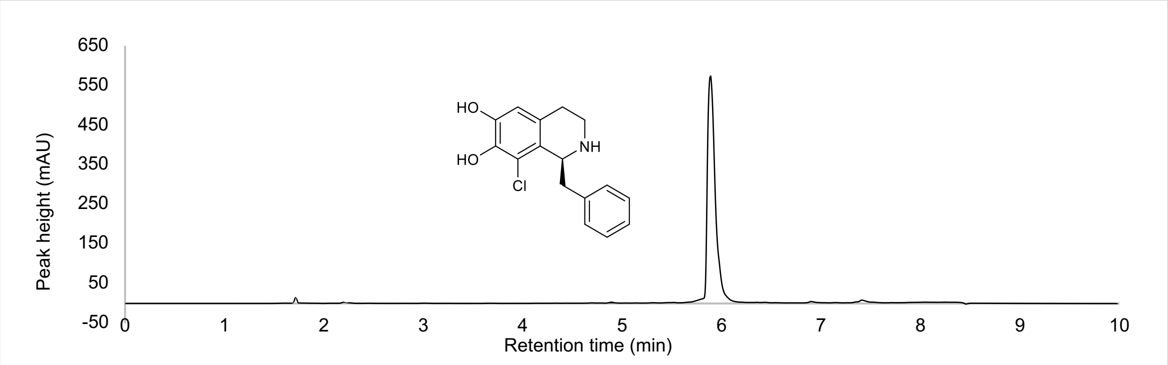

Supplementary Fig. 18 Analytical HPLC result for (*S*)-1-benzyl-8-chloro-1,2,3,4-tetrahydroisoquinoline-6,7-diol (*S*)-**48**.

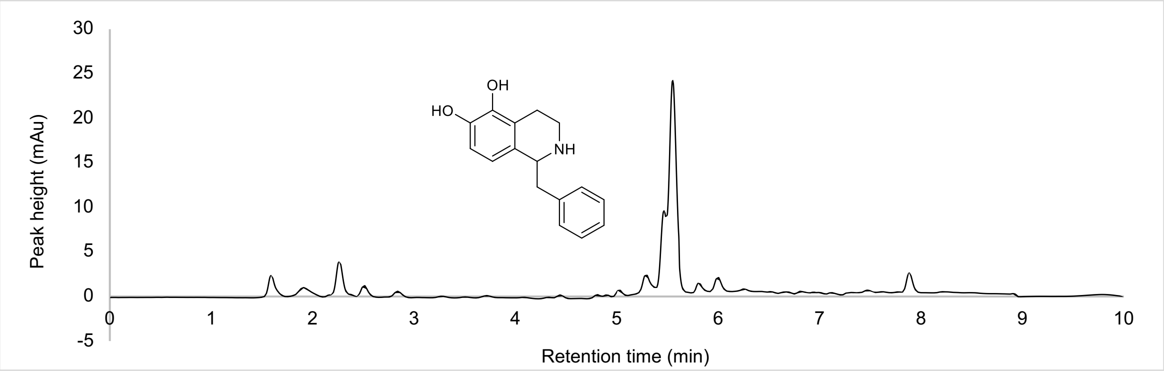

Supplementary Fig. 19 Analytical HPLC result for 1-benzyl-1,2,3,4-tetrahydroisoquinoline-5,6-diol (*S*)-**49**.

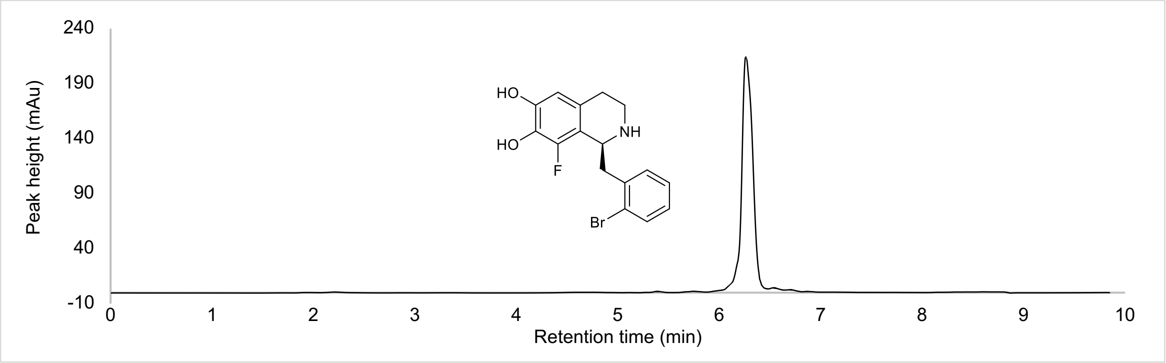

Supplementary Fig. 20 Analytical HPLC result for (*S*)-1-(2-bromobenzyl)-8-fluoro-1,2,3,4-tetrahydroisoquinoline-6,7-diol (*S*)-**51**.

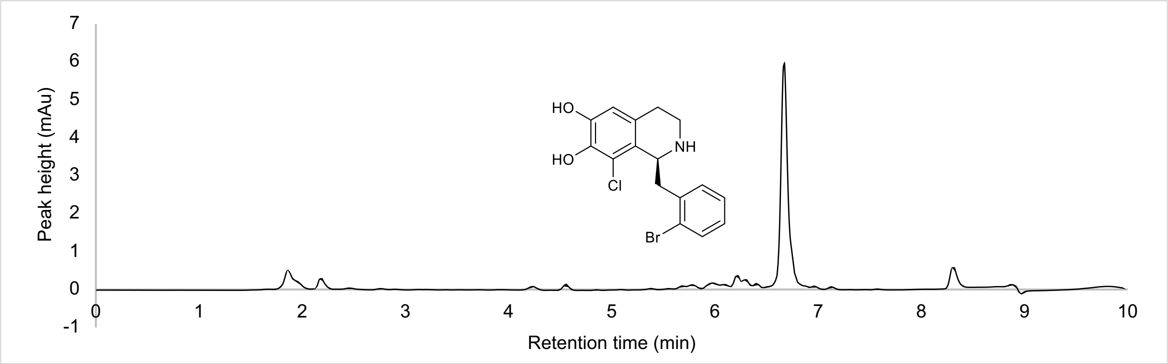

Supplementary Fig. 21 Analytical HPLC result for (S)-1-(2-bromobenzyl)-8-chloro-1,2,3,4-tetrahydroisoquinoline-6,7-diol (*S*)-**52**.

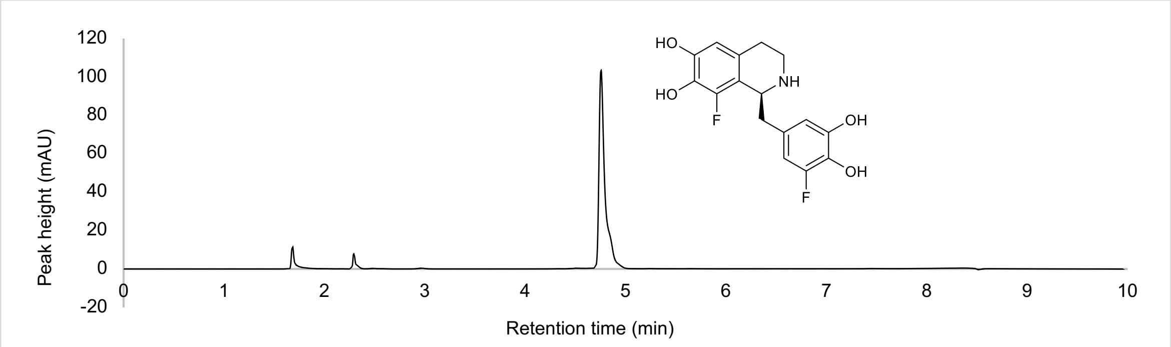

Supplementary Fig. 22 Analytical HPLC result for (*S*)-8-fluoro-1-(3-fluoro-4,5-dihydroxybenzyl)-1,2,3,4-tetrahydro-isoquinoline-6,7-diol (*S*)-**53**.

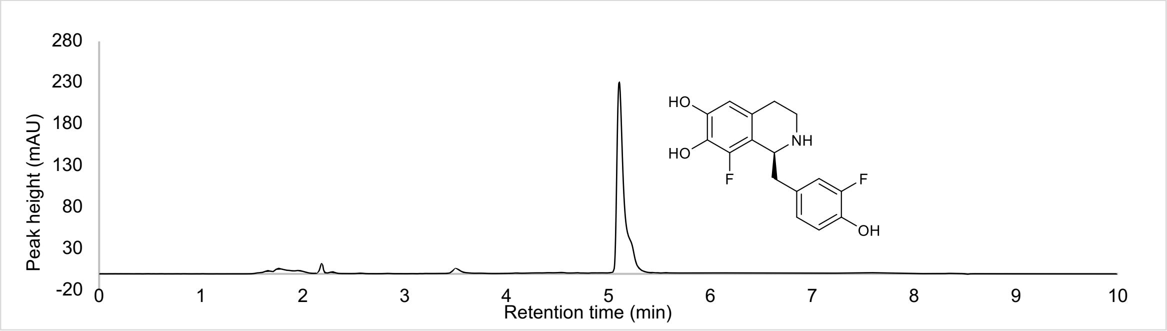

Supplementary Fig. 23 Analytical HPLC result for (*S*)-8-fluoro-1-(3-fluoro-4-hydroxybenzyl)-1,2,3,4-tetrahydro-isoquinoline-6,7-diol (*S*)-**54**.

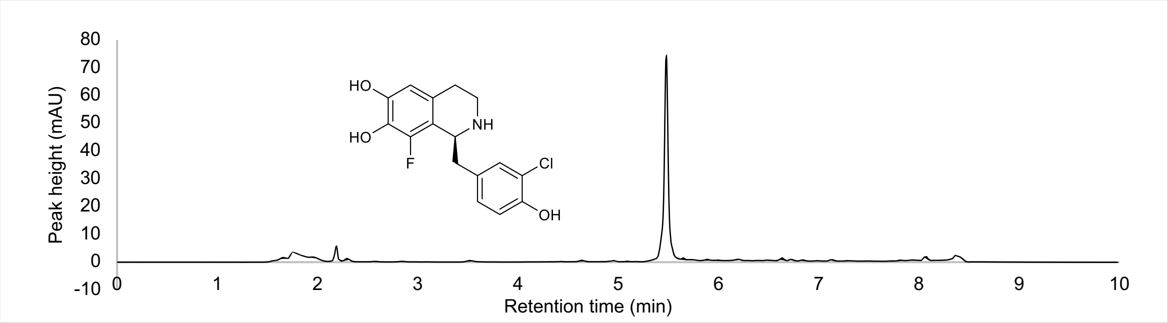

Supplementary Fig. 24 Analytical HPLC result for (*S*)-1-(3-chloro-4-hydroxybenzyl)-8-fluoro-1,2,3,4-tetrahydro-isoquinoline-6,7-diol (*S*)-**55**.

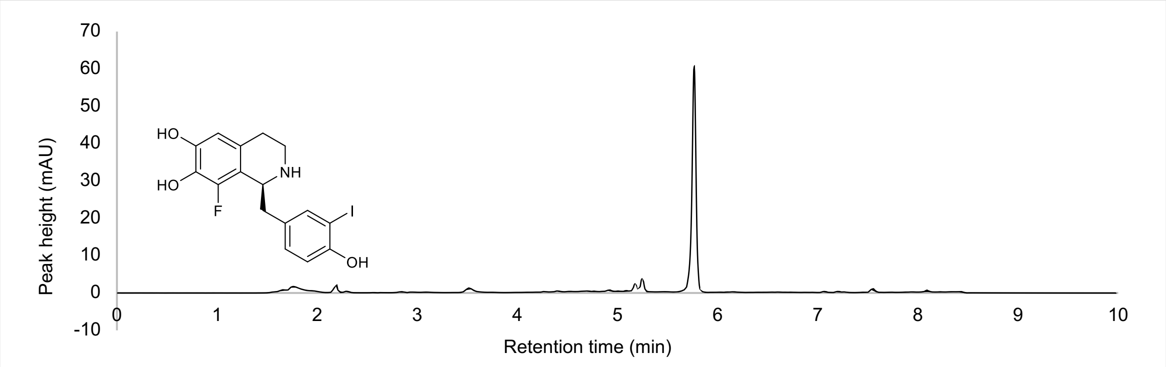

Supplementary Fig. 25 Analytical HPLC result for (*S*)-8-fluoro-1-(4-hydroxy-3-iodobenzyl)-1,2,3,4-tetrahydro-isoquinoline-6,7-diol (*S*)-**56**.

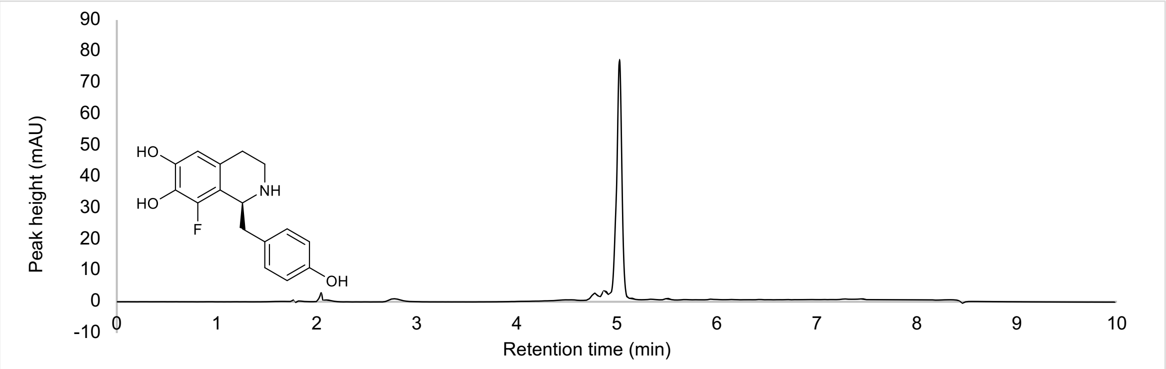

Supplementary Fig. 26 Analytical HPLC result for (*S*)-8-Fluoro-1-(4-hydroxybenzyl)-1,2,3,4-tetrahydroisoquinoline-6,7-diol (*S*)-**57**.

6 Calibration curves for analytical HPLC results of products

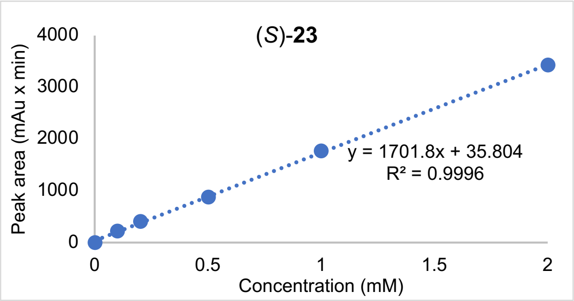

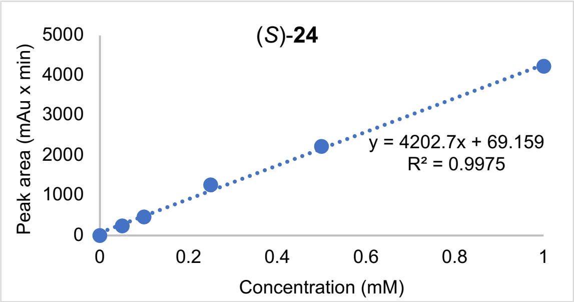

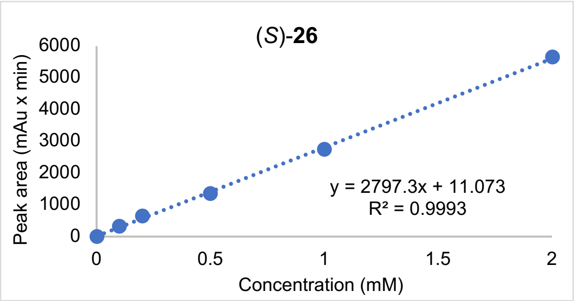

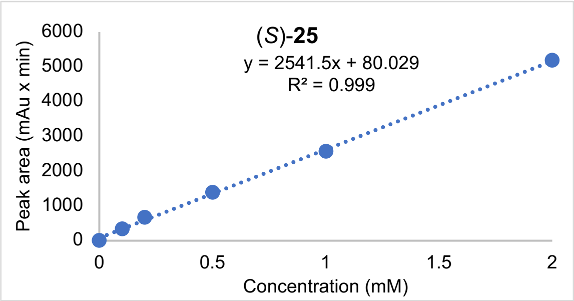

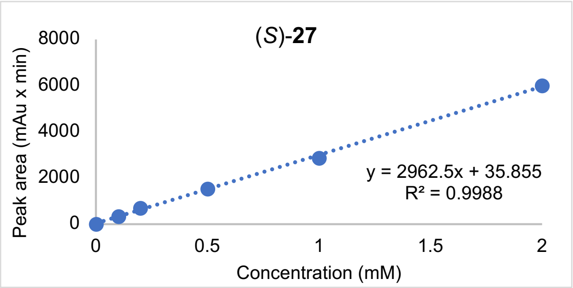

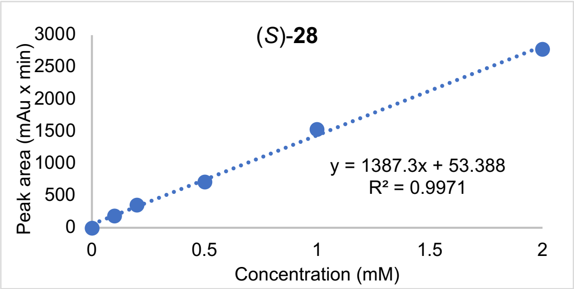

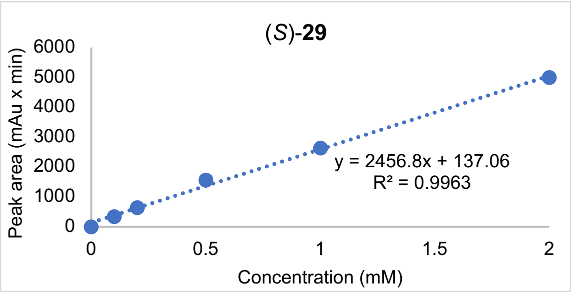

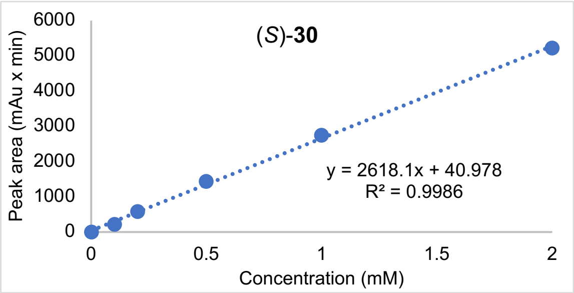

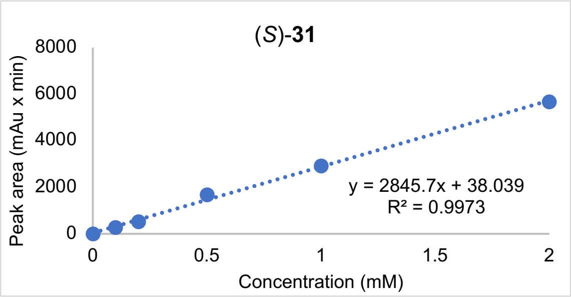

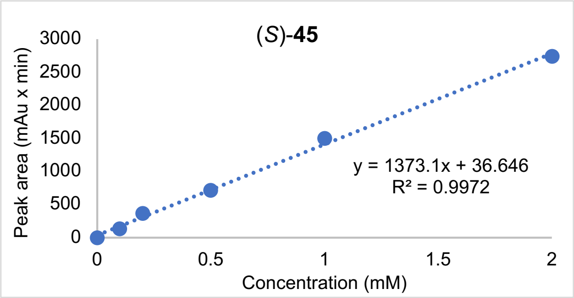

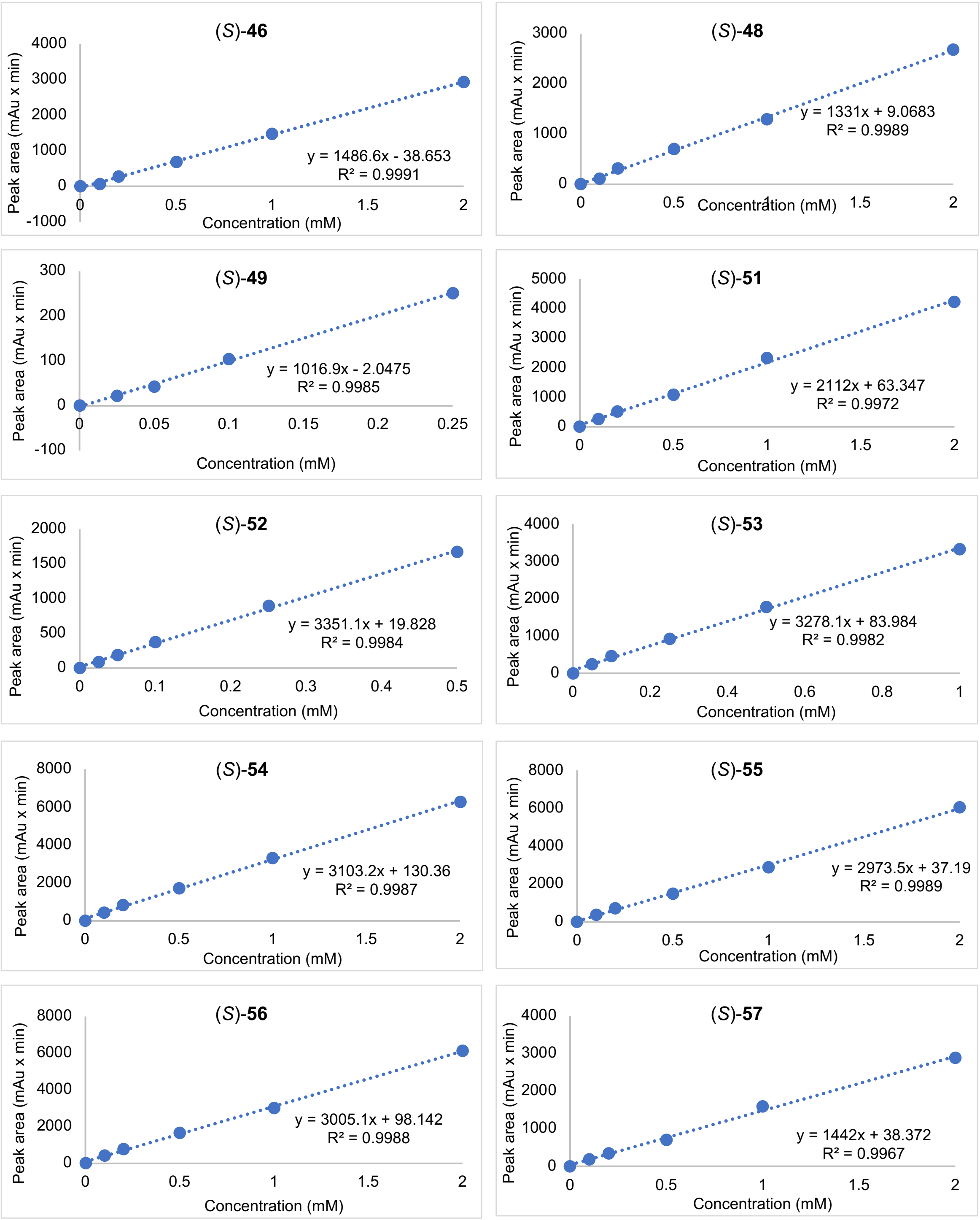

Supplementary Fig. 27 Calibration curves for analytical HPLC results of each product.

7 Chiral analytical HPLC results for cascade reaction products

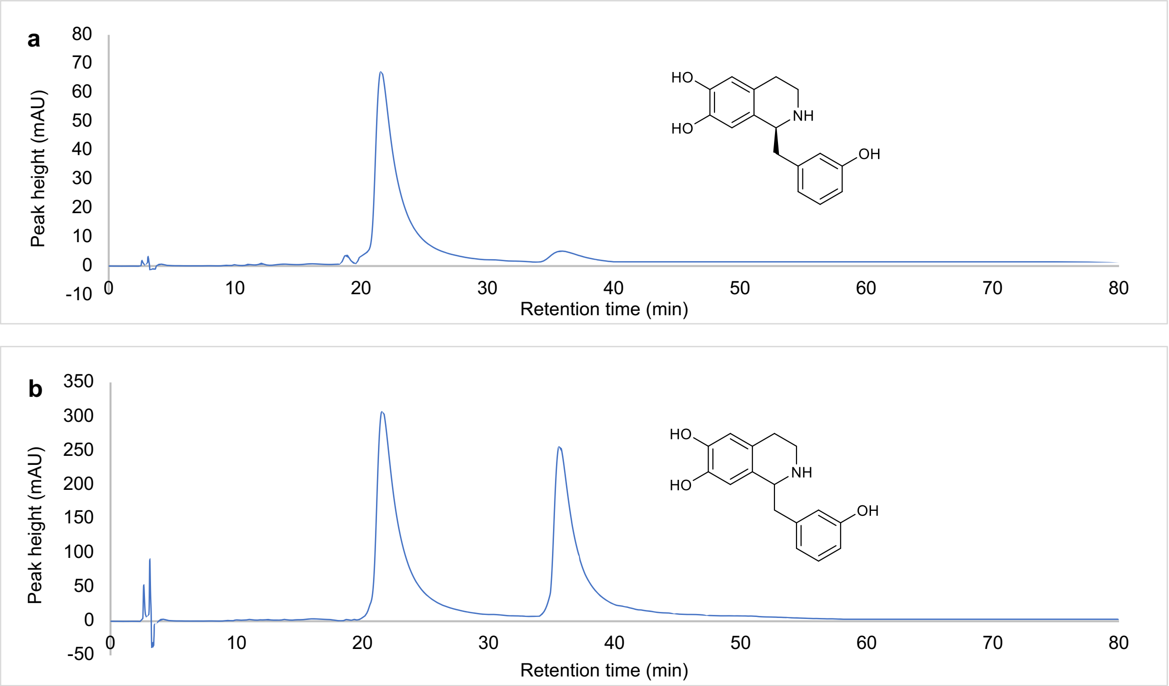

Supplementary Fig. 28 Chiral HPLC results for compounds (*S*)-23 and (*rac*)-23. a Chiral HPLC trace for (*S*)-**23**. **b** Chiral HPLC trace for (*rac*)-**23**. (Method 2, T2 column)

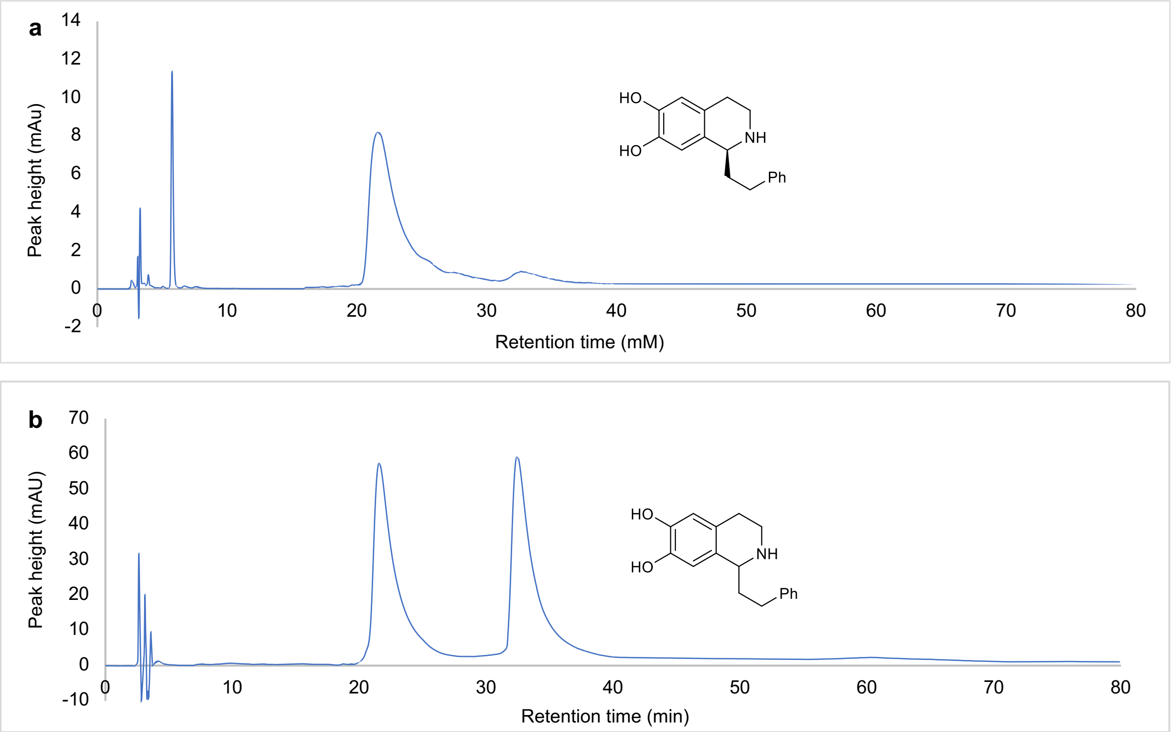

Supplementary Fig. 29 Chiral HPLC results for compounds (*S*)-24 and (*rac*)-24. a Chiral HPLC trace for (*S*)-**24**. **b** Chiral HPLC trace for (*rac*)-**24**. (Method 2, T2 column)

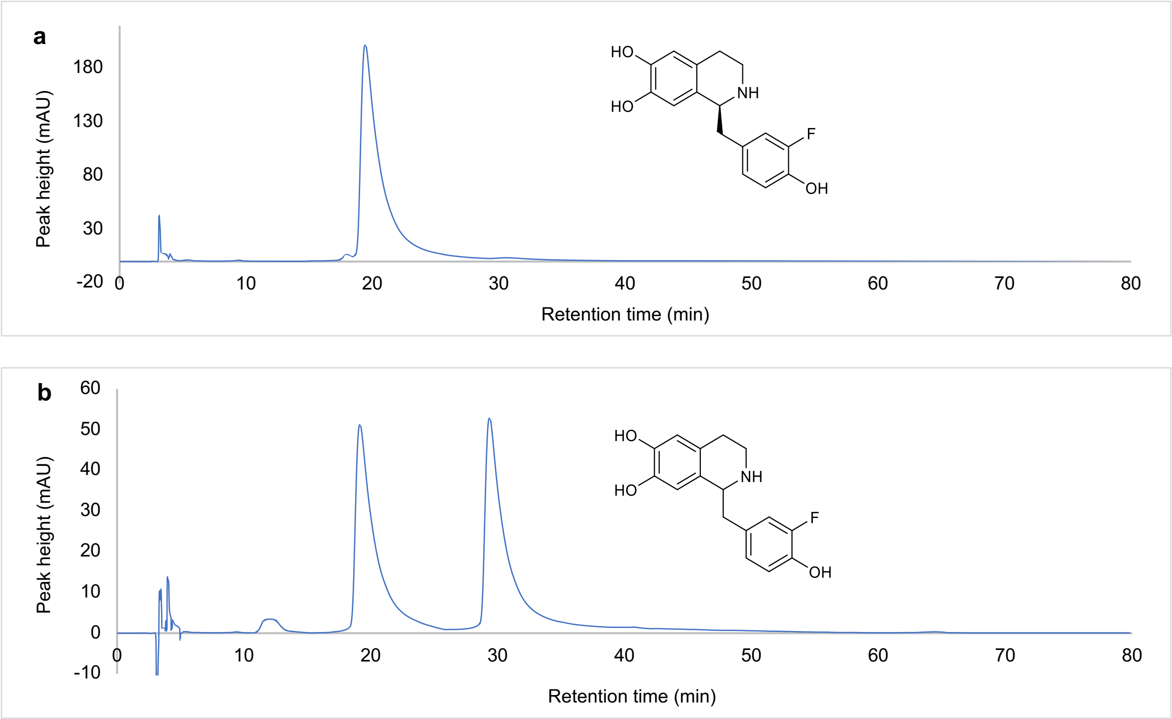

Supplementary Fig. 30 Chiral HPLC results for compounds (*S*)-25 and (*rac*)-25. a Chiral HPLC trace for (*S*)-**25**. **b** Chiral HPLC trace for (*rac*)-**25**. (Method 2, T2 column)

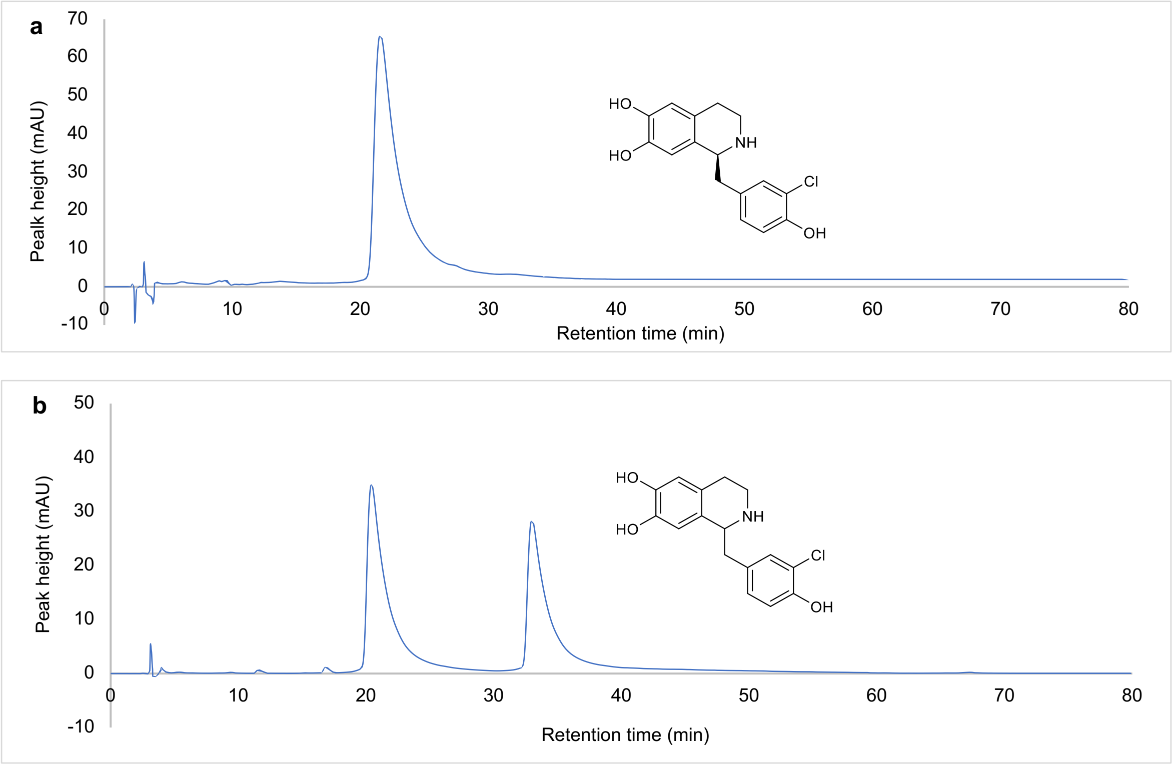

Supplementary Fig. 31 Chiral HPLC results for compounds (*S*)-26 and (*rac*)-26. a Chiral HPLC trace for (*S*)-**26**. **b** Chiral HPLC trace for (*rac*)-**26**. (Method 2, T2 column)

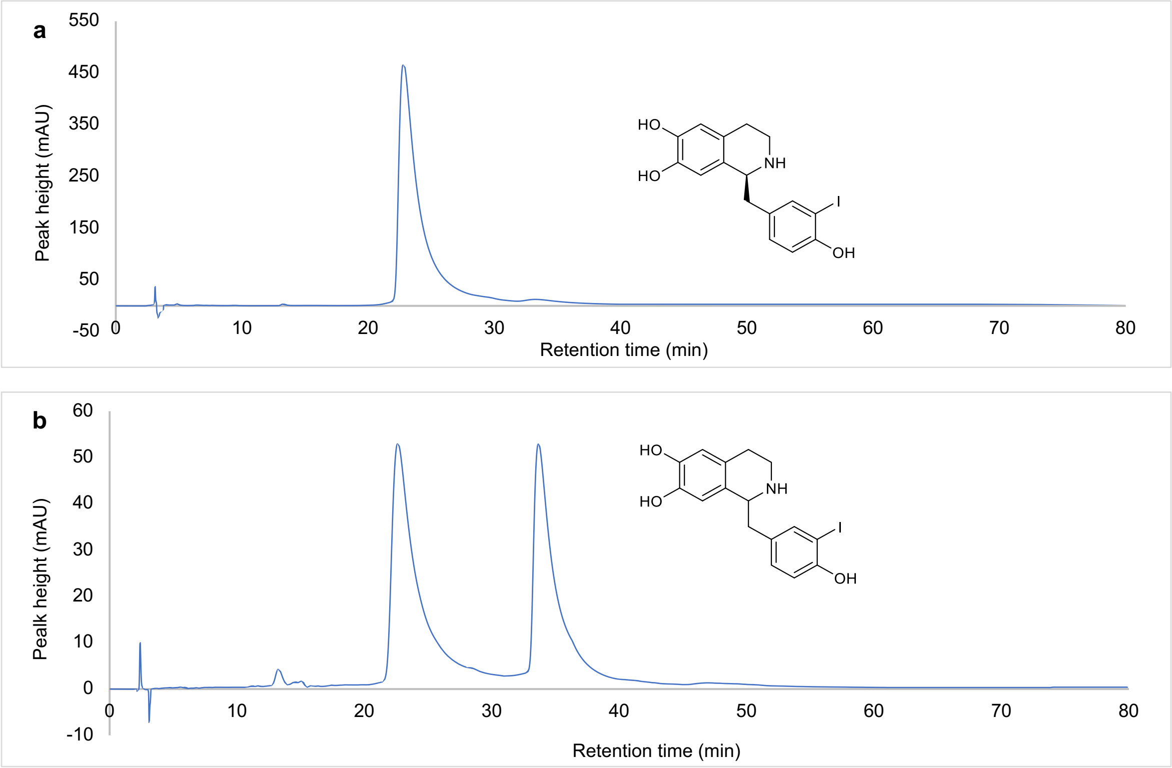

Supplementary Fig. 32 Chiral HPLC results for compounds (*S*)-27 and (*rac*)-27[2]. a Chiral HPLC trace for (*S*)-**27**. **b** Chiral HPLC trace for (*rac*)-**27**. (Method 2, T2 column)

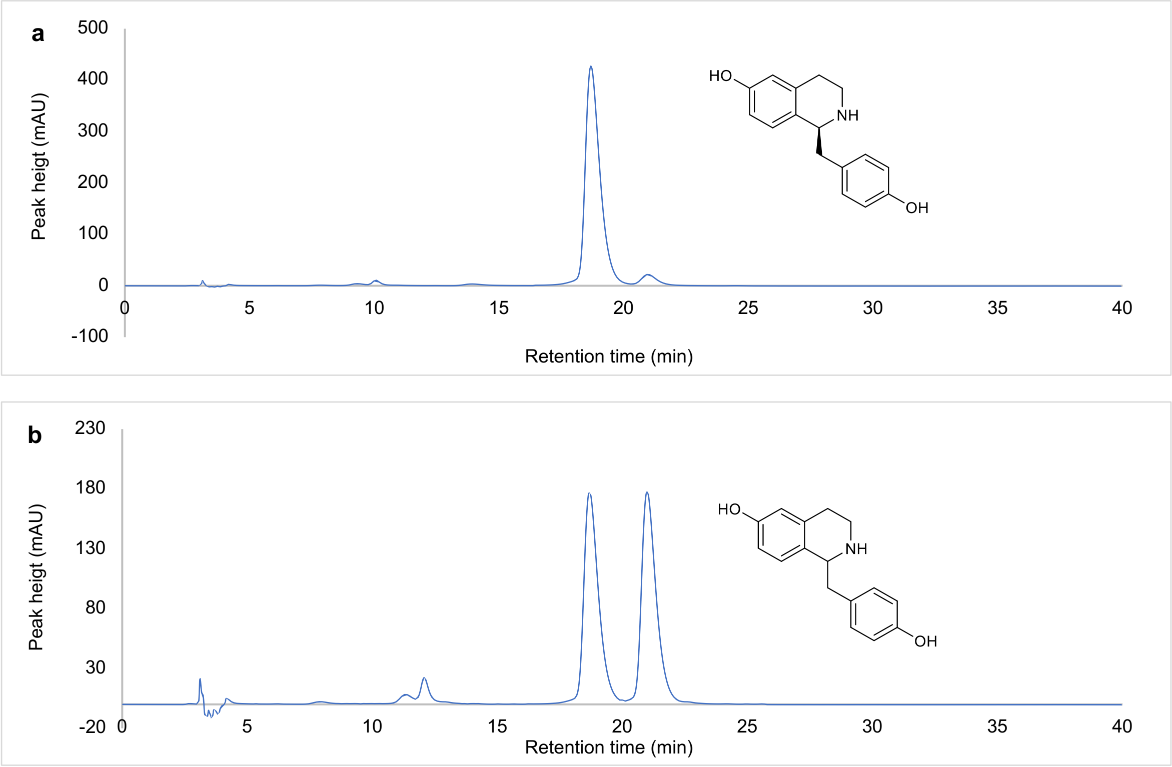

Supplementary Fig. 33 Chiral HPLC results for compounds (*S*)-28 and (*rac*)-28[3]. a Chiral HPLC trace for (*S*)-**28**. **b** Chiral HPLC trace for (*rac*)-**28**. (Method 2, T2 column)

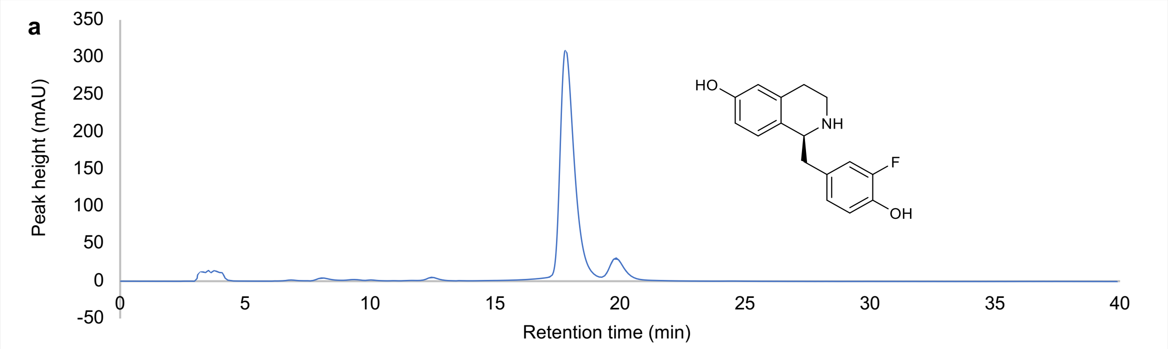

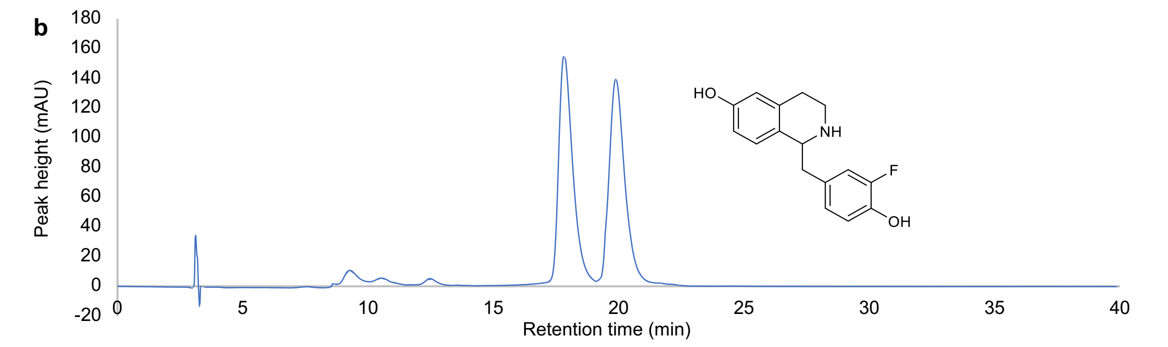

Supplementary Fig. 34 Chiral HPLC results for compounds (*S*)-29 and (*rac*)-29. a Chiral HPLC trace for (*S*)-**29**. **b** Chiral HPLC trace for (*rac*)-**29**. (Method 2, T2 column)

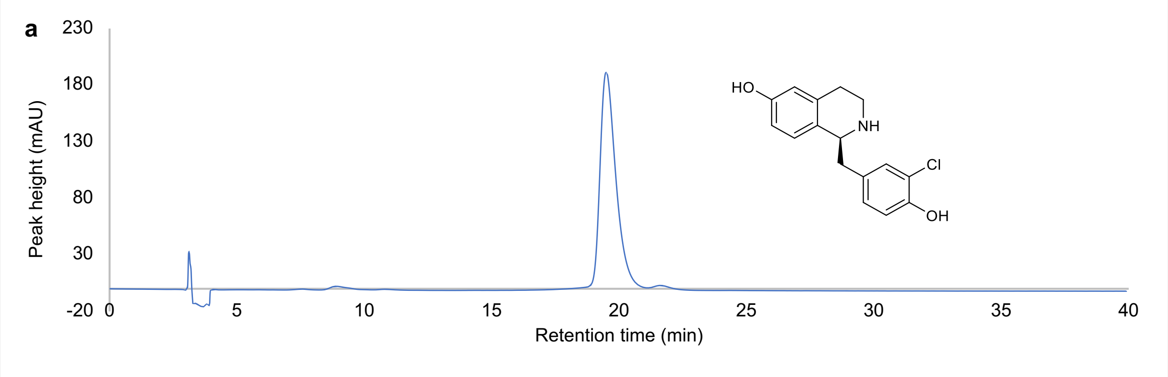

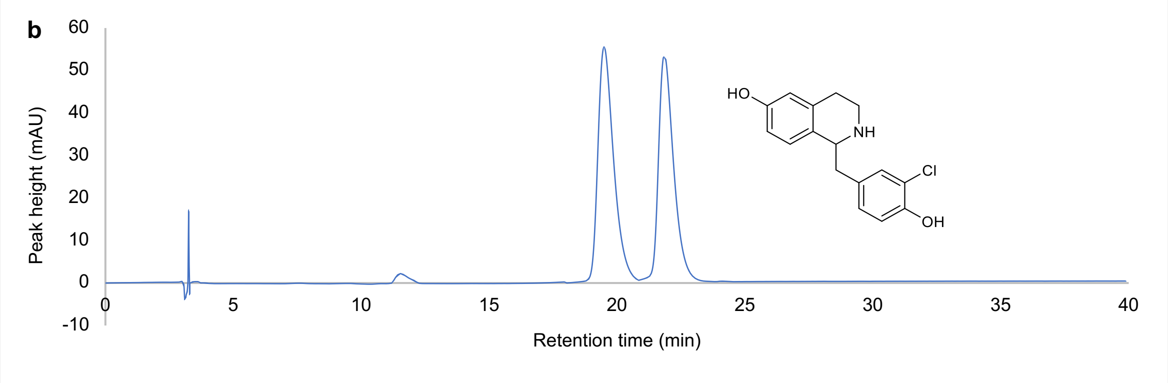

Supplementary Fig. 35 Chiral HPLC results for compounds (*S*)-30 and (*rac*)-30. a Chiral HPLC trace for (*S*)-**30**. **b** Chiral HPLC trace for (*rac*)-**30**. (Method 2, T2 column)

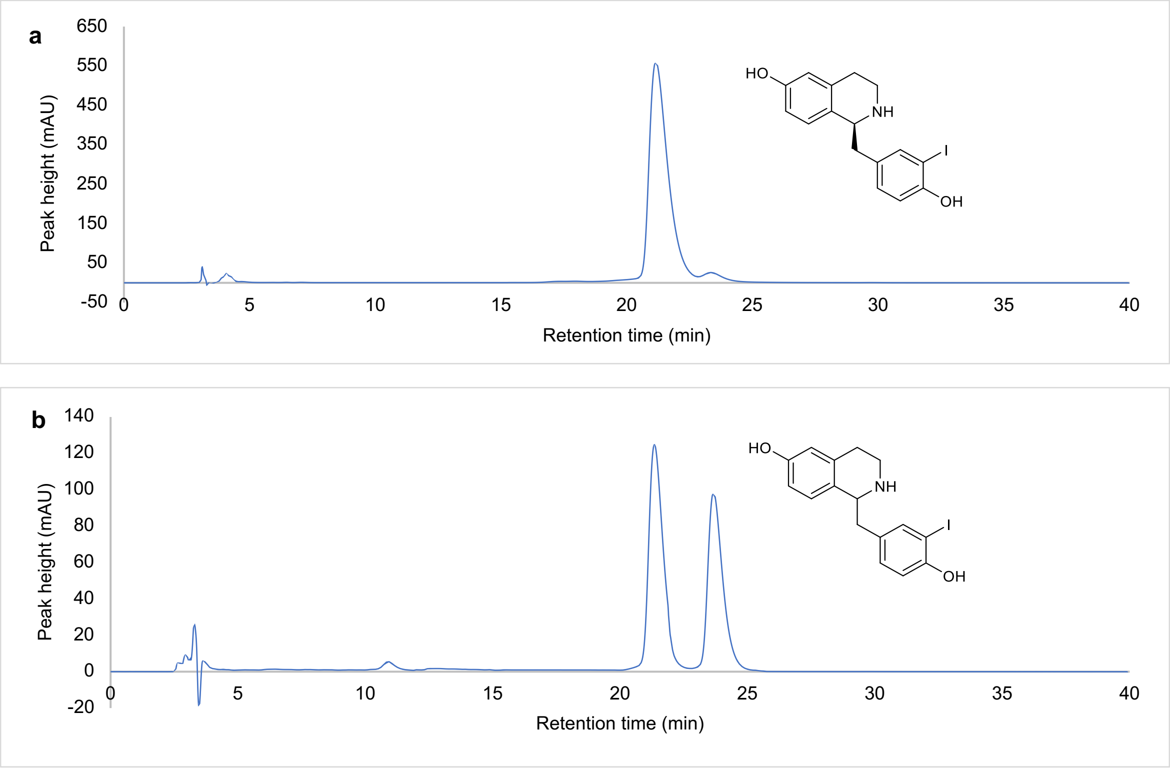

Supplementary Fig. 36 Chiral HPLC results for compounds (*S*)-31 and (*rac*)-31. a Chiral HPLC trace for (*S*)-**31**. **b** Chiral HPLC trace for (*rac*)-**31**. (Method 2, T2 column)

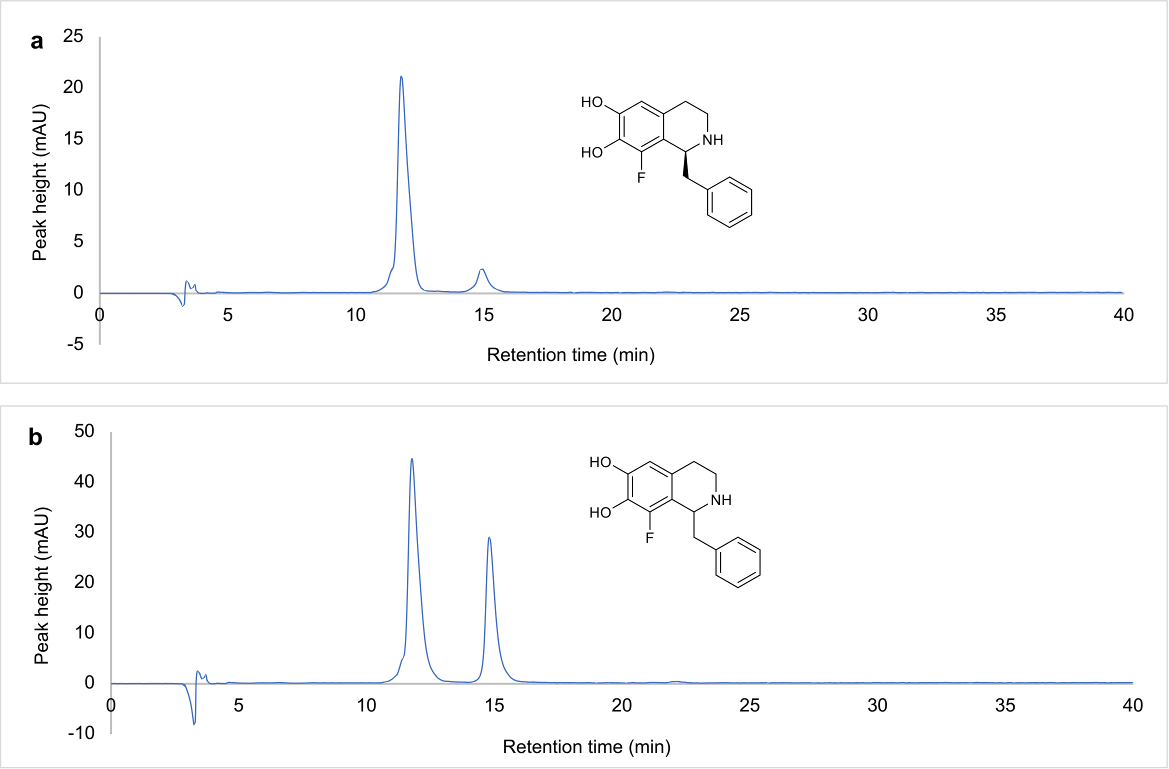

Supplementary Fig. 37 Chiral HPLC results for compounds (*S*)-45 and (*rac*)-45[4]**. a** Chiral HPLC trace for (*S*)-**45**. **b** Chiral HPLC trace for (*rac*)-**45**. (Method 2, T2 column)

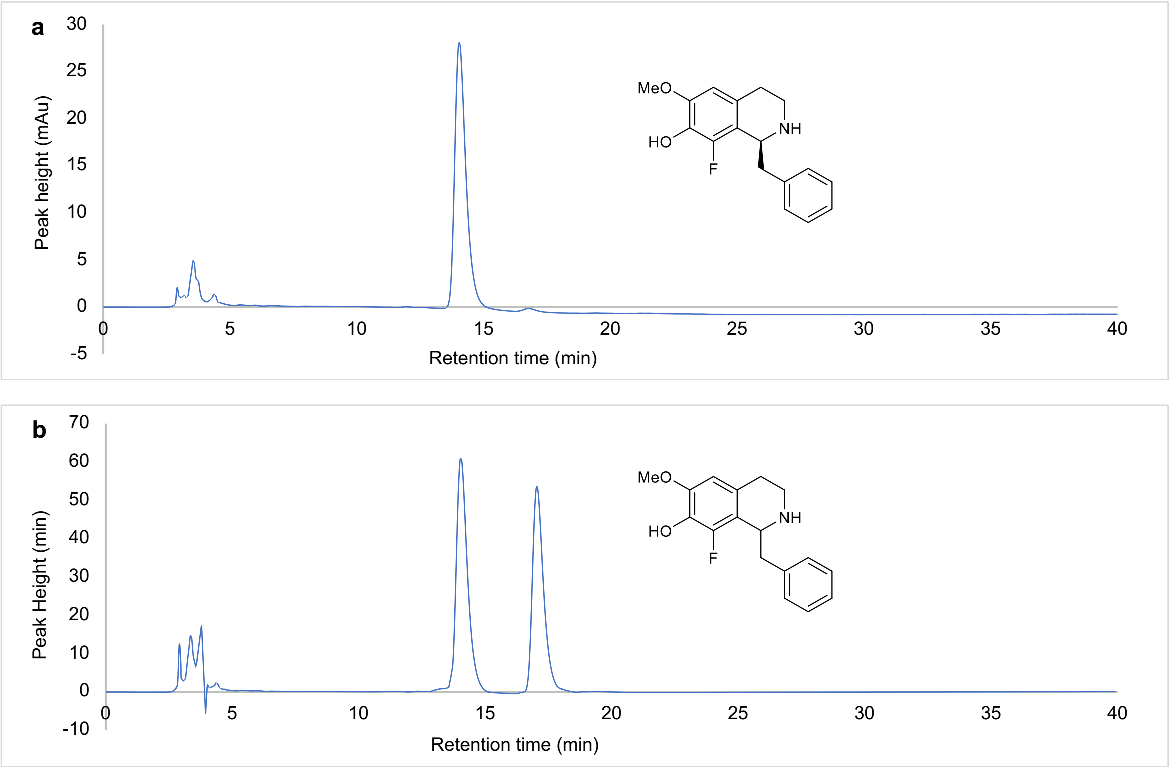

Supplementary Fig. 38 Chiral HPLC results for compounds (*S*)-46 and (*rac*)-46[5]**. a** Chiral HPLC trace for (*S*)-**46**. **b** Chiral HPLC trace for (*rac*)-**46**. (Method 2, T2 column)

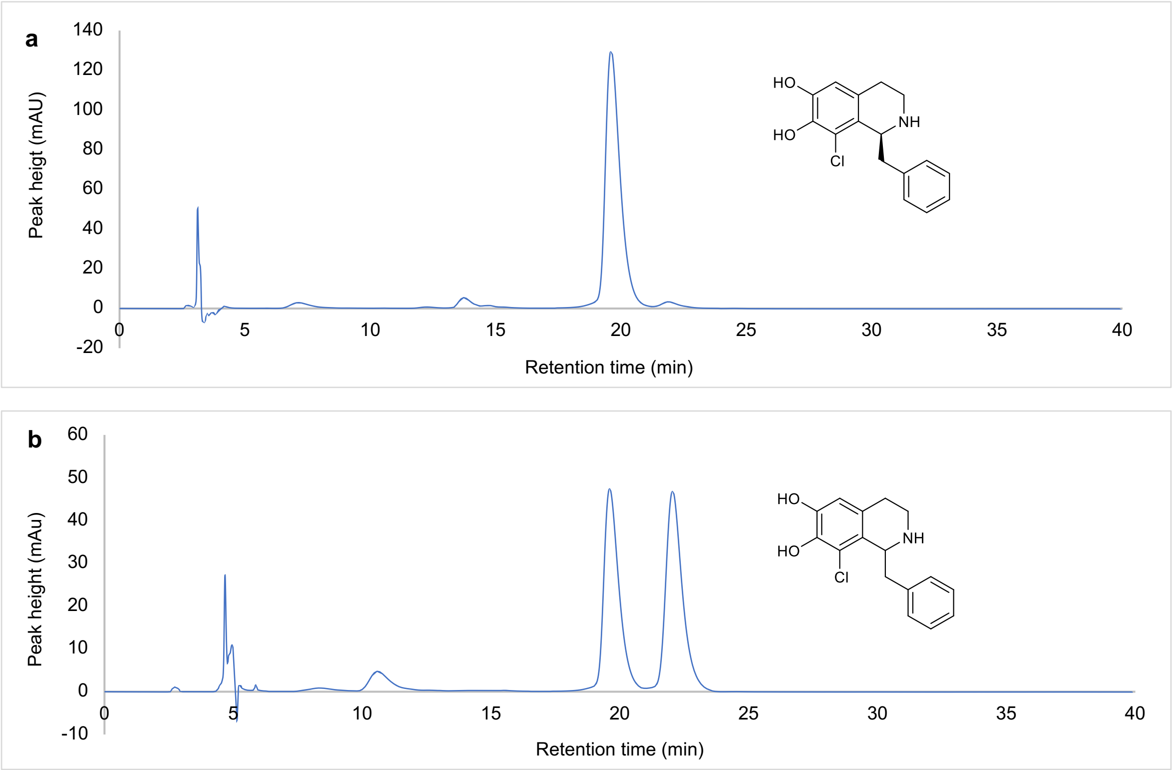

Supplementary Fig. 39 Chiral HPLC results for compounds (*S*)-48 and (*rac*)-48. a Chiral HPLC trace for (*S*)-**48**. **b** Chiral HPLC trace for (*rac*)-**48**. (Method 2, T2 column)

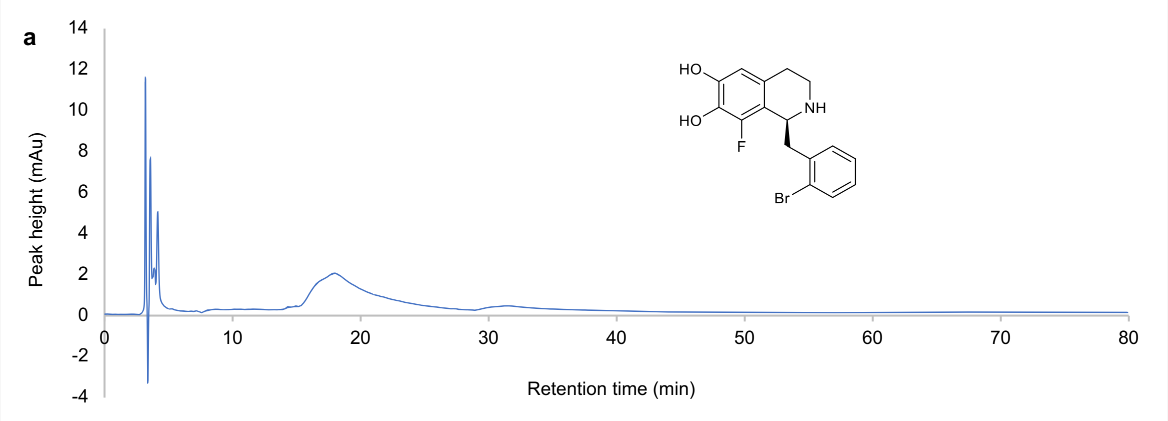

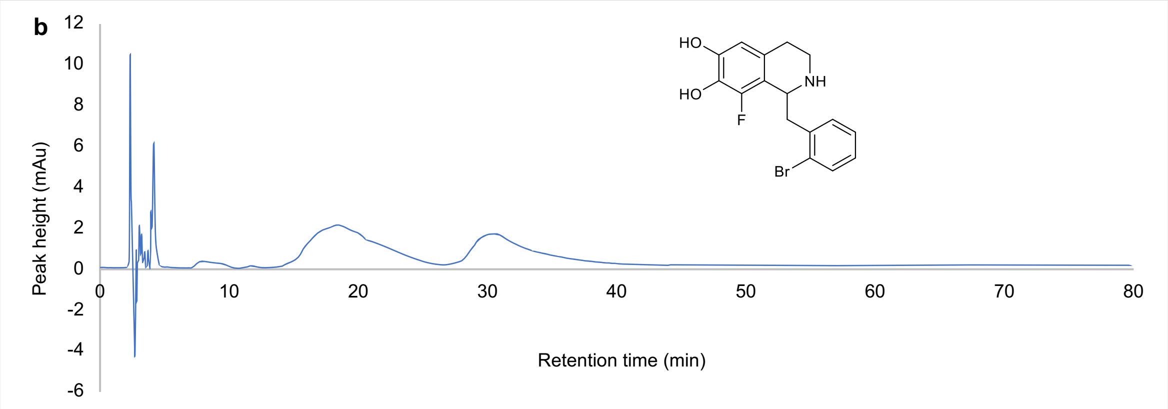

Supplementary Fig. 40 Chiral HPLC results for compounds (*S*)-51 and (*rac*)-51. a Chiral HPLC trace for (*S*)-**51**. **b** Chiral HPLC trace for (*rac*)-**51**. (Method 2, T2 column)

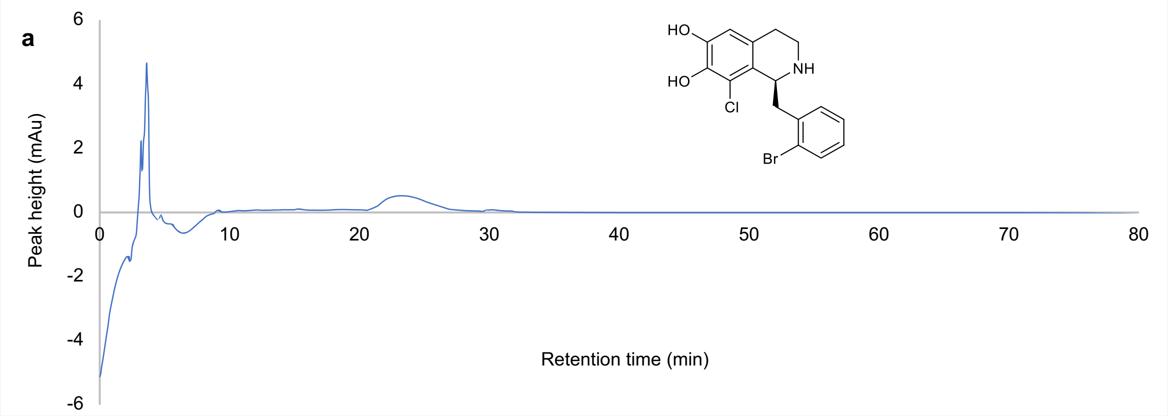

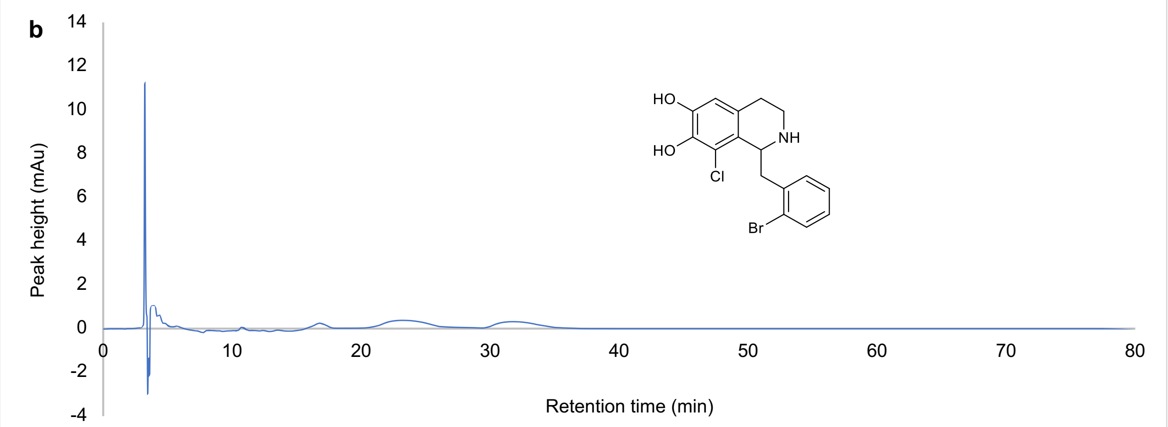

Supplementary Fig. 41 Chiral HPLC results for compounds (*S*)-52 and (*rac*)-52. a Chiral HPLC trace for (*S*)-**52**. **b** Chiral HPLC trace for (*rac*)-**52**. (Method 2, T2 column)

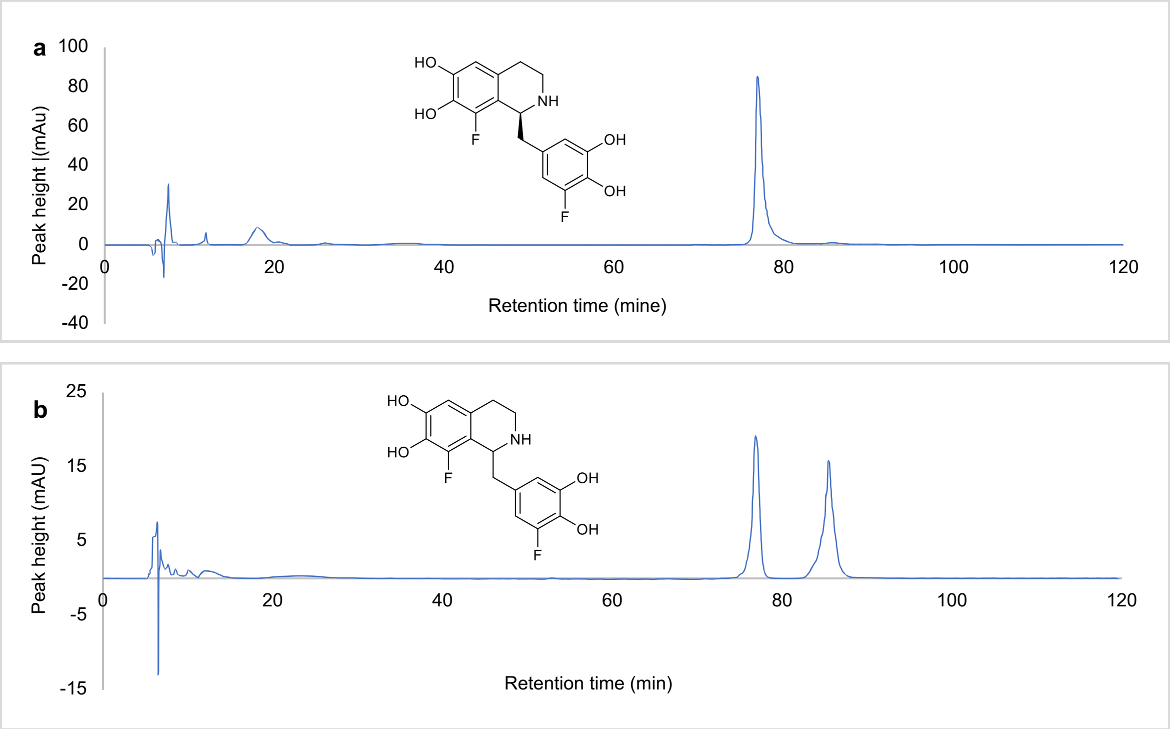

Supplementary Fig. 42 Chiral HPLC results for compounds (*S*)-53 and (*rac*)-53. a Chiral HPLC trace for (*S*)-**53**. **b** Chiral HPLC trace for (*rac*)-**53**. (Method 3, T column)

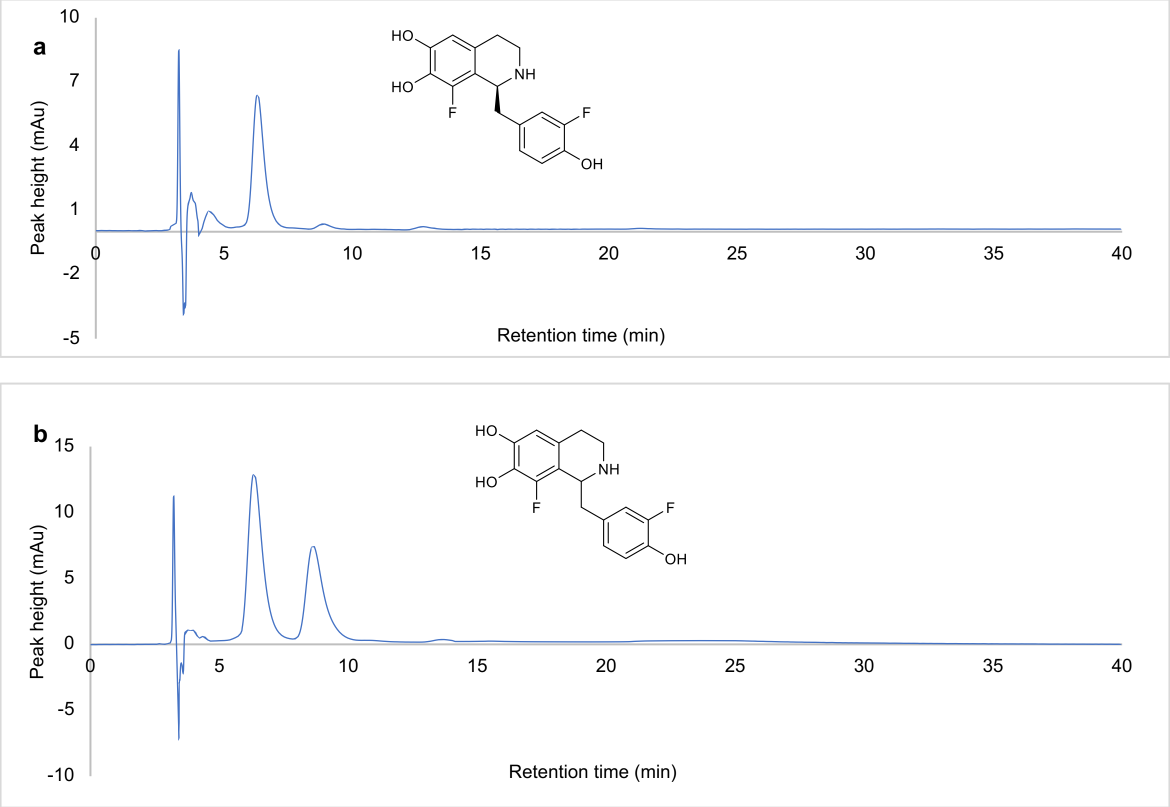

Supplementary Fig. 43 Chiral HPLC results for compounds (*S*)-54 and (*rac*)-54. a Chiral HPLC trace for (*S*)-**54**. **b** Chiral HPLC trace for (*rac*)-**54**. (Method 2, T column)

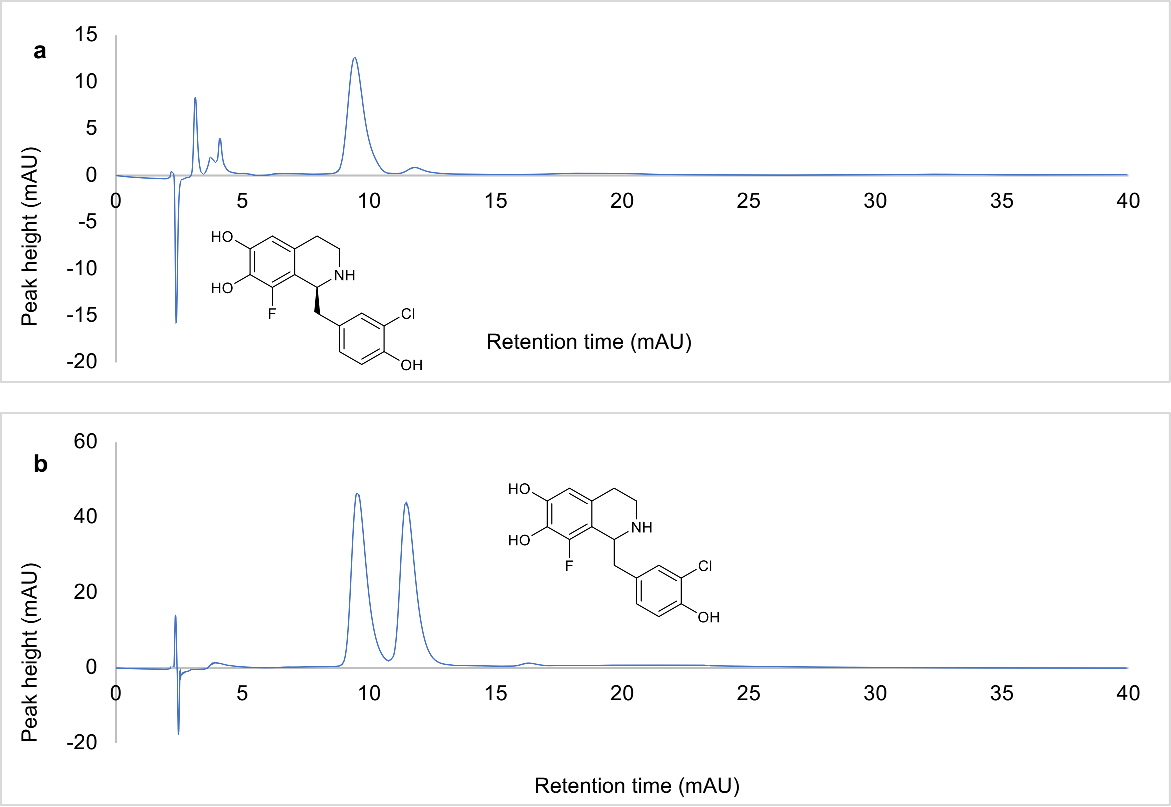

Supplementary Fig. 44 Chiral HPLC results for compounds (*S*)-55 and (*rac*)-55. a Chiral HPLC trace for (*S*)-**55**. **b** Chiral HPLC trace for (*rac*)-**55**. (Method 2, T column)

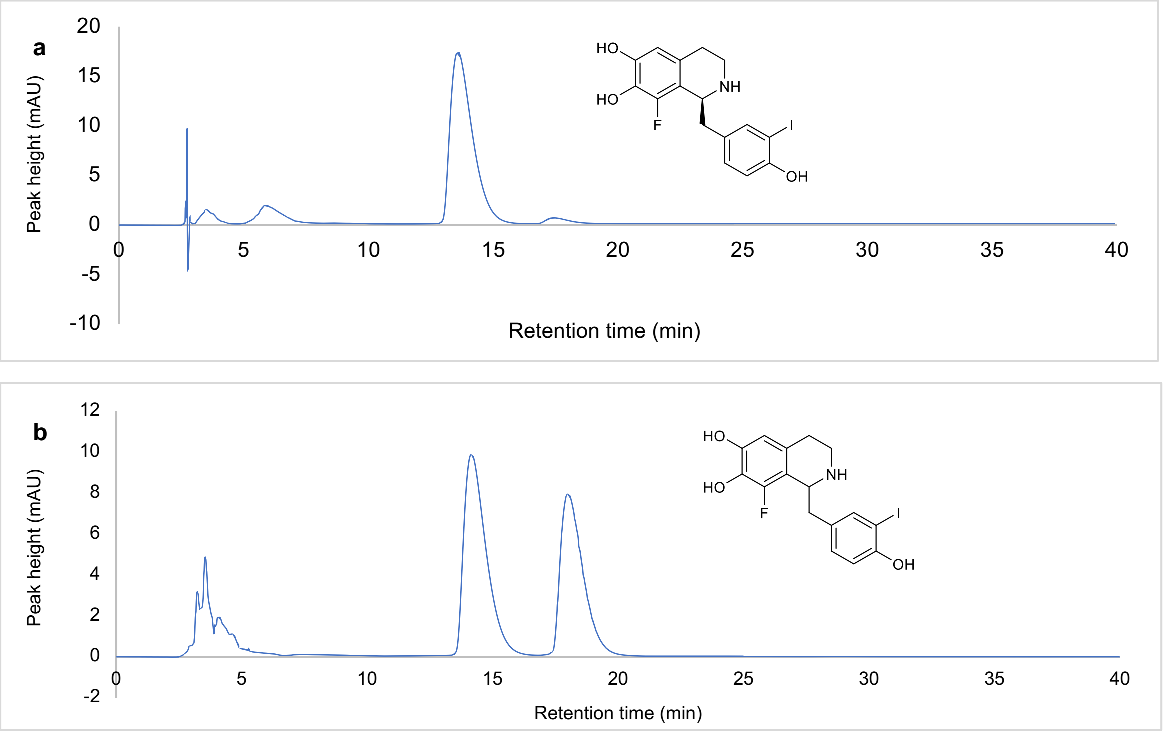

Supplementary Fig. 45 Chiral HPLC results for compounds (*S*)-56 and (*rac*)-56. a Chiral HPLC trace for (*S*)-**56**. **b** Chiral HPLC trace for (*rac*)-**56**. (Method 2, T column)

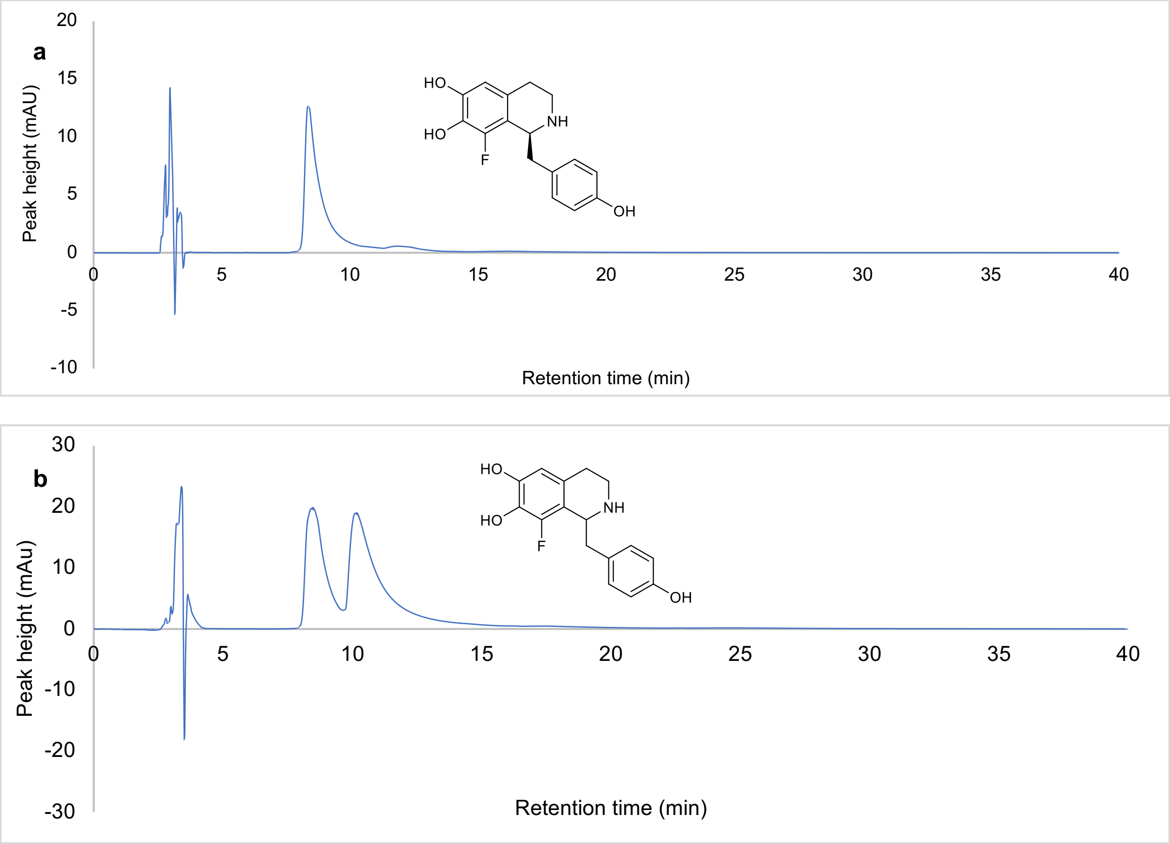

Supplementary Fig. 46 Chiral HPLC results for compounds (*S*)-57 and (*rac*)-57. a Chiral HPLC trace for (*S*)-**57**. **b** Chiral HPLC trace for (*rac*)-**57**. (Method 2, T column)

8 Accurate mass spectra for the novel products

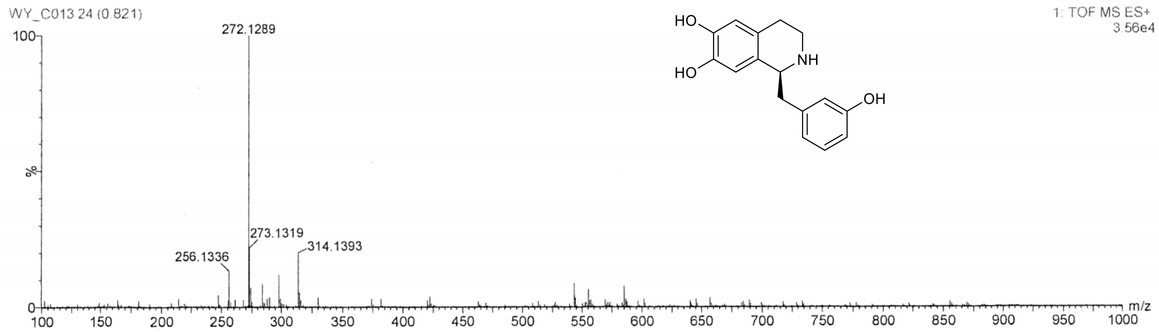

Supplementary Fig. 47 Accurate mass spectra for (*S*)-1-(3-hydroxybenzyl)-1,2,3,4-tetrahydroisoquinoline-6,7-diol (*S*)-**23**.

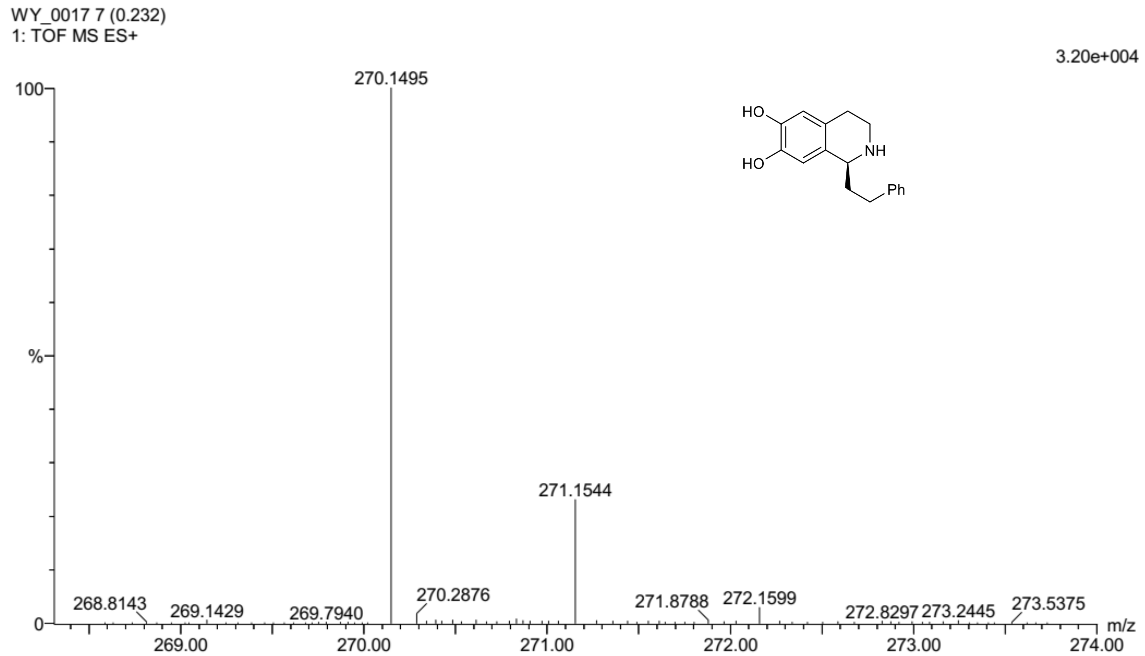

Supplementary Fig. 48 Accurate mass spectra for (*S*)-1-Phenethyl-1,2,3,4-tetrahydroisoquinoline-6,7-diol (*S*)-**24**[1].

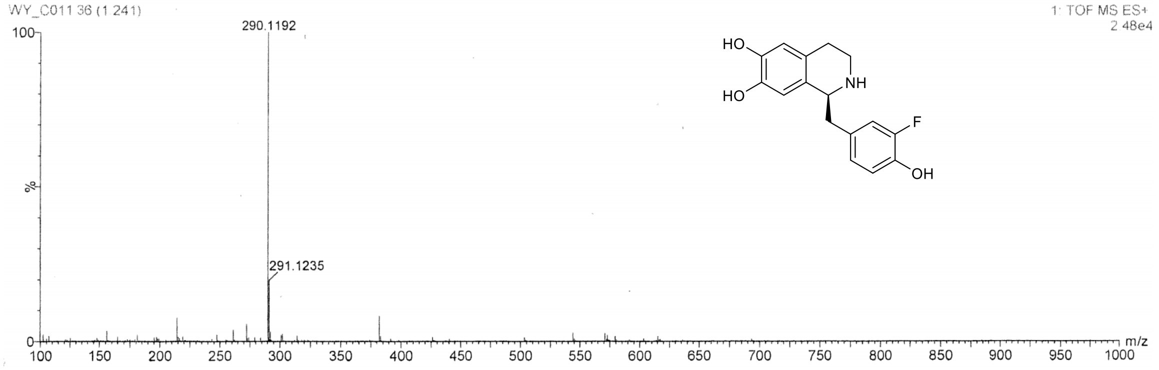

Supplementary Fig. 49 Accurate mass spectra for (*S*)-1-(3-fluoro-4-hydroxybenzyl)-1,2,3,4-tetrahydroisoquinoline-6,7-diol (*S*)-**25**.

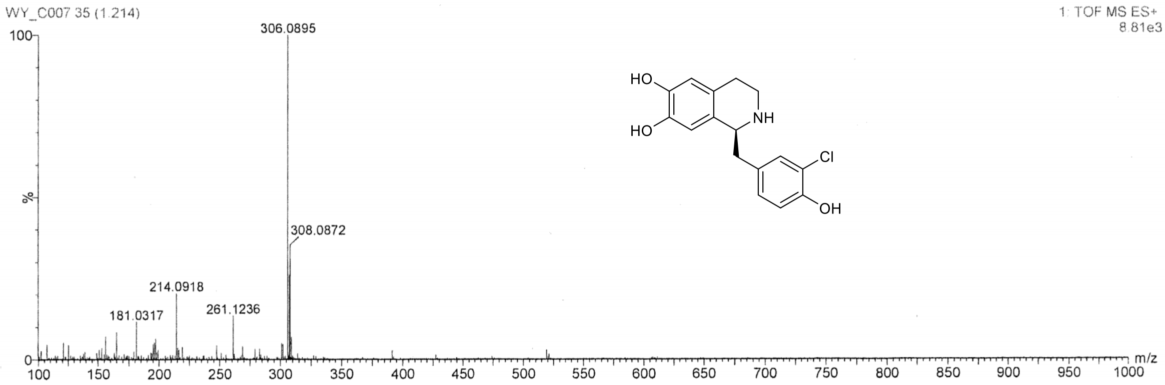

Supplementary Fig. 50 Accurate mass spectra for (*S*)-1-(3-chloro-4-hydroxybenzyl)-1,2,3,4-tetrahydroisoquinoline-6,7-diol (*S*)-**26**[2].

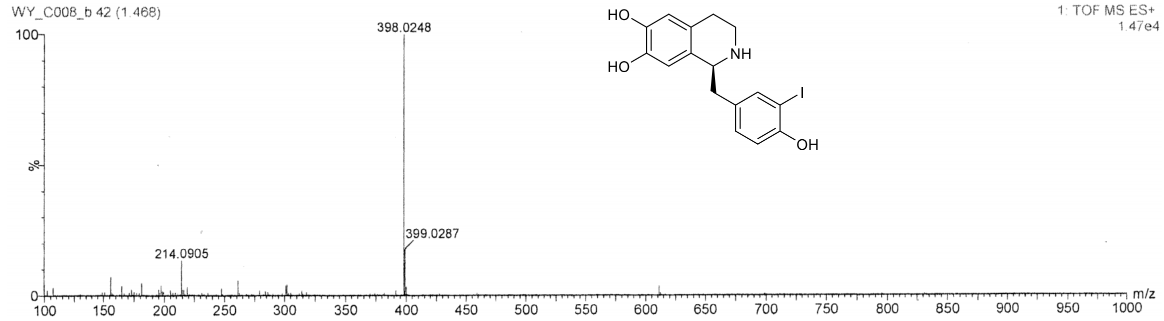

Supplementary Fig. 51 Accurate mass spectra for (*S*)-1-(3-iodo-4-hydroxybenzyl)-1,2,3,4-tetrahydroisoquinoline-6,7-diol (*S*)-**27**[2].

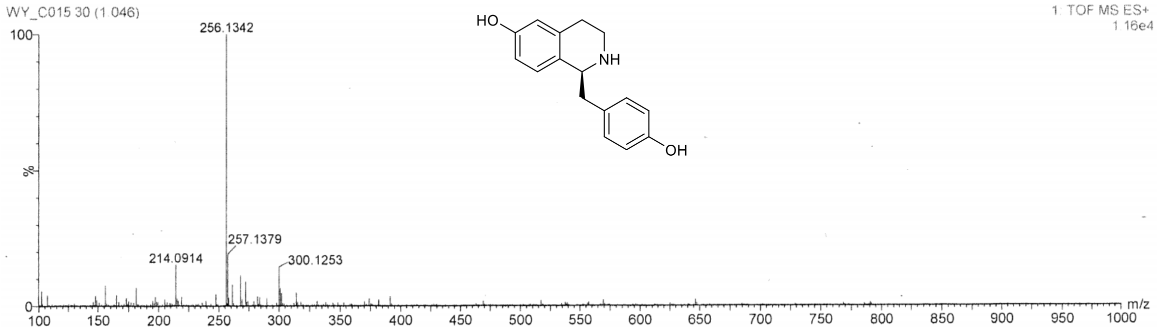

Supplementary Fig. 52 Accurate mass spectra for (*S*)-1-(4-hydroxybenzyl)-1,2,3,4-tetrahydroisoquinolin-6-ol (*S*)-**28**[3].

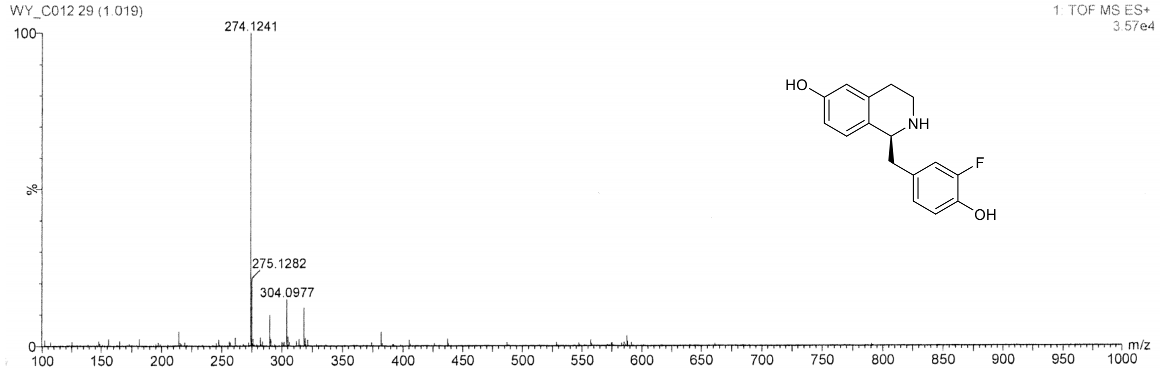

Supplementary Fig. 53 Accurate mass spectra for (*S*)-1-(3-fluoro-4-hydroxybenzyl)-1,2,3,4-tetrahydroisoquinolin-6-ol (*S*)-**29**.

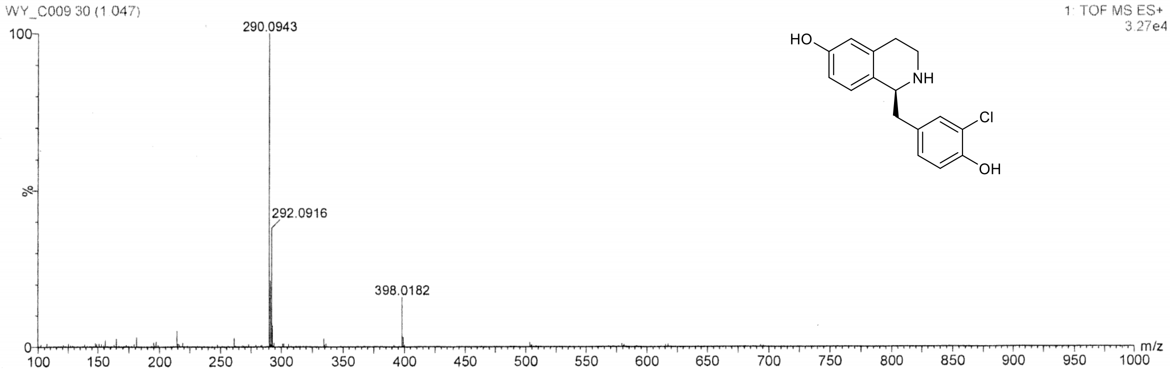

Supplementary Fig. 54 Accurate mass spectra for (*S*)-1-(3-chloro-4-hydroxybenzyl)-1,2,3,4-tetrahydroisoquinolin-6-ol (*S*)-**30**.

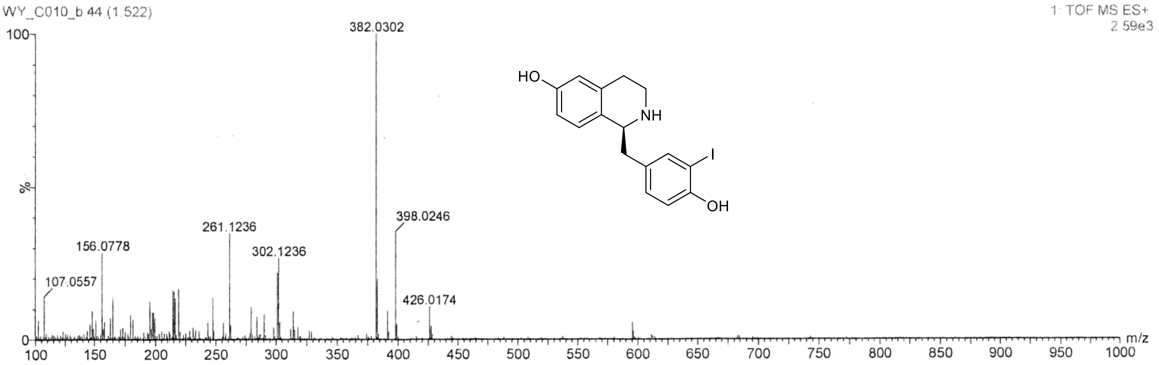

Supplementary Fig. 55 Accurate mass spectra for (*S*)-1-(3-iodo-4-hydroxybenzyl)-1,2,3,4-tetrahydroisoquinolin-6-ol (*S*)-**31**.

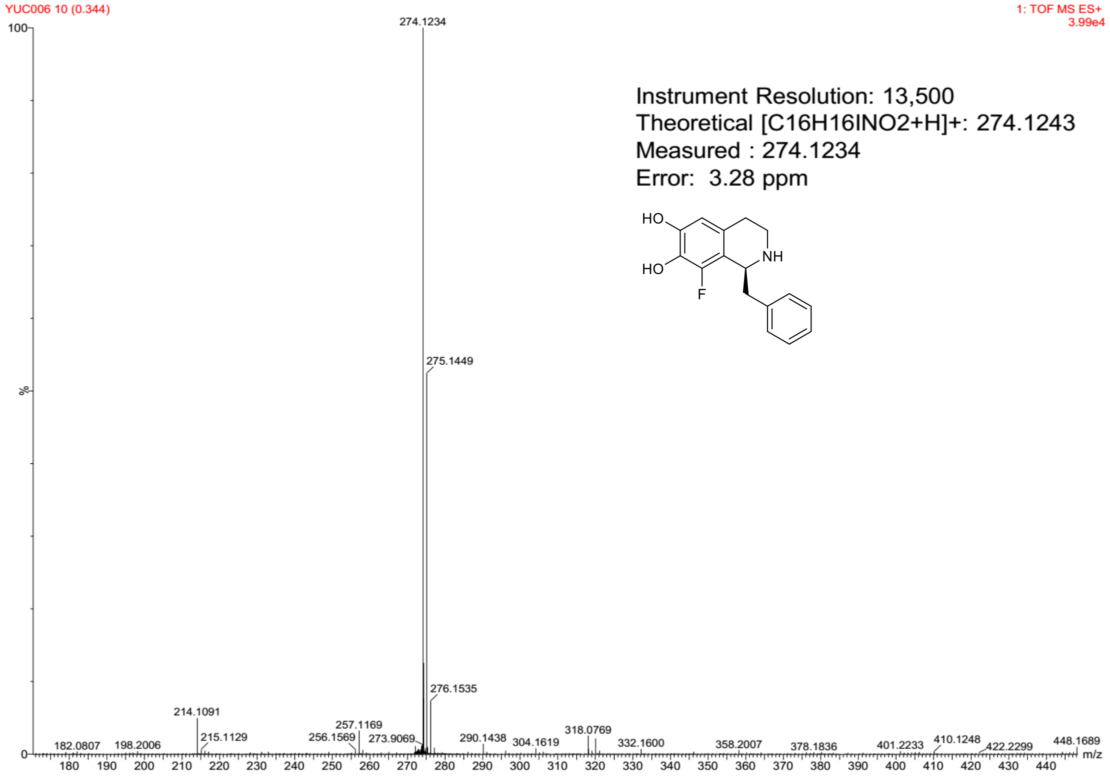

Supplementary Fig. 56 Accurate mass spectra for (*S*)-1-benzyl-8-fluoro-1,2,3,4-tetrahydroisoquinoline-6,7-diol (*S*)-**45**[4].

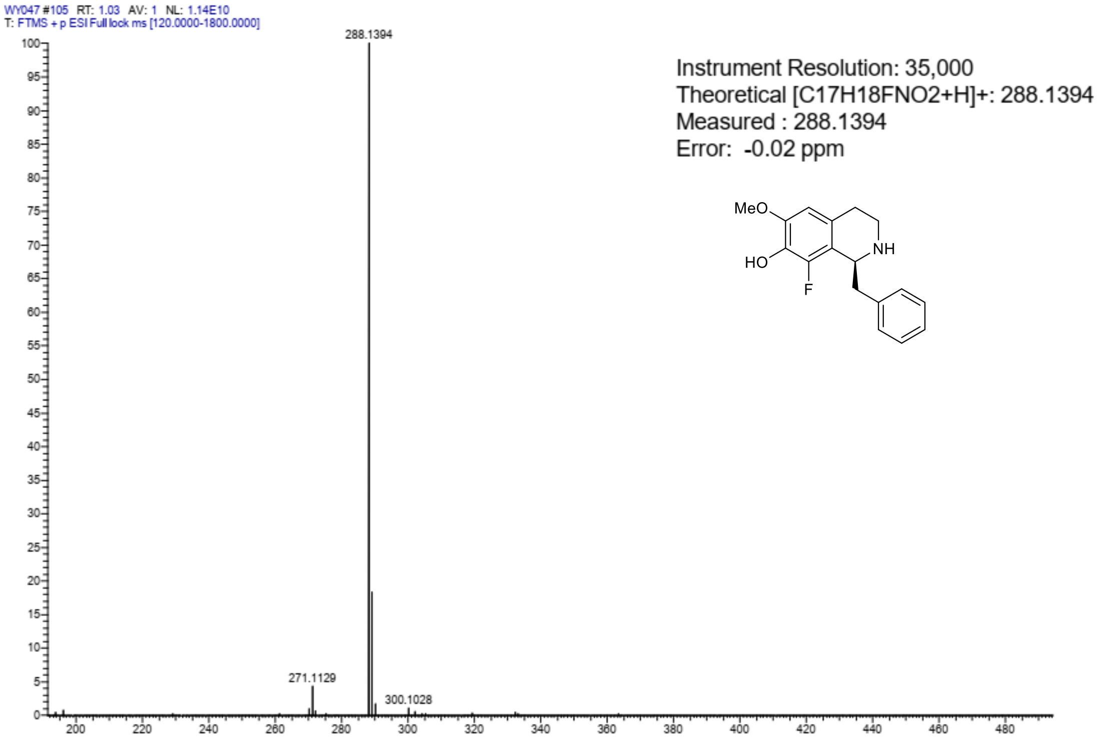

Supplementary Fig. 57 Accurate mass spectra for (*S*)-1-benzyl-8-fluoro-6-methoxy-1,2,3,4-tetrahydroisoquinolin-7-ol (*S*)-**46**[5].

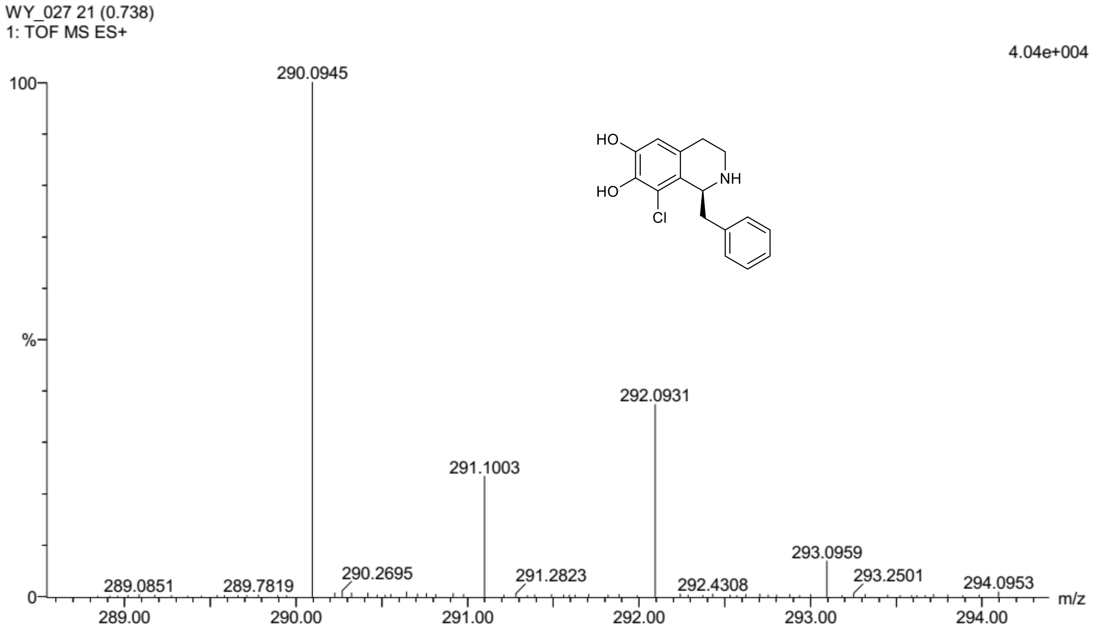

Supplementary Fig. 58 Accurate mass spectra for (*S*)-1-benzyl-8-chloro-1,2,3,4-tetrahydroisoquinoline-6,7-diol (*S*)-**48**.

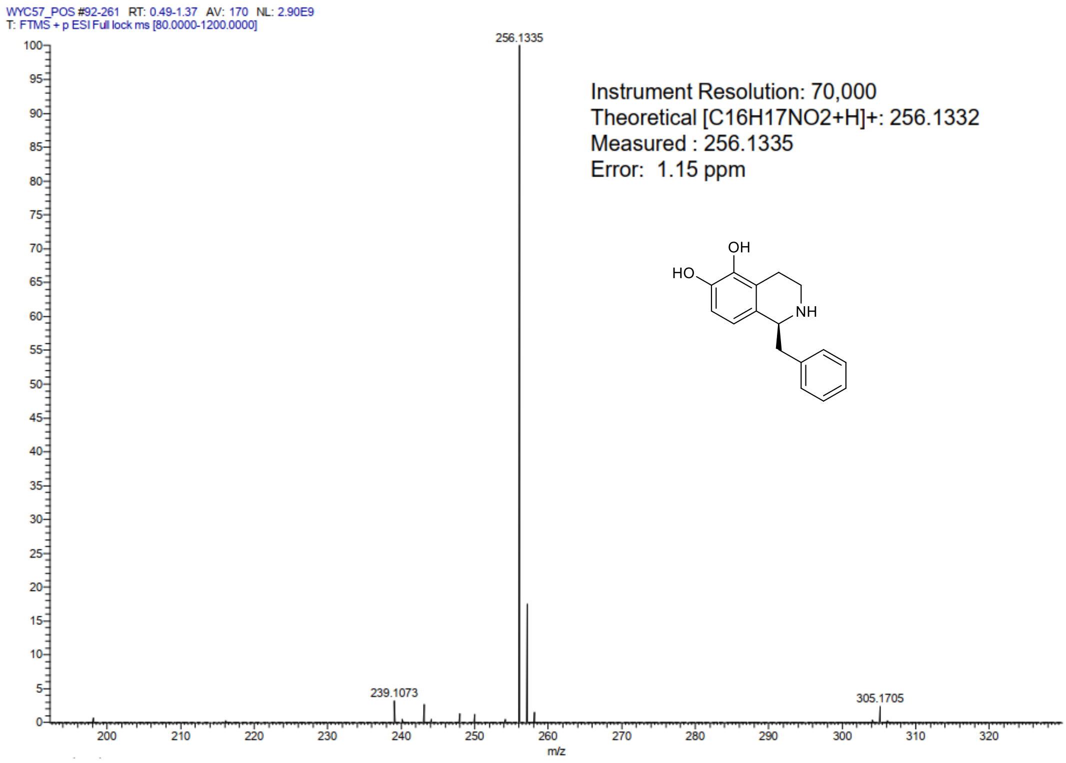

Supplementary Fig. 59 Accurate mass spectra for (*S*)-1-benzyl-1,2,3,4-tetrahydroisoquinoline-5,6-diol (*S*)-**49**.

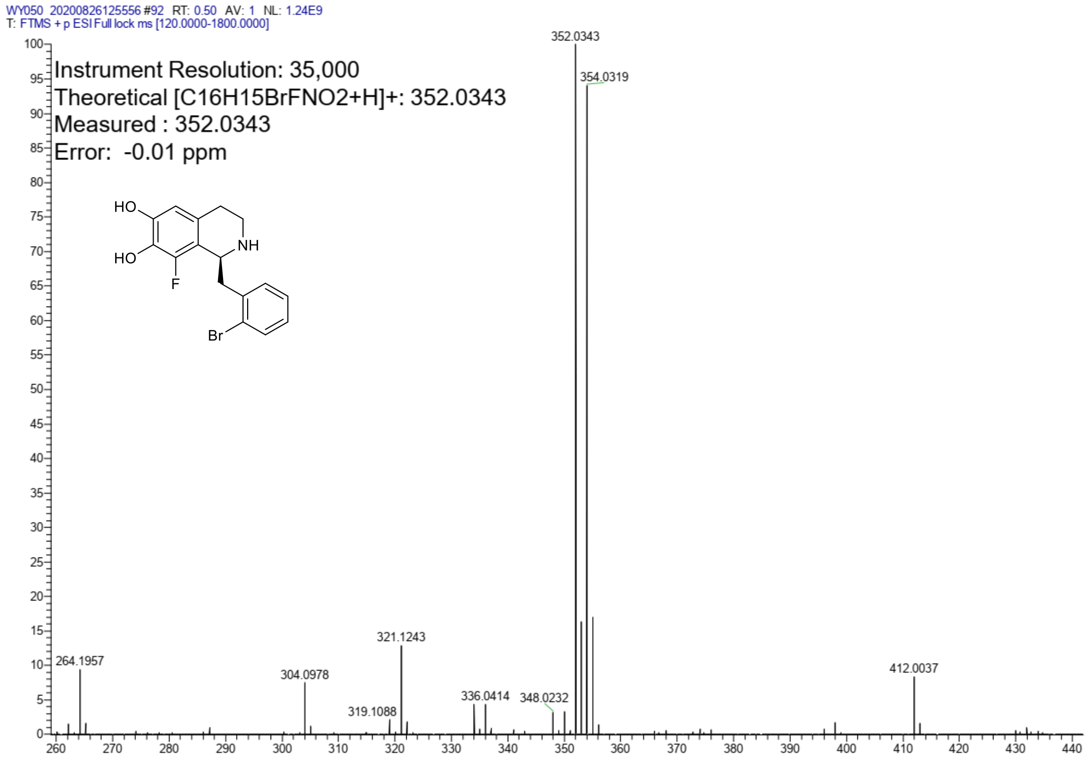

Supplementary Fig. 60 Accurate mass spectra for (*S*)-1-(2-bromobenzyl)-8-fluoro-1,2,3,4-THIQ-6,7-diol (*S*)-**51**.

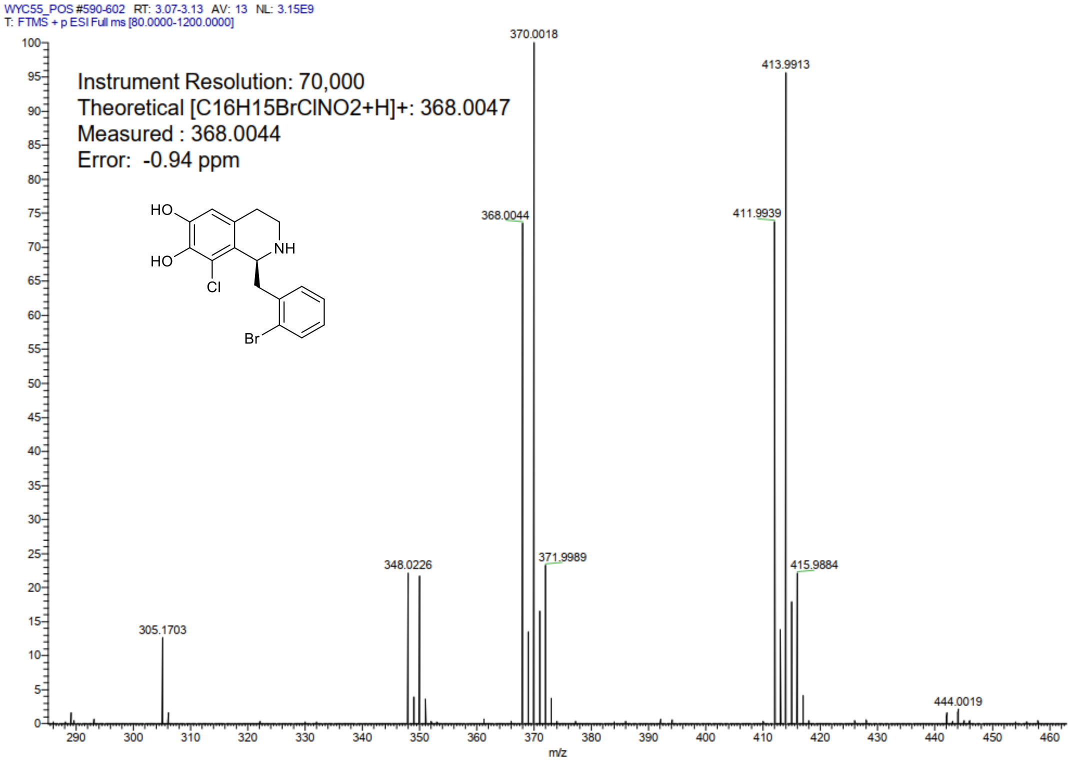

Supplementary Fig. 61 Accurate mass spectra for (*S*)-1-(2-bromobenzyl)-8-chloro-1,2,3,4-tetrahydroisoquinoline-6,7-diol (*S*)-**52**.

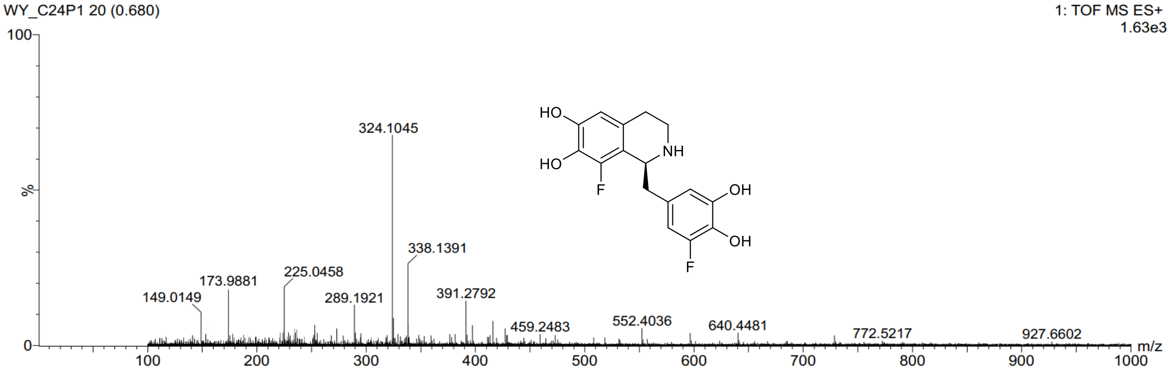

Supplementary Fig. 62 Accurate mass spectra for (*S*)-8-fluoro-1-(3-fluoro-4,5-dihydroxybenzyl)-1,2,3,4-tetrahydro-isoquinoline-6,7-diol (*S*)-**53**.

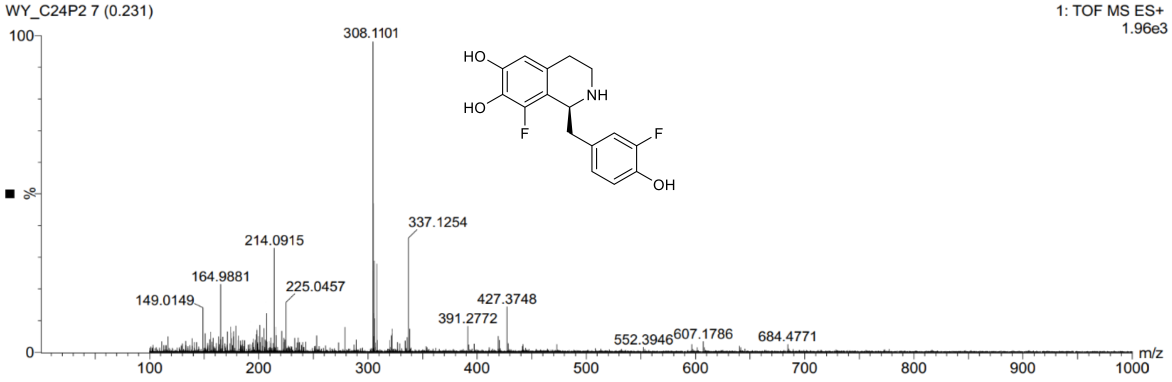

Supplementary Fig. 63 Accurate mass spectra for (*S*)-8-fluoro-1-(3-fluoro-4-hydroxybenzyl)-1,2,3,4-tetrahydro- isoquinoline-6,7-diol (*S*)-**54**.

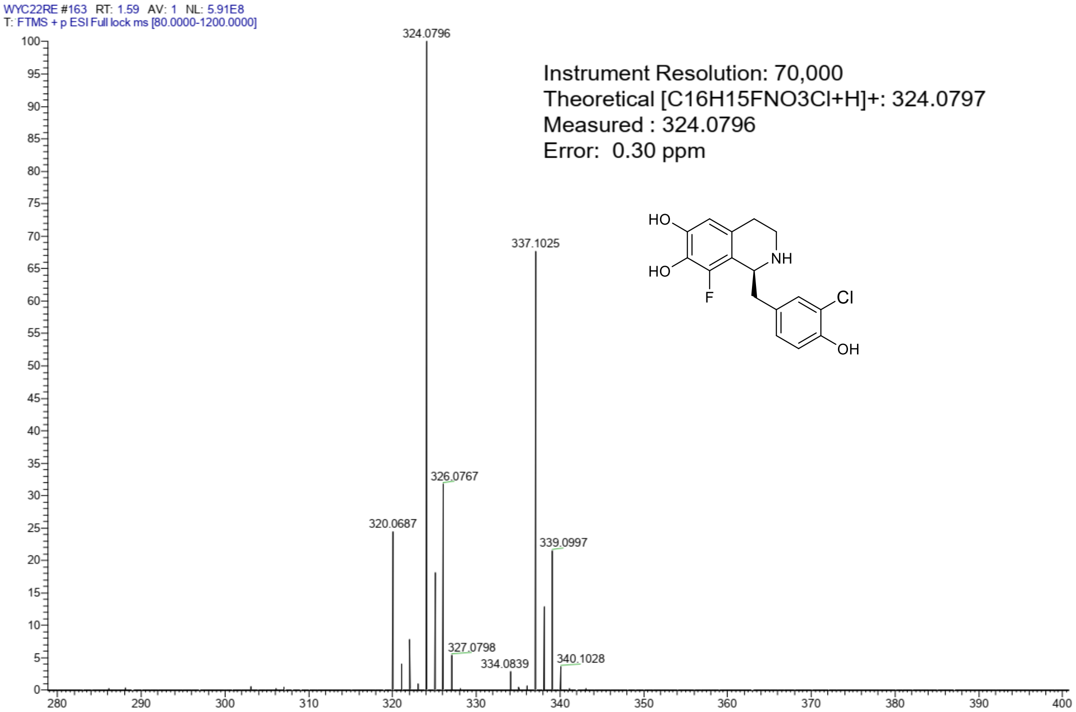

Supplementary Fig. 64 Accurate mass spectra for (*S*)-1-(3-chloro-4-hydroxybenzyl)-8-fluoro-1,2,3,4-tetrahydro-isoquinoline-6,7-diol (*S*)-**55**.

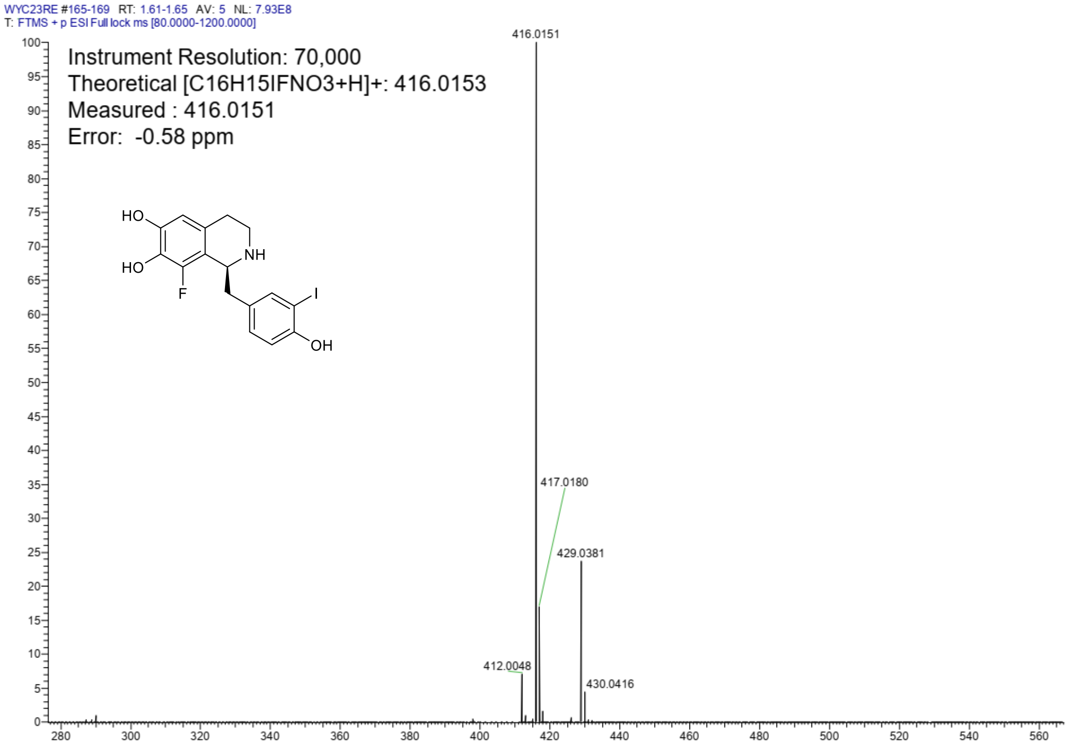

Supplementary Fig. 65 Accurate mass spectra for (*S*)-1-(3-iodo-4-hydroxybenzyl)-8-fluoro-1,2,3,4-tetrahydro-isoquinoline-6,7-diol (*S*)-**56**.

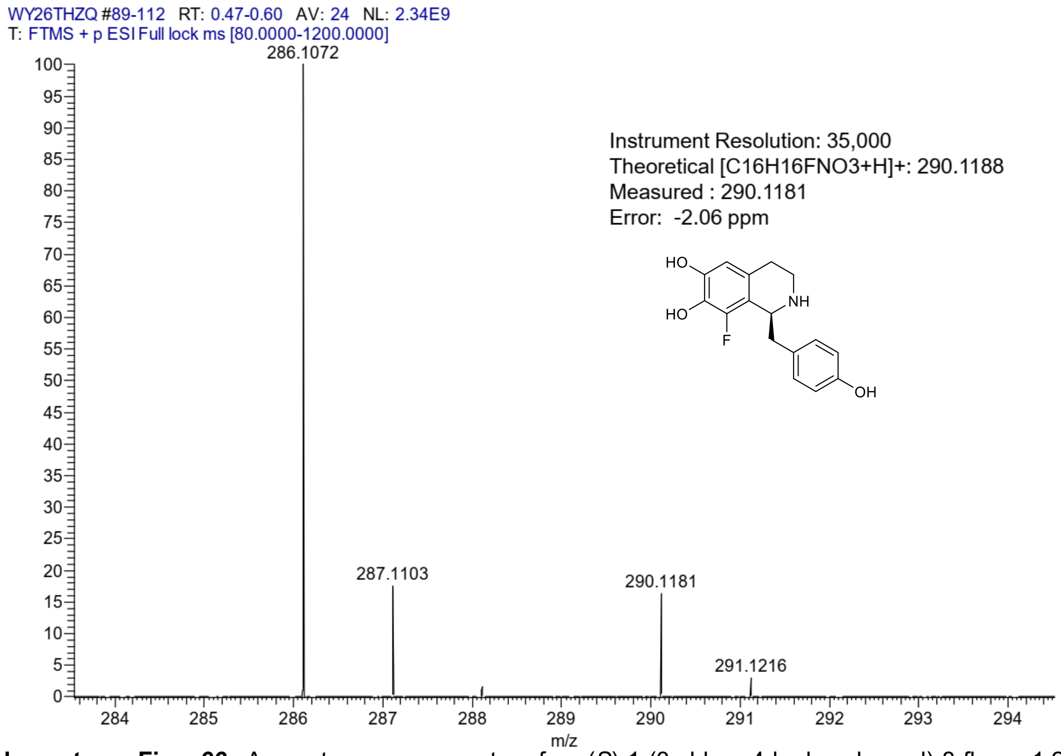

Supplementary Fig. 66 Accurate mass spectra for (*S*)-1-(3-chloro-4-hydroxybenzyl)-8-fluoro-1,2,3,4-tetrahydro-isoquinoline-6,7-diol (*S*)-**57**.

9 NMR spectroscopic data for cascade reaction products

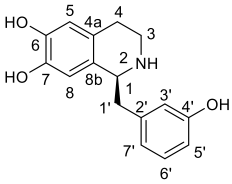

^1H NMR (600 MHz; CD_3OD)

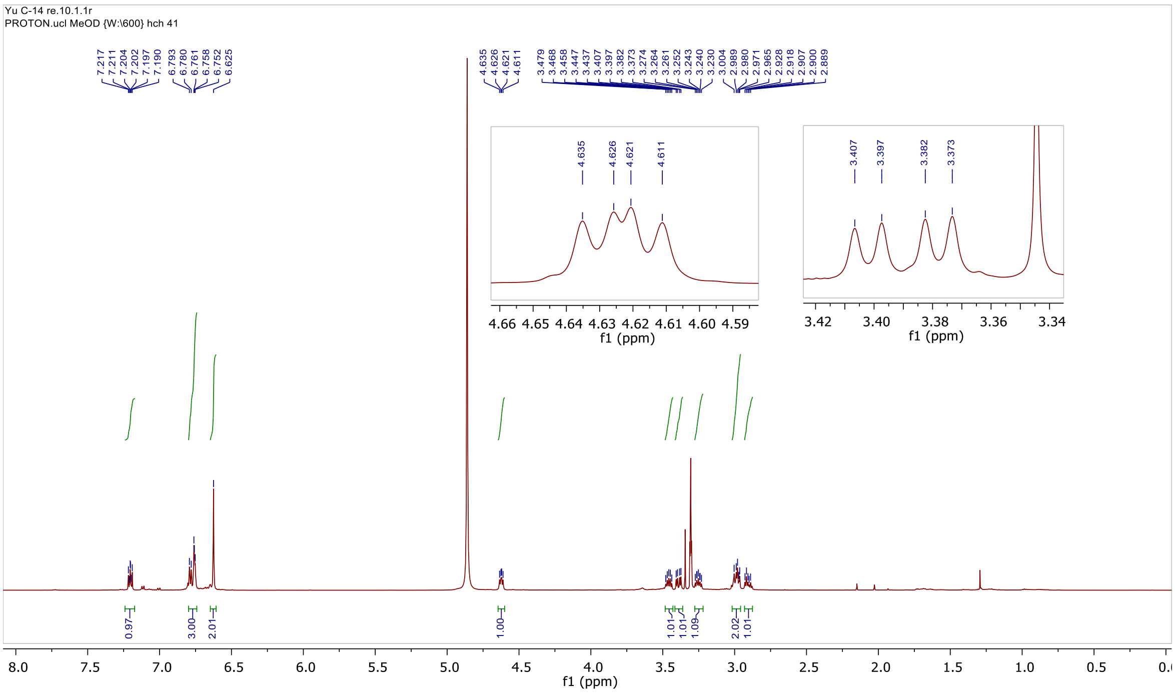

^{13}C NMR (150 MHz; CD_3OD)

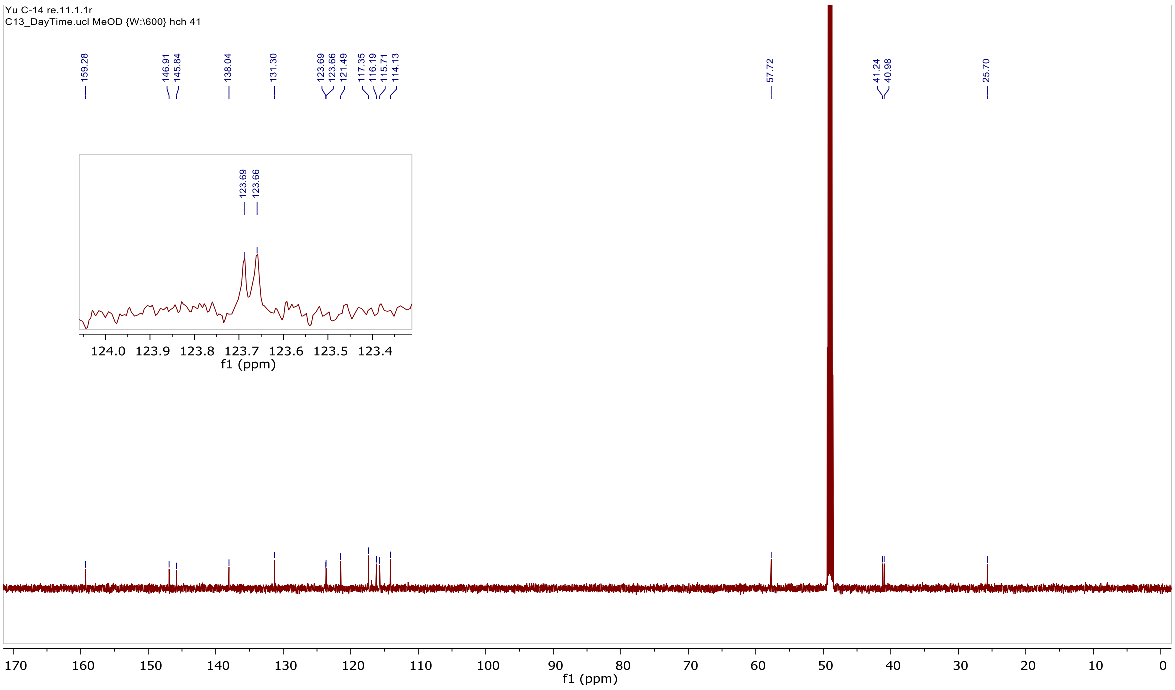

Supplementary Fig. 67 NMR spectra for (*S*)-1-(3-Hydroxybenzyl)-1,2,3,4-tetrahydroisoquinoline-6,7-diol (*S*)-**23**.

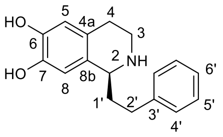

^{1}H NMR (700 MHz; CD_3OD)

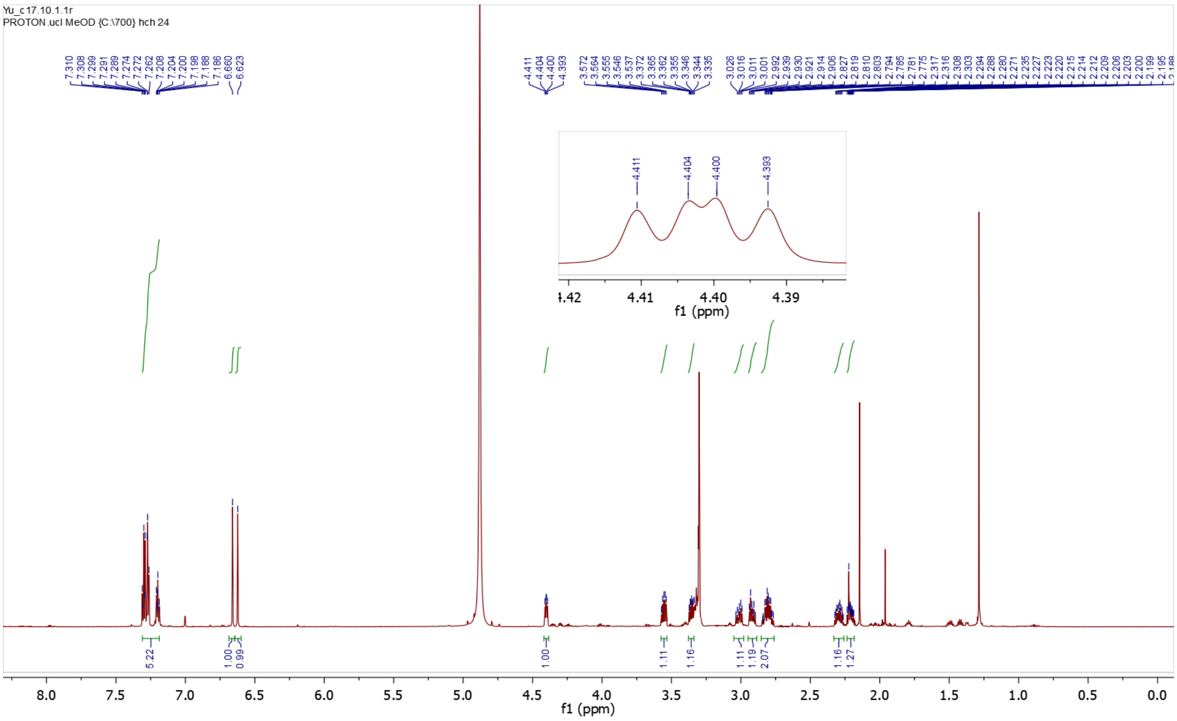

^{13}C NMR (175 MHz; CD_3OD)

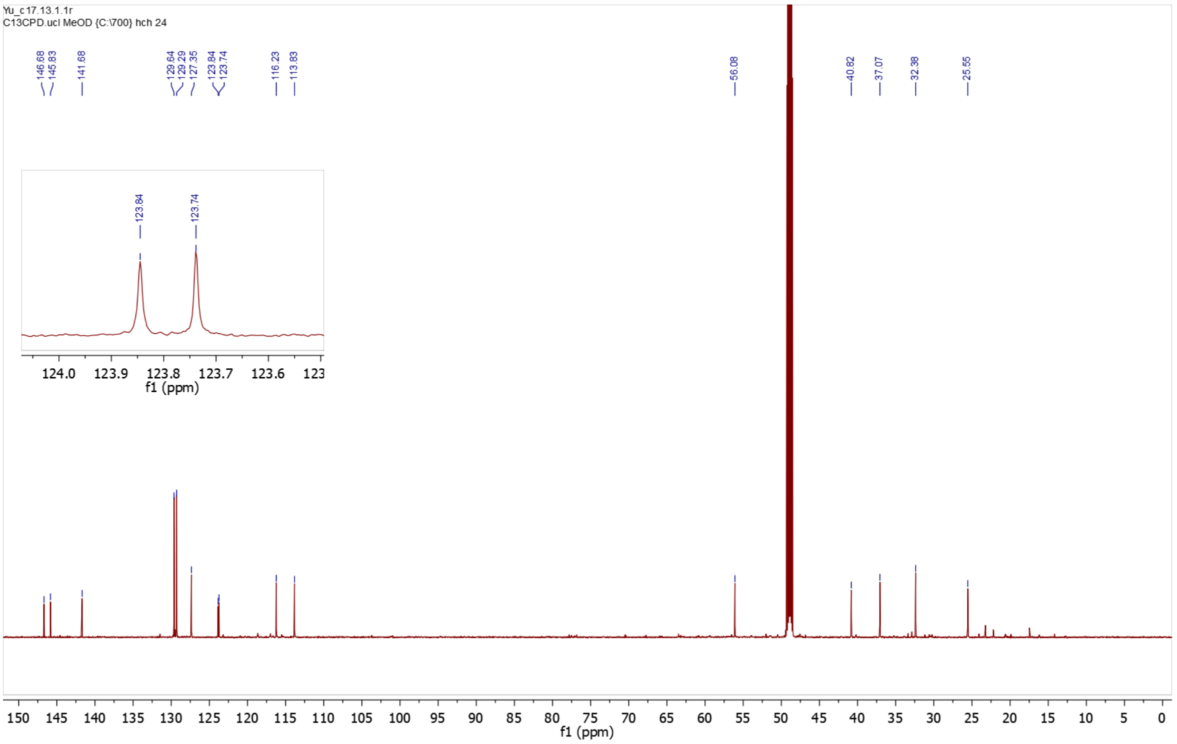

Supplementary Fig. 68 NMR spectra for (*S*)-1-Phenethyl-1,2,3,4-tetrahydroisoquinoline-6,7-diol (*S*)-**24**[1].

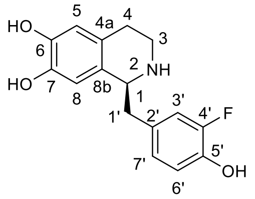

^{1}H NMR (600 MHz; CD_3OD)

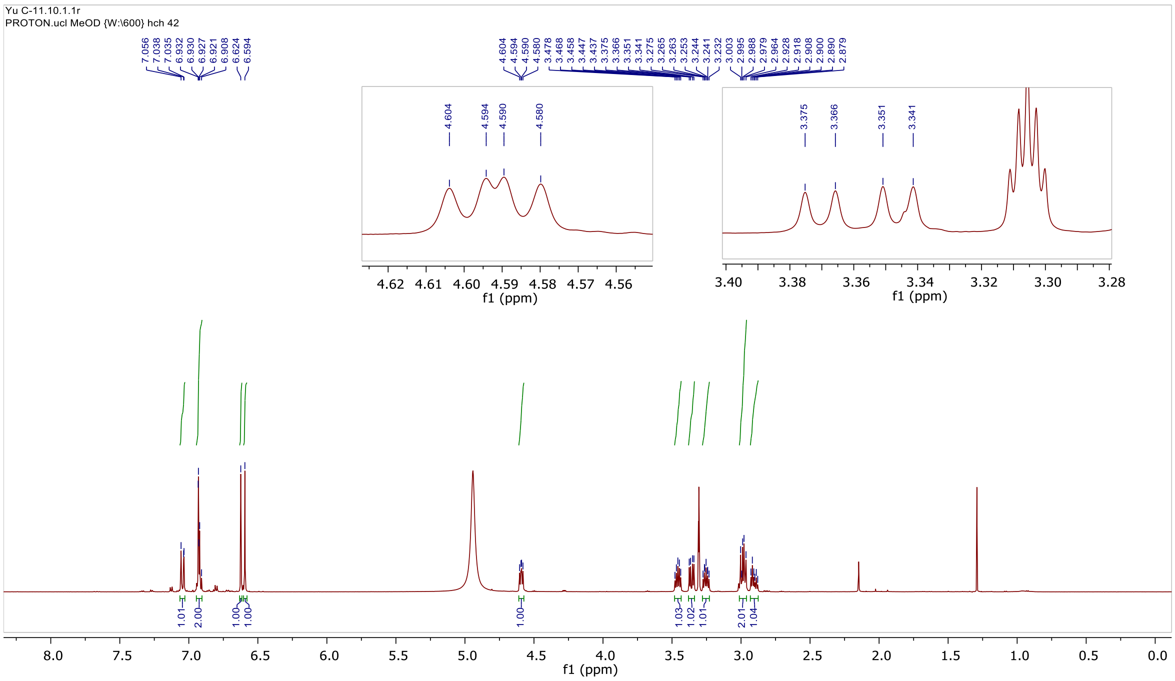

^{13}C NMR (150 MHz; CD_3OD)

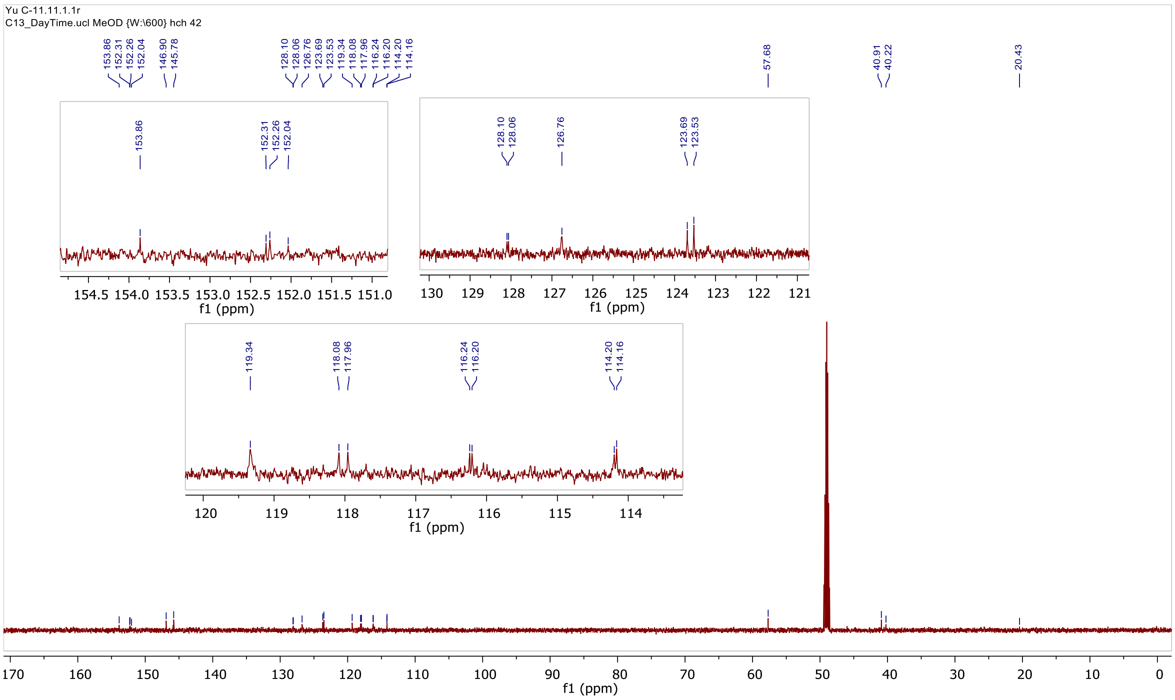

Supplementary Fig. 69 NMR spectra for (*S*)-1-(3-Fluoro-4-hydroxybenzyl)-1,2,3,4-tetrahydroisoquinoline-6,7-diol (*S*)-**25**.

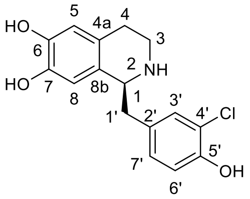

^{1}H NMR (600 MHz; CD_3OD)

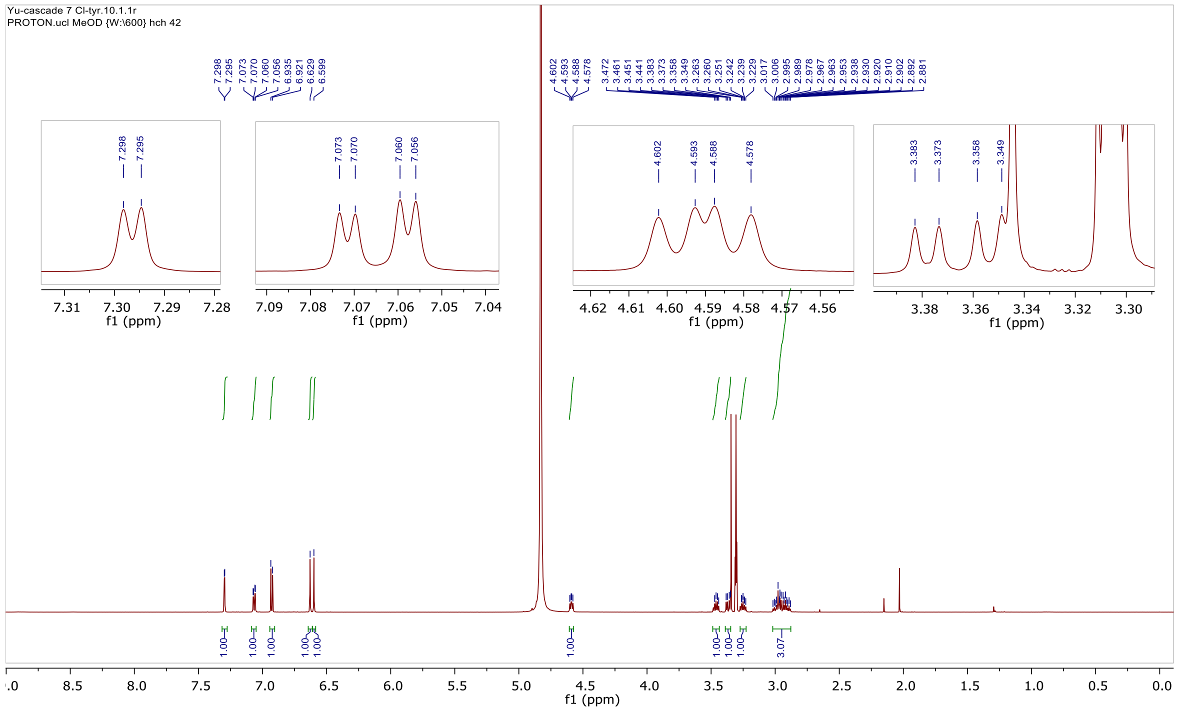

^{13}C NMR (150 MHz; CD_3OD)

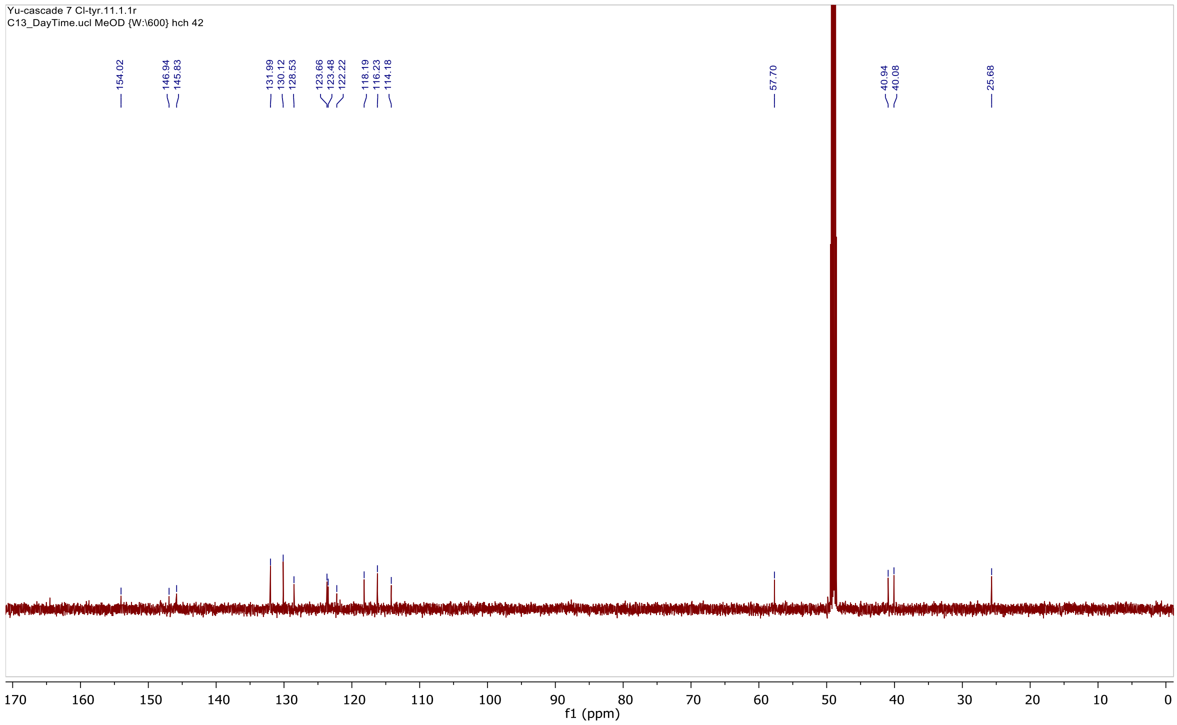

Supplementary Fig. 70 NMR spectra for (*S*)-1-(3-Chloro-4-hydroxybenzyl)-1,2,3,4-tetrahydroisoquinoline-6,7-diol (*S*)-**26**[2].

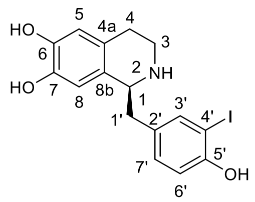

^{1}H NMR (600 MHz; CD_3OD)

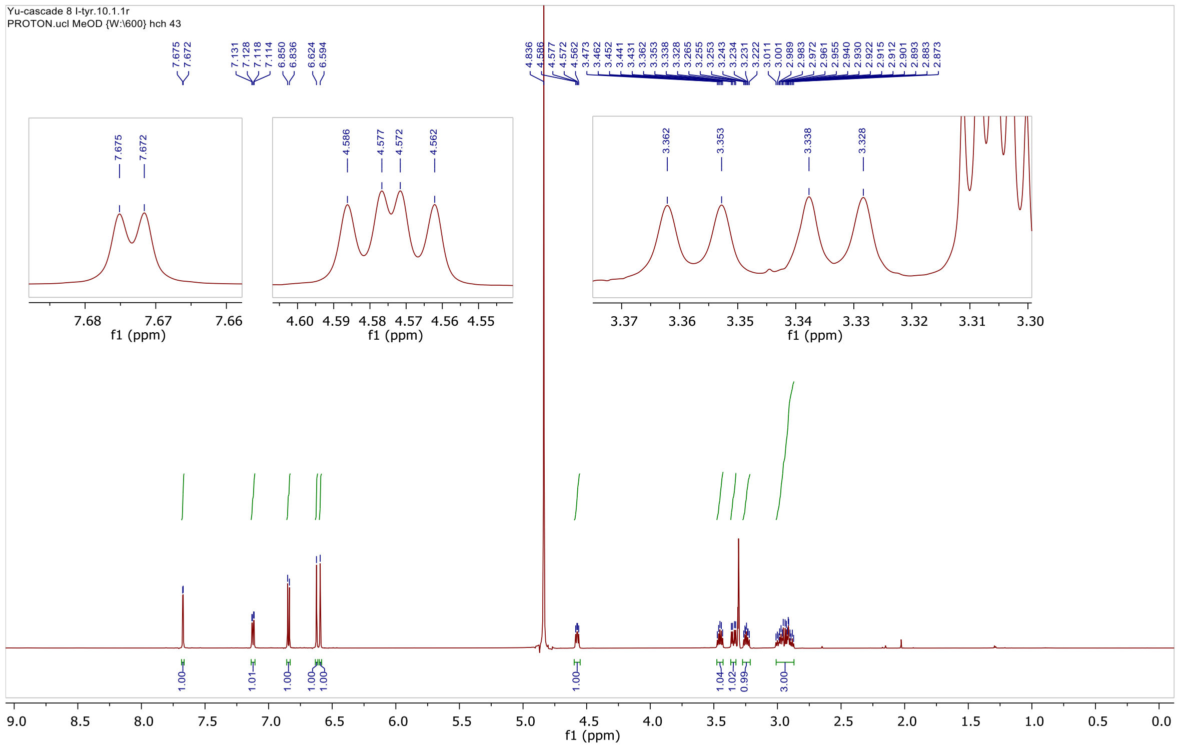

^{13}C NMR (150 MHz; CD_3OD)

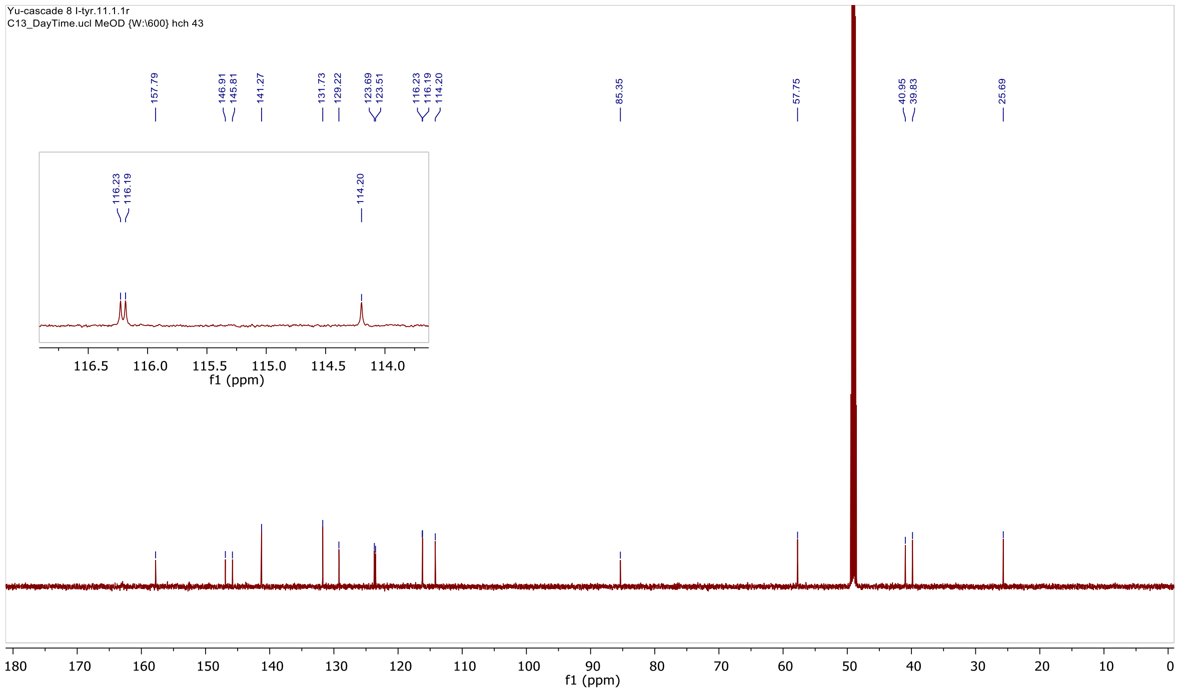

Supplementary Fig. 71 NMR spectra for (*S*)-1-(3-Iodo-4-hydroxybenzyl)-1,2,3,4-tetrahydroisoquinoline-6,7-diol (*S*)-**27**[2].

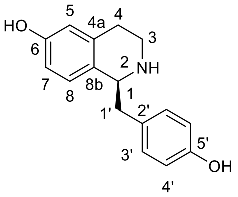

^{1}H NMR (600 MHz; CD_3OD)

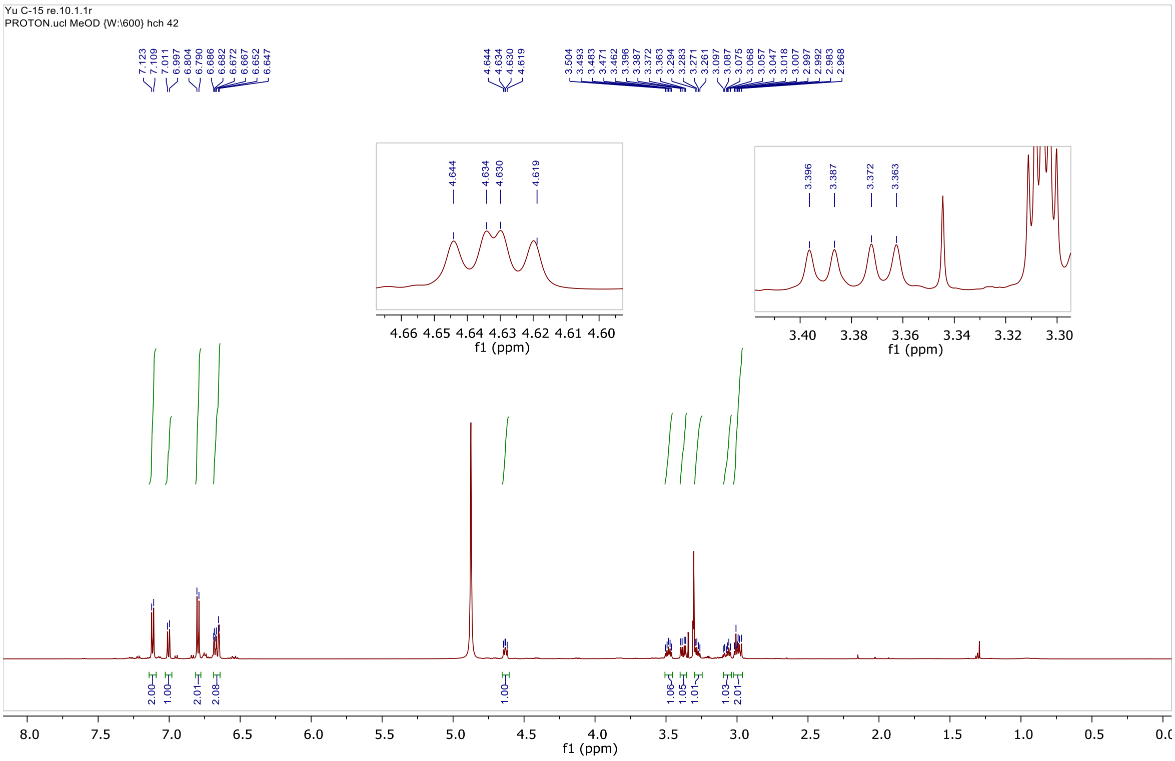

^{13}C NMR (150 MHz; CD_3OD)

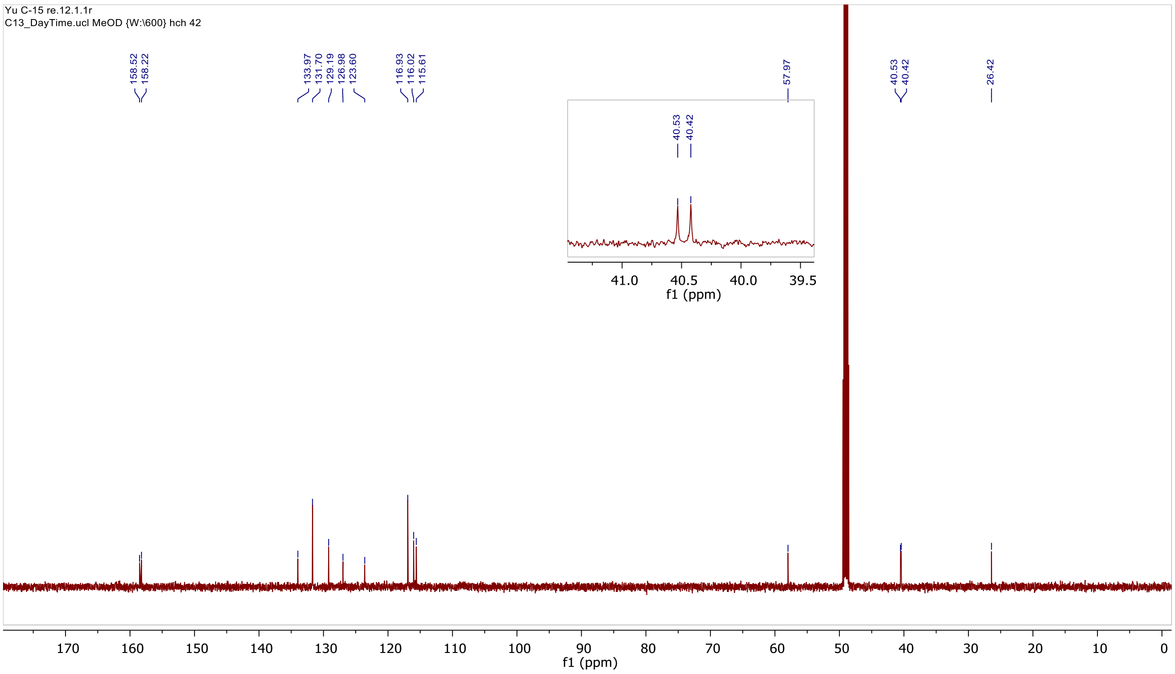

Supplementary Fig. 72 NMR spectra for (*S*)-1-(4-Hydroxybenzyl)-1,2,3,4-tetrahydroisoquinolin-6-ol (*S*)-**28**[3].

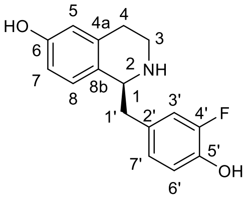

^{1}H NMR (600 MHz; CD_3OD)

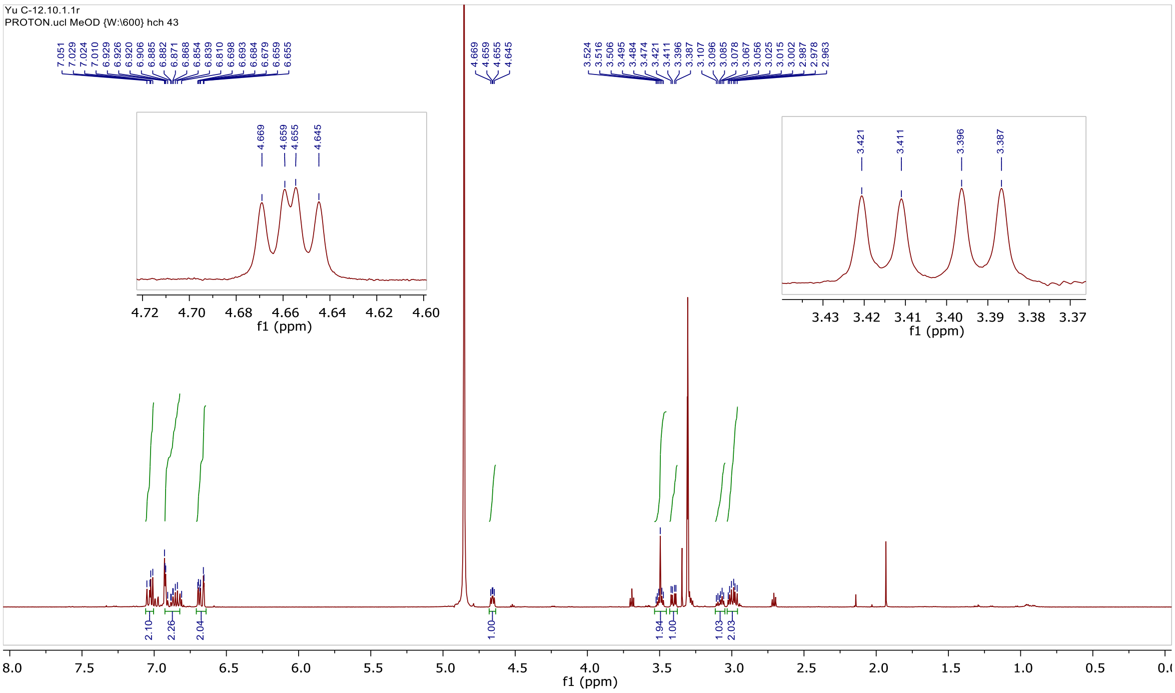

^{13}C NMR (150 MHz; CD_3OD)

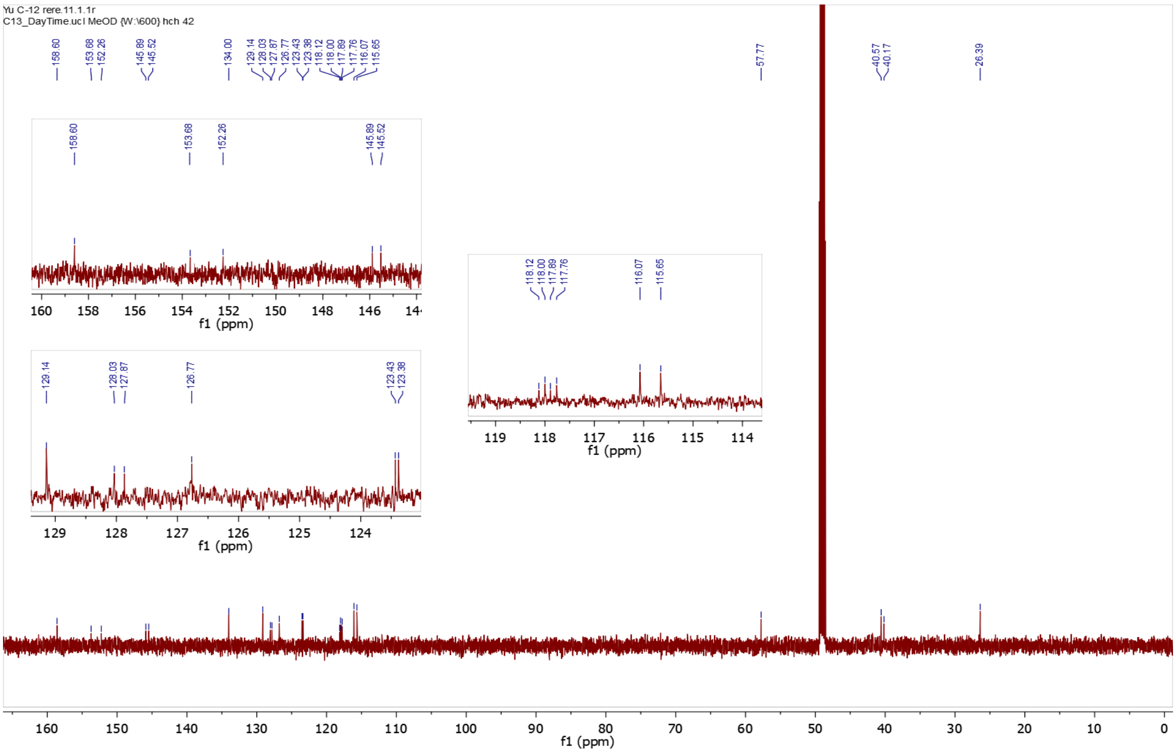

Supplementary Fig. 73 NMR spectra for (*S*)-1-(3-Fluoro-4-hydroxybenzyl)-1,2,3,4-tetrahydroisoquinolin-6-ol (*S*)-**29**.

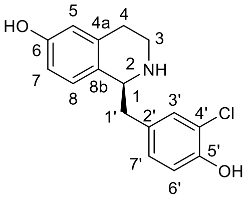

^{1}H NMR (600 MHz; CD_3OD)

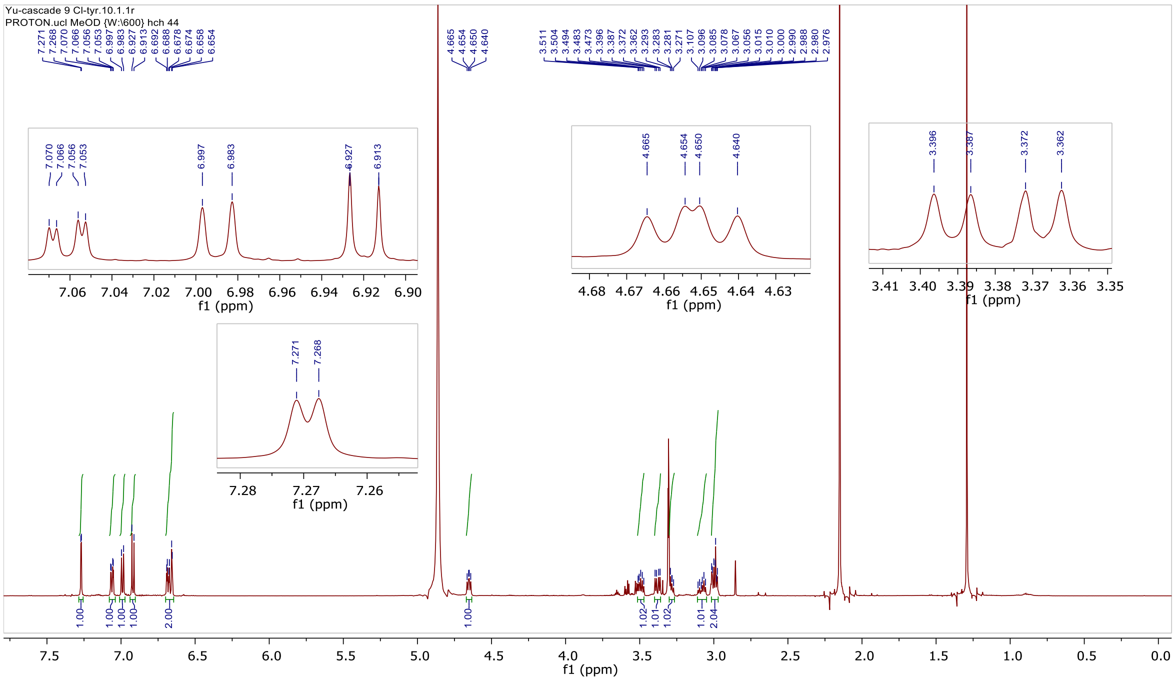

^{13}C NMR (150 MHz; CD_3OD)

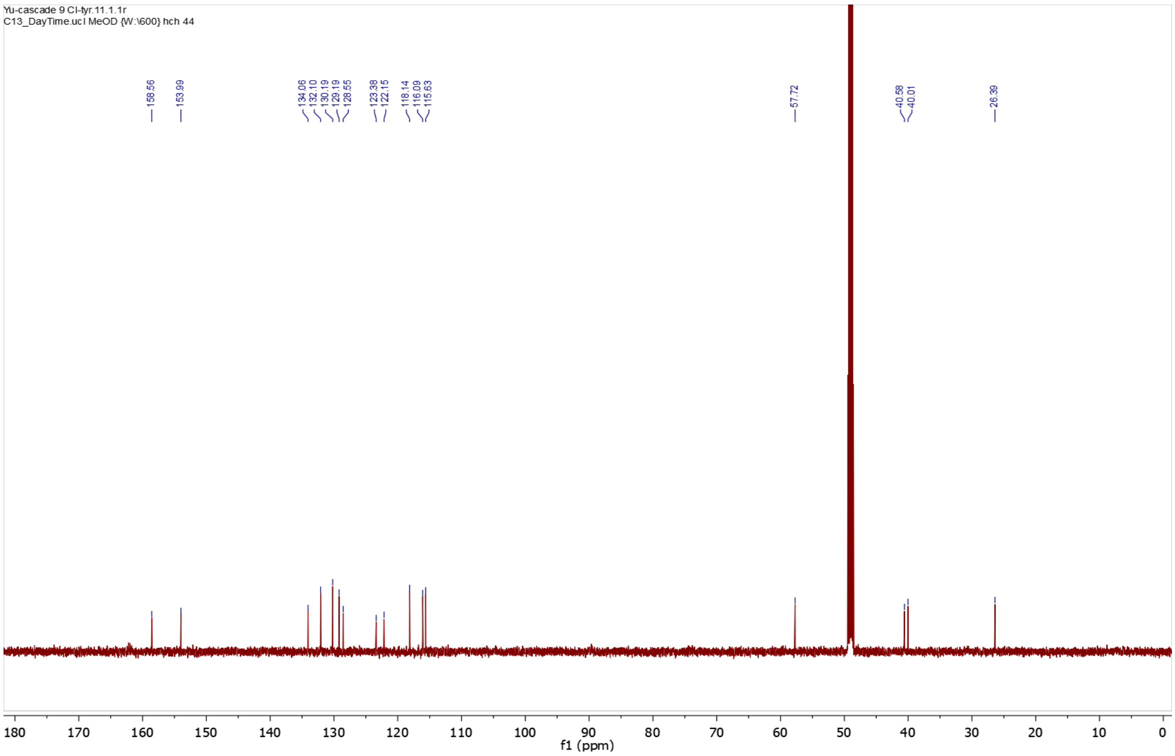

Supplementary Fig. 74 NMR spectra for (*S*)-1-(3-Chloro-4-hydroxybenzyl)-1,2,3,4-tetrahydroisoquinolin-6-ol (*S*)-**30**.

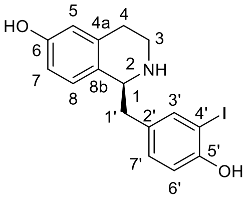

^{1}H NMR (600 MHz; CD_3OD)

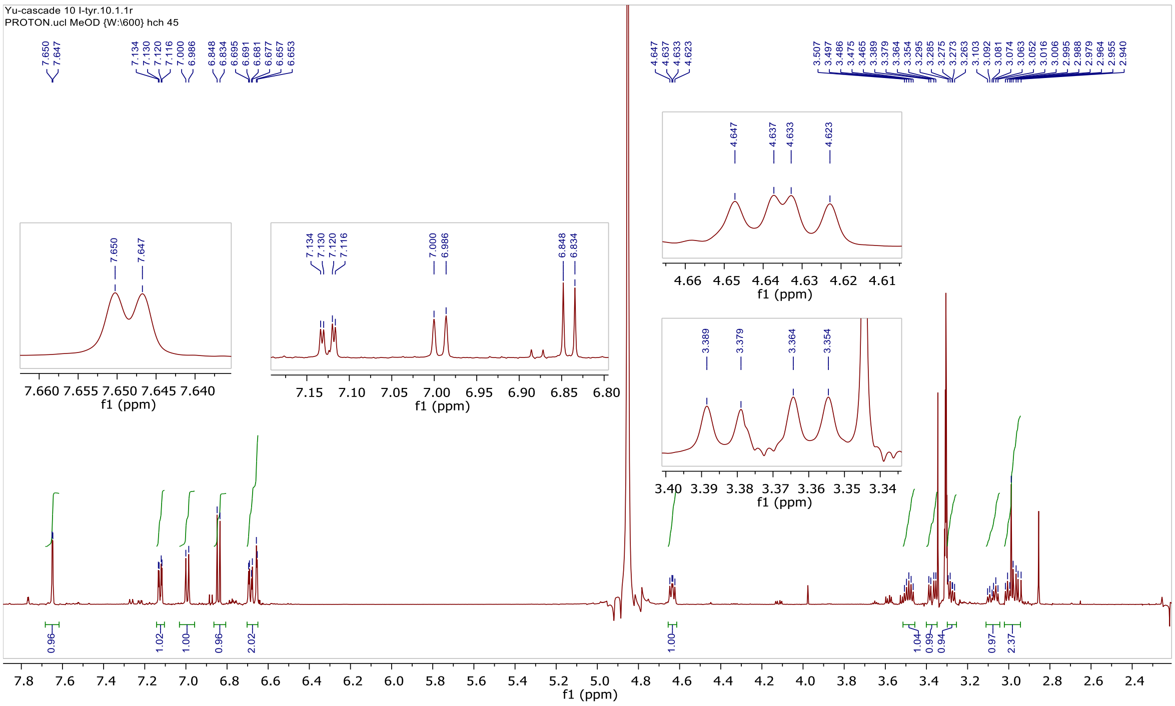

^{13}C NMR (150 MHz; CD_3OD)

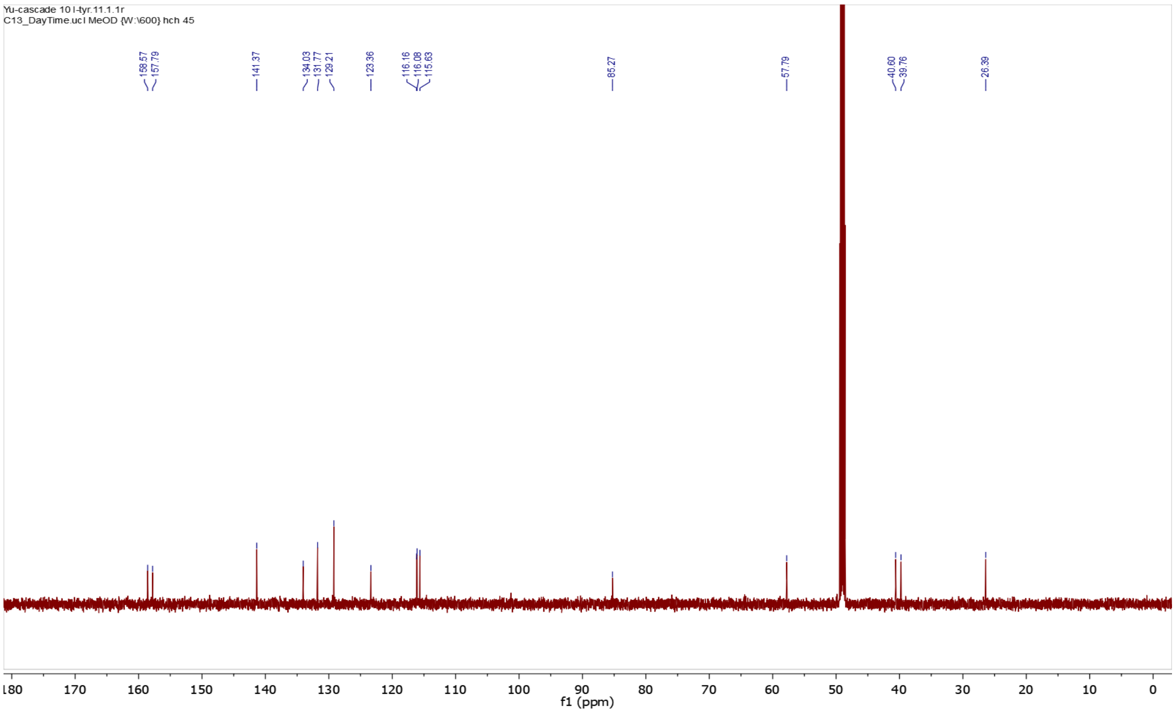

Supplementary Fig. 75 NMR spectra for (*S*)-1-(3-Iodo-4-hydroxybenzyl)-1,2,3,4-tetrahydroisoquinolin-6-ol (*S*)-**31**.

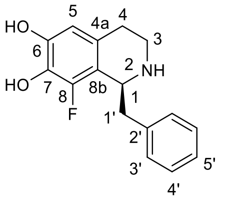

^{1}H NMR (600 MHz; CD_3OD)

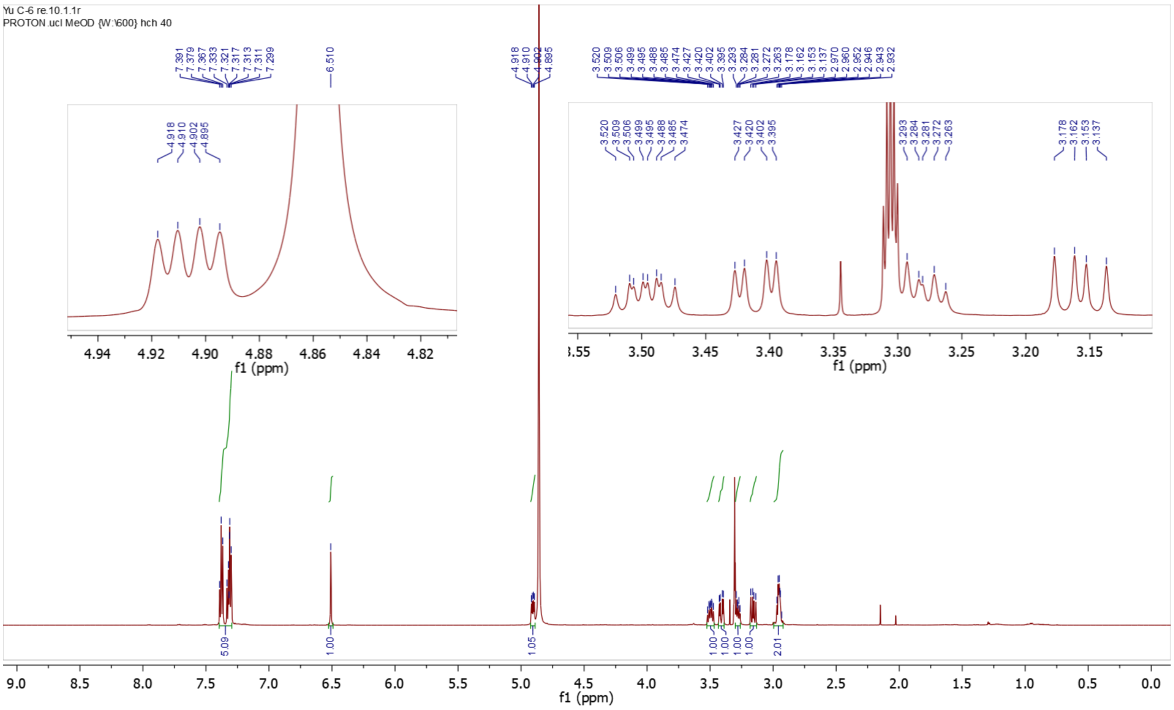

^{13}C NMR (150 MHz; CD_3OD)

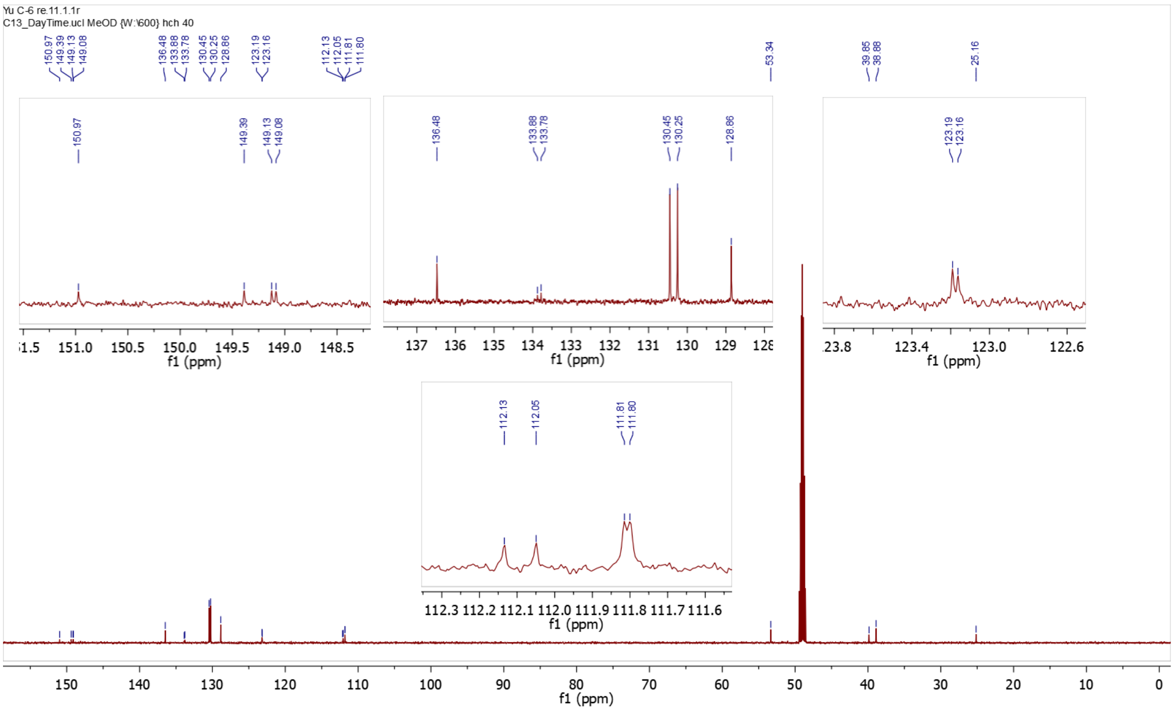

Supplementary Fig. 76 NMR spectra for (*S*)-1-Benzyl-8-fluoro-1,2,3,4-tetrahydroisoquinoline-6,7-diol (*S*)-**45**[4].

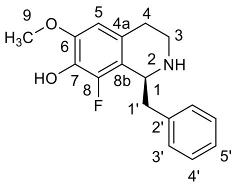

^{1}H NMR (700 MHz; CD_3OD)

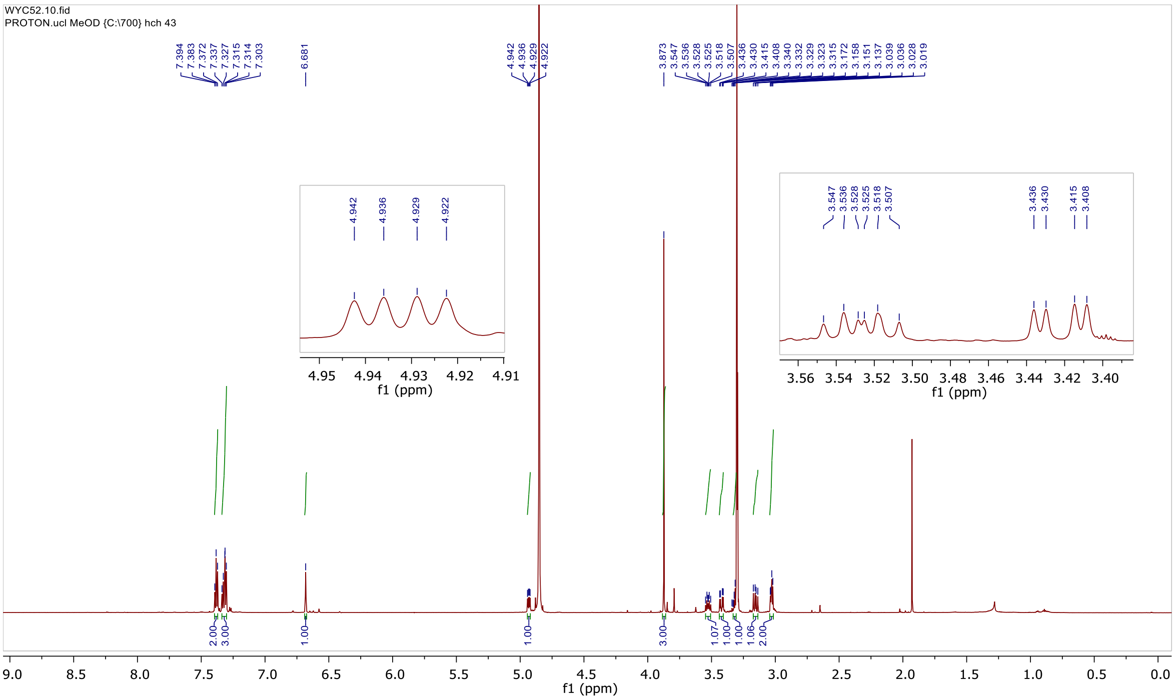

^{13}C NMR (175 MHz; CD_3OD)

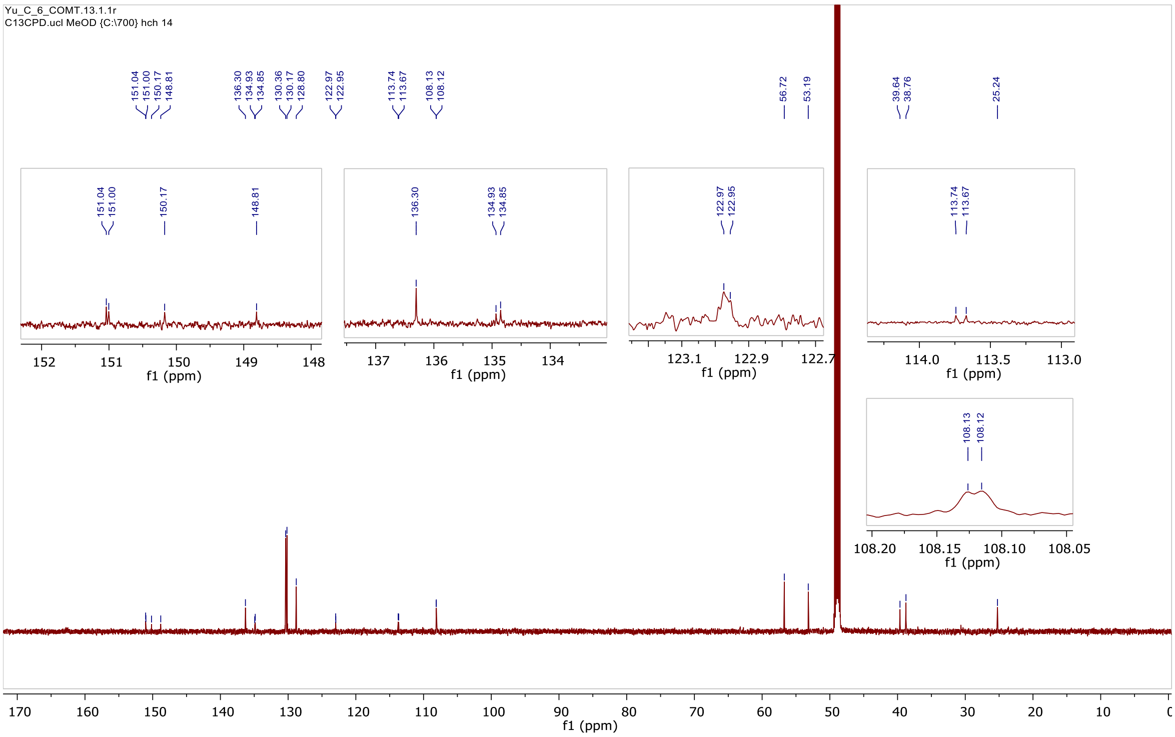

Supplementary Fig. 77 NMR spectra for (*S*)-1-Benzyl-8-fluoro-6-methoxy-1,2,3,4-tetrahydroisoquinolin-7-ol (*S*)-**46**[5].

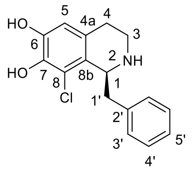

^{1}H NMR (700 MHz; CD_3OD)

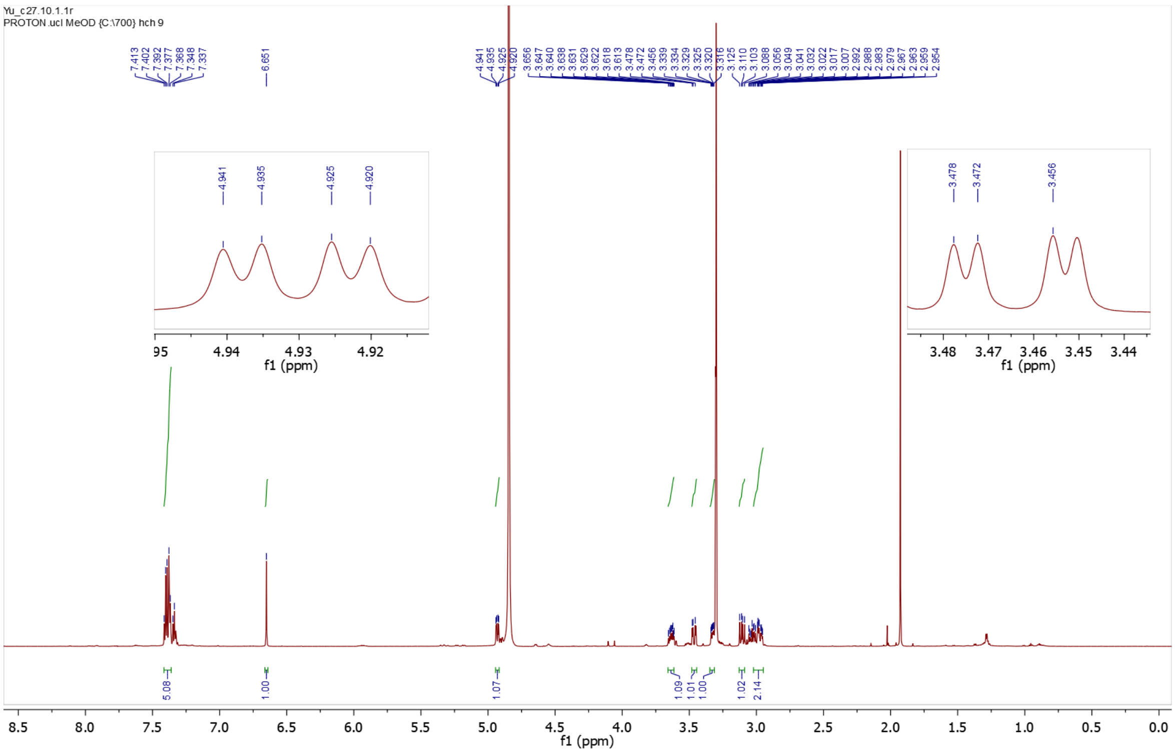

^{13}C NMR (175 MHz; CD_3OD)

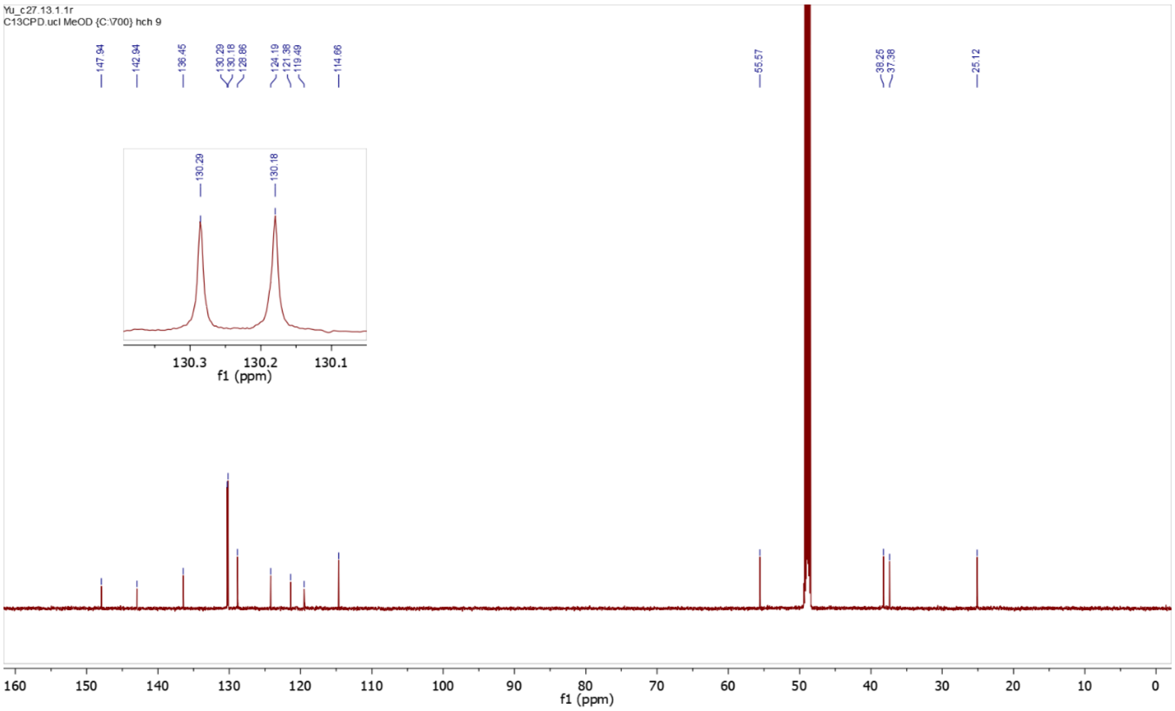

Supplementary Fig. 78 NMR spectra for (*S*)-1-Benzyl-8-chloro-1,2,3,4-tetrahydroisoquinoline-6,7-diol (*S*)-**48**.

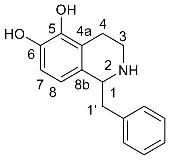

^{1}H NMR (700 MHz; CD_3OD)

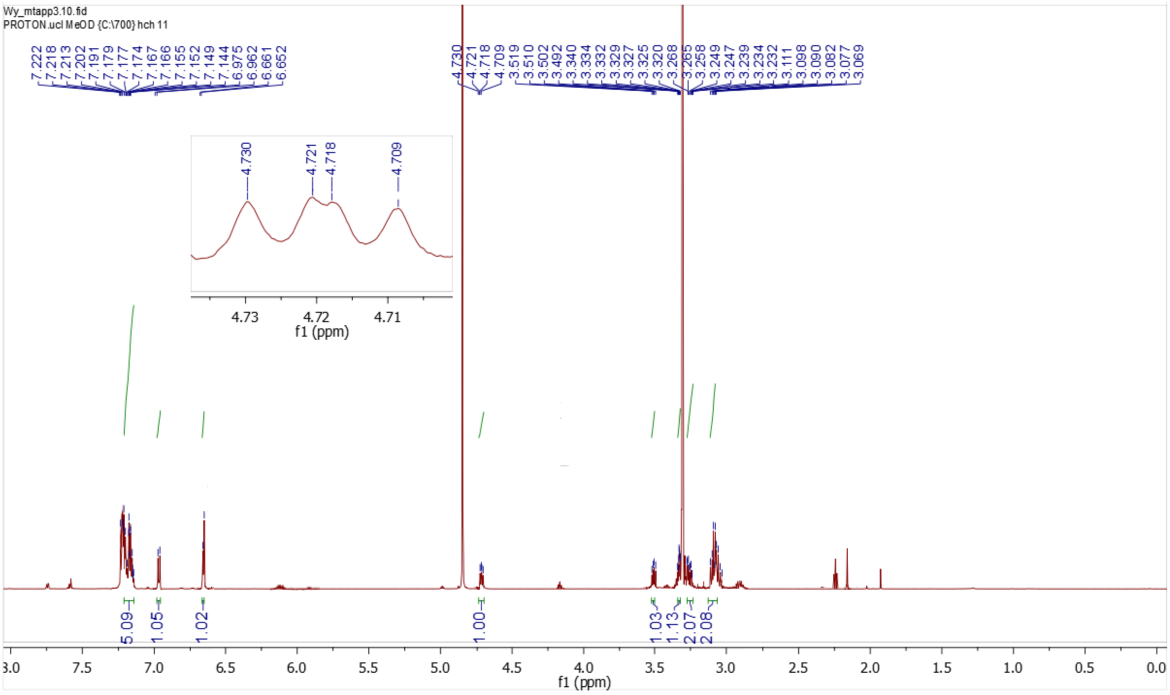

^{13}C NMR (175 MHz; CD_3OD)

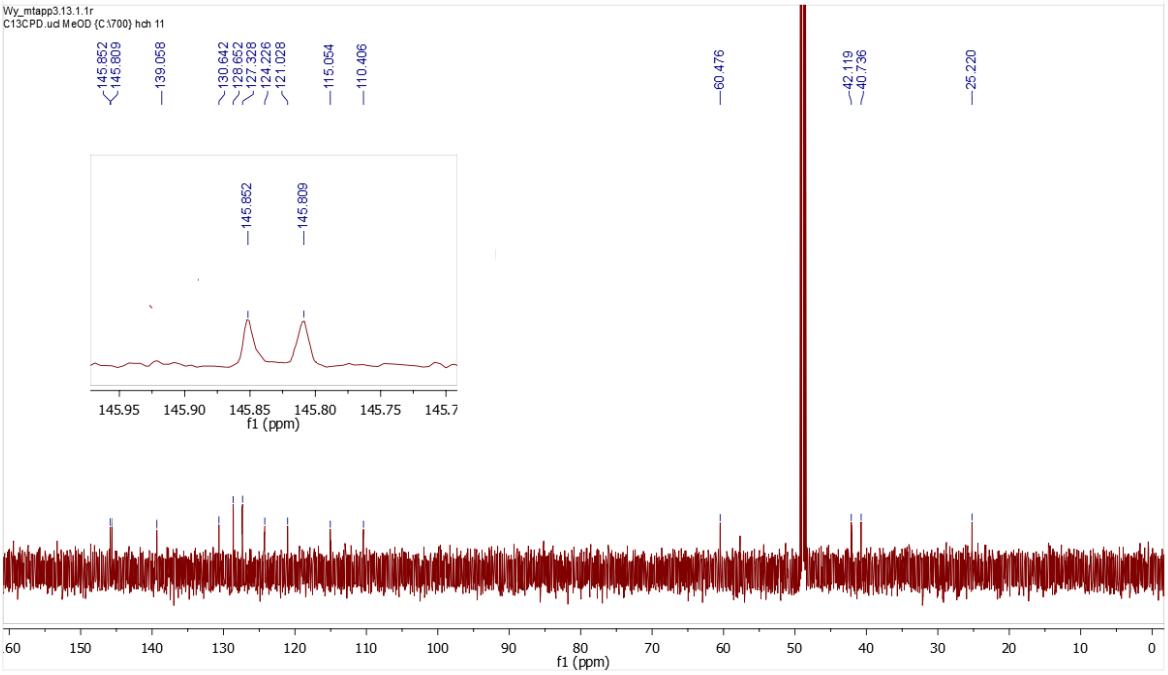

Supplementary Fig. 79 NMR spectra for (*S*)-1-Benzyl-1,2,3,4-tetrahydroisoquinoline-5,6-diol (*S*)-**49**.

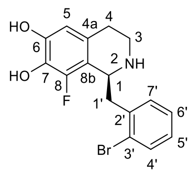

^{1}H NMR (700 MHz; CD_3OD)

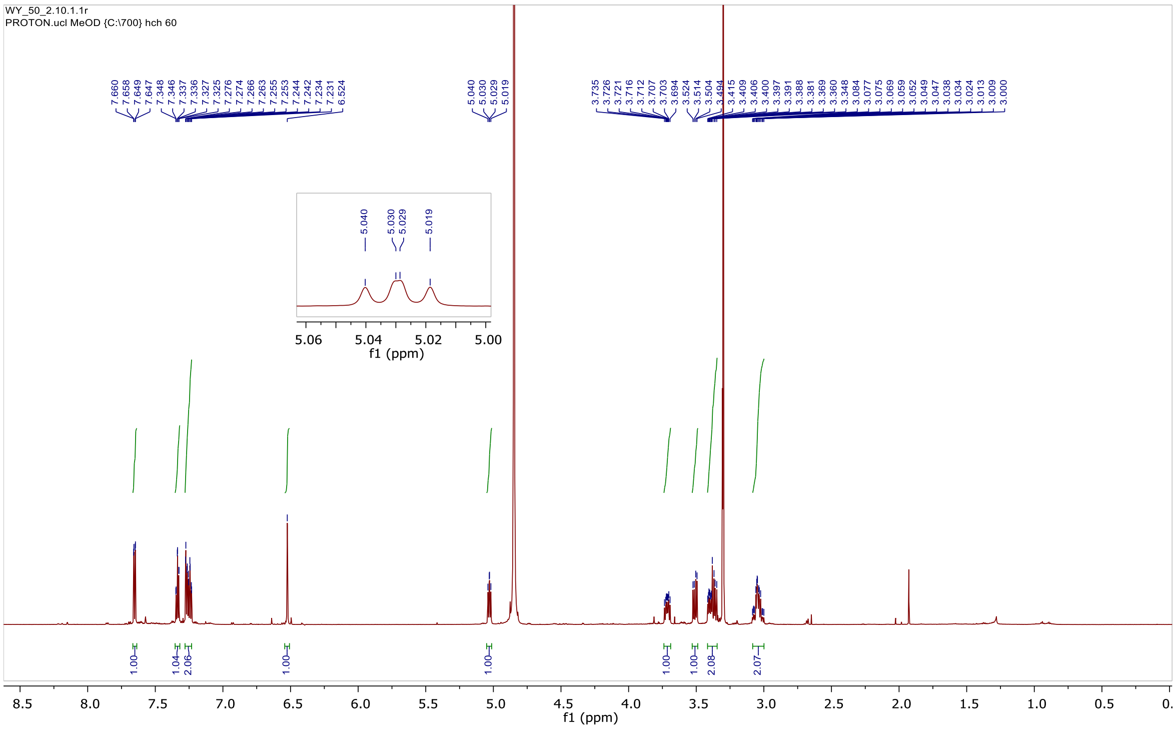

^{13}C NMR (175 MHz; CD_3OD)

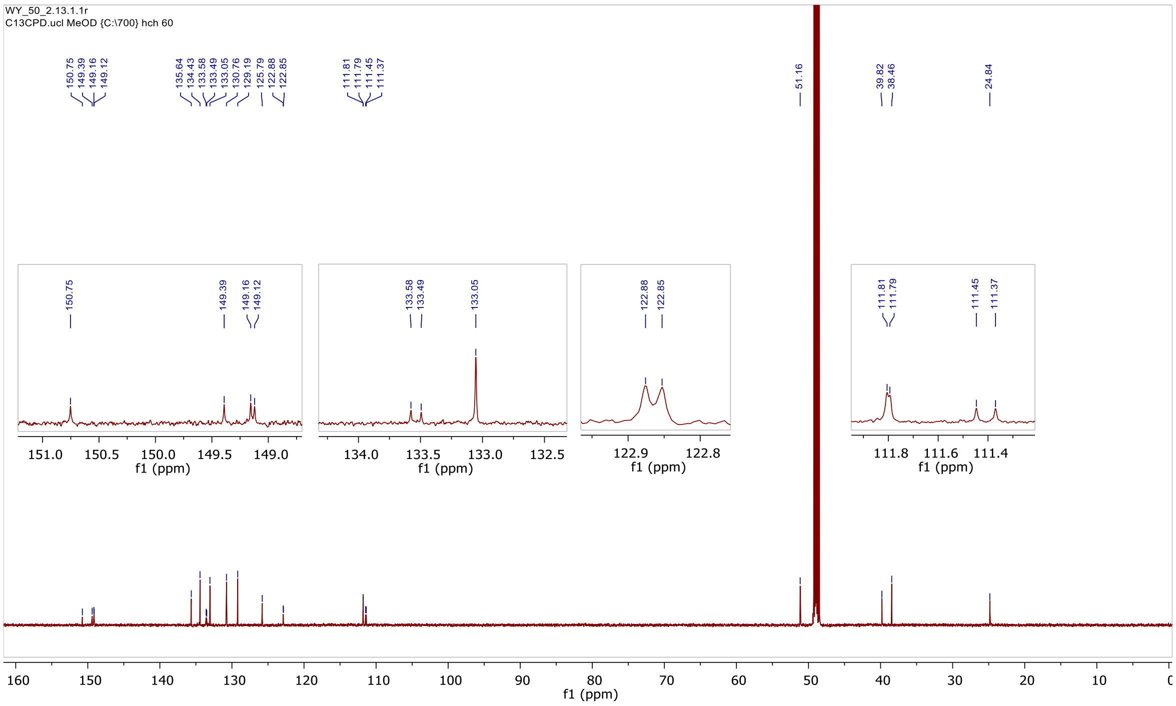

Supplementary Fig. 80 NMR spectra for (*S*)-1-(2-Bromobenzyl)-8-fluoro-1,2,3,4-tetrahydroisoquinoline-6,7-diol (*S*)-**51**.

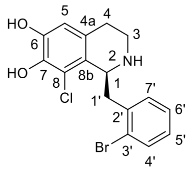

^{1}H NMR (700 MHz; CD_3OD)

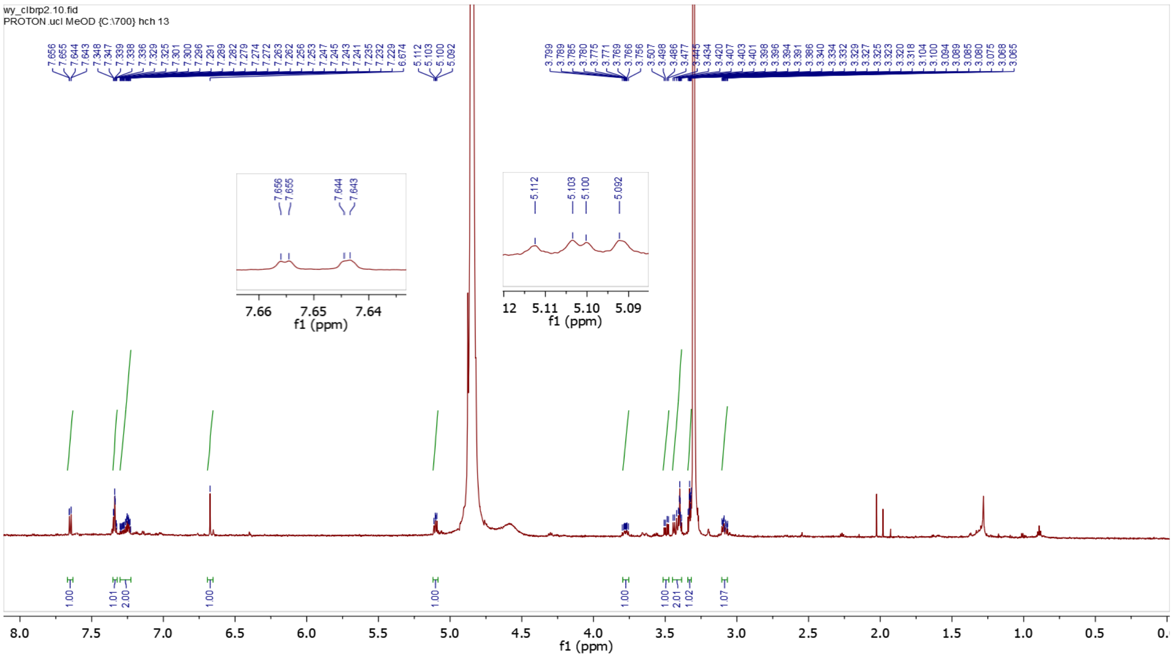

^{13}C NMR (175 MHz; CD_3OD)

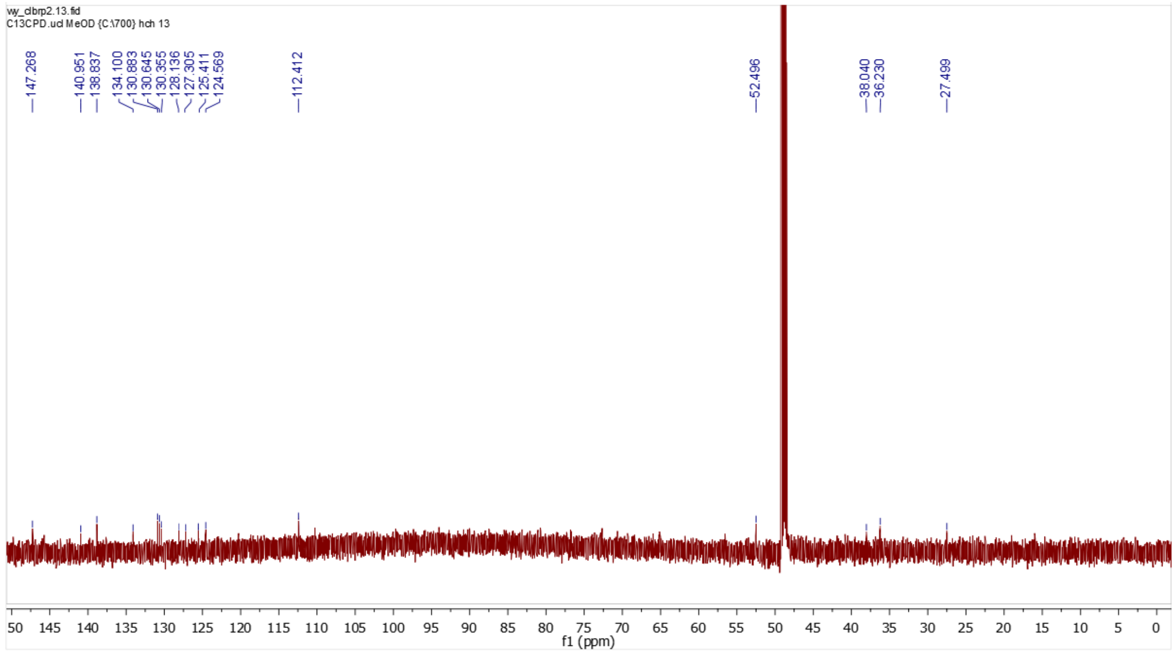

Supplementary Fig. 81 NMR spectra for (*S*)-1-(2-Bromobenzyl)-8-chloro-1,2,3,4-tetrahydroisoquinoline-6,7-diol (*S*)-**52**.

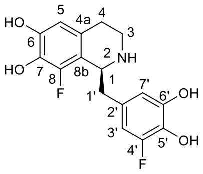

^{1}H NMR (600 MHz; CD_3OD)

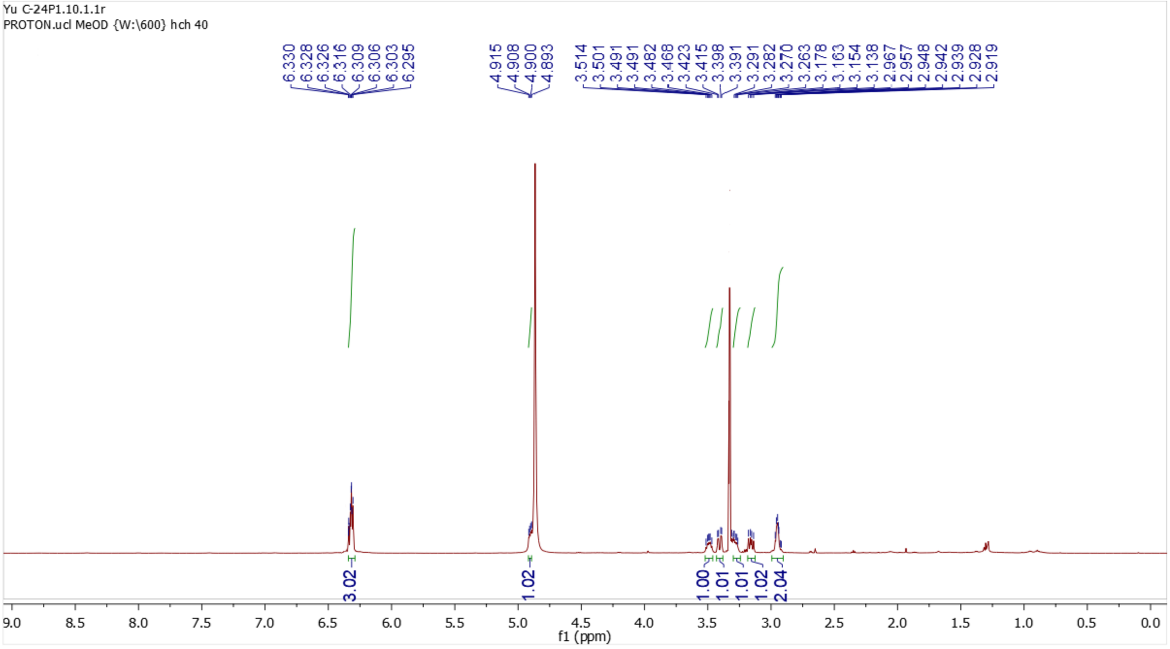

^{13}C NMR (150 MHz; CD_3OD)

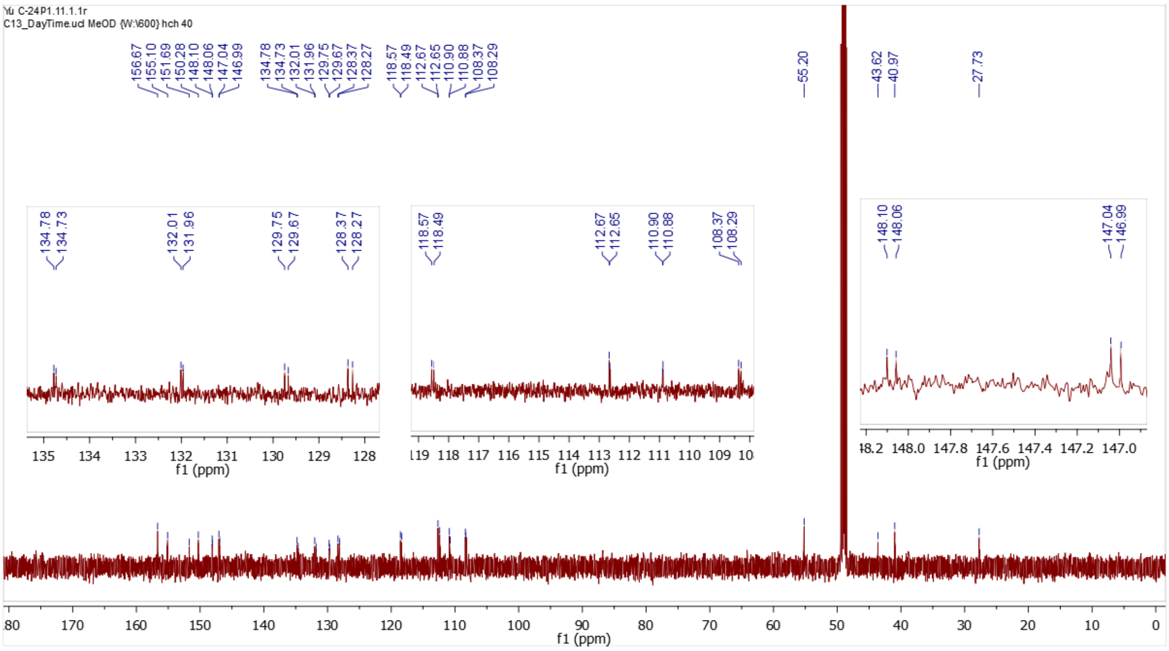

Supplementary Fig. 82 NMR spectra for (*S*)-8-Fluoro-1-(3-fluoro-4,5-dihydroxybenzyl)-1,2,3,4-tetrahydroisoquinoline-6,7-diol (*S*)-**53**.

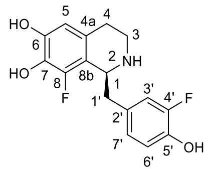

^{1}H NMR (600 MHz; CD_3OD)

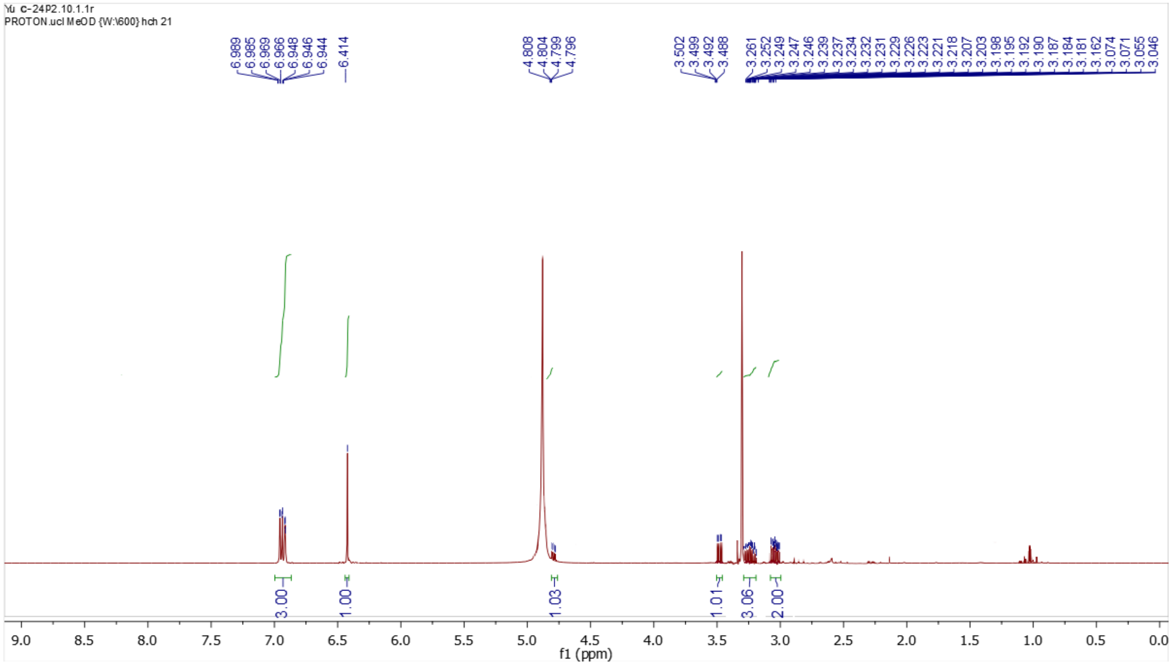

^{13}C NMR (150 MHz; CD_3OD)

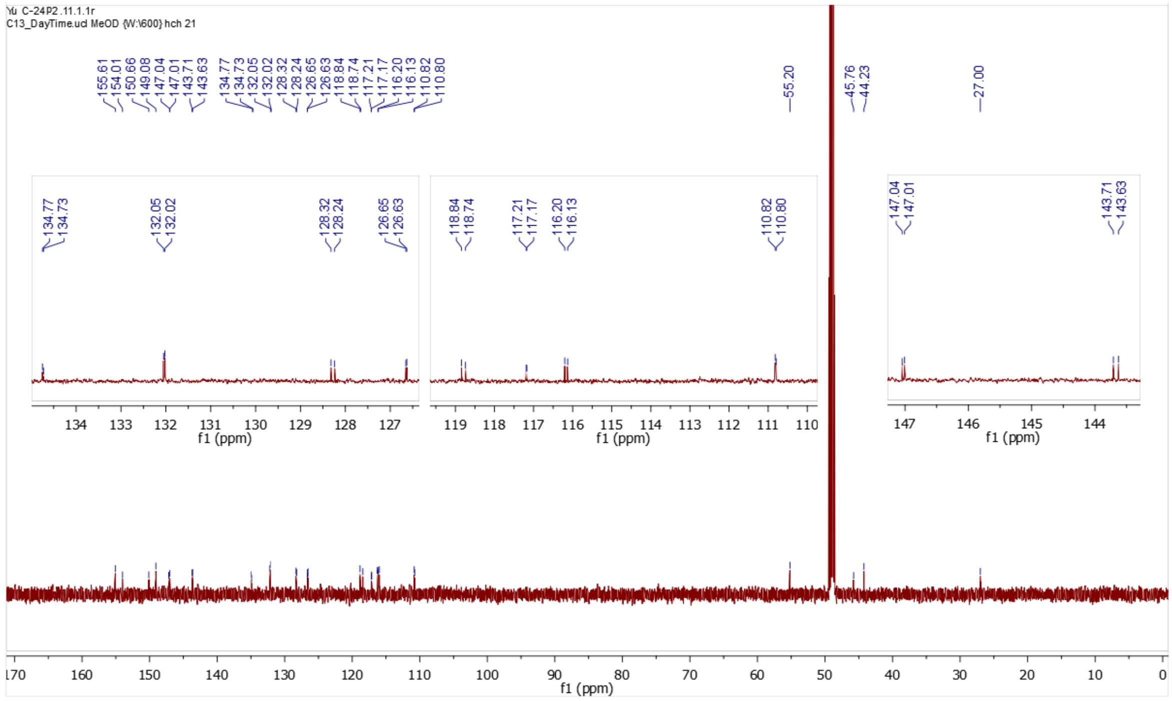

Supplementary Fig. 83 NMR spectra for (*S*)-8-Fluoro-1-(3-fluoro-4-hydroxybenzyl)-1,2,3,4-tetrahydroisoquinoline-6,7-diol (*S*)-**54**.

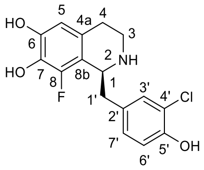

^{1}H NMR (700 MHz; CD_3OD)

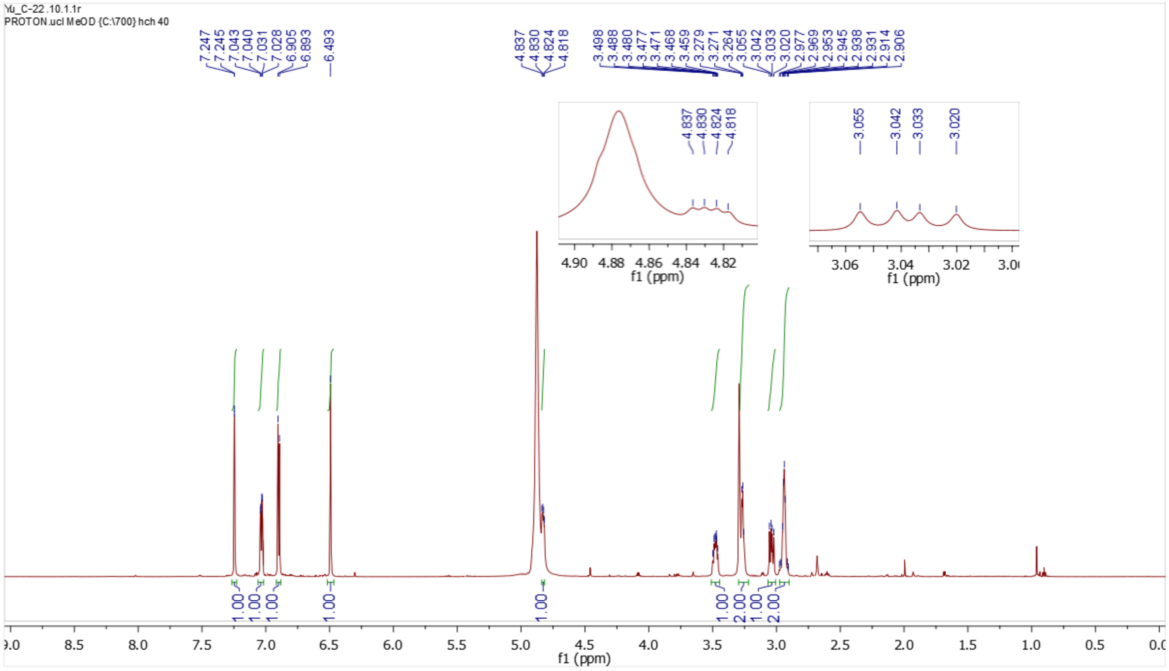

^{13}C NMR (175 MHz; CD_3OD)

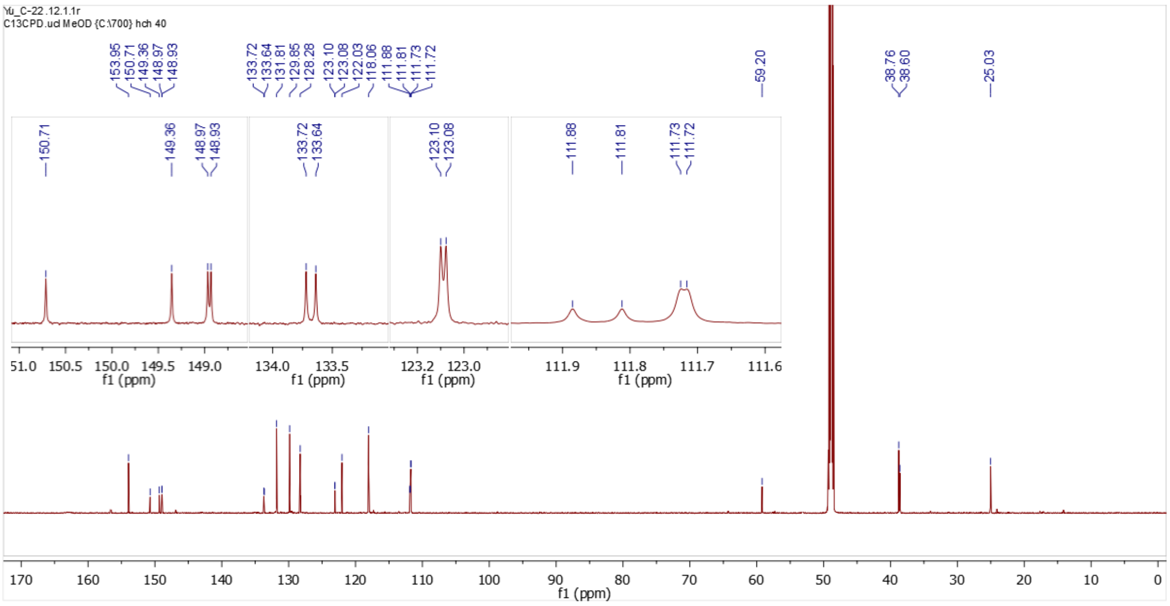

Supplementary Fig. 84 NMR spectra for (*S*)-1-(3-Chloro-4-hydroxybenzyl)-8-fluoro-1,2,3,4-tetrahydroisoquinoline-6,7-diol (*S*)-**55**.

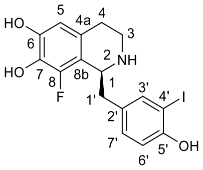

^{1}H NMR (700 MHz; CD_3OD)

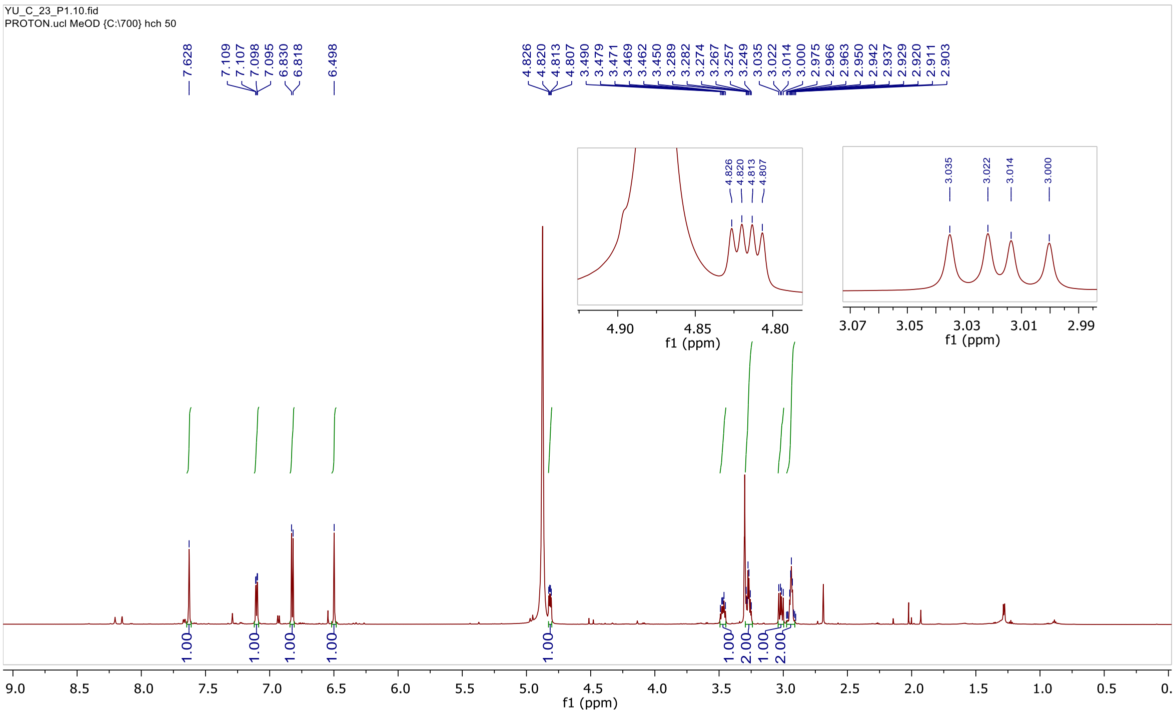

^{13}C NMR (175 MHz; CD_3OD)

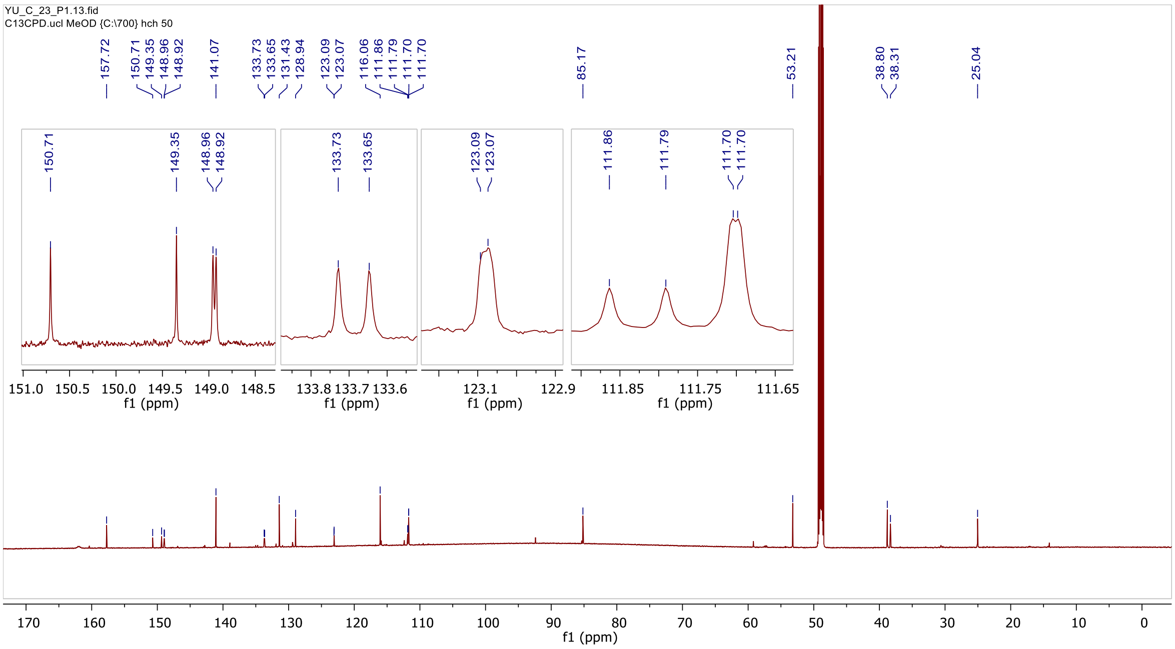

Supplementary Fig. 85 NMR spectra for (*S*)-1-(3-Iodo-4-hydroxybenzyl)-8-fluoro-1,2,3,4-tetrahydroisoquinoline-6,7-diol (*S*)-**56**.

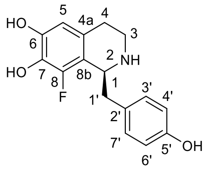

^{1}H NMR (700 MHz; CD_3OD)

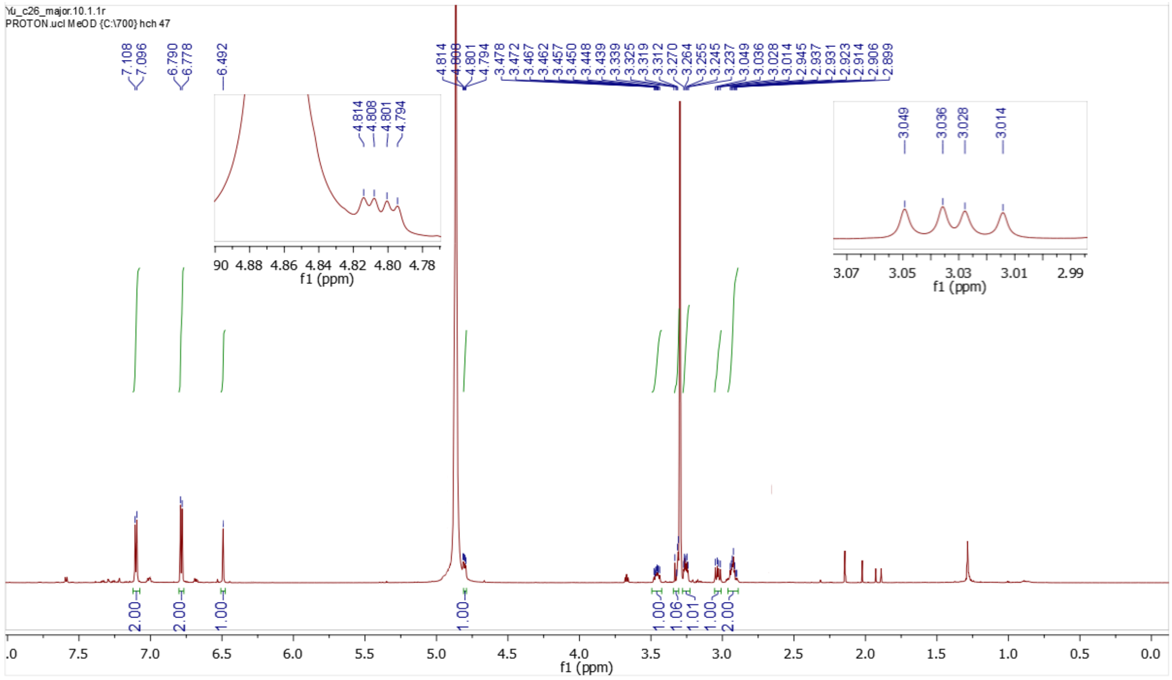

^{13}C NMR (175 MHz; CD_3OD)

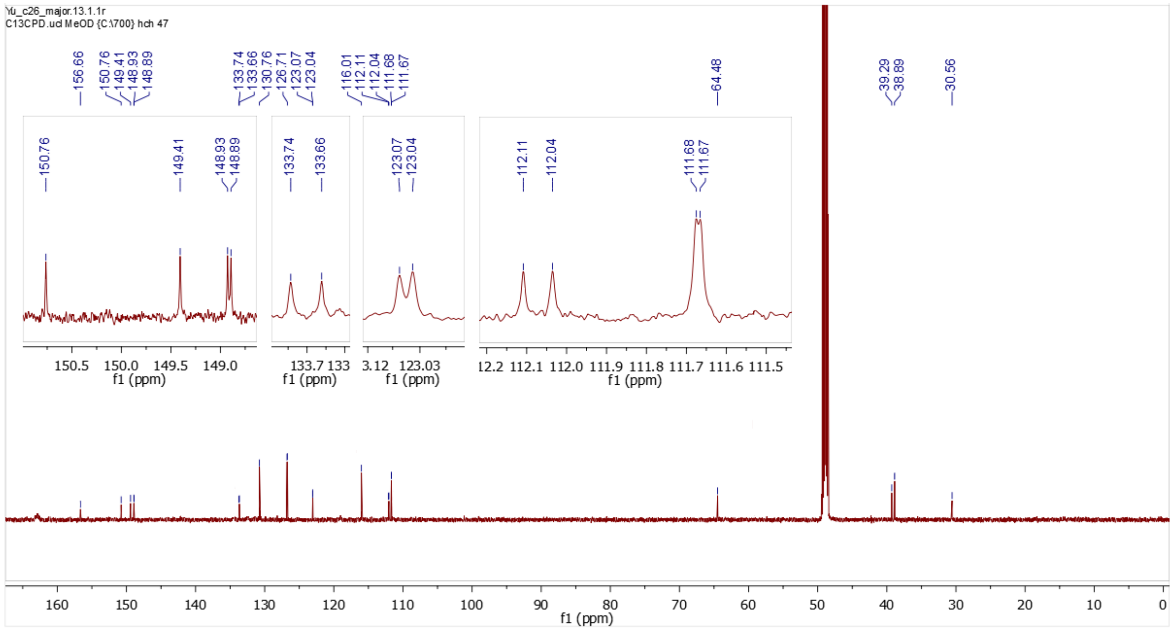

Supplementary Fig. 86 NMR spectra for (*S*)-8-Fluoro-1-(4-hydroxybenzyl)-1,2,3,4-tetrahydroisoquinoline-6,7-diol (*S*)-**57**.

10 Global docking via AutoDock Vina

10.1 Preparing your protein

Using Chimera (UCFS Chimera version 1.13.1), the protein PDB was loaded and any water molecules and other subunits deleted. Selenomethionine residues were mutated to methionine and selenocysteine to cysteine. The protein was then saved as a pdb file. The protein pdb file was opened using AutoDock Tools (version 1.5.6) and hydrogens added to the protein. The box size for ligand docking was set to cover the whole protein (to encompass the active site, but not bias the results) and saved as a pdbqt file.

10.2 Preparing the ligand

ChemDraw smiles were generated and the energy of the ligand minimised using Avogadro (version 1.2.0). Again, the ligand pdb file was converted into a pdbqt file via AutoDock Tools.

10.3 Using Autodock Vina (v.1.2.0)

A text file was created input all the parameters for docking:

```
receptor = protein file name.pdbqt
ligand = ligand file name.pdbqt
out = out.pdbqt
center_x = 21.023
center_y = 11.027
center_z = 11.504
size_x = 40
size_y = 40
size_z = 40
```

Center_x,y and z and the size parameters were for the docking box - the values that were recorded from autodock tools. ‘out = out.pdbqt’ stored output files (different conformations and positions of the ligand). This was saved as the receptor and ligand pdbqt files. Then docking was performed via Vina through a terminal window. Vina was run and the binding modes results and energies were saved in a log file (log.txt).

10.4 Viewing the results

Docking results (binding modes) were viewed using Chimera by loading in the receptor pdb file and then out.pdb (all the output files). Nine possible binding modes were generated and ranked according to the affinity free energy. The scoring function used in Vina was derived using the PDBbind data set, in which the receptors were treated as rigid components, and the ligands as flexible molecules with the number of active rotatable bonds ranging from 0 to 32. Vina uses a gradient optimization method in its local optimization procedure. The calculation of the gradient gives the optimization algorithm a ”sense of direction” from a single evaluation. By using multithreading, Vina can further speed up the execution by taking advantage of multiple CPUs or CPU cores.[7]

Supplementary Table 1 Global docking analysis for *Cn*TYR mutants with compounds **4, 9, 11** and **12.**

Enzyme	Affinity (kcal/mol)	Ranking[b]	In catalytic pot	Orentation state[c]	Distance 1[d]	Distance 2[e]
Modelled Wild-type *Cn*TYR[a] (with L-tyrosine **4**)	-6.3	1	In	Yes	2.45 Å	3.92 Å
	-5.5	2	In	No	10.24 Å	-
	-5.3	3	In	Yes	2.20 Å	4.15 Å
	-5.0	4	Out	-	-	-
	-4.9	5	In	No	9.86 Å	-
	-4.8	6	In	No	4.86 Å	-
	-4.8	7	Out	-	-	-
	-4.7	8	Out	-	-	-
	-4.6	9	Out	-	-	-
Modelled Wild-type *Cn*TYR[a] (with *meta*-L-tyrosine **9**)	-6.0	1	Out	-	-	-
	-5.7	2	Out	-	-	-
	-5.5	3	In	No	5.32 Å	-
	-5.4	4	Out	-	-	-
	-5.2	5	Out	-	-	-
	-5.0	6	Out	-	-	-
	-4.9	7	Out	-	-	-
	-4.7	8	Out	-	-	-
	-4.7	9	Out	-	-	-
Modelled Wild-type *Cn*TYR[a] (with 3-F-L-tyrosine **11**)	-6.4	1	In	No	3.26 Å	-
	-5.8	2	In	No	10.37 Å	-
	-5.7	3	In	No	2.20 Å	-
	-5.3	4	In	Yes	2.50 Å	4.90 Å
	-5.2	5	In	No	3.53 Å	-
	-5.1	6	In	No	3.05 Å	-
	-5.1	7	Out	-	-	-
	-5.1	8	Out	-	-	-
	-5.1	9	Out	-	-	-
Modelled Wild-type *Cn*TYR[a] (with 3-Cl-L-tyrosine **12**)	-6.4	1	Out	-	-	-
	-6.2	2	Out	-	-	-
	-5.8	3	In	No	3.88 Å	-
	-5.7	4	Out	-	-	-
	-4.9	5	Out	-	-	-
	-4.9	6	Out	-	-	-
	-4.9	7	Out	-	-	-
	-4.9	8	Out	-	-	-
	-4.9	9	Out	-	-	-
Modelled G63L (with 3-Cl-L-tyrosine **12**)	-6.4	1	Out	-	-	-
	-5.7	2	Out	-	-	-
	-5.6	3	In	No	9.66 Å	-
	-5.4	4	Out	-	-	-
	-5.2	5	Out	-	-	-

	-5.2	6	Out	-	-	-
	-5.1	7	Out	-	-	-
	-4.9	8	Out	-	-	-
	-4.9	9	Out	-	-	-
Modelled E185L (with 3-Cl-L-tyrosine **12**)	-5.8	1	in	No	4.08 Å	-
	-5.8	2	Out	-	-	-
	-5.4	3	In	No	10.27 Å	-
	-5.1	4	Out	-	-	-
	-5.0	5	Out	-	-	-
	-4.9	6	in	No	N/A	-
	-4.8	7	Out	-		
	-4.8	8	Out	-		
	-4.7	9	Out	-		
Modelled N201S (with L-tyrosine **4**)	-5.9	1	In	Yes	2.28 Å	4.06 Å
	-5.4	2	In	Yes	2.19 Å	4.48 Å
	-5.1	3	In	No	10.40 Å	-
	-4.9	4	In	No	10.29 Å	-
	-4.7	5	Out	-	-	-
	-4.6	6	Out	-	-	-
	-4.5	7	Out	-	-	-
	-4.5	8	Out	-	-	-
	-4.5	9	Out	-	-	-
Modelled N201S (with *meta*-L-tyrosine **9**)	-5.5	1	Out	-	-	-
	-5.2	2	In	Yes	2.41 Å	5.04 Å
	-5.2	3	Out	-	-	-
	-5.2	4	In	No	10.03 Å	-
	-5.1	5	Out	-	-	-
	-5.0	6	Out	-	-	-
	-5.0	7	Out	-	-	-
	-4.9	8	In	No	10.91 Å	-
	-4.9	9	Out	-	-	-
Modelled N201S (with 3-F-L-tyrosine **11**)	-6.1	1	Out	-	-	-
	-6.0	2	In	Yes	2.45 Å	4.11 Å
	-5.9	3	In	No	2.39 Å	-
	-5.4	4	In	No	2.28 Å	-
	-5.2	5	In	No	10. 54 Å	-
	-5.1	6	In	No	3.30 Å	-
	-5.0	7	Out	-	-	-
	-4.9	8	In	Yes	2.59 Å	4.35 Å
	-4.9	9	Out	-	-	-
Modelled N201S (with 3-Cl-L-	-6.4	1	Out	-	-	-
	-5.4	2	In	Yes	2.43 Å	4.73 Å
	-5.0	3	Out	-	-	-

tyrosine **12**)	-4.9	4	Out	-	-	-
	-4.9	5	Out	-	-	-
	-4.8	6	Out	-	-	-
	-4.8	7	Out	-	-	-
	-4.7	8	Out	-	-	-
	-4.7	9	Out	-	-	-
Modelled N132A/L289V (with 3-Cl-L-tyrosine **12**)	-6.2	1	Out	-	-	-
	-5.8	2	In	No	N/A	-
	-5.8	3	Out	-	-	-
	-5.4	4	In	No	10.84 Å	-
	-5.4	5	In	No	2.43 Å	
	-5.2	6	Out	-	-	-
	-5.0	7	In	No	10.78 Å	
	-4.9	8	Out	-	-	-
	-4.7	9	Out	-	-	-
Modelled S161I/L163Y (with 3-Cl-L-tyrosine **12**)	-5.6	1	Out	-	-	-
	-5.4	2	In	No	10.45 Å	-
	-5.4	3	Out	-	-	-
	-5.4	4	Out	-	-	-
	-5.0	5	Out	-	-	-
	-5.0	6	Out	-	-	-
	-4.8	7	Out	-	-	-
	-4.7	8	Out	-	-	-
	-4.6	9	Out	-	-	-

a. The model of wild-type *Cn*TYR was generated by homology modelling (SWISS-MODEL)[12] using the crystal structure of *Bm*TYR (PDB code: 3NPY)[11] as a template.

b. The ranking order followed the affinity energy.

c. Orientation state (see below): If Yes, the *para/meta*-OH on the ligand aromatic ring binds to CuA, meanwhile the near carbon of *para/meta*-OH is orientated towards CuB. Otherwise, the ligand is not in a suitable orientation state. Docking was performed by Autodoc vina.[7] Images were generated using Chimera.[8-10]

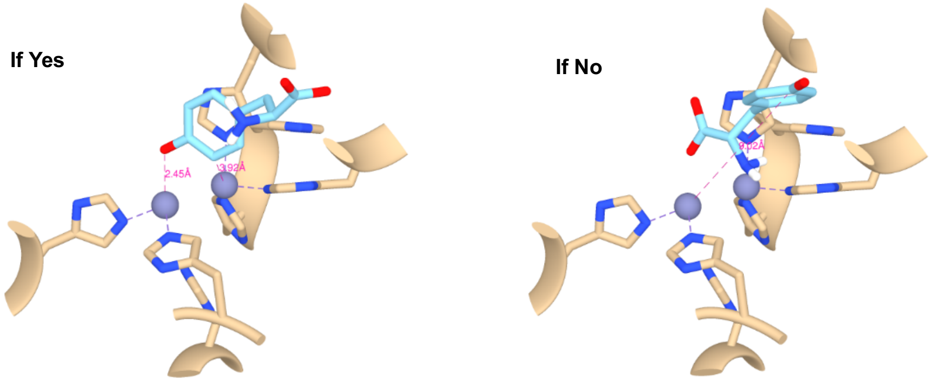

d. Distance 1: the *para/meta*-OH on the ligand aromatic ring to CuA of *Cn*TYRs.

e. Distance 2: the near carbon of *para/meta*-OH on the ligand aromatic ring to CuB of *Cn*TYRs.

11 Protein sequence alignment for *Cn*TYR, *Rs*TYR and *Bm*TYR

a *Cn*TYR and *Bm*TYR

```
BmTYR_AJI23457.1 MSNKYRVRKNVLHLTDTEKRDFVRTVLILKEKGI-------YDRYIAWHGAAGKFHTPPGSDRNAAHMSSAFLPWHREYL 73
CnTYR_EIJ65432.1 -----MVRKNASSLNPIERENFCKAVLTLKNTKIPGHALNRYDEFVAIHFGVTSRERANLPIGDGAHGNSGFLPWHREFL 75

BmTYR_AJI23457.1 LRFERDLQSINPEVTLPYWEWETDAQMQDPSQSQIWSADFMGGNGNPIKDFIVDTGPFAA--GRWTTIDEQGNPSGGLKR 151
CnTYR_EIJ65432.1 CRFEHALKSVDPTVSLPYWDWSSGDTS---DTIDIFNDDFMGPAGTVNSGYFSGTGNSFNSNRPWIVHPSLDQTSPGQ-P 151

BmTYR_AJI23457.1 NFGATKEAPTLPTRDDVLNA------LKITQYDTPPWDMTSQNSFRNQLEGFINGPQLHNRVH-RWVGGQMGVVPTAPND 224
CnTYR_EIJ65432.1 PLGS-----TLIRNSNLLSASTLNYLMDLGEMARDSLNESTYNAFRSTLE----HPP-HNHVHGVTVQGHMGW-MTSPND 220

BmTYR_AJI23457.1 PVFFLHHANVDRIWAVWQIVHR-NQNYQPMKNGPFGQNFRDPMYPWNTTPEDVMNHRKLGYVYDIELRKSKRSS------ 297
CnTYR_EIJ65432.1 PIFFLHHANVDRLWAEWQRTHPGSSNYTPNATEPYGVHLNDPMWPWQGADTTVTTRTHTDSNASLNTLLPSFSTADLVTP 300

BmTYR_AJI23457.1 -------------------------------------------------------------------------------- 297
CnTYR_EIJ65432.1 NDVLDHIQRCGPYDTDPISKPKEFEKIPKEIIKEIIKDKEKEFGDKNPKEIIKEIIKDKEKEFGDKNPKEIIKEIIKDKE 380

BmTYR_AJI23457.1 -------------------------------------------------------------------------------- 297
CnTYR_EIJ65432.1 KEFGDKNPKEIKEIIKDKEKEFGDKNPKEIKEIIKDKEKEFGDKNPKEIIETGDIKIENNKDVVEILSTPSTTVSSPKHP 460

BmTYR_AJI23457.1 -------------------------------------------------------------------------------- 297
CnTYR_EIJ65432.1 KEQSKETLEITNTLFDPLSKINHRLDMLENEIKGTAFIKSTERPNITKRAISKNTSTKKTTRKKTKNTKNTMPKKSNTSK 540

BmTYR_AJI23457.1 ----- 297
CnTYR_EIJ65432.1 RKRIS 545
```

b *Cn*TYR and *Rs*TYR

```
RsTYR_CAD13865.1 MVVRRTVLKAIAGTSVATVFAGKLTGLSAVAADAAPLRVRRNLHGMKMDDPDLSAYREFVGIMKGKDQTQALSWLGFANQ 80
CnTYR_EIJ65432.1 -MVRK-----------------NASSLNPIERE-NFCKAVLTLKNTKIPGHALNRYDEFVAIHFGVTSRERA-------- 53

RsTYR_CAD13865.1 HGTLNGGYKYCPHGDWYFLPWHRGFVLMYERAVAALTGYKTFAMPYWNWTEDRLLPEA------FTA------KTYNGKT 148
CnTYR_EIJ65432.1 ----NLPIGDGAHGNSGFLPWHREFLCRFEHALKSVD--PTVSLPYWDWSSGDTSDTIDIFNDDFMGPAGTVNSGYFSGT 127

RsTYR_CAD13865.1 NPLYVPNRNELTGPYALTDAIVGQKEVMDKIYAETNFEVFGT---SRSVDRSVRPPLVQNSLDPKWVPMGGGNQGILERT 225
CnTYR_EIJ65432.1 GNSFNSNRPWIVHPS-LDQTSPGQPPLGSTLIRNSNLLSASTLNYLMDLGEMAR-----DSLNE---STYNAFRSTLEHP 198

RsTYR_CAD13865.1 PHNTVHN-NIGAFMPTAASPRDPVFMMHHGNIDRVWATWNALGRKNS--------------TDPLWLGMKFPNNYIDPQG 290
CnTYR_EIJ65432.1 PHNHVHGVTVQGHMGWMTSPNDPIFFLHHANVDRLWAEWQRTHPGSSNYTPNATEPYGVHLNDPMWPWQG---------- 268

RsTYR_CAD13865.1 RYYTQGVSDLLSTEALGYRYDVMPRADNKVVNNARAEHLLALFKTGDSVKLAD---HIRLRSVLKGEHPVATAVEPLNSA 367
CnTYR_EIJ65432.1 ------ADTTVTTR-------------THTDSNASLNTLLPSFSTADLVTPNDVLDHIQRCGPY--------DTDPISKP 321

RsTYR_CAD13865.1 VQFEAGT----------------------------------------------VTGALGADVGTGSTTEVVALIKNIRIP 401
CnTYR_EIJ65432.1 KEFEKIPKEIIKEIIKDKEKEFGDKNPKEIIKEIIKDKEKEFGDKNPKEIIKEIIKDKEKEFGDKNPKEIKEIIKDKEKE 401

RsTYR_CAD13865.1 YNVISIRVFVN-LPNANLDVPETDPHF---------------VTSLS-------FLTHAAGHDHHALPSTMVNLTDTLKA 458
CnTYR_EIJ65432.1 FGDKNPKEIKEIIKDKEKEFGDKNPKEIIETGDIKIENNKDVVEILSTPSTTVSSPKHPKEQSKETLEI-TNTLFDPLSK 480

RsTYR_CAD13865.1 LNIRDDNFSINLVAVPQPGVAVESSG---GVTPESIEVAVI----------------------------- 496
CnTYR_EIJ65432.1 INHRLDML-----ENEIKGTAFIKSTERPNITKRAISKNTSTKKTTRKKTKNTKNTMPKKSNTSKRKRIS 545
```

Supplementary Fig. 87 Protein sequence alignment for *Cn*TYR, *Rs*TYR and *Bm*TYR. a DNA alignment for *Cn*TYR and *Bm*TYR. **b** DNA alignment for *Cn*TYR and *Rs*TYR. Black colour represents residues that are identical among the sequences, and grey colour represents residues are similar among the sequences. Residues that were mutated in literatures are shown in red, blue, green, pink and yellow.

12 Docking studies of *Cn*TYR variants with Cl-tyrosine 12

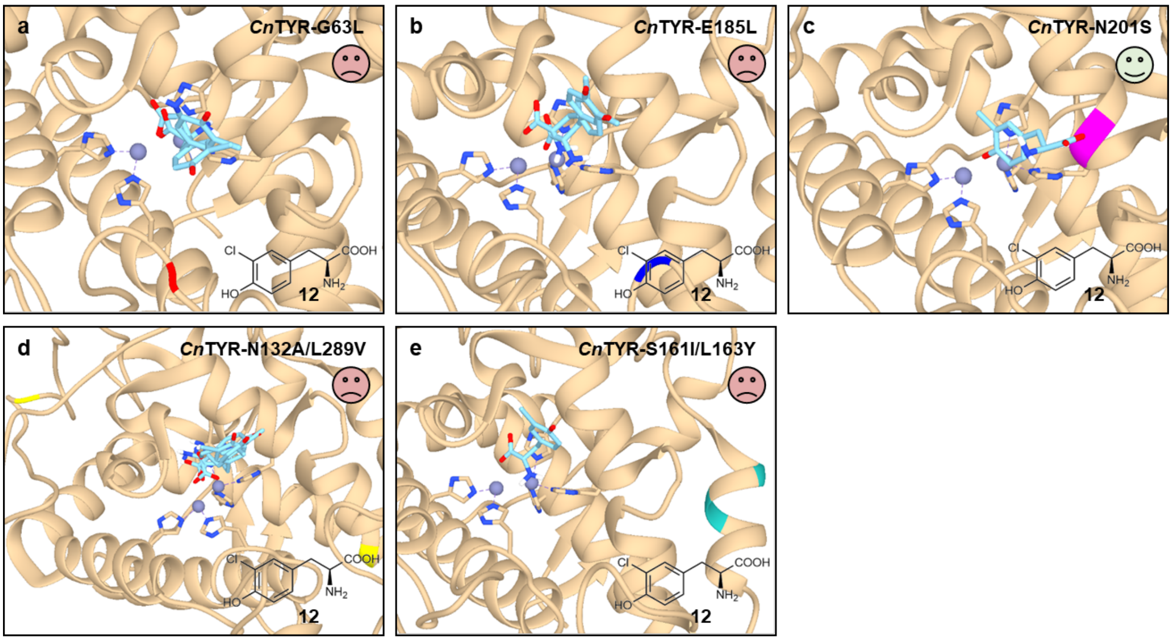

Supplementary Fig. 88 Molecular docking studies with *Cn*TYR variants and Cl-L-tyrosine 12 using AutoDock Vina. a Docking of Cl-L-tyrosine **12** with *Cn*TYR-G36L (residue labeled with red): Cl-L-tyrosine **12** not fits well into the active sites. **b** Docking of Cl-L-tyrosine **12** with *Cn*TYR-E185K (residue labeled with blue): Cl-L-tyrosine **12** not fits well into the active sites. **c** Docking of Cl-L-tyrosine **12** with *Cn*TYR-N201S (residue labeled with pink): Cl-L-tyrosine **12** fits well into the active sites. **d** Docking of Cl-L-tyrosine **12** with *Cn*TYR-N132A/L289V (residue labeled with yellow): Cl-L-tyrosine **12** not fits well into the active sites. **e** Docking of Cl-L-tyrosine **12** with *Cn*TYR-S161I/L163Y (residue labeled with green): Cl-L-tyrosine **12** not fits well into the active sites. The functional histamine residues in the tyrosinase active sites and substrates are shown in stick and ribbon forms. Cl-L-tyrosine **12** is shown in light-blue and enzymes are shown in tan. Using the crystal structure of *Bm*TYR (PDB code: 3NPY)[11] as a template, homology modelling (SWISS-MODEL)[12] was used to develop a model of WT-*Cn*TYR. *Cn*TYR variants were homology modelled by Chimera[9]. Images were generated by Chimera.[8,10]

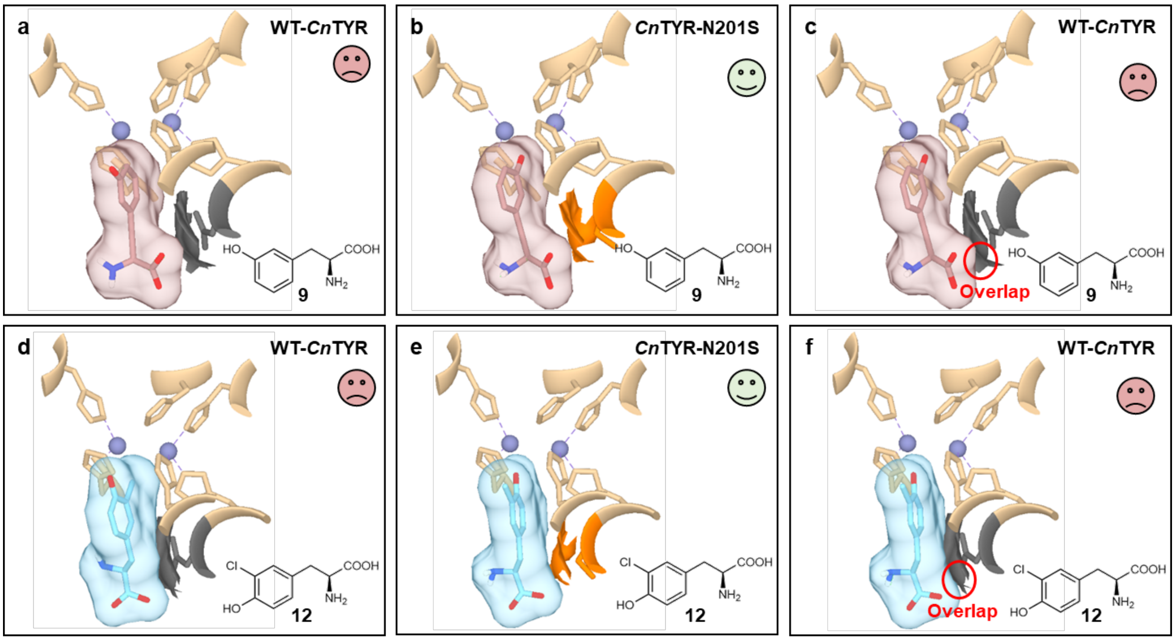

Supplementary Fig. 89 Effect of Asp201 and Ser201 residues of *Cn*TYR on productive orientation of *meta*-L-tyrosine 12 Cl-L-tyrosine 12 using AutoDock Vina. a-c A smaller size Ser201 residue allows compound **9** to adopt a productive orientation with *Cn*TYR-N201S. **d-f** A smaller Ser201 residue allows

compound **12** to adopt a productive orientation; The blue balls represent the di-copper centre of *Cn*TYR. The functional histamine residues in the tyrosinase active sites and substrates are shown in stick and ribbon forms. *meta*-L-tyrosine **9** is shown in rose and Cl-L-tyrosine **12** is shown in light-blue. Enzymes are shown in tan. Using the crystal structure of *Bm*TYR (PDB code: 3NPY)[11] as a template, homology modelling (SWISS-MODEL)[12] was used to develop a model of WT-*Cn*TYR. *Cn*TYR variants were homology modelled by Chimera. Asp201 is shown in dark-grey and Ser201 is shown in orange. *Cn*TYR variants were homology modelled by Chimera[9]. Figures were generated by Chimera.[8,10]

13 Colorimetric selection for *Cn*TYR variants with different substrates

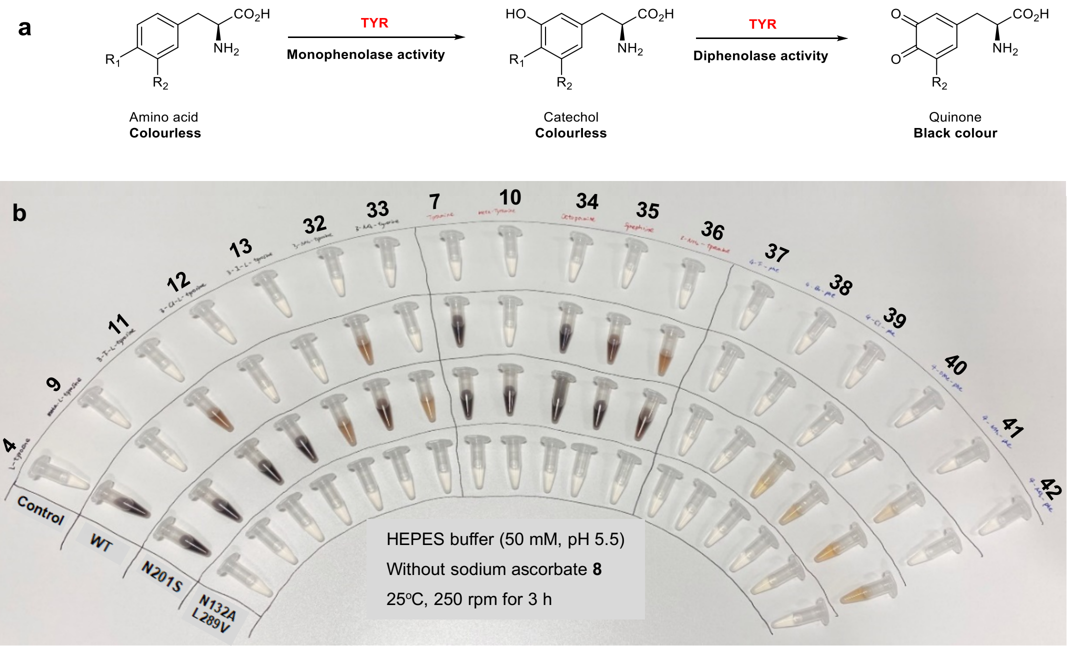

Supplementary Fig. 90 Substrate screening with the wildtype *Cn*TYR, *Cn*TYR-N201S and *Cn*TYR-N132A/L289V using colorimetric reactions. a Scheme for colorimetric reactions using tyrosinases. **b** Substrate screening with the wildtype *Cn*TYR, *Cn*TYR-N201S and *Cn*TYR-N132A/L289V using colorimetric reactions. Reaction conditions: a reaction mixture (1 mL, pH 5) containing HEPES (50 mM), substrates (2.5 mM), $CuSO_4$ (5 μM) and enzyme cell lysates (10% (v/v)) was run at 25 °C for 3 h. The black colour indicated the enzyme activities. Structures of the compounds are shown in supplementary notes 1.

14 Docking study for *Tf*NCS variants with halogenated BIAs

An initial docking study was carried out using the iminium ions **58** and **59** (generated from 3-F-dopamine **60** and 3-Cl-dopamine **61** with phenylacetaldehyde **47**) with WT-*Tf*NCS (PDB: 5N8Q)[6] and *Tf*NCS-A79I (homology modelled). In NCS, Glu110 is believed to be involved in the addition of the amine in dopamine to the aldehyde moiety for imine formation, and the molecular docking suggested that the iminium carbon of both **58** and **59** was in closer proximity to the carbon ortho to the F/Cl on the aromatic ring in A79I. The variant *Tf*NCS-A79I has also been reported recently for its high activities on synthesising non-natural alkaloids, such as chiral 1,1'-disubstituted- and spiro-THIQs using ketones in the NCS reaction, but here it is evidently a useful variant with halogenated dopamines.[13-15]

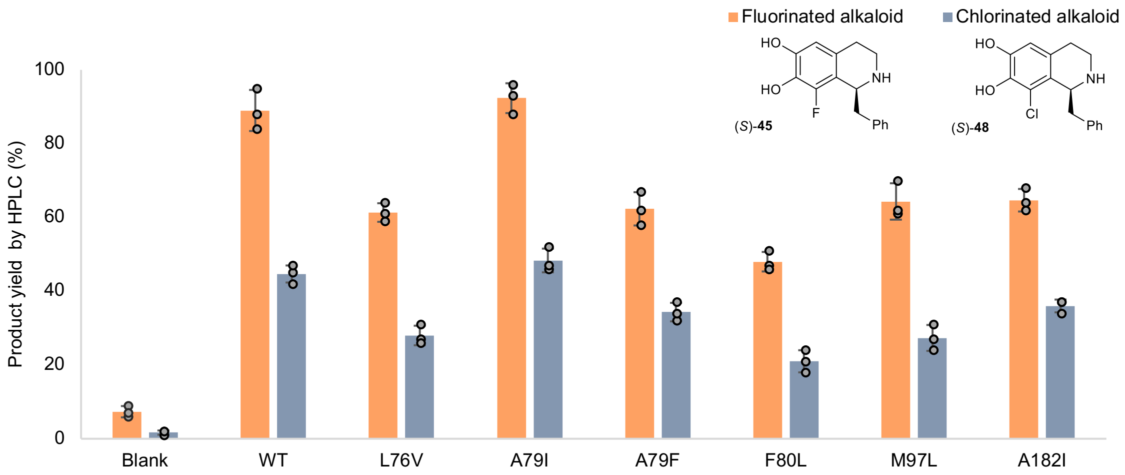

Supplementary Fig. 91 Yields of the halogenated alkaloids (*S*)-45 and (*S*)-48 using *Tf*NCS variants. Orange bars represent the yields of the fluorinated alkaloid (*S*)-**45**. Grey bars represent the yields of the chlorinated alkaloid (*S*)-**48** using the wildtype *Tf*NCS and variants L76V, A79I, A79F, F80L, M97F and A182I. The 100% product yield refers to the theoretical yield. Experiments were performed in triplicates, and measurements were taken from distinct samples. Error bars indicate the standard error of the triplicates.

Global docking protocol for *Tf*NCS variants and intermediates **58/59** was the same as described in SI11. The grid box size for proteins was as follows:

center_x = -16.629
center_y = -8.301
center_z = 21.301

size_x = 40
size_y = 28
size_z = 44

Supplementary Table 2 Global docking analysis of wildtype *Tf*NCS and *Tf*NCS variants with intermediates 58 and 59.

Enzyme	Affinity (kcal/mol)	Ranking[a]	Fold state[b]	Distance [c]
Wild-type (5n8q, with intermediates **58**)	-7.2	1	unfolded	-
	-7.2	2	unfolded	-
	-7.1	3	folded	4.77 Å
	-6.9	4	unfolded	-
	-6.8	5	unfolded	-
	-6.4	6	unfolded	-
	-6.3	7	unfolded	-
	-6.3	8	unfolded	-
	-6.1	9	unfolded	-
Wild-type (5n8q, with intermediates **59**)	-7.6	1	folded	4.68 Å
	-7.5	2	unfolded	-
	-7.2	3	unfolded	-
	-6.9	4	unfolded	-
	-6.9	5	unfolded	-
	-6.9	6	unfolded	-

	-6.8	7	unfolded	-
	-6.7	8	unfolded	-
	-6.7	9	unfolded	-
Modelled A76V (with intermediates **58**)	-7.4	1	folded	4.44 Å
	-7.4	2	unfolded	-
	-7.1	3	unfolded	-
	-7.1	4	unfolded	-
	-6.8	5	unfolded	-
	-6.6	6	unfolded	-
	-6.6	7	unfolded	-
	-6.4	8	unfolded	-
	-6.3	9	unfolded	-
Modelled A76V (with intermediates **59**)	-7.5	1	folded	5.30 Å
	-7.3	2	unfolded	-
	-7.2	3	unfolded	-
	-6.9	4	unfolded	-
	-6.8	5	unfolded	-
	-6.7	6	unfolded	-
	-6.6	7	unfolded	-
	-6.5	8	unfolded	-
	-6.5	9	unfolded	-
Modelled A79I (with intermediates **58**)	-7.8	1	unfolded	-
	-7.6	2	unfolded	-
	-7.5	3	folded	3.90 Å
	-7.3	4	unfolded	-
	-7.1	5	unfolded	-
	-7.1	6	unfolded	-
	-6.9	7	unfolded	-
	-6.9	8	unfolded	-
	-6.8	9	unfolded	-
Modelled A79I (with intermediates **59**)	-7.8	1	folded	3.78 Å
	-7.7	2	unfolded	-
	-7.5	3	unfolded	-
	-7.4	4	unfolded	-
	-7.3	5	unfolded	-
	-7.1	6	unfolded	-
	-6.9	7	unfolded	-
	-6.5	8	unfolded	-
	-6.3	9	unfolded	-
Modelled A79F (with intermediates **58**)	-7.7	1	unfolded	
	-7.6	2	unfolded	-
	-7.5	3	unfolded	-
	-7.4	4	folded	4.25 Å

	-7.2	5	unfolded	-
	-7.2	6	unfolded	-
	-7.1	7	unfolded	-
	-7.0	8	unfolded	-
	-7.0	9	unfolded	-
Modelled A79F (with intermediates **59**)	-8.0	1	unfolded	-
	-7.4	2	unfolded	-
	-7.3	3	folded	4.98 Å
	-7.3	4	unfolded	-
	-7.2	5	unfolded	-
	-7.2	6	unfolded	-
	-7.2	7	unfolded	-
	-7.0	8	unfolded	-
	-6.9	9	unfolded	-
Modelled F80L (with intermediates **58**)	-6.8	1	unfolded	-
	-6.7	2	unfolded	-
	-6.7	3	unfolded	-
	-6.6	4	unfolded	-
	-6.6	5	unfolded	-
	-6.5	6	unfolded	-
	-6.4	7	unfolded	-
	-6.3	8	unfolded	-
	-6.2	9	unfolded	-
Modelled F80L (with intermediates **59**)	-7.2	1	unfolded	-
	-6.9	2	unfolded	-
	-6.6	3	unfolded	-
	-6.4	4	unfolded	-
	-6.4	5	unfolded	-
	-6.3	6	unfolded	-
	-6.0	7	unfolded	-
	-5.9	8	unfolded	-
	-5.9	9	unfolded	-
Modelled M97F (with intermediates **58**)	-7.4	1	unfolded	-
	-7.4	2	folded	4.32 Å
	-7.1	3	unfolded	-
	-7.0	4	unfolded	-
	-7.0	5	unfolded	-
	-6.8	6	unfolded	-
	-6.8	7	unfolded	-
	-6.7	8	unfolded	-
	-6.7	9	unfolded	-
Modelled M97F (with	-7.5	1	unfolded	-
	-7.1	2	unfolded	-

intermediates **59**)	-7.0	3	unfolded	-
	-6.9	4	unfolded	-
	-6.7	5	unfolded	-
	-6.7	6	folded	4.56 Å
	-6.5	7	unfolded	-
	-6.3	8	unfolded	-
	-6.3	9	unfolded	-
Modelled A182I (with intermediates **58**)	-7.5	1	folded	4.03 Å
	-7.4	2	unfolded	-
	-7.4	3	unfolded	-
	-7.3	4	unfolded	-
	-7.2	5	unfolded	-
	-7.1	6	unfolded	-
	-7.0	7	unfolded	-
	-7.0	8	unfolded	-
	-6.8	9	unfolded	-
Modelled A182I (with intermediates **59**)	-7.4	1	unfolded	
	-7.3	2	unfolded	
	-7.1	3	unfolded	
	-6.9	4	unfolded	
	-6.8	5	unfolded	
	-6.8	6	unfolded	
	-6.7	7	folded	4.64 Å
	-6.6	8	unfolded	
	-6.3	9	unfolded	

a The ranking order followed the affinity energy. b Conformation state of the heterocyclic ring. c Distance: the distance between the iminium carbon of both **58** and **59** and the carbon ortho to the F/Cl on the aromatic ring.

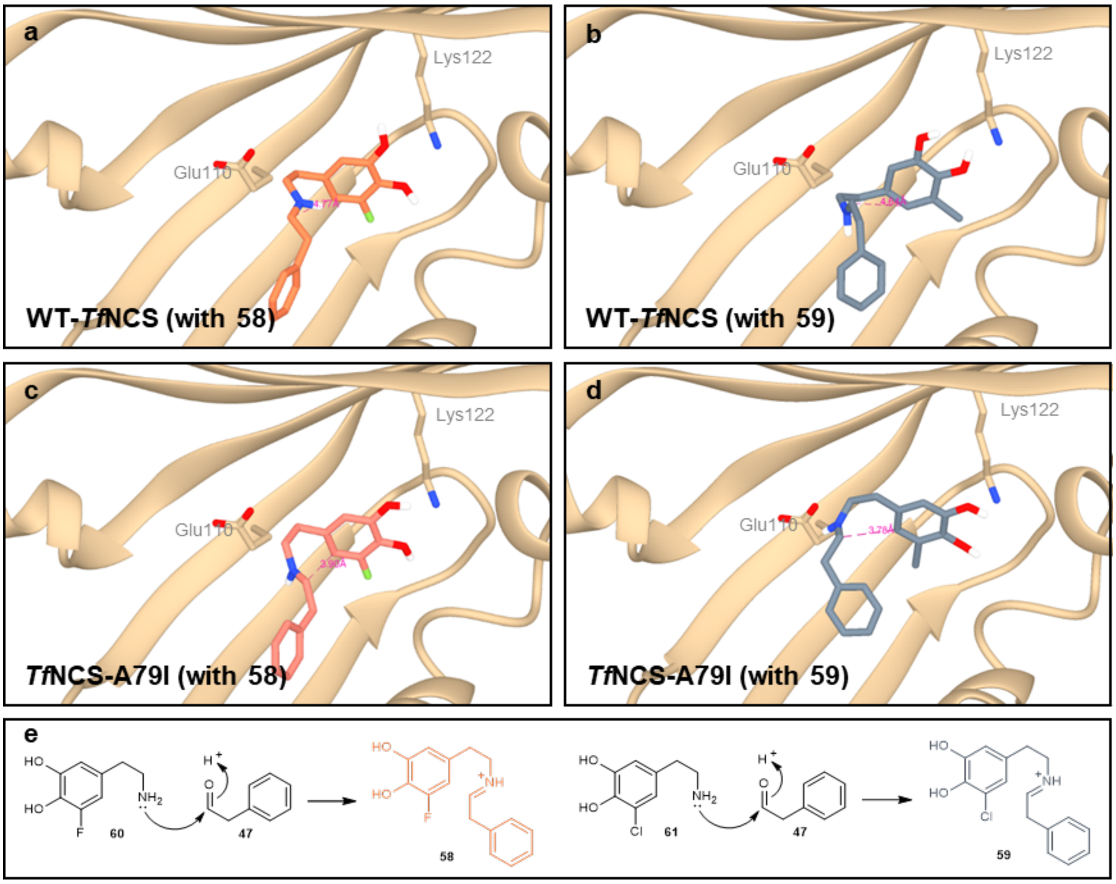

Supplementary Fig. 92 Docking studies of the iminium ions 58 and 59 with the wildtype *Tf*NCS and the variants A79I. a-b Docking of iminium ions **58** and **59** with the WT-*Tf*NCS. **c-d** Docking of iminium ions **58** and **59** with the *Tf*NCS-A79I. **e** Scheme for iminium ions **58** and **59** formation. Enzymes are shown in ribbon forms (tan). Substrates and the key residues of the enzyme are shown in stick forms. Orange colour represents iminium ion **58** formed between 3-F-dopamine **60** and phenylacetaldehyde **47**. Grey colour represents iminium ion **59** formed between 3-Cl-dopamine **61** and **47**. Crystal structure of WT-*Tf*NCS was obtained from PDB database (PDB: 5N8Q)[6] and homology modelled *Tf*NCS-A79I structure is achieved by Chimera.[9] Images were generated by Chimera.[8,10]

Supplementary References

1. Nishihachijo, M., Hirai, Y., Kawano, S., Nishiyama, A., Minami, H., Katayama, T., Yasohara, Y., Sato, F. & Kumagai, H. Asymmetric synthesis of tetrahydroisoquinolines by enzymatic Pictet–Spengler reaction. *Biosci. Biotech. Biochem*. **78**, 701-707 (2014).

2. Maresh, J. J., Crowe, S. O., Ralko, A. A., Aparece, M. D., Murphy, C. M., Krzeskowiec, M. & Mulloweney, M. W. Facile one-pot synthesis of tetrahydroisoquinolines from amino acids via hypochlorite-mediated decarboxylation and Pictet-Spengler condensation. *Tetrahedron Lett.* **55**, 5047-5051 (2014).

3. Pesnot, T., Gershater, M. C., Ward, J. M. & Hailes, H. C. The catalytic potential of *Coptis japonica* NCS2 revealed–Development and utilisation of a fluorescamine-based assay, *Adv. Synth. Catal.* **354**, 2997-3008 (2012).

4. Wang, Y., Tappertzhofen, N., Méndez-Sánchez, D., Bawn, M., Lyu, B., Ward, J. M. & Hailes, H. C. Design and use of *de novo* cascades for the biosynthesis of new benzylisoquinoline alkaloids. *Angew. Chem. Int. Ed.* **58**, 10120-10125 (2019).

5. Subrizi, F., Wang, Y., Thair, B., Méndez-Sánchez, D., Roddan, R., Cárdenas-Fernández, M., Siegrist, J., Richter, M., Andexer, J. N., Ward, J. M. & Hailes, H. C. Multienzyme one-pot cascades incorporating methyltransferases for the strategic diversification of tetrahydroisoquinoline alkaloids. *Angew. Chem. Int. Ed*. **60**, 18673-18679 (2021).

6. Lichman B. R., Sula A., Pesnot T., Hailes H. C., Ward J. M. & Keep N. H. Structural evidence for the dopamine-first mechanism of norcoclaurine synthase. *Biochemistry* **56**, 5274-5277(2017).

7. Trott O. & Olson A. J. AutoDock Vina: improving the speed and accuracy of docking with a new scoring function, efficient optimization and multithreading. *J. Comput. Chem*. **31**, 455-461 (2010).

8. Pettersen, E. F., Goddard, T. D., Huang, C. C., Couch, G. S., Greenblatt, D. M., Meng, E. C. & Ferrin, T. E. UCSF Chimera--a visualization system for exploratory research and analysis. *J. Comput. Chem*. **25**, 1605-1612 (2004).

9. Hertig, S., Goddard, T. D., Johnson, G. T. & Ferrin, T. E. Multidomain Assembler (MDA) generates models of large multidomain proteins. *Biophys J*. **108**, 2097-2102 (2015).

10. Chen, J. E., Huang, C. C., Ferrin & T. E. RRDistMaps: a UCSF Chimera tool for viewing and comparing protein distance maps. *Bioinformatics* **31**, 1484-1486 (2015).

11. Sendovski, M., Kanteev, M., Ben-Yosef, V. S., Adir, N. & Fishman, A. First structures of an active bacterial tyrosinase reveal copper plasticity. *J. Mol. Biol.* **405**, 227-237 (2011).

12. Waterhouse, A., Bertoni, M., Bienert, S., Studer, G., Tauriello, G., Gumienny, R., Heer, F. T., de Beer, T. A. P., Rempfer, C., Bordoli, L., Lepore, R. & Schwede, T. SWISS-MODEL: homology modelling of protein structures and complexes. *Nucleic Acids Res.* **46**, W296-W303 (2018).

13. Lichman, B. R., Zhao, J., Hailes, H. C. & Ward, J. M., Enzyme catalysed Pictet-Spengler formation of chiral 1,1'-disubstituted- and spirotetrahydroisoquinolines. *Nat. Commun*. **8**, 14883 (2019).

14. Erdmann, V., Lichman, B. R., Zhao, J., Simon, R. C., Kroutil, W., Ward, J. M., Hailes, H. C. & Rother, D. Enzymatic and chemoenzymatic three-step cascades for the synthesis of stereochemically complementary trisubstituted tetrahydroisoquinolines. *Angew. Chem. Int. Ed.* **56**, 12503-12507 (2017).

15. Roddan, R., Gygli, G., Sula, A., Méndez-Sánchez, D., Pleiss, J., Ward, J. M., Keep, N. H. & Hailes, H. C. Acceptance and kinetic resolution of α-methyl-substituted aldehydes by norcoclaurine synthases. *ACS Catal.* **9**, 9640-9649 (2019).
