# Supplementary material for: Enzymatic synthesis of benzylisoquinoline alkaloids using a parallel cascade strategy and tyrosinase variants
Source: Nat Commun. 2022 Sep 16;13:5436. doi: 10.1038/s41467-022-33122-1 (PMC9481557; doi:10.1038/s41467-022-33122-1)
Supplement: Supplementary file 2 — Reporting Summary [file 41467_2022_33122_MOESM2_ESM.pdf]

nature portfolio

Corresponding author(s): Helen Hailes

Last updated by author(s): Aug 12, 2022

Reporting Summary

Nature Portfolio wishes to improve the reproducibility of the work that we publish. This form provides structure for consistency and transparency in reporting. For further information on Nature Portfolio policies, see our Editorial Policies and the Editorial Policy Checklist.

Statistics

For all statistical analyses, confirm that the following items are present in the figure legend, table legend, main text, or Methods section.

n/a	Confirmed	
☐	☒	The exact sample size (*n*) for each experimental group/condition, given as a discrete number and unit of measurement
☐	☒	A statement on whether measurements were taken from distinct samples or whether the same sample was measured repeatedly
☒	☐	The statistical test(s) used AND whether they are one- or two-sided<br>*Only common tests should be described solely by name; describe more complex techniques in the Methods section.*
☒	☐	A description of all covariates tested
☒	☐	A description of any assumptions or corrections, such as tests of normality and adjustment for multiple comparisons
☐	☒	A full description of the statistical parameters including central tendency (e.g. means) or other basic estimates (e.g. regression coefficient) AND variation (e.g. standard deviation) or associated estimates of uncertainty (e.g. confidence intervals)
☒	☐	For null hypothesis testing, the test statistic (e.g. *F*, *t*, *r*) with confidence intervals, effect sizes, degrees of freedom and *P* value noted<br>*Give P values as exact values whenever suitable.*
☒	☐	For Bayesian analysis, information on the choice of priors and Markov chain Monte Carlo settings
☒	☐	For hierarchical and complex designs, identification of the appropriate level for tests and full reporting of outcomes
☒	☐	Estimates of effect sizes (e.g. Cohen's *d*, Pearson's *r*), indicating how they were calculated

Our web collection on statistics for biologists contains articles on many of the points above.

Software and code

Policy information about availability of computer code

Data collection	UCFS Chimera version 1.13.1, AutoDock Tools (version 1.5.6), Avogadro (version 1.2.0), Autodock Vina (v.1.2.0), AlphaViewTM FluorChem QTM software (ProteinSimple, US), AlphaImagerTM gel documentation system (ProteinSimple, US)
Data analysis	no unreported custom computer code or algorithm was used

For manuscripts utilizing custom algorithms or software that are central to the research but not yet described in published literature, software must be made available to editors and reviewers. We strongly encourage code deposition in a community repository (e.g. GitHub). See the Nature Portfolio guidelines for submitting code & software for further information.

Data

Policy information about availability of data

All manuscripts must include a data availability statement. This statement should provide the following information, where applicable:

- Accession codes, unique identifiers, or web links for publicly available datasets
- A description of any restrictions on data availability
- For clinical datasets or third party data, please ensure that the statement adheres to our policy

Data to support this work (calibration curves, chemical syntheses and analyses, docking studies) is available in the Supplementary Information and from the corresponding authors upon request.
The protein sequence for enzymes used in this study are available in the Genbank database under accession code list below:

1. Tyrosinase from Candidatus Nitrosopumilus salaria BD31(CnTYR, accession code: EIJ65432.1) [https://www.ncbi.nlm.nih.gov/protein/EIJ65432.1]
2. Tyrosine decarboxylase from Enterococcus faecalis (EfTyrDC, accession code: AFO43338.1) [https://www.ncbi.nlm.nih.gov/protein/AFO43338.1]
3. Transaminase from Chromobacterium violaceum (CvTAm, accession code: AAQ59973.1) [https://www.ncbi.nlm.nih.gov/protein/ AAQ59973.1]
4. (S)-Norcoclaurine synthase from Thalictrum flavum (Δ29TfNCS, accession code: AAR22502.1) [https://www.ncbi.nlm.nih.gov/protein/EIJ65432.1]
5. Catechol-O-methyltransferase from rat liver (RnCOMT, accession code: AAA40881.1) [https://www.ncbi.nlm.nih.gov/protein/ EIJ65432.1]
6. Catechol-O-methyltransferase from Myxococcus xanthus (MxSafC, accession code: AAC44130.1) [https://www.ncbi.nlm.nih.gov/protein/ AAA40881.1]
7. Norcoclaurine 6-O-methyltransferase from Coptis japonica (Cj6OMT, accession code: BAB08004.1) [https://www.ncbi.nlm.nih.gov/protein/ BAB08004.1]
8. S-adenosylmethionine synthetase from E. coli (EcMAT, accession code: AAA24164.1) [https://www.ncbi.nlm.nih.gov/protein/ AAA24164.1]
9. Methylthioadenosine/SAH nucleosidase from E. coli (EcMTAN, accession code: AAB08589.1) [https://www.ncbi.nlm.nih.gov/protein/ AAB08589.1]

The protein sequence for CnTYR variants and TfNCS variants are available in the supplementary information. The crystal structure data for enzymes used in this study are provided in the RCSB Protein Data Bank (PDB) under accession code list below:
1. Crystal structure of tyrosinase from Bacillus megaterium (BmTYR, accession code: 3NPY) [https://www.rcsb.org/structure/3NPY]
2. Crystal structure of (S)-norcoclaurine synthase from Thalictrum flavum (Δ29TfNCS, accession code:5N8Q) [https://www.rcsb.org/structure/5N8Q]

Human research participants

Policy information about studies involving human research participants and Sex and Gender in Research.

Reporting on sex and gender	N/A
Population characteristics	N/A
Recruitment	N/A
Ethics oversight	N/A

Note that full information on the approval of the study protocol must also be provided in the manuscript.

Field-specific reporting

Please select the one below that is the best fit for your research. If you are not sure, read the appropriate sections before making your selection.

☒ Life sciences ☐ Behavioural & social sciences ☐ Ecological, evolutionary & environmental sciences

For a reference copy of the document with all sections, see nature.com/documents/nr-reporting-summary-flat.pdf

Life sciences study design

All studies must disclose on these points even when the disclosure is negative.

Sample size	Enzyme Reactions were performed in triplicates, and measurements (production yields analysed by HPLC) were taken from distinct samples. The sample size was selected on the basis of literature precedent in the field of biocatalysis where routinely such experiments are performed in triplicate to determine reaction yields as this is recognised as providing sound and representative data.
Data exclusions	No data was excluded from the analysis
Replication	All replications were successful. Enzyme reactions were repeated (i.e. performed independently) in triplicate.
Randomization	This is not relevant to our study as the system was designed to build towards halogenated products as targets.
Blinding	Blinding is not relevant to the study. However negative controls for the biocatalytic reactions were performed.

Reporting for specific materials, systems and methods

We require information from authors about some types of materials, experimental systems and methods used in many studies. Here, indicate whether each material, system or method listed is relevant to your study. If you are not sure if a list item applies to your research, read the appropriate section before selecting a response.

Materials & experimental systems

n/a	Involved in the study
☒	☐ Antibodies
☒	☐ Eukaryotic cell lines
☒	☐ Palaeontology and archaeology
☒	☐ Animals and other organisms
☒	☐ Clinical data
☒	☐ Dual use research of concern

Methods

n/a	Involved in the study
☒	☐ ChIP-seq
☒	☐ Flow cytometry
☒	☐ MRI-based neuroimaging
